# Environmental DNA metabarcoding reveals local fish communities in a species-rich coastal sea

**DOI:** 10.1038/srep40368

**Published:** 2017-01-12

**Authors:** Satoshi Yamamoto, Reiji Masuda, Yukuto Sato, Tetsuya Sado, Hitoshi Araki, Michio Kondoh, Toshifumi Minamoto, Masaki Miya

**Affiliations:** 1 Graduate School of Human Development and Environment, Kobe University, Hyogo, Japan; 2 Maizuru Fisheries Research Station, Kyoto University, Kyoto, Japan; 3 Tohoku Medical Megabank Organization, Tohoku University, Miyagi, Japan; 4 Department of Ecology and Environmental Sciences, Natural History Museum and Institute, Chiba, Japan; 5 Research Faculty of Agriculture, Hokkaido University, Hokkaido, Japan; 6 Department of Environmental Solution Technology, Faculty of Science and Technology, Ryukoku University, Shiga, Japan

## Abstract

Environmental DNA (eDNA) metabarcoding has emerged as a potentially powerful tool to assess aquatic community structures. However, the method has hitherto lacked field tests that evaluate its effectiveness and practical properties as a biodiversity monitoring tool. Here, we evaluated the ability of eDNA metabarcoding to reveal fish community structures in species-rich coastal waters. High-performance fish-universal primers and systematic spatial water sampling at 47 stations covering ~11 km^2^ revealed the fish community structure at a species resolution. The eDNA metabarcoding based on a 6-h collection of water samples detected 128 fish species, of which 62.5% (40 species) were also observed by underwater visual censuses conducted over a 14-year period. This method also detected other local fishes (≥23 species) that were not observed by the visual censuses. These eDNA metabarcoding features will enhance marine ecosystem-related research, and the method will potentially become a standard tool for surveying fish communities.

Over 18,000 fish species that use the sea for their reproduction and/or growth have been scientifically described^1^,^2^. At least 20% of species remain to be further described, and thus global marine fish diversity is a vital issue in marine ecology^3^,^4^. In addition, local diversity is also a pivotal issue for the management, conservation, and ecological understanding of marine ecosystems. For example, the spatial accumulation of local fish communities has revealed biodiversity hotspots^5^,^6^, and chronological accumulation has revealed the impact of industrial fishing on both species and communities^7^,^8^. However, investigating marine fish community structures is often difficult because it is restricted by a lack of taxonomic expertise and requires extensive fieldwork. Moreover, there are some marine areas in which it is difficult to observe fish communities (e.g. the deep sea). Therefore, ecological and conservation research often requires costly surveys to examine a specific hypothesis and to reveal the species diversity in specific areas. In addition, given that previous studies suggest that fishing^9^,^10^ and environmental factors^11^ result in precipitous changes in community structure, rapid and continual investigations of marine communities are becoming increasingly essential.

A method that retrieves DNA from environmental samples has been used to explore aquatic organisms in conservation and ecological studies^12^,^13^,^14^,^15^. In such surveillances, genetic material shed by organisms, hereafter referred to as environmental DNA (eDNA), is collected by filtering the water, and species-specific DNA sequences are detected by polymerase chain reaction (PCR) or sequencing. Because this method does not require locating and capturing target organisms during fieldwork, aquatic and semi-aquatic organisms can be detected noninvasively^16^,^17^. In addition, the detection performance of eDNA-based surveys may be higher than that of alternative surveillance methods (e.g. fishing and visual observations)^18^,^19^,^20^,^21^. Therefore, surveillance based on eDNA has been conducted to detect rare or endangered aquatic species^22^,^23^,^24^ and invasive species^25^,^26^,^27^, and also to describe biodiversity^28^,^29^.

The eDNA detection method will become more valuable and essential if it could reveal the entire fish diversity in a given area^30^,^31^. One approach to this end is metabarcoding combined with massively parallel sequencing. One far-sighted study actually detected 15 fishes from seawaters by using two generic and four species-specific primer sets^18^. Kelly *et al*.^32^ also described the species diversity in large mesocosms by metabarcoding using a single generic primer pair. More recently, fish-universal primers for eDNA metabarcoding have been developed, which will promote fish diversity research^33^,^34^. In this regard, the set of fish-universal PCR primers, MiFish^33^, are suitable for eDNA metabarcoding. These MiFish primers amplify hyper-variable regions of the mitochondrial 12S ribosomal RNA (rRNA) gene and enable taxonomic identity to be distinguished mostly at the species level. The fact that eDNA metabarcoding using these primers detected >90% of fish species (i.e. 168 species from 14 orders) in aquarium tanks indicates that the primers can cover phylogenetically diverse species. Moreover, because the amplicon length is ~170 bp, the target region can be PCR-amplified even from degraded genetic material, and the short amplicons are suitable for massively parallel sequencing using MiSeq. Thus, eDNA metabarcoding is becoming an increasingly useful approach for revealing the composition of entire fish communities.

Similar to species-specific detection using the eDNA method, the performances of eDNA metabarcoding and alternative survey methods have been compared. In previous comparative studies, >50% species observed by alternative survey methods were detected by eDNA metabarcoding (e.g. 100% in Thomsen *et al*.^18^, 63–100% in Valentini *et al*.^34^, 92% in Port *et al*.^35^, and 72% in Shaw *et al*.^36^). In addition to detection performance, Port *et al*.^35^ suggested that eDNA metabarcoding can reveal fine-scale community structure. On the other hand, although these previous studies referred to eDNA metabarcoding performance, the efficiency of this technique is still unclear under field conditions because examinations are lacking. In the present study, we evaluated the species detection performance of eDNA metabarcoding and the spatial scale of fish assemblages detected by eDNA metabarcoding. We used eDNA samples collected in a systematic grid survey (Fig. 1) within a species-rich bay^37^. More than 80 fish species have been observed in the bay by underwater visual censuses that would have the highest detection performance among alternative methods^18^. These multiple samples and censuses provide an opportunity to compare the performances of eDNA metabarcoding and visual surveys. Moreover, multipoint sampling using a grid survey enabled us to evaluate the spatial scale of eDNA metabarcoding. Thus, we applied eDNA metabarcoding using MiFish primers (hereafter referred to as MiFish metabarcoding) to the eDNA samples. Our objectives were (1) to compare species detection by underwater visual census and MiFish metabarcoding, and (2) to examine whether eDNA metabarcoding reveals the structure of local fish communities. These approaches will allow us to clarify how efficiently eDNA metabarcoding detects the composition of local fish communities.

## Results

### MiSeq sequencing, assignment, and negative controls

We obtained 8,094,567 MiSeq reads, of which 2,784,828 passed the quality control processes (Table 1; Supplementary Table S1). Of these reads, only 8.1% (226,966 reads) were singletons and the other 2,557,862 reads clustered into 19,260 unique sequences. A majority of the unique sequences (15,972 sequences) were assigned to 147 operational taxonomic units (OTUs). However, after possible contaminant sequences (i.e. sequences that also occurred in the negative controls) were removed and read number cut-off (see Materials and Methods) was applied, the number of OTUs subjected to the following analyses was reduced to 128 (Supplementary Table S2). These 128 OTUs were assigned to fish taxa. Of note, only 3,288 unique sequences were dissimilar to any species in the reference database, and these were not subjected to further data analyses.

### PCR replicates and OTU numbers

We identified species from all 282 PCR samples (47 sampling stations × surface and bottom water samples × three PCR replicates). The mean number of species detected by each PCR amplification was 7.1 and 5.3 for surface and bottom samples, respectively (Fig. 2a). The mean number of detected species from each eDNA sample increased as the number of PCR replications increased to 11.3 and 14.7 for two and three PCR replications for surface samples, and 8.1 and 10.1 for two and three PCR replications for bottom samples, respectively (Fig. 2a). The mean of total species number was 14.7 for surface and 10.1 for bottom samples (Fig. 2b), whereas three PCR replications shared an average of 2.1 and 1.0 species for surface and bottom samples, respectively. The numbers of detected species were lower than the estimated species richness (i.e. Chao1 index) of 31.0 for surface and 24.2 for bottom samples.

### Fish communities detected in the samples

The number of fish species varied among the sampling stations. The highest number of species was detected at St. 16 (39 species from surface and bottom samples) (Fig. 3). The maximum number of fishery target species was also detected at St. 16. Freshwater fishes showed a small variation among stations and the maximum number was detected at St. 27. The minimum number of fish species (12 species) was detected at Sts. 24 and 47, the latter of which is closest to the mouth of the bay. A Mantel correlogram suggested spatial autocorrelation only within ~800 m (Fig. 4). The composition of detected species was also different between vertical positions in the water column (i.e. surface and bottom waters) (Fig. 5). Of all 128 species, 64 (50%) were detected in both surface and bottom samples, whereas the remaining species were detected either in surface (33 species) or bottom (31 species) samples (Fig. 5a). This trend was slightly different among species groups, for example, ~40% of detected freshwater fish were only detected in surface samples. Among all 2,323 species detections from the 282 PCR samples, more detections were from surface samples (1,459 detections) than from bottom samples (864 detections) (Fig. 5b).

### Comparison of underwater visual censuses with MiFish metabarcoding

Over 14 years of underwater visual censuses (140 censuses), 73,709 individuals belonging to 80 species were recorded. Observed numbers for individuals of particular species were highly variable. For instance, 25,413 *Trachurus japonicus* were recorded, whereas only one *Dasyatis akajei* was observed (Fig. 6; Supplementary Table S3). Of the 80 species recorded by visual census, we detected 40 species by MiFish metabarcoding. Considering that seven species of the 80 species were not included in our reference dataset, MiFish metabarcoding detected 54.8% (=40/73) of the visually observed fishes. Furthermore, although MiFish metabarcoding detected species of the genera *Sebastes* and *Takifugu*, these OTUs cannot be assigned at the species level due to their close relatedness, whereas visual observation detected eight species of these genera. Thus, excluding these 8 species, our metabarcoding actually detected 62.5% (=40/65) of the visually detected species. Species accumulation curves based on visual census data showed that 14 rounds of underwater visual census were needed to observe the same number of species detected by MiFish metabarcoding (Fig. 7). For pelagic fishes, MiFish metabarcoding detected 23 species, and 16 rounds of underwater visual censuses would be needed to achieve the same number. Similarly, MiFish metabarcoding detected 17 benthic species, for which 12 rounds of underwater visual censuses would be needed to achieve the same number.

## Discussion

MiFish metabarcoding efficiently detected the composition of the fish assemblage in Maizuru Bay. We detected 112 marine fish species by MiFish metabarcoding of 94 eDNA samples, which were collected within 6 h^37^, whereas 80 fish species were detected by 140 underwater visual censuses over a period of 14 years. Of these 80 visually detected species, 65 species had reference barcodes available for our metabarcoding, and thus MiFish metabarcoding detected 62.5% of species (i.e. 40 species). However, the 14-year underwater visual census would have provided more opportunities to observe rare migratory species that do not necessarily occur in the bay every year, and it is unlikely that these rare species would be present in Maizuru Bay on the day that we collected samples. Indeed, 31 of the visually observed species were represented by <10 individuals over the 14 years. Our MiFish metabarcoding detected 78.6% of fish species for which ≥10 individuals were observed in the visual censuses. Furthermore, we detected these species in a 6-h survey, whereas 14 rounds of underwater visual censuses were needed to observe the same 40 species (Fig. 7a). Moreover, eDNA metabarcoding does not require the taxonomic expertise to visually identify fish species, which is an important consideration in this species-rich bay. In addition, MiFish metabarcoding detected fishes that have not been recorded over 14 years of visual censuses. In particular, at least 23 fishes not observed in our visual censuses have been recorded in or around Maizuru Bay^38^,^39^. The difference in the detected fish community between the visual census and MiFish metabarcoding could be partially attributable to the efficiency of detecting cryptic or rare species and tiny individuals (e.g. larvae). For example, the detection of a demersal fish, *Oplegnathus fasciatus*, from surface samples might suggest the presence of its pelagic larvae (Supplementary Fig. S1). Adult *Oplegnathus fasciatus* are very rare or absent in Maizuru Bay, whereas their pelagic larvae are expected to come to the bay during the research season considering that they spawn from May to August in this area (Reiji Masuda, unpublished data). Although we cannot exclude the possibility that eDNA from rare adult *O. fasciatus* could be detected, detection of their pelagic larvae would be more likely. We also detected demersal marine gobiid species from surface samples, which is consistent with the fact that their pelagic larvae are known to be present in the bay during our sampling season. Thus, eDNA metabarcoding would be able to detect larvae that are often overlooked by alternative survey methods. Although the advantages of eDNA metabarcoding in species detection performance compared with alternative methods have been previously suggested^18^,^34^,^35^,^36^, prior to the present study, no comparisons had been conducted. The present study demonstrated that eDNA metabarcoding is a more time-efficient method for examining a whole fish community than a visual census, having a very high detection performance among the alternative methods^18^. This efficiency is potentially important, particularly in species-rich waters, because a greater effort is required to investigate the whole fish community as the number of species in the community increases. Thus, our comparison demonstrates that eDNA metabarcoding detects marine fishes more efficiently than visual surveys in sites that harbour diverse species.

MiFish metabarcoding also detected species that rarely inhabit Maizuru Bay. At least 27 of the detected species are caught offshore for commercial purposes and are landed at a wholesale fish market on Maizuru Bay. In addition, at least one species, *Oncorhynchus nerka*, has never been observed in Maizuru Bay, nor has it ever been landed. However, these fish are commercially distributed in Japan and their DNA could flow into the bay via sewage from residential areas and fish processing factories. Of course, these species are impossible to be observed visually but MiFish metabarcoding can detect them.

Detection performance was obviously affected by PCR replication number. The number of fish detected at each station increased as the number of PCR replications increased (Fig. 2); three PCR replications revealed twice as many species as a single PCR replication. Although Ficetola *et al*.^40^ have previously suggested that PCR replication is necessary to reduce false negatives, the relationship between PCR replication and the number of detected species has been unclear. The present study suggests that PCR replication is needed to more accurately reconstruct the composition of a fish community. Moreover, given that the number of species detected at each station was considerably lower than the corresponding estimated species richness (Chao1 index), more replications would be necessary for eDNA metabarcoding analysis of a species-rich site like Maizuru Bay.

**Figure 1:**
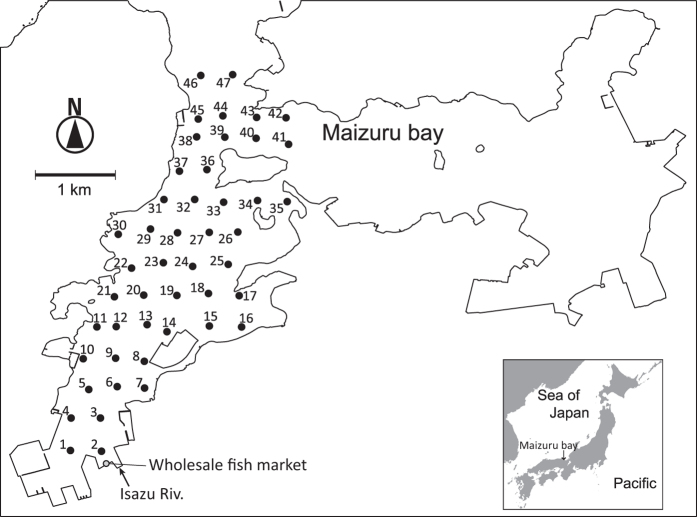
Sampling stations in Maizuru Bay (n = 47). Water sampling was conducted using a bucket for surface water and a van Dorn sampler for bottom water at each station on 18 June 2014. Further details can be found in our previous paper (ref. 37). This map was created using QGIS version 2.8 (http://www.qgis.org/en/site/) based on the Administrative Zones Data (2016) [(**c**) National Spatial Planning and Regional Policy Bureau, Ministry of Land, Infrastructure, Transportation and Tourism (http://nlftp.mlit.go.jp/ksj/gml/datalist/KsjTmplt-N03-v2_3.html), edited by Satoshi Yamamoto].

**Figure 2:**
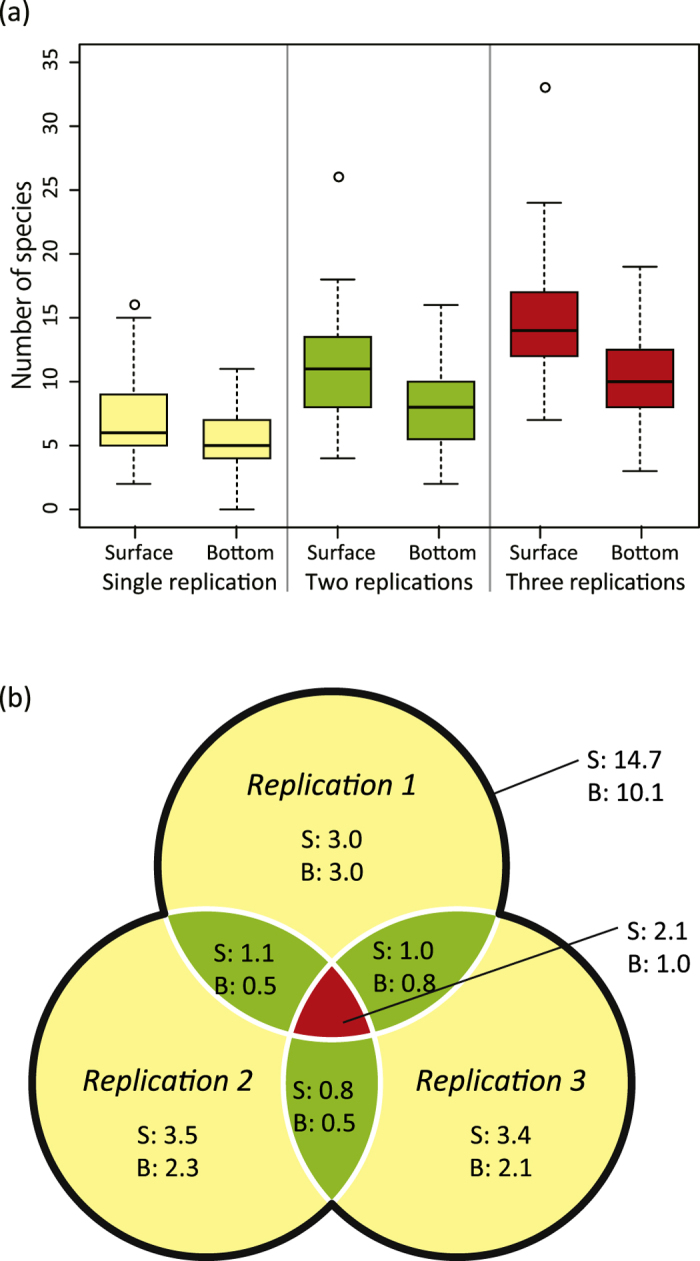
Relationship between the number of detected species and number of PCR replications. (**a**) Boxplots indicate the numbers of detected species from surface and bottom samples. (**b**) The Venn diagram indicates the number of shared species among PCR replications (‘S’ indicates surface samples and ‘B’ indicates bottom samples). Note that ‘S: 14.7 and B: 10.1’ refer to the mean of the total species number.

**Figure 3:**
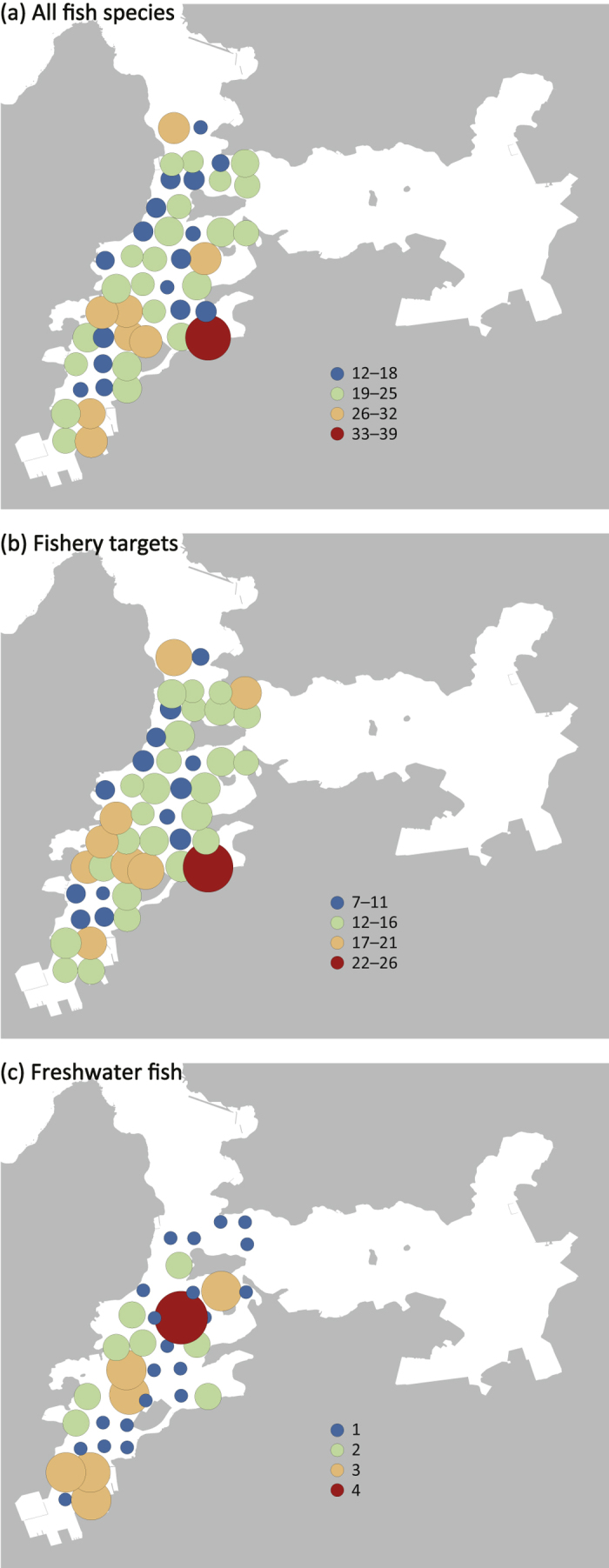
Species diversity detected by MiFish metabarcoding at each station. Circles on each map indicate the number of detected species for (**a**) all fish species, (**b**) fishery targets and (**c**) freshwater fish. Both size and colour reflect the species number. This map was created using QGIS version 2.8 (http://www.qgis.org/en/site/) based on the Administrative Zones Data (2016) [(**c**) National Spatial Planning and Regional Policy Bureau, Ministry of Land, Infrastructure, Transportation and Tourism (http://nlftp.mlit.go.jp/ksj/gml/datalist/KsjTmplt-N03-v2_3.html), edited by Satoshi Yamamoto].

**Figure 4:**
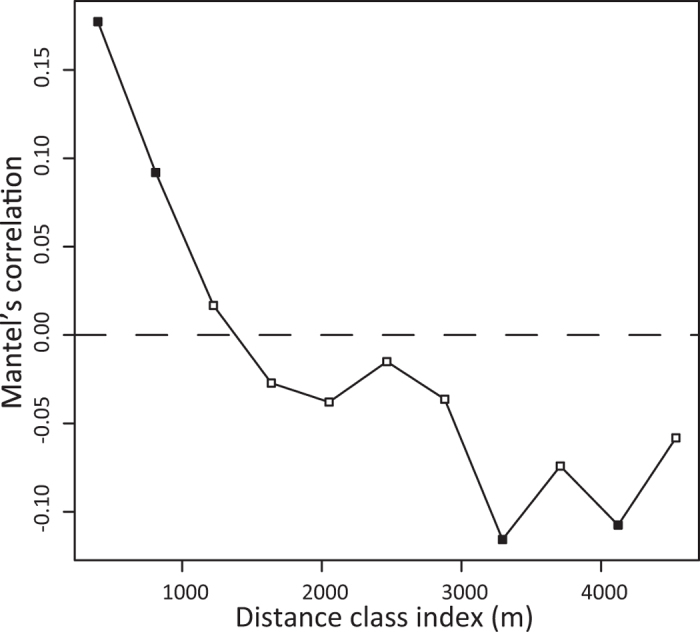
Spatial correlation of fish communities. Mantel correlation between dissimilarity in fish species compositions (Bray-Curtis *β*-diversity) and distance between sampling stations is shown for each distance class. Filled symbols represent significant correlations.

**Figure 5:**
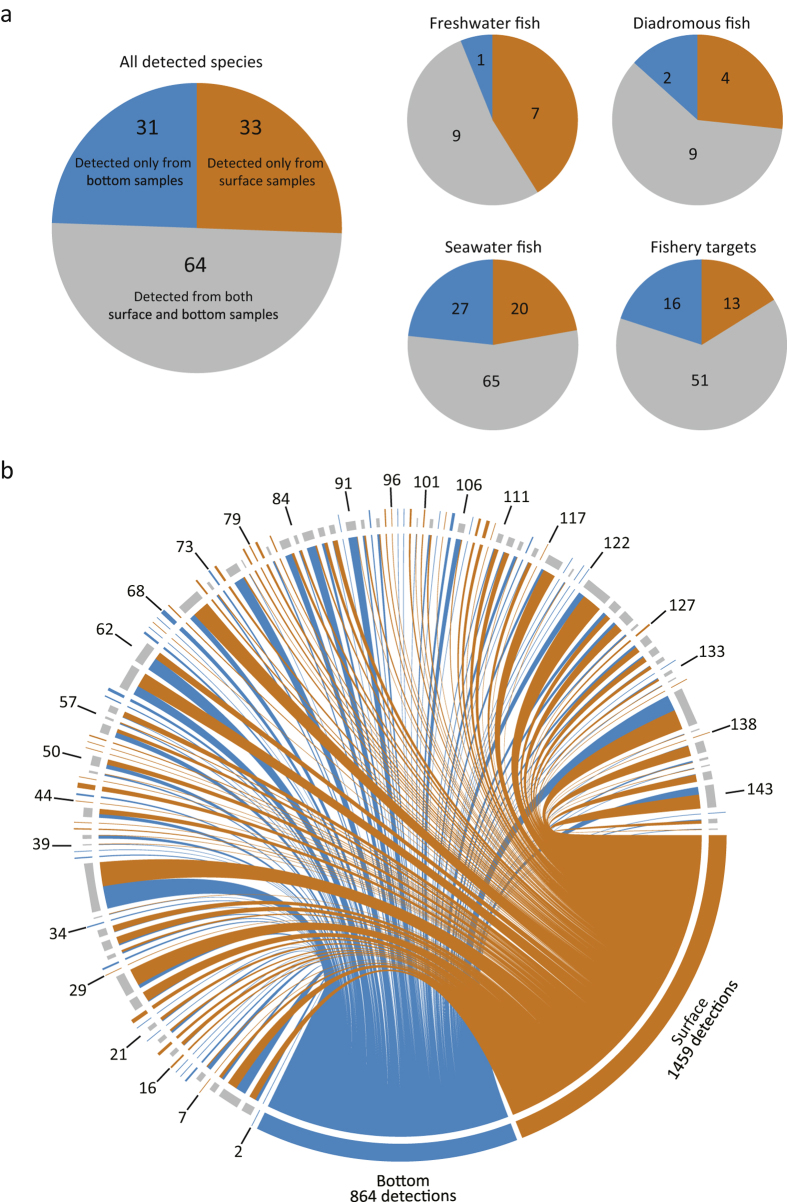
Species detection from surface and bottom samples. The ratio of detected species from only surface samples, only bottom samples, and from both of surface and bottom samples. (**a**) Pie charts indicate species proportion from surface samples (orange), bottom samples (blue), and from both samples (grey). (**b**) The bipartite graph indicates how all 2,323 detections (1,459 and 864 detection events for surface and bottom samples, respectively) were assign to the respective species; tips with broad bars indicate the sample source (i.e. surface or bottom) and the opposite tips indicate species. Species were sorted by operational taxonomic unit (OTU) ID (see Supplementary Table S2) and the numerals around the bipartite graph indicate the OTU ID for approximately every five OTUs.

**Figure 6:**
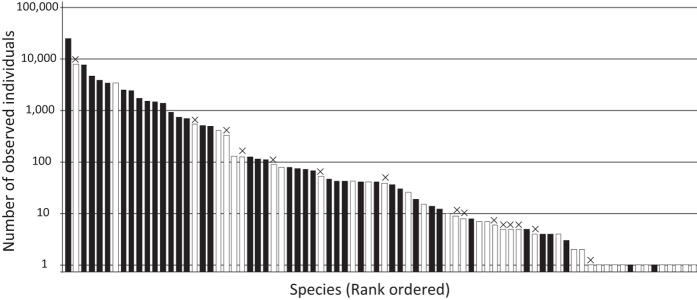
Species abundance as observed in 140 underwater visual censuses. The number of observed individuals is indicated by the bar height. Closed and open bars indicate species detected or not detected by MiFish metabarcoding, respectively. The ‘×’ on the bars indicates species not included in the reference database or indistinguishable due to close relatedness. This bar graph depicts data shown in Supplementary Table S3.

**Figure 7:**
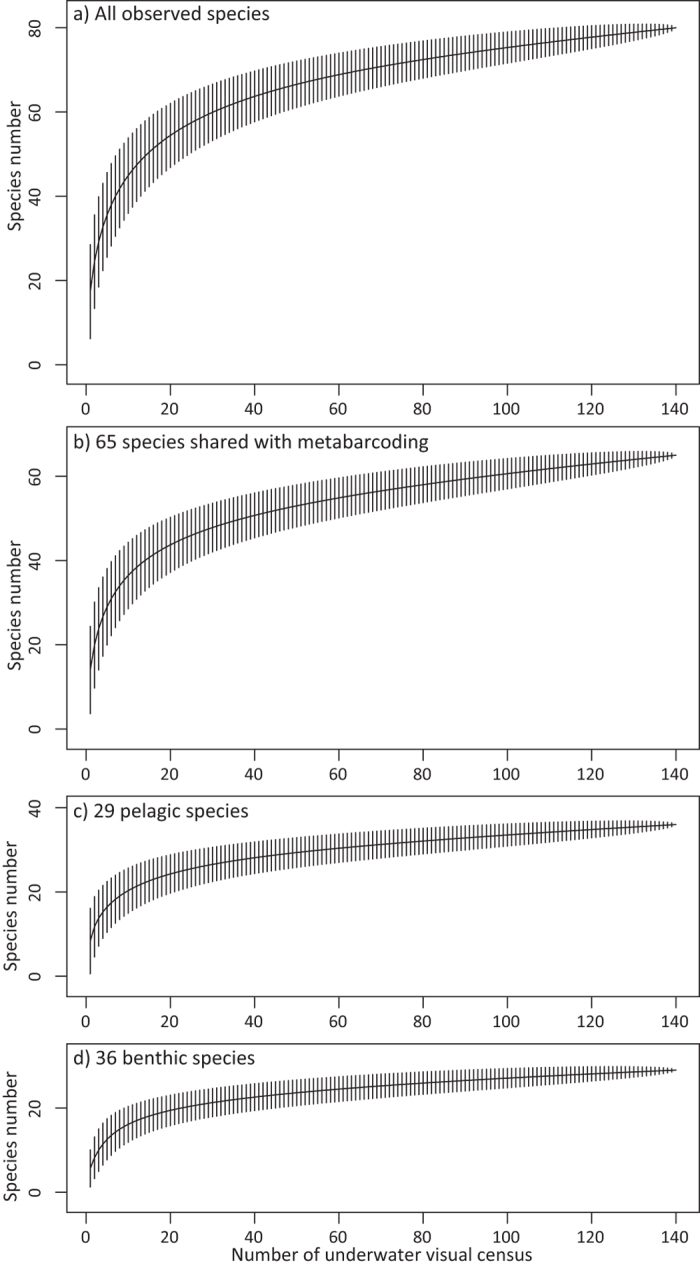
Species accumulation curve of the fish community as observed in 140 underwater visual censuses. (**a**) Species accumulation curve based on all observed species. (**b**–**d**) Species accumulation curve based on only 65 species (**b**), the 29 pelagic species (**c**) and the 36 benthic species (**d**) for which 12S rRNA sequences were included in the reference database. Vertical bars indicate confidence intervals.

We were able to reveal the structure of local fish communities by a metabarcoding analysis of eDNA using systematic grid sampling. This is supported by the detection of species that depend on particular habitats. For example, *Zoarchias major* inhabits seaweed beds and was detected along the west coast of Maizuru Bay where seaweed beds occur (St. 30 and 38; Fig. 1). Although small seaweed beds are scattered in shallow bay areas, the seaweed beds along the west coast are large and continue into the offshore area where we collected water samples (Hideki Sawada, unpublished data). Port *et al*.^35^ suggested that eDNA samples contain information on the fish community from where the samples were collected, and MiFish metabarcoding can reveal the composition of such localised fish communities. However, the accuracy of the estimated species distribution may be lowered by the detection of eDNA transported from distant sources^29^,^41^. In this regard, our correlogram (Fig. 4) suggests a spatial autocorrelation of the detected fish community within ~800 m. We believe that real fish communities also show a spatial autocorrelation, but the suggested range might be partially affected by eDNA transportation. Indeed, some freshwater fish (e.g. *Cyprinus carpio*) were detected from stations that were some distance from rivers (Supplementary Fig. S1) and they were detected from surface water samples (Fig. 5). Because freshwater is lighter than seawater, the riverine water is transported via the sea surface and the genetic material of these freshwater fishes would also be transported to offshore areas (Fig. 3; Supplementary Fig. S1). Although eDNA metabarcoding can provide an approximation of the structure of a local fish community, transported eDNA must be considered as potential noise.

The present study showed that MiFish metabarcoding can reveal the fish diversity in Maizuru Bay. Not only did we detect ~63% species that had been observed over 14 years of underwater visual censuses, our 6-h research also detected >20 species expected to occur but had not been observed by the visual censuses. Some of these species are considered to be at the larval stage and difficult to detect visually. In addition, eDNA metabarcoding also revealed fish communities in localised habitats. This opens up a new approach to revealing the interaction between fish communities and the local environment, and also between fish species within a community. For example, when using conventional methods to survey fish communities, it is difficult to detect pelagic larvae that are an important food web component; however, eDNA metabarcoding is likely to detect these larvae. Thus, eDNA metabarcoding has the potential to more accurately reflect community composition, and may reveal more information about species interactions within a community. However, there are some areas where surveillance based on eDNA metabarcoding could be improved. Firstly, 12S rRNA sequences cannot distinguish some closely related species. Although we detected *Sebastes* spp. and *Takifugu* spp. using MiFish metabarcoding, the species in these genera are impossible to distinguish based on our target 12S rRNA region. Secondly, transported eDNA affects accurate community reconstruction, and, finally, the number of PCR replications should be optimized. These points are potential barriers to ecological research and limit the development of conservation policies based on eDNA surveillance results. However, these problems can be solved by using carefully designed research plans. Moreover, the advantages of eDNA metabarcoding (e.g. time-efficiency and the requirement for less taxonomic expertise) outweigh the current drawbacks.

## Materials and Methods

### eDNA samples

For eDNA metabarcoding using MiSeq, we used eDNA samples collected on 18 June 2014 in west Maizuru Bay, Sea of Japan (35.481°N, 135.332°E)^37^. Briefly, a 1-L water sample was collected from surface waters using a bucket and from bottom waters using a van Dorn sampler from 47 sites in west Maizuru Bay (Fig. 1). Water samples were immediately filtered through a 47-mm diameter GF/F filter (nominal pore size, 0.7 μm; GE Healthcare, Whatman) on the research vessel. Collection of 94 water samples from an ~11 km^2^ area took ~6 h. To minimize cross-contamination, the filter funnels and measuring cups were bleached after every filtration and artificial seawater was filtered (i.e. equipment blank). Total eDNA was extracted from each filter using a DNeasy Blood and Tissue Kit (Qiagen) following ref. 37. To check for cross-contamination during eDNA extraction, eDNA was simultaneously extracted from deionized water (i.e. extraction blank). These eDNA samples and negative control samples were originally obtained to estimate the distribution of the eDNA of Japanese jack mackerel^37^, and we actually collected three filter replicates for all sampling stations. However, as we reported in ref. 37, eDNA in two of the three replicates appeared to be degraded. In the present study, we used the highest quality set of the three replicates (i.e. eDNA samples referred to ‘filter series 1’ in ref. 37).

### Amplicon library and MiSeq sequencing

Amplicon libraries of partial 12S rRNA genes were obtained by PCR amplification using the fish-universal primer pairs MiFish-U and -E^33^. We prepared the amplicon library following the protocol described in ref. 33. The first PCR was performed using the two universal primer pairs. The total reaction volume was 12 μL containing 6.0 μL 2 × KAPA HiFi HotStart ReadyMix (KAPA Biosystems, Wilmington, MA, USA), 3.6 pmol of each MiFish primer, 1 μL template, and H_2_O. The thermal cycle profile was 95 °C for 3 min; 35 cycle of 98 °C for 20 s, 65 °C for 15 s, and 72 °C for 15 s; and 72 °C for 5 min. The first PCR products were diluted 10 times using Milli-Q water, and used as a template for the following PCR. The second PCR was performed to add MiSeq adaptor sequences and 8-bp index sequences^42^ to both amplicon ends. The total reaction volume of the second PCR was also 12 μL containing 6.0 μL 2 × KAPA HiFi HotStart ReadyMix, 3.6 pmol of forward and reverse primers, 1 μL template, and H_2_O. The thermal cycle profile for the second PCR was 95 °C for 3 min; 12 cycle of 98 °C for 20 s and 72 °C for 30 s; and 72 °C for 5 min. PCR amplifications were performed in triplicate for each eDNA sample. As a result, three replications of a single eDNA sample had different index sequences, allowing to assess whether PCR replication increases the number of detected species. All the indexed PCR products were pooled in equal volume and the pooled libraries were purified by agarose gel electrophoresis. Finally, the libraries were sequenced using an Illumina MiSeq v2 Reagent kit for 2 × 150 bp PE (Illumina, San Diego, CA, USA). We note that all samples analysed in the present study were sequenced on a single MiSeq run, and that samples analysed in other research projects were simultaneously sequenced on this run. The total number of reads obtained from the run was 22,917,336. The sequencing reads obtained in the present study were deposited in the DNA Data Bank of Japan (DDBJ) Sequence Read Archive (accession number: DRA004570).

### Quality control and assembling of MiSeq reads

Using the program FastQC^43^, the tails of each MiSeq read were trimmed until the Phred score (an index of the base call quality) of the last base was ≥20. The paired-end reads (R1 and R2 in the MiSeq platform) were then assembled using the program FLASH^44^ when read pairs overlapped by >9 bp. Reads that could not be assembled were discarded. Then, we discarded reads with ambiguous sites (Ns). After that, because the expected amplicon length (target region +127 bp of the first PCR primer sequences) was 297 ± 25 bp, according to comparisons of fish 12S rRNA gene sequences, reads with sequence lengths outside the range 272–322 bp were similarly discarded. In addition, chimeric reads were searched and removed by using UCHIME^45^. Finally, primer sequences were removed from each read using TagCleaner^46^. In this process, we allowed for mismatches in <4 bases in the search for primer sequences because of the following two reasons; the sequence of MiFish-U and -E primer sets show two-base difference in forward primers and one-base difference in reverse primers, and PCR can amplify fish 12S rRNA sequences even if the sequences have a few mismatches in the primer binding sites. When primer sequences were not found, the read was discarded. This data processing was implemented using a custom pipeline program: http://dx.doi.org/10.5061/dryad.54v2q33.

### Taxonomic assignment and OTUs

We used the same pipeline program mentioned above for taxonomic assignment of the obtained sequences. Before taxonomic assignment, MiSeq reads with an identical sequence (i.e. 100% sequence similarity) were assembled using UCLAST^45^ and assembled sequences with ≥2 MiSeq reads were subjected to a BLAST search^47^ (i.e. all singletons were discarded). If the sequence similarity between queries (i.e. unique sequences) and the top BLAST hit was ≥99% and the E-value was less than ≤10^–5^, the assembled sequence was assigned to the top-hit species. Conversely, if the top hit sequence was <99%, the unique sequence was not subjected to the following analyses. Note that ≥99% similarity indicates less than two-base difference between query and reference sequences because the maximum sequence length subjected to taxonomic assignment are 195 bp. This procedure also works as a filter for erroneous reads because erroneous reads are expected never to match the reference species DNA at ≥99% similarity by chance. After BLAST searches, assembled sequences assigned to the same species were clustered, and we considered the clustered sequences as an OTU. The reliability of each assignment was evaluated and classified as HIGH, MODERATE, or LOW following the methods described in ref. 33. Of those three classes, LOW-confidence assignments suggest that the taxon assigned to an OTU cannot be distinguished from second candidate taxon. In the present study, an updated database (i.e. MiFish DB version 19) was used for the BLAST search; the database was composed of MitoFish version 3.05^48^ and our original 12S rRNA datasets version 20160806.

After this automatic taxonomic assignment, we modified the taxonomic assignments because (1) the program may assign a single species name to an OTU, whereas closely related species cannot be distinguished using the 12S rRNA gene, and (2) the program returned evidently incorrect assignments (i.e. assigned species unlikely to occur around Maizuru Bay, to be landed by fishing boats, or to be on sale in markets). Therefore, we modified the taxonomic assignment as follows. (1) Initially, we assigned genus or a higher taxonomic rank to an OTU if the OTU was composed of sequences with LOW-confidence assignment. Taxonomic ranks were determined by comparing the taxonomic status of automatically assigned species and second candidate species. However, even such ambiguously assigned OTUs were re-assigned to a single species if only one species from the taxa occurs around Maizuru Bay. For example, an OTU, ID 59, was ambiguous at the species level and was assigned to the genus rank *Acanthopagrus* sp. However, we were able to assign the species rank *Acanthopagrus schlegelii* to this OTU because *A. schlegelii* is the only species from the genus *Acanthopagrus* in Maizuru Bay. (2) When assigned species were unlikely to occur in Maizuru Bay, we suspected an incorrect assignment because the reference database lacks information on the species that is the actual source of query sequence. In this case, because the initially assigned species would be closely related to the actual source species, we adopted a species that is known to occur in Maizuru Bay and is closely related to the initially assigned species. For example, we obtained *Artediellus neyelovi* by automatic taxonomic assignment. Although this species potentially occurs in Maizuru Bay, according to its known distribution range, it is very rare or possibly absent from our research area. However, a closely related species, *Artediellus fuscimentus*, is observed much more frequently in the bay but was not included in our reference databases. We accordingly adopted *A. fuscimentus* as the taxonomic identity of the OTU in this case. Because this second criterion is somewhat arbitrary, we only applied it when we were certain of the identity. When there were no closely related candidates, we considered that the initially assigned species was detected by metabarcoding.

To remove possible contaminants, we (1) removed sequences that were identical to sequences occurring in the negative controls, and (2) OTUs that have less than 1.5% of sample-total reads were considered to be absent in the sample. We calculated the cut-off value of the read count based on the read count in the negative controls. We obtained 3,778 reads from the negative controls (Supplementary Table S4). Given that 30 negative controls included 3,778 reads, the number of possible contaminant reads were 39,291 in a total of 312 PCR samples (282 eDNA samples and 30 negative controls). This was 1.5% of the total 2,557,862 reads. Therefore, we used 1.5% of sample-total reads as a read number cut-off for each sample.

### Fish community analysis

Detected species were grouped according to their habitat type (marine fish, freshwater fish, diadromous fish, terrestrial animals, and fishery targets). To evaluate the geographic trends of these species groups, the species number detected for each group was depicted on a map using QGIS version 2.8. A spatial autocorrelation of the fish community was examined by Mantel correlogram analysis using the mantel.correlog function in the R package vegan^49^,^50^. In the mantel correlogram analysis, we used the Bray-Curtis *β*-diversity as an index of dissimilarity between communities and performed 999 permutations. To estimate the species richness of each sample, Chao1 indices^51^ were calculated. Species detected from a single PCR replication of the three replications were defined as singletons and species detected from two of the three PCR replications were defined as doubletons in the Chao1 index calculation.

### Underwater visual census

To evaluate the detection efficiency of MiFish metabarcoding, we calculated the detection rate and detection efficiency by comparing MiFish metabarcoding with underwater visual censuses. The detection rate was defined as the proportion of species detected by metabarcoding compared to the species observed by visual censuses, and the detection efficiency is defined as the requisite number of visual censuses until the observed species number becomes the same as the species number detected by MiFish metabarcoding. The underwater visual censuses were conducted along the coast of the Maizuru Fishery Research Station of Kyoto University (35.490°N, 135.368°E). The same investigator (R.M.) has been recording the fish species and abundance along a 600-m-long and 2-m-wide transect every 2 weeks since 2002^52^,^53^. Because our eDNA samples were collected in June, we used visual census data collected from April to August (140 censuses in total, with a mean census time of 44.0 ± 7.1 min). To estimate the detection efficiency, analysis of species accumulation curves was conducted using the specaccum function in the R package vegan.

## Additional Information

**How to cite this article**: Yamamoto, S. *et al*. Environmental DNA metabarcoding reveals local fish communities in a species-rich coastal sea. *Sci. Rep.* **7**, 40368; doi: 10.1038/srep40368 (2017).

**Publisher's note:** Springer Nature remains neutral with regard to jurisdictional claims in published maps and institutional affiliations.

## Supplementary Material

Supplementary Information

## Figures and Tables

**Table 1:** Summary of taxonomic assignment of MiSeq reads.

	Number of MiSeq reads (%)		Number of unique sequence (%)	
Assigned to species	2,347,224	(84.3)	15,972	(5.6)
Not assigned	210,638	(7.6)	3,288	(1.1)
Discarded singleton	226,966	(8.1)	266,966	(93.3)
Total	2,784,828		286,226	
