# Supplementary material for: Environmental DNA metabarcoding reveals local fish communities in a species-rich coastal sea
Source: Sci Rep. 2017 Jan 12;7:40368. doi: 10.1038/srep40368 (PMC5227697; doi:10.1038/srep40368)

Supplementary Information for:

Environmental DNA metabarcoding reveals local fish communities in a species-rich coastal sea

Satoshi Yamamoto[1], Reiji Masuda[2], Yukuto Sato[3], Tetsuya Sado[4], Hitoshi Araki[5], Michio Kondoh[6], Toshifumi Minamoto[1], Masaki Miya[4]

[1] Graduate School of Human Development and Environment, Kobe University, Hyogo, Japan

[2] Maizuru Fisheries Research Station, Kyoto University, Kyoto, Japan

[3] Tohoku Medical Megabank Organization, Tohoku University, Miyagi, Japan

[4] Department of Ecology and Environmental Sciences, Natural History Museum and Institute, Chiba, Japan

[5] Research Faculty of Agriculture, Hokkaido University, Hokkaido, Japan

[6] Department of Environmental Solution Technology, Faculty of Science and Technology, Ryukoku University, Shiga, Japan

Corresponding author:

Satoshi Yamamoto

Email: yamamoto@people.kobe-u.ac.jp

This file contains four Supplementary Tables and Supplementary Figures.

Table S1. Number of reads passed through each quality control process.

Table S2. Detected species by MiFish metabarcoding, taxonomic rank, habitat type, number of MiSeq reads, number of unique sequences, information of the species (F, fishery target; O, observed in underwater visual censuses; L, observed around Maizuru Bay according to the literatures), results of automatic taxonomic assignment, and reason for modification of taxonomic assignment.

Table S3. Species that have been observed by underwater visual censuses for 14 years.

Table S4. Detected species and its number of MiSeq read from negative controls.

Fig. S1. Stations where each species was detected. PCR replication numbers from which each fish species was detected indicated by pie charts on the 47 sampling stations (red, surface samples; blue, bottom samples). Maps were created using R version 3.0.2 (https://www.r-project.org/) based on the Administrative Zones Data (2016) [(c) National Spatial Planning and Regional Policy Bureau, Ministry of Land, Infrastructure, Transportation and Tourism (http://nlftp.mlit.go.jp/ksj/gml/datalist/KsjTmplt-N03-v2_3.html), edited by Satoshi Yamamoto].

Table S1. Number of reads passed through each quality control process.

Sample name	Raw read # (fastq)		(1) Tail-trimming		(2) PE assembly		(3) N removal	(4) Length filtering		(5) Primers removal		% remained
	R1*	R2*	R1*	R2*	# assembled	decrement	N removed	Length-filtered	decrement	# removed	decrement	
Surface_PCR1_St1	60203	60203	60203	60203	42829	17374	42829	35675	7154	22627	13048	37.58%
Surface_PCR1_St2	119651	119651	119651	119651	73609	46042	73609	61244	12365	38326	22918	32.03%
Surface_PCR1_St3	56408	56408	56408	56408	38438	17970	38438	32068	6370	20226	11842	35.86%
Surface_PCR1_St4	77923	77923	77923	77923	53834	24089	53834	44953	8881	28112	16841	36.08%
Surface_PCR1_St5	27817	27817	27817	27817	19308	8509	19308	15892	3416	9898	5994	35.58%
Surface_PCR1_St6	47372	47372	47372	47372	31703	15669	31703	25770	5933	16154	9616	34.10%
Surface_PCR1_St7	50923	50923	50923	50923	33651	17272	33651	27508	6143	17390	10118	34.15%
Surface_PCR1_St8	29615	29615	29615	29615	18626	10989	18626	14988	3638	9413	5575	31.78%
Surface_PCR1_St9	25200	25200	25200	25200	16373	8827	16373	13232	3141	8257	4975	32.77%
Surface_PCR1_St10	43141	43141	43141	43141	27824	15317	27824	22390	5434	13967	8423	32.38%
Surface_PCR1_St11	49906	49906	49906	49906	32854	17052	32854	26846	6008	16688	10158	33.44%
Surface_PCR1_St12	48701	48701	48701	48701	30656	18045	30656	24607	6049	15320	9287	31.46%
Surface_PCR1_St13	30094	30094	30094	30094	19239	10855	19239	15660	3579	9607	6053	31.92%
Surface_PCR1_St14	48544	48544	48544	48544	31263	17281	31263	24992	6271	15609	9383	32.15%
Surface_PCR1_St15	41500	41500	41500	41500	27314	14186	27314	22092	5222	13618	8474	32.81%
Surface_PCR1_St16	85525	85525	85525	85525	55249	30276	55249	44889	10360	28052	16837	32.80%
Surface_PCR1_St17	40030	40030	40030	40030	27753	12277	27753	22994	4759	14567	8427	36.39%
Surface_PCR1_St18	28108	28108	28108	28108	19248	8860	19248	15719	3529	9786	5933	34.82%
Surface_PCR1_St19	31208	31208	31208	31208	20879	10329	20879	16962	3917	10681	6281	34.23%
Surface_PCR1_St20	48209	48209	48209	48209	31771	16438	31771	26358	5413	16490	9868	34.21%
Surface_PCR1_St21	64156	64156	64156	64156	44074	20082	44074	36052	8022	22767	13285	35.49%
Surface_PCR1_St22	40323	40323	40323	40323	27020	13303	27020	22027	4993	13751	8276	34.10%
Surface_PCR1_St23	16255	16255	16255	16255	10589	5666	10589	8549	2040	5369	3180	33.03%
Surface_PCR1_St24	21813	21813	21813	21813	14874	6939	14874	11946	2928	7436	4510	34.09%
Surface_PCR1_St25	37229	37229	37229	37229	25219	12010	25219	20361	4858	12613	7748	33.88%
Surface_PCR1_St26	36075	36075	36075	36075	25232	10843	25232	20536	4696	12806	7730	35.50%
Surface_PCR1_St27	13787	13787	13787	13787	9554	4233	9554	8021	1533	4975	3046	36.08%
Surface_PCR1_St28	10408	10408	10408	10408	6905	3503	6905	5598	1307	3495	2103	33.58%
Surface_PCR1_St29	8935	8935	8935	8935	6015	2920	6015	4604	1411	2823	1781	31.59%
Surface_PCR1_St30	23149	23149	23149	23149	15160	7989	15160	12277	2883	7555	4722	32.64%

Surface_PCR1_St31	11448	11448	11448	11448	7544	3904	7544	5965	1579	3715	2250	32.45%
Surface_PCR1_St32	37856	37856	37856	37856	26227	11629	26227	21306	4921	13187	8119	34.83%
Surface_PCR1_St33	56334	56334	56334	56334	40681	15653	40681	34079	6602	21716	12363	38.55%
Surface_PCR1_St34	22289	22289	22289	22289	15191	7098	15191	12464	2727	7932	4532	35.59%
Surface_PCR1_St35	50191	50191	50191	50191	36010	14181	36010	30089	5921	19003	11086	37.86%
Surface_PCR1_St36	53589	53589	53589	53589	37751	15838	37751	31246	6505	19943	11303	37.21%
Surface_PCR1_St37	13148	13148	13148	13148	9278	3870	9278	7460	1818	4819	2641	36.65%
Surface_PCR1_St38	7658	7658	7658	7658	4984	2674	4984	3710	1274	2288	1422	29.88%
Surface_PCR1_St39	28552	28552	28552	28552	20663	7889	20663	16868	3795	10761	6107	37.69%
Surface_PCR1_St40	42405	42405	42405	42405	30048	12357	30048	24167	5881	15144	9023	35.71%
Surface_PCR1_St41	18254	18254	18254	18254	12343	5911	12343	9823	2520	6142	3681	33.65%
Surface_PCR1_St42	25386	25386	25386	25386	17897	7489	17897	14854	3043	9419	5435	37.10%
Surface_PCR1_St43	20847	20847	20847	20847	14240	6607	14240	11335	2905	7149	4186	34.29%
Surface_PCR1_St44	82219	82219	82219	82219	57303	24916	57303	47347	9956	29760	17587	36.20%
Surface_PCR1_St45	32040	32040	32040	32040	21483	10557	21483	17301	4182	10864	6437	33.91%
Surface_PCR1_St46	35331	35331	35331	35331	22626	12705	22626	18253	4373	11516	6737	32.59%
Surface_PCR1_St47	23741	23741	23741	23741	16504	7237	16504	13317	3187	8376	4941	35.28%
NegativeControl_DNAextraction1	3978	3978	3978	3978	1622	2356	1622	523	1099	344	179	8.65%
NegativeControl_DNAextraction2	4849	4849	4849	4849	1549	3300	1549	1	1548	1		0.02%
NegativeControl_DNAextraction3	6676	6676	6676	6676	2267	4409	2267		2267			0.00%
NegativeControl_DNAextraction4	7384	7384	7384	7384	2345	5039	2345	100	2245	60	40	0.81%
NegativeControl_PCR_first_round	5751	5751	5751	5751	2095	3656	2095	49	2046	27	22	0.47%
Surface_PCR2_St1	64531	64531	64531	64531	45629	18902	45629	38345	7284	24525	13820	38.00%
Surface_PCR2_St2	115431	115431	115431	115431	73018	42413	73018	60993	12025	39040	21953	33.82%
Surface_PCR2_St3	65124	65124	65124	65124	43827	21297	43827	36634	7193	23437	13197	35.99%
Surface_PCR2_St4	74342	74342	74342	74342	51388	22954	51388	43127	8261	27709	15418	37.27%
Surface_PCR2_St5	23737	23737	23737	23737	16696	7041	16696	13692	3004	8743	4949	36.83%
Surface_PCR2_St6	48276	48276	48276	48276	33440	14836	33440	27584	5856	17549	10035	36.35%
Surface_PCR2_St7	43897	43897	43897	43897	27799	16098	27799	22893	4906	14632	8261	33.33%
Surface_PCR2_St8	24407	24407	24407	24407	15664	8743	15664	12529	3135	7961	4568	32.62%
Surface_PCR2_St9	38631	38631	38631	38631	24907	13724	24907	20402	4505	12576	7826	32.55%
Surface_PCR2_St10	33486	33486	33486	33486	22762	10724	22762	18728	4034	11478	7250	34.28%
Surface_PCR2_St11	50452	50452	50452	50452	32207	18245	32207	25997	6210	15788	10209	31.29%
Surface_PCR2_St12	53217	53217	53217	53217	33359	19858	33359	27263	6096	16564	10699	31.13%
Surface_PCR2_St13	24728	24728	24728	24728	16516	8212	16516	13141	3375	8058	5083	32.59%
Surface_PCR2_St14	43113	43113	43113	43113	28179	14934	28179	22500	5679	13799	8701	32.01%
Surface_PCR2_St15	33085	33085	33085	33085	21612	11473	21612	17263	4349	10632	6631	32.14%

Surface_PCR2_St16	59059	59059	59059	59059	39181	19878	39181	31316	7865	19049	12267	32.25%
Surface_PCR2_St17	48538	48538	48538	48538	34636	13902	34636	28766	5870	18398	10368	37.90%
Surface_PCR2_St18	29612	29612	29612	29612	19274	10338	19274	15417	3857	9610	5807	32.45%
Surface_PCR2_St19	22615	22615	22615	22615	15211	7404	15211	12205	3006	7640	4565	33.78%
Surface_PCR2_St20	33698	33698	33698	33698	20712	12986	20712	16984	3728	10761	6223	31.93%
Surface_PCR2_St21	44001	44001	44001	44001	29415	14586	29415	24233	5182	15169	9064	34.47%
Surface_PCR2_St22	36966	36966	36966	36966	24432	12534	24432	19753	4679	12470	7283	33.73%
Surface_PCR2_St23	7369	7369	7369	7369	4641	2728	4641	3472	1169	2232	1240	30.29%
Surface_PCR2_St24	21973	21973	21973	21973	14064	7909	14064	11496	2568	7158	4338	32.58%
Surface_PCR2_St25	39295	39295	39295	39295	28182	11113	28182	23216	4966	14827	8389	37.73%
Surface_PCR2_St26	24884	24884	24884	24884	17544	7340	17544	14422	3122	9168	5254	36.84%
Surface_PCR2_St27	13669	13669	13669	13669	8810	4859	8810	7092	1718	4493	2599	32.87%
Surface_PCR2_St28	11804	11804	11804	11804	7222	4582	7222	5533	1689	3465	2068	29.35%
Surface_PCR2_St29	12357	12357	12357	12357	8154	4203	8154	6539	1615	4172	2367	33.76%
Surface_PCR2_St30	21800	21800	21800	21800	13962	7838	13962	11196	2766	7086	4110	32.50%
Surface_PCR2_St31	5052	5052	5052	5052	2854	2198	2854	2040	814	1324	716	26.21%
Surface_PCR2_St32	22421	22421	22421	22421	15868	6553	15868	13137	2731	8254	4883	36.81%
Surface_PCR2_St33	38070	38070	38070	38070	27072	10998	27072	22480	4592	14326	8154	37.63%
Surface_PCR2_St34	21208	21208	21208	21208	14252	6956	14252	11607	2645	7376	4231	34.78%
Surface_PCR2_St35	33787	33787	33787	33787	23552	10235	23552	19331	4221	12382	6949	36.65%
Surface_PCR2_St36	33394	33394	33394	33394	21495	11899	21495	17263	4232	11167	6096	33.44%
Surface_PCR2_St37	10542	10542	10542	10542	6914	3628	6914	5234	1680	3360	1874	31.87%
Surface_PCR2_St38	15806	15806	15806	15806	10321	5485	10321	7957	2364	5008	2949	31.68%
Surface_PCR2_St39	21931	21931	21931	21931	15198	6733	15198	12417	2781	7894	4523	35.99%
Surface_PCR2_St40	30558	30558	30558	30558	21023	9535	21023	16621	4402	10607	6014	34.71%
Surface_PCR2_St41	21805	21805	21805	21805	15440	6365	15440	12776	2664	8096	4680	37.13%
Surface_PCR2_St42	19871	19871	19871	19871	14364	5507	14364	11872	2492	7554	4318	38.02%
Surface_PCR2_St43	7052	7052	7052	7052	4464	2588	4464	3298	1166	2082	1216	29.52%
Surface_PCR2_St44	53512	53512	53512	53512	38601	14911	38601	32590	6011	20568	12022	38.44%
Surface_PCR2_St45	20619	20619	20619	20619	13738	6881	13738	11020	2718	6953	4067	33.72%
Surface_PCR2_St46	25745	25745	25745	25745	18136	7609	18136	14484	3652	9177	5307	35.65%
Surface_PCR2_St47	16346	16346	16346	16346	10704	5642	10704	8649	2055	5484	3165	33.55%
NegativeControl_DNAextraction1	2748	2748	2748	2748	827	1921	827	6	821	2	4	0.07%
NegativeControl_DNAextraction2	3906	3906	3906	3906	1371	2535	1371	445	926	285	160	7.30%
NegativeControl_DNAextraction3	4713	4713	4713	4713	1749	2964	1749	421	1328	261	160	5.54%
NegativeControl_DNAextraction4	3385	3385	3385	3385	1010	2375	1010	188	822	116	72	3.43%
NegativeControl_PCR_second_round	5797	5797	5797	5797	2603	3194	2603	1124	1479	731	393	12.61%

Surface_PCR3_St1	67130	67130	67130	67130	47260	19870	47260	39528	7732	25005	14523	37.25%
Surface_PCR3_St2	117331	117331	117331	117331	69839	47492	69839	58033	11806	36371	21662	31.00%
Surface_PCR3_St3	63451	63451	63451	63451	41770	21681	41770	34954	6816	22091	12863	34.82%
Surface_PCR3_St4	71710	71710	71710	71710	45681	26029	45681	37823	7858	23606	14217	32.92%
Surface_PCR3_St5	41038	41038	41038	41038	27667	13371	27667	22851	4816	14236	8615	34.69%
Surface_PCR3_St6	35956	35956	35956	35956	23655	12301	23655	19373	4282	12028	7345	33.45%
Surface_PCR3_St7	57711	57711	57711	57711	36033	21678	36033	29931	6102	18882	11049	32.72%
Surface_PCR3_St8	28673	28673	28673	28673	18430	10243	18430	14829	3601	9440	5389	32.92%
Surface_PCR3_St9	63294	63294	63294	63294	45090	18204	45090	38010	7080	24005	14005	37.93%
Surface_PCR3_St10	60819	60819	60819	60819	41034	19785	41034	33636	7398	21328	12308	35.07%
Surface_PCR3_St11	73268	73268	73268	73268	50316	22952	50316	42182	8134	26805	15377	36.58%
Surface_PCR3_St12	83506	83506	83506	83506	56906	26600	56906	47157	9749	29968	17189	35.89%
Surface_PCR3_St13	35156	35156	35156	35156	24241	10915	24241	19874	4367	12639	7235	35.95%
Surface_PCR3_St14	99653	99653	99653	99653	65939	33714	65939	55024	10915	34975	20049	35.10%
Surface_PCR3_St15	40639	40639	40639	40639	28090	12549	28090	22946	5144	14561	8385	35.83%
Surface_PCR3_St16	104581	104581	104581	104581	70530	34051	70530	58593	11937	37223	21370	35.59%
Surface_PCR3_St17	49846	49846	49846	49846	36044	13802	36044	30128	5916	19328	10800	38.78%
Surface_PCR3_St18	14922	14922	14922	14922	9619	5303	9619	7721	1898	4865	2856	32.60%
Surface_PCR3_St19	31007	31007	31007	31007	21873	9134	21873	18170	3703	11518	6652	37.15%
Surface_PCR3_St20	16674	16674	16674	16674	10673	6001	10673	8912	1761	5717	3195	34.29%
Surface_PCR3_St21	36577	36577	36577	36577	25590	10987	25590	21558	4032	13808	7750	37.75%
Surface_PCR3_St22	36761	36761	36761	36761	26433	10328	26433	22141	4292	14215	7926	38.67%
Surface_PCR3_St23	12712	12712	12712	12712	8788	3924	8788	7328	1460	4701	2627	36.98%
Surface_PCR3_St24	24443	24443	24443	24443	17372	7071	17372	14595	2777	9274	5321	37.94%
Surface_PCR3_St25	26307	26307	26307	26307	18387	7920	18387	15147	3240	9550	5597	36.30%
Surface_PCR3_St26	32573	32573	32573	32573	22558	10015	22558	18423	4135	11613	6810	35.65%
Surface_PCR3_St27	13711	13711	13711	13711	9459	4252	9459	7861	1598	4907	2954	35.79%
Surface_PCR3_St28	9727	9727	9727	9727	6239	3488	6239	5108	1131	3172	1936	32.61%
Surface_PCR3_St29	11756	11756	11756	11756	7626	4130	7626	6242	1384	3895	2347	33.13%
Surface_PCR3_St30	21228	21228	21228	21228	13909	7319	13909	11334	2575	7098	4236	33.44%
Surface_PCR3_St31	16535	16535	16535	16535	11545	4990	11545	9382	2163	5775	3607	34.93%
Surface_PCR3_St32	25982	25982	25982	25982	16873	9109	16873	13689	3184	8406	5283	32.35%
Surface_PCR3_St33	52655	52655	52655	52655	38092	14563	38092	32062	6030	20483	11579	38.90%
Surface_PCR3_St34	13116	13116	13116	13116	8305	4811	8305	6709	1596	4284	2425	32.66%
Surface_PCR3_St35	36561	36561	36561	36561	26162	10399	26162	21904	4258	13966	7938	38.20%
Surface_PCR3_St36	39756	39756	39756	39756	28472	11284	28472	23729	4743	15205	8524	38.25%
Surface_PCR3_St37	12060	12060	12060	12060	8443	3617	8443	6798	1645	4284	2514	35.52%

Surface_PCR3_St38	13989	13989	13989	13989	9984	4005	9984	8289	1695	5202	3087	37.19%
Surface_PCR3_St39	35562	35562	35562	35562	26047	9515	26047	21737	4310	13722	8015	38.59%
Surface_PCR3_St40	34169	34169	34169	34169	24259	9910	24259	19816	4443	12547	7269	36.72%
Surface_PCR3_St41	17056	17056	17056	17056	12136	4920	12136	10073	2063	6417	3656	37.62%
Surface_PCR3_St42	32195	32195	32195	32195	23271	8924	23271	19601	3670	12280	7321	38.14%
Surface_PCR3_St43	14115	14115	14115	14115	10049	4066	10049	8383	1666	5252	3131	37.21%
Surface_PCR3_St44	80688	80688	80688	80688	58175	22513	58175	48733	9442	30680	18053	38.02%
Surface_PCR3_St45	12601	12601	12601	12601	8724	3877	8724	7065	1659	4416	2649	35.04%
Surface_PCR3_St46	25150	25150	25150	25150	17429	7721	17429	14115	3314	8770	5345	34.87%
Surface_PCR3_St47	21958	21958	21958	21958	15221	6737	15221	12657	2564	7987	4670	36.37%
NegativeControl_DNAextraction1	1073	1073	1073	1073	260	813	260	5	255	3	2	0.28%
NegativeControl_DNAextraction2	1314	1314	1314	1314	375	939	375		375			0.00%
NegativeControl_DNAextraction3	1831	1831	1831	1831	709	1122	709	339	370	190	149	10.38%
NegativeControl_DNAextraction4	5333	5333	5333	5333	3154	2179	3154	2444	710	1501	943	28.15%
NegativeControl_PCR_third_round	1114	1114	1114	1114	292	822	292	1	291	1		0.09%
Bottom_PCR1_St1	22686	22686	22686	22686	15521	7165	15521	12602	2919	8057	4545	35.52%
Bottom_PCR1_St2	77746	77746	77746	77746	44858	32888	44858	37080	7778	23086	13994	29.69%
Bottom_PCR1_St3	43596	43596	43596	43596	30466	13130	30466	24976	5490	15691	9285	35.99%
Bottom_PCR1_St4	33385	33385	33385	33385	23022	10363	23022	18730	4292	11673	7057	34.96%
Bottom_PCR1_St5	10852	10852	10852	10852	7207	3645	7207	5626	1581	3504	2122	32.29%
Bottom_PCR1_St6	10072	10072	10072	10072	4580	5492	4580	3323	1257	2019	1304	20.05%
Bottom_PCR1_St7	36726	36726	36726	36726	25516	11210	25516	20721	4795	13128	7593	35.75%
Bottom_PCR1_St8	62355	62355	62355	62355	42458	19897	42458	33691	8767	20875	12816	33.48%
Bottom_PCR1_St9	13397	13397	13397	13397	9524	3873	9524	7679	1845	4871	2808	36.36%
Bottom_PCR1_St10	7067	7067	7067	7067	3968	3099	3968	2883	1085	1829	1054	25.88%
Bottom_PCR1_St11	16775	16775	16775	16775	11580	5195	11580	9385	2195	5999	3386	35.76%
Bottom_PCR1_St12	22883	22883	22883	22883	15895	6988	15895	12824	3071	8196	4628	35.82%
Bottom_PCR1_St13	30287	30287	30287	30287	21310	8977	21310	17256	4054	11247	6009	37.13%
Bottom_PCR1_St14	10311	10311	10311	10311	6866	3445	6866	5478	1388	3535	1943	34.28%
Bottom_PCR1_St15	21711	21711	21711	21711	15217	6494	15217	12294	2923	7902	4392	36.40%
Bottom_PCR1_St16	35171	35171	35171	35171	23530	11641	23530	19464	4066	12457	7007	35.42%
Bottom_PCR1_St17	27424	27424	27424	27424	19063	8361	19063	15379	3684	9579	5800	34.93%
Bottom_PCR1_St18	5159	5159	5159	5159	3201	1958	3201	2425	776	1522	903	29.50%
Bottom_PCR1_St19	9223	9223	9223	9223	6108	3115	6108	4890	1218	3065	1825	33.23%
Bottom_PCR1_St20	41986	41986	41986	41986	29122	12864	29122	23976	5146	15249	8727	36.32%
Bottom_PCR1_St21	18538	18538	18538	18538	12474	6064	12474	10158	2316	6317	3841	34.08%
Bottom_PCR1_St22	21610	21610	21610	21610	14668	6942	14668	11605	3063	7050	4555	32.62%

Bottom_PCR1_St23	6753	6753	6753	6753	4232	2521	4232	3176	1056	1973	1203	29.22%
Bottom_PCR1_St24	6819	6819	6819	6819	4265	2554	4265	3078	1187	1918	1160	28.13%
Bottom_PCR1_St25	44721	44721	44721	44721	30899	13822	30899	26446	4453	16956	9490	37.92%
Bottom_PCR1_St26	38329	38329	38329	38329	27329	11000	27329	22762	4567	14668	8094	38.27%
Bottom_PCR1_St27	11389	11389	11389	11389	7626	3763	7626	6049	1577	3779	2270	33.18%
Bottom_PCR1_St28	17806	17806	17806	17806	11898	5908	11898	9417	2481	6015	3402	33.78%
Bottom_PCR1_St29	9019	9019	9019	9019	6030	2989	6030	4637	1393	2912	1725	32.29%
Bottom_PCR1_St30	40632	40632	40632	40632	29218	11414	29218	24249	4969	15231	9018	37.49%
Bottom_PCR1_St31	8037	8037	8037	8037	5586	2451	5586	4564	1022	2888	1676	35.93%
Bottom_PCR1_St32	8193	8193	8193	8193	5467	2726	5467	4335	1132	2792	1543	34.08%
Bottom_PCR1_St33	21602	21602	21602	21602	14497	7105	14497	11626	2871	7165	4461	33.17%
Bottom_PCR1_St34	25819	25819	25819	25819	17687	8132	17687	14097	3590	8622	5475	33.39%
Bottom_PCR1_St35	14513	14513	14513	14513	9925	4588	9925	7929	1996	4886	3043	33.67%
Bottom_PCR1_St36	19705	19705	19705	19705	13298	6407	13298	10604	2694	6484	4120	32.91%
Bottom_PCR1_St37	15225	15225	15225	15225	9959	5266	9959	7601	2358	4730	2871	31.07%
Bottom_PCR1_St38	21168	21168	21168	21168	13921	7247	13921	10873	3048	6479	4394	30.61%
Bottom_PCR1_St39	27151	27151	27151	27151	18451	8700	18451	14763	3688	9128	5635	33.62%
Bottom_PCR1_St40	20072	20072	20072	20072	12114	7958	12114	9446	2668	5456	3990	27.18%
Bottom_PCR1_St41	29486	29486	29486	29486	21270	8216	21270	17751	3519	11217	6534	38.04%
Bottom_PCR1_St42	14838	14838	14838	14838	10101	4737	10101	8088	2013	5118	2970	34.49%
Bottom_PCR1_St43	24648	24648	24648	24648	17433	7215	17433	14247	3186	8975	5272	36.41%
Bottom_PCR1_St44	20065	20065	20065	20065	14273	5792	14273	11718	2555	7526	4192	37.51%
Bottom_PCR1_St45	23035	23035	23035	23035	15392	7643	15392	12625	2767	7972	4653	34.61%
Bottom_PCR1_St46	56436	56436	56436	56436	39558	16878	39558	32389	7169	20291	12098	35.95%
Bottom_PCR1_St47	12777	12777	12777	12777	8599	4178	8599	6786	1813	4239	2547	33.18%
NegativeControl_DNAextraction5	1865	1865	1865	1865	655	1210	655	36	619	20	16	1.07%
NegativeControl_DNAextraction6	2267	2267	2267	2267	664	1603	664	1	663	1		0.04%
NegativeControl_DNAextraction7	2243	2243	2243	2243	704	1539	704		704			0.00%
NegativeControl_DNAextraction8	2611	2611	2611	2611	1066	1545	1066	481	585	248	233	9.50%
NegativeControl_DNAextraction9	2423	2423	2423	2423	711	1712	711		711			0.00%
Bottom_PCR2_St1	21980	21980	21980	21980	15521	6459	15521	12546	2975	7959	4587	36.21%
Bottom_PCR2_St2	91022	91022	91022	91022	56658	34364	56658	47377	9281	30290	17087	33.28%
Bottom_PCR2_St3	39774	39774	39774	39774	28335	11439	28335	23427	4908	14979	8448	37.66%
Bottom_PCR2_St4	10220	10220	10220	10220	7106	3114	7106	5886	1220	3647	2239	35.68%
Bottom_PCR2_St5	13601	13601	13601	13601	9829	3772	9829	8060	1769	5055	3005	37.17%
Bottom_PCR2_St6	4606	4606	4606	4606	3002	1604	3002	2437	565	1529	908	33.20%
Bottom_PCR2_St7	25905	25905	25905	25905	18927	6978	18927	15267	3660	9817	5450	37.90%

Bottom_PCR2_St8	57026	57026	57026	57026	39857	17169	39857	33120	6737	21043	12077	36.90%
Bottom_PCR2_St9	9042	9042	9042	9042	5757	3285	5757	4411	1346	2642	1769	29.22%
Bottom_PCR2_St10	1329	1329	1329	1329	638	691	638	407	231	258	149	19.41%
Bottom_PCR2_St11	8165	8165	8165	8165	5528	2637	5528	4467	1061	2719	1748	33.30%
Bottom_PCR2_St12	24458	24458	24458	24458	16833	7625	16833	13489	3344	8389	5100	34.30%
Bottom_PCR2_St13	29320	29320	29320	29320	19501	9819	19501	15458	4043	9513	5945	32.45%
Bottom_PCR2_St14	19156	19156	19156	19156	12281	6875	12281	10185	2096	6324	3861	33.01%
Bottom_PCR2_St15	21503	21503	21503	21503	15016	6487	15016	12135	2881	7631	4504	35.49%
Bottom_PCR2_St16	49669	49669	49669	49669	32021	17648	32021	25995	6026	16259	9736	32.73%
Bottom_PCR2_St17	15268	15268	15268	15268	10983	4285	10983	9173	1810	5686	3487	37.24%
Bottom_PCR2_St18	7057	7057	7057	7057	4708	2349	4708	3812	896	2347	1465	33.26%
Bottom_PCR2_St19	6165	6165	6165	6165	4204	1961	4204	3424	780	2149	1275	34.86%
Bottom_PCR2_St20	13794	13794	13794	13794	9678	4116	9678	7891	1787	4977	2914	36.08%
Bottom_PCR2_St21	17080	17080	17080	17080	12045	5035	12045	9833	2212	6123	3710	35.85%
Bottom_PCR2_St22	22339	22339	22339	22339	15124	7215	15124	12447	2677	7511	4936	33.62%
Bottom_PCR2_St23	18073	18073	18073	18073	12574	5499	12574	10448	2126	6630	3818	36.68%
Bottom_PCR2_St24	11353	11353	11353	11353	7402	3951	7402	5888	1514	3809	2079	33.55%
Bottom_PCR2_St25	41662	41662	41662	41662	27758	13904	27758	24428	3330	15754	8674	37.81%
Bottom_PCR2_St26	18590	18590	18590	18590	13287	5303	13287	10827	2460	6873	3954	36.97%
Bottom_PCR2_St27	12205	12205	12205	12205	8351	3854	8351	6837	1514	4401	2436	36.06%
Bottom_PCR2_St28	18283	18283	18283	18283	12801	5482	12801	10472	2329	6840	3632	37.41%
Bottom_PCR2_St29	5241	5241	5241	5241	3505	1736	3505	2740	765	1743	997	33.26%
Bottom_PCR2_St30	21242	21242	21242	21242	14347	6895	14347	11917	2430	7605	4312	35.80%
Bottom_PCR2_St31	4519	4519	4519	4519	3087	1432	3087	2528	559	1581	947	34.99%
Bottom_PCR2_St32	8455	8455	8455	8455	5816	2639	5816	4680	1136	2940	1740	34.77%
Bottom_PCR2_St33	11075	11075	11075	11075	7530	3545	7530	6236	1294	4005	2231	36.16%
Bottom_PCR2_St34	9689	9689	9689	9689	6715	2974	6715	5500	1215	3508	1992	36.21%
Bottom_PCR2_St35	12988	12988	12988	12988	9302	3686	9302	7551	1751	4871	2680	37.50%
Bottom_PCR2_St36	21654	21654	21654	21654	15278	6376	15278	12598	2680	8012	4586	37.00%
Bottom_PCR2_St37	10156	10156	10156	10156	7112	3044	7112	5742	1370	3688	2054	36.31%
Bottom_PCR2_St38	19570	19570	19570	19570	13509	6061	13509	11166	2343	6976	4190	35.65%
Bottom_PCR2_St39	9564	9564	9564	9564	6668	2896	6668	5423	1245	3520	1903	36.80%
Bottom_PCR2_St40	18433	18433	18433	18433	12711	5722	12711	10334	2377	6332	4002	34.35%
Bottom_PCR2_St41	18579	18579	18579	18579	13022	5557	13022	10819	2203	6730	4089	36.22%
Bottom_PCR2_St42	31674	31674	31674	31674	22343	9331	22343	17794	4549	10985	6809	34.68%
Bottom_PCR2_St43	17072	17072	17072	17072	11625	5447	11625	9586	2039	5915	3671	34.65%
Bottom_PCR2_St44	29617	29617	29617	29617	20663	8954	20663	17059	3604	10665	6394	36.01%

Bottom_PCR2_St45	21279	21279	21279	21279	15062	6217	15062	12468	2594	7676	4792	36.07%
Bottom_PCR2_St46	49805	49805	49805	49805	34199	15606	34199	27611	6588	16991	10620	34.12%
Bottom_PCR2_St47	16496	16496	16496	16496	11362	5134	11362	9185	2177	5759	3426	34.91%
NegativeControl_DNAextraction5	1072	1072	1072	1072	281	791	281	6	275	3	3	0.28%
NegativeControl_DNAextraction6	3061	3061	3061	3061	1721	1340	1721	1194	527	715	479	23.36%
NegativeControl_DNAextraction7	2624	2624	2624	2624	1459	1165	1459	1171	288	719	452	27.40%
NegativeControl_DNAextraction8	762	762	762	762	200	562	200	50	150	25	25	3.28%
NegativeControl_DNAextraction9	919	919	919	919	240	679	240	16	224	12	4	1.31%
Bottom_PCR3_St1	8232	8232	8232	8232	4573	3659	4573	3646	927	2199	1447	26.71%
Bottom_PCR3_St2	79657	79657	79657	79657	46975	32682	46975	38661	8314	23569	15092	29.59%
Bottom_PCR3_St3	30651	30651	30651	30651	20360	10291	20360	16621	3739	10192	6429	33.25%
Bottom_PCR3_St4	12220	12220	12220	12220	7914	4306	7914	6336	1578	3878	2458	31.73%
Bottom_PCR3_St5	5154	5154	5154	5154	3071	2083	3071	2297	774	1401	896	27.18%
Bottom_PCR3_St6	6676	6676	6676	6676	3708	2968	3708	2728	980	1618	1110	24.24%
Bottom_PCR3_St7	12558	12558	12558	12558	7958	4600	7958	6243	1715	3946	2297	31.42%
Bottom_PCR3_St8	42829	42829	42829	42829	28964	13865	28964	23394	5570	14204	9190	33.16%
Bottom_PCR3_St9	11315	11315	11315	11315	7801	3514	7801	6434	1367	4040	2394	35.70%
Bottom_PCR3_St10	4116	4116	4116	4116	2448	1668	2448	1913	535	1180	733	28.67%
Bottom_PCR3_St11	15699	15699	15699	15699	10467	5232	10467	8592	1875	5368	3224	34.19%
Bottom_PCR3_St12	9870	9870	9870	9870	6574	3296	6574	5249	1325	3324	1925	33.68%
Bottom_PCR3_St13	14201	14201	14201	14201	9428	4773	9428	7525	1903	4789	2736	33.72%
Bottom_PCR3_St14	10586	10586	10586	10586	6675	3911	6675	5448	1227	3406	2042	32.17%
Bottom_PCR3_St15	14068	14068	14068	14068	9604	4464	9604	7648	1956	4781	2867	33.98%
Bottom_PCR3_St16	28361	28361	28361	28361	19011	9350	19011	15687	3324	9898	5789	34.90%
Bottom_PCR3_St17	11545	11545	11545	11545	7522	4023	7522	6090	1432	3791	2299	32.84%
Bottom_PCR3_St18	3795	3795	3795	3795	1838	1957	1838	1378	460	894	484	23.56%
Bottom_PCR3_St19	1991	1991	1991	1991	752	1239	752	520	232	325	195	16.32%
Bottom_PCR3_St20	7958	7958	7958	7958	4650	3308	4650	3765	885	2392	1373	30.06%
Bottom_PCR3_St21	11206	11206	11206	11206	7282	3924	7282	5893	1389	3760	2133	33.55%
Bottom_PCR3_St22	16885	16885	16885	16885	11139	5746	11139	9068	2071	5523	3545	32.71%
Bottom_PCR3_St23	5543	5543	5543	5543	3091	2452	3091	2405	686	1549	856	27.95%
Bottom_PCR3_St24	3633	3633	3633	3633	1596	2037	1596	1098	498	691	407	19.02%
Bottom_PCR3_St25	16972	16972	16972	16972	10874	6098	10874	9294	1580	5831	3463	34.36%
Bottom_PCR3_St26	16931	16931	16931	16931	12053	4878	12053	9768	2285	6220	3548	36.74%
Bottom_PCR3_St27	6508	6508	6508	6508	4178	2330	4178	3485	693	2196	1289	33.74%
Bottom_PCR3_St28	10226	10226	10226	10226	6622	3604	6622	5395	1227	3455	1940	33.79%
Bottom_PCR3_St29	8508	8508	8508	8508	5289	3219	5289	4110	1179	2596	1514	30.51%

Bottom_PCR3_St30	10984	10984	10984	10984	7518	3466	7518	6140	1378	3877	2263	35.30%
Bottom_PCR3_St31	6766	6766	6766	6766	4382	2384	4382	3373	1009	2158	1215	31.89%
Bottom_PCR3_St32	6347	6347	6347	6347	3926	2421	3926	3149	777	1958	1191	30.85%
Bottom_PCR3_St33	3817	3817	3817	3817	2048	1769	2048	1609	439	1024	585	26.83%
Bottom_PCR3_St34	13623	13623	13623	13623	9453	4170	9453	7764	1689	5066	2698	37.19%
Bottom_PCR3_St35	12673	12673	12673	12673	8748	3925	8748	7260	1488	4580	2680	36.14%
Bottom_PCR3_St36	6007	6007	6007	6007	3553	2454	3553	2647	906	1637	1010	27.25%
Bottom_PCR3_St37	11233	11233	11233	11233	7077	4156	7077	5697	1380	3647	2050	32.47%
Bottom_PCR3_St38	12208	12208	12208	12208	7876	4332	7876	6269	1607	3924	2345	32.14%
Bottom_PCR3_St39	15496	15496	15496	15496	10815	4681	10815	8853	1962	5831	3022	37.63%
Bottom_PCR3_St40	7290	7290	7290	7290	4124	3166	4124	3267	857	2065	1202	28.33%
Bottom_PCR3_St41	18245	18245	18245	18245	12227	6018	12227	10067	2160	6410	3657	35.13%
Bottom_PCR3_St42	13965	13965	13965	13965	9028	4937	9028	7145	1883	4508	2637	32.28%
Bottom_PCR3_St43	22378	22378	22378	22378	15315	7063	15315	12413	2902	7836	4577	35.02%
Bottom_PCR3_St44	12807	12807	12807	12807	7990	4817	7990	6434	1556	4106	2328	32.06%
Bottom_PCR3_St45	17212	17212	17212	17212	11600	5612	11600	9388	2212	5952	3436	34.58%
Bottom_PCR3_St46	36157	36157	36157	36157	24461	11696	24461	19954	4507	12738	7216	35.23%
Bottom_PCR3_St47	11956	11956	11956	11956	7658	4298	7658	6275	1383	3883	2392	32.48%
NegativeControl_DNAextraction5	2895	2895	2895	2895	338	2557	338	2	336		2	0.00%
NegativeControl_DNAextraction6	2831	2831	2831	2831	475	2356	475	4	471	3	1	0.11%
NegativeControl_DNAextraction7	2598	2598	2598	2598	300	2298	300	1	299	1		0.04%
NegativeControl_DNAextraction8	2433	2433	2433	2433	347	2086	347	2	345	2		0.08%
NegativeControl_DNAextraction9	2482	2482	2482	2482	278	2204	278	1	277	1		0.04%
Total	2783095	2783095	2783095	2783095	1854418	928677	1854418	1504145	350273	944893	559252	33.95%

* R1 and R2 are MiSeq read sequenced from 5' and 3' ends, respectively.

Table S2. Detected species by MiFish metabarcoding, taxonomic rank, habitat type, number of MiSeq reads, number of unique resuences, information of the species (F, fishery target; O, observed in underwater visual censuses; L, observed around Maizuru Bay according to the literatures), results of automatic taxonomic assignment, and reason for modication of taxonomic assignment.

OTU ID	Assigned taxon*	Taxonomic infromation			Habitat type	Total Read**	Unique seq.**	Prior infromarion	LOW-conf. seq.**	Automated taxomic assignment	Reason for modification
		Group	Order	Family							
1	*(Sphyrna zygaena)*	Shark	Carcharhiniformes	Carcharhinidae	seawater	11	1	F	0%	*Sphyrna zygaena*	
2	*Dipturus kwangtungensis*	Ray	Rajiformes	Rajidae	seawater	50	4	F	0%	*Dipturus kwangtungensis*	
3	*(Dasyatis akajei)*	Ray	Myliobatiformes	Dasyatidae	seawater	24	4	F,O,L	0%	*Dasyatis akajei*	
4	*Muraenesox cinereus*	Bony fish	Anguilliformes	Muraenesocidae	seawater	1364	17	F	0%	*Muraenesox cinereus*	
5	*Engraulis japonicus*	Bony fish	Clupeiformes	Engraulidae	seawater	1146528	6454	F,O,L	0%	*Engraulis japonicus*	
6	*Etrumeus teres*	Bony fish	Clupeiformes	Clupeidae	seawater	3553	24	F,O,L	0%	*Etrumeus teres*	
7	*Konosirus punctatus*	Bony fish	Clupeiformes	Clupeidae	amphidromous	74972	511	F,O,L	0%	*Konosirus punctatus*	
8	*(Sardinella zunasi)*	Bony fish	Clupeiformes	Clupeidae	seawater	19	1	F,L	0%	*Sardinella zunasi*	
9	*Sardinops melanostictus*	Bony fish	Clupeiformes	Clupeidae	seawater	6815	40	F,O,L	100%	*Sardinops sagax*	2
10	*Carassius auratus*	Bony fish	Cypriniformes	Cyprinidae	freshwater	2881	13	F	100%	*Carassius auratus* subsp. KINBUNA	3
11	(*Carassius* sp.)	Bony fish	Cypriniformes	Cyprinidae	freshwater	8	1		100%	*Carassius cuvieri*	1
12	*(Cobitis biwae)*	Bony fish	Cypriniformes	Cobitidae	freshwater	170	3		0%	*Cobitis* sp. BIWAE typeB NorthernKyoto	
13	*Cyprinus carpio*	Bony fish	Cypriniformes	Cyprinidae	freshwater	5979	52	F	100%	*Cyprinus carpio* Lake Kasumigaura	
	(*Cyprinus carpio* [local population 1])	Bony fish	Cypriniformes	Cyprinidae	freshwater	976	17	F	0%	*Cyprinus carpio* Hyogo	
	(*Cyprinus carpio* [local population 2])	Bony fish	Cypriniformes	Cyprinidae	freshwater	12	2	F	100%	*Cyprinus carpio* Lake Biwa	
14	*Gnathopogon elongatus elongatus*	Bony fish	Cypriniformes	Cyprinidae	freshwater	3102	25		0%	*Gnathopogon elongatus elongatus*	
15	(*Hemibarbus* sp.)	Bony fish	Cypriniformes	Cyprinidae	freshwater	8	2		100%	*Hemibarbus labeo*	1
16	*Hemibarbus longirostris*	Bony fish	Cypriniformes	Cyprinidae	freshwater	394	1	O,L	0%	*Hemibarbus longirostris*	

17	*Misgurnus anguillicaudatus*	Bony fish	Cypriniformes	Cobitidae	freshwater	1006	5	F	0%	*Misgurnus anguillicaudatus*	
18	*Nipponocypris temminckii*	Bony fish	Cypriniformes	Cyprinidae	freshwater	3677	28	O,L	0%	*Nipponocypris temminckii*	
19	*Pseudogobio esocinus*	Bony fish	Cypriniformes	Cyprinidae	freshwater	1104	6	O,L	0%	*Pseudogobio esocinus*	
20	*Pungtungia herzi*	Bony fish	Cypriniformes	Cyprinidae	freshwater	589	8	O,L	0%	*Pungtungia herzi*	
21	*Tribolodon hakonensis*	Bony fish	Cypriniformes	Cyprinidae	amphidromous	13399	49	O,L	0%	*Tribolodon hakonensis*	
22	*Zacco platypus*	Bony fish	Cypriniformes	Cyprinidae	freshwater	3424	20	O,L	0%	*Zacco platypus*	
23	*Liobagrus reini*	Bony fish	Siluriformes	Amblycipitidae	freshwater	676	3		0%	*Liobagrus reini*	
24	*Plotosus japonicus*	Bony fish	Siluriformes	Plotosidae	seawater	758	11	F,O,L	0%	*Plotosus japonicus*	
25	(*Silurus asotus*)	Bony fish	Siluriformes	Siluridae	freshwater	18	1	F,O,L	0%	*Silurus asotus*	
26	*Plecoglossus altivelis altivelis*	Bony fish	Osmeriformes	Oseridae	amphidromous	576	13	O,L	100%	*Plecoglossus altivelis altivelis*	
27	(*Hucho perryi*)	Bony fish	Salmoniformes	Salmonidae	freshwater	4	1	F	100%	*Hucho perryi*	
28	*(Oncorhynchus keta)*	Bony fish	Salmoniformes	Salmonidae	amphidromous	6990	20		0%	*Oncorhynchus keta*	
29	*Oncorhynchus nerka*	Bony fish	Salmoniformes	Salmonidae	amphidromous	8725	43		16%	*Oncorhynchus nerka*	4
30	Saurida sp.	Bony fish	Aulopiformes	Synodontidae	seawater	1799	16	O,L	100%	*Saurida elongata*	1
31	*Saurida wanieso*	Bony fish	Aulopiformes	Synodontidae	seawater	1424	9		0%	*Saurida wanieso*	
32	*Gadus chalcogrammus*	Bony fish	Gadiformes	Gadidae	seawater	1964	18	F	100%	*Gadus chalcogrammus*	
33	*Lophius litulon*	Bony fish	Lophiiformes	Lophiidae	seawater	2704	27	F	59%	*Lophius litulon*	Problematic data****
34	*Chelon affinis*	Bony fish	Mugiliformes	Mugilidae	amphidromous	193	2	F	100%	*Chelon affinis*	
35	*Chelon haematocheilus*	Bony fish	Mugiliformes	Mugilidae	amphidromous	4641	32	F	100%	*Chelon haematocheilus*	
36	*Mugil cephalus*	Bony fish	Mugiliformes	Mugilidae	amphidromous	18596	158	F,O,L	0%	*Mugil cephalus*	
37	*Cypselurus* sp.1	Bony fish	Beloniformes	Exocoetidae	seawater	44525	310	F	70%	*Cypselurus heterurus doederleini*	1
38	*Cypselurus* sp.2	Bony fish	Beloniformes	Exocoetidae	seawater	98008	614	F	100%	*Cypselurus hiraii*	1
39	*Cypselurus poecilopterus*	Bony fish	Beloniformes	Exocoetidae	seawater	457	3	F	0%	*Cypselurus poecilopterus*	
40	*Hyporhamphus sajori*	Bony fish	Beloniformes	Hemiramphidae	seawater	11259	49	F,L	0%	*Hyporhamphus sajori*	
41	*Strongylura anastomella*	Bony fish	Beloniformes	Belonidae	seawater	6950	41	L	0%	*Strongylura anastomella*	
42	*Artediellus fuscimentus*	Bony fish	Scorpaeniformes	Cottidae	seawater	823	2		0%	*Artediellus neyelovi*	3

43	*Chelidonichthys spinosus*	Bony fish	Scorpaeniformes	Triglidae	seawater	4267	24	F,L	0%	*Chelidonichthys spinosus*	
44	*Cottiusculus nihonkaiensis*	Bony fish	Scorpaeniformes	Cottidae	seawater	231	3		0%	*Cottiusculus nihonkaiensis*	
45	*(Cottus kazika)*	Bony fish	Scorpaeniformes	Cottidae	amphidromous	6	1	F,O,L	0%	*Cottus kazika*	
46	*Hexagrammos* sp.	Bony fish	Scorpaeniformes	Hexagrammidae	seawater	417	4		100%	*Hexagrammos lagocephalus*	1
47	*Hexagrammos otakii*	Bony fish	Scorpaeniformes	Hexagrammidae	seawater	859	7	F,O,L	0%	*Hexagrammos otakii*	
48	*Lepidotrigla microptera*	Bony fish	Scorpaeniformes	Triglidae	seawater	4375	32	F	0%	*Lepidotrigla microptera*	
49	*Liparis tessellatus*	Bony fish	Scorpaeniformes	Liparidae	seawater	588	3		0%	*Liparis tessellatus*	
50	*Paracentropogon rubripinnis*	Bony fish	Scorpaeniformes	Scorpaenidae	seawater	20544	91	O,L	0%	*Paracentropogon rubripinnis*	
51	*Platycephalus* sp. (MAGOCHI)	Bony fish	Scorpaeniformes	Platycephalidae	seawater	4738	25	F,O,L	100%	*Platycephalus* sp. MAGOCHI	2
52	*Sebastes* sp.	Bony fish	Scorpaeniformes	Scorpaenidae	seawater	5476	42		100%	*Sebastes aleutianus*	2
53	*(Sebastes crameri)*	Bony fish	Scorpaeniformes	Scorpaenidae	seawater	4	1		0%	*Sebastes crameri*	
54	*(Sebastes koreanus)*	Bony fish	Scorpaeniformes	Scorpaenidae	seawater	4	1		0%	*Sebastes koreanus*	
55	*Sebastiscus marmoratus*	Bony fish	Scorpaeniformes	Scorpaenidae	seawater	5364	42	F,O,L	2%	*Sebastiscus marmoratus*	4
56	*Sebastiscus tertius*	Bony fish	Scorpaeniformes	Scorpaenidae	seawater	624	2	F,O,L	0%	*Sebastiscus tertius*	
57	*Acanthocepola krusensternii*	Bony fish	Perciformes	Cepolidae	seawater	801	6		0%	*Acanthocepola krusensternii*	
58	*Acanthogobius flavimanus*	Bony fish	Perciformes	Gobiidae	brackish	16653	105	F,L	0%	*Acanthogobius flavimanus*	
59	*Acanthopagrus schlegelii*	Bony fish	Perciformes	Sparidae	amphidromous	153578	1033	F,O,L	100%	*Acanthopagrus sivicolus*	2
60	*Acentrogobius pflaumii*	Bony fish	Perciformes	Gobiidae	seawater	4479	21	O,L	0%	*Acentrogobius pflaumii*	
61	*Acentrogobius virgatulus* species complex	Bony fish	Perciformes	Gobiidae	seawater	775	4		100%	*Acentrogobius virgatulus*	2
62	Gobiid species	Bony fish	Perciformes	Gobiidae	seawater	34105	217		100%	*Amblychaeturichthys hexanema*	1
63	*Amblychaeturichthys sciistius*	Bony fish	Perciformes	Gobiidae	seawater	7345	33		0%	*Amblychaeturichthys sciistius*	
64	*Ammodytes japonicus*	Bony fish	Perciformes	Ammodytidae	seawater	487	2	F,O,L	100%	*Ammodytes personatus*	3
65	*(Arctoscopus japonicus)*	Bony fish	Perciformes	Trichodontidae	seawater	142	1	F	0%	*Arctoscopus japonicus*	
66	*Branchiostegus japonicus*	Bony fish	Perciformes	Malacanthidae	seawater	635	5	F,O,L	0%	*Branchiostegus japonicus*	
67	*Chaenogobius gulosus*	Bony fish	Perciformes	Gobiidae	seawater	1860	11	O,L	0%	*Chaenogobius gulosus*	
68	*Davidijordania poecilimon*	Bony fish	Perciformes	Zoarcidae	seawater	2526	8		0%	*Davidijordania poecilimon*	

69	*Decapterus maruadsi*	Bony fish	Perciformes	Carangidae	seawater	1984	14	F	0%	*Decapterus maruadsi*	
70	*Dentex hypselosomus*	Bony fish	Perciformes	Sparidae	seawater	9530	67	F	100%	*Dentex hypselosomus*	2
71	*Dictyosoma burgeri*	Bony fish	Perciformes	Stichaeidae	seawater	10822	70	F,O,L	0%	*Dictyosoma burgeri*	
72	*Ditrema temminckii temminckii*	Bony fish	Perciformes	Embiotocidae	seawater	16552	93	F,O,L	100%	*Ditrema temminckii pacificum*	2
73	*Epinephelus akaara*	Bony fish	Perciformes	Serranidae	seawater	1276	8	F,O,L	0%	*Epinephelus akaara*	
74	*Epinephelus awoara*	Bony fish	Perciformes	Serranidae	seawater	1044	5	F,O,L	0%	*Epinephelus awoara*	
75	*(Equulites rivulatus)*	Bony fish	Perciformes	Leiognathidae	seawater	204	1	F,O,L	0%	*Equulites rivulatus*	
76	*Ernogrammus hexagrammus*	Bony fish	Perciformes	Stichaeidae	seawater	2270	17	O,L	0%	*Ernogrammus hexagrammus*	
77	*Eviota abax*	Bony fish	Perciformes	Gobiidae	seawater	664	9	O,L	0%	*Eviota abax*	
78	*Girella punctata*	Bony fish	Perciformes	Kyphosidae	seawater	13683	98	F,L	0%	*Girella punctata*	
79	*Glossogobius olivaceus*	Bony fish	Perciformes	Gobiidae	amphidromous	598	3		0%	*Glossogobius olivaceus*	
80	*Gymnogobius breunigii*	Bony fish	Perciformes	Gobiidae	amphidromous	478	2		0%	*Gymnogobius breunigii*	
81	*Halichoeres tenuispinis*	Bony fish	Perciformes	Labridae	seawater	3952	33	O,L	0%	*Halichoeres tenuispinis*	
82	*Helichoeres poecilopterus*	Bony fish	Perciformes	Labridae	seawater	5324	40	F,O,L	0%	*Helichoeres poecilopterus*	
83	*Hyperoglyphe japonica*	Bony fish	Perciformes	Centrolophidae	seawater	1686	14	F	0%	*Hyperoglyphe japonica*	
84	*Istigobius campbelli*	Bony fish	Perciformes	Gobiidae	seawater	339	3	O,L	0%	*Istigobius campbelli*	
85	*Jaydia lineata*	Bony fish	Perciformes	Apogonidae	seawater	1470	11		0%	*Jaydia lineata*	
86	*(Kaiwarinus equula)*	Bony fish	Perciformes	Carangidae	seawater	186	1	F	0%	*Kaiwarinus equula*	
87	*Lateolabrax japonicus*	Bony fish	Perciformes	Moronidae	amphidromous	55231	347	F,O,L	0%	*Lateolabrax japonicus*	4
88	*Leucopsarion petersii*	Bony fish	Perciformes	Gobiidae	amphidromous	3085	23	F,O,L	0%	*Leucopsarion petersii*	
89	*(Luciogobius guttatus)*	Bony fish	Perciformes	Gobiidae	seawater	35	1	O,L	100%	*Luciogobius platycephalus*	2
90	*Mugilogobius abei*	Bony fish	Perciformes	Gobiidae	brackish	1100	6		0%	*Mugilogobius abei*	
91	*Myersina filifer*	Bony fish	Perciformes	Gobiidae	seawater	1094	9	O,L	0%	*Myersina filifer*	
92	*Nuchequula nuchalis*	Bony fish	Perciformes	Leiognathidae	seawater	104695	703	F,O,L	0%	*Nuchequula nuchalis*	
93	*Odontobutis obscura*	Bony fish	Perciformes	Odontobutidae	freshwater	957	4	O,L	0%	*Odontobutis obscura*	
94	*Omobranchus elegans*	Bony fish	Perciformes	Blenniidae	seawater	1718	9	O,L	0%	*Omobranchus elegans*	

95	*Omobranchus fasciolatoceps*	Bony fish	Perciformes	Blenniidae	seawater	1392	8		0%	*Omobranchus fasciolatoceps*	
96	*Omobranchus punctatus*	Bony fish	Perciformes	Blenniidae	seawater	2083	13		0%	*Omobranchus punctatus*	
97	*Oplegnathus fasciatus*	Bony fish	Perciformes	Oplegnathidae	seawater	1610	11	F,O,L	0%	*Oplegnathus fasciatus*	
98	*Pagrus major*	Bony fish	Perciformes	Sparidae	seawater	8476	52	F,O,L	100%	*Pagrus major*	
99	*Parablennius yatabei*	Bony fish	Perciformes	Blenniidae	seawater	5069	30	O,L	0%	*Parablennius yatabei*	
100	*Parapristipoma trilineatum*	Bony fish	Perciformes	Haemulidae	seawater	4858	32	F,O,L	0%	*Parapristipoma trilineatum*	
101	*Paratrypauchen microcephalus*	Bony fish	Perciformes	Gobiidae	seawater	29	1	F,O,L	0%	*Paratrypauchen microcephalus*	
102	*Pennahia argentata*	Bony fish	Perciformes	Sciaenidae	seawater	11771	85	F	0%	*Pennahia argentata*	
103	*Pholis fangi*	Bony fish	Perciformes	Pholidae	seawater	3691	35		0%	*Pholis fangi*	
104	*Pholis nebulosa*	Bony fish	Perciformes	Pholidae	seawater	945	7	F,O,L	0%	*Pholis nebulosa*	
105	*Plectorhinchus cinctus*	Bony fish	Perciformes	Haemulidae	seawater	681	3	F	0%	*Plectorhinchus cinctus*	
106	*Pseudolabrus sieboldi*	Bony fish	Perciformes	Labridae	seawater	1939	12	F,O,L	0%	*Pseudolabrus sieboldi*	
107	Callionymid species	Bony fish	Perciformes	Callionymidae	seawater	1364	6	F,O,L	100%	*Repomucenus curvicornis*	1
108	*Repomucenus valenciennei*	Bony fish	Perciformes	Callionymidae	seawater	3366	26	F,O,L	0%	*Repomucenus valenciennei*	
109	*Rhinogobius flumineus*	Bony fish	Perciformes	Gobiidae	freshwater	34	2	O,L	0%	*Rhinogobius flumineus*	
110	*Rhinogobius giurinus*	Bony fish	Perciformes	Gobiidae	amphidromous	3153	25		0%	*Rhinogobius giurinus*	
111	*Rhynchopelates oxyrhynchus*	Bony fish	Perciformes	Terapontidae	seawater	4574	33	F,O,L	0%	*Rhynchopelates oxyrhynchus*	
112	*Sarda orientalis*	Bony fish	Perciformes	Scombridae	seawater	11825	64	F	0%	*Sarda orientalis*	
113	*Scomber* sp.	Bony fish	Perciformes	Scombridae	seawater	5394	40	F	100%	*Scomber japonicus*	1
114	*Scomber scombrus*	Bony fish	Perciformes	Scombridae	seawater	1235	13	F	0%	*Scomber scombrus*	
115	*Scomberomorus niphonius*	Bony fish	Perciformes	Scombridae	seawater	69754	455	F,O,L	0%	*Scomberomorus niphonius*	
116	*(Seriola dumerili)*	Bony fish	Perciformes	Carangidae	seawater	140	1	F,O,L	0%	*Seriola dumerili*	
117	*Seriola lalandi*	Bony fish	Perciformes	Carangidae	seawater	246	1	F	0%	*Seriola lalandi*	
118	*Seriola quinqueradiata*	Bony fish	Perciformes	Carangidae	seawater	33749	582	F,L	0%	*Seriola quinqueradiata*	
119	*Sillago japonica*	Bony fish	Perciformes	Sillaginidae	seawater	8351	57	F,O,L	0%	*Sillago japonica*	
120	*Sphyraena pinguis*	Bony fish	Perciformes	Sphyraenidae	seawater	20810	143	F,O,L	0%	*Sphyraena pinguis*	

121	*Thunnus* sp.	Bony fish	Perciformes	Scombridae	seawater	637	9	F	100%	*Thunnus alalunga*	1
122	*Thunnus orientalis*	Bony fish	Perciformes	Scombridae	seawater	975	7	F	0%	*Thunnus orientalis*	
123	*Trachurus japonicus*	Bony fish	Perciformes	Carangidae	seawater	155611	1014	F,O,L	3%	*Trachurus japonicus*	4
124	*Trachurus* sp.	Bony fish	Perciformes	Carangidae	seawater	875	4		50%	*Trachurus symmetricus*	1
125	*Trachurus trachurus*	Bony fish	Perciformes	Carangidae	seawater	217	3	F	0%	*Trachurus trachurus*	
126	*Trichiurus japonicus*	Bony fish	Perciformes	Trichiuridae	seawater	201	6	F,O,L	0%	*Trichiurus japonicus*	
127	*Tridentiger* sp.1	Bony fish	Perciformes	Gobiidae	brackish	562	5		100%	*Tridentiger bifasciatus*	1
128	*Tridentiger* sp.2	Bony fish	Perciformes	Gobiidae	freshwater	8258	62	O,L	100%	*Tridentiger obscurus*	1
129	*Tridentiger trigonocephalus*	Bony fish	Perciformes	Gobiidae	seawater	22498	173	O,L	0%	*Tridentiger trigonocephalus*	
130	*(Trimma grammistes)*	Bony fish	Perciformes	Gobiidae	seawater	6	1	O,L	0%	*Trimma grammistes*	
131	*Upeneus japonicus*	Bony fish	Perciformes	Mullidae	seawater	445	3	F,O,L	100%	*Upeneus japonicus*	
132	*Zoarchias major*	Bony fish	Perciformes	Zoarcidae	seawater	1864	7	O,L	0%	*Zoarchias veneficus*	3
133	*Glyptocephalus stelleri*	Bony fish	Pleuronectiformes	Pleuronectidae	seawater	2094	16	F	100%	*Glyptocephalus zachirus*	2
134	*Hippoglossoides* sp.	Bony fish	Pleuronectiformes	Pleuronectidae	seawater	14663	99		100%	*Hippoglossoides platessoides*	1
135	*Paralichthys olivaceus*	Bony fish	Pleuronectiformes	Paralichthyidae	seawater	3634	29	F,O,L	0%	*Paralichthys olivaceus*	
136	*Pleuronectes* sp.	Bony fish	Pleuronectiformes	Pleuronectidae	seawater	1604	13	F,L	100%	*Pleuronectes yokohamae*	1
137	*Pleuronichthys cornutus*	Bony fish	Pleuronectiformes	Pleuronectidae	seawater	751	1	F,O,L	0%	*Pleuronichthys cornutus*	
138	*Pseudaesopia japonica*	Bony fish	Pleuronectiformes	Soleidae	seawater	85	3	F,O,L	0%	*Pseudaesopia japonica*	
139	*Pseudorhombus* sp.	Bony fish	Pleuronectiformes	Paralichthyidae	seawater	173	1	F	100%	*Pseudorhombus oligodon*	1
140	*Tanakius kitaharae*	Bony fish	Pleuronectiformes	Pleuronectidae	seawater	665	4	F	0%	*Tanakius kitaharae*	
141	*Rudarius ercodes*	Bony fish	Tetraodontiformes	Monacanthidae	seawater	48	1	O,L	0%	*Rudarius ercodes*	
142	*Stephanolepis cirrhifer*	Bony fish	Tetraodontiformes	Monacanthidae	seawater	248	8	F,O,L	0%	*Stephanolepis cirrhifer*	
143	*Takifugu* sp.1	Bony fish	Tetraodontiformes	Tetraodontidae	seawater	81773	341		80%	*Takifugu niphobles*	1
144	*Takifugu* sp.2	Bony fish	Tetraodontiformes	Tetraodontidae	seawater	55669	411		100%	*Takifugu pardalis*	1
145	(*Takifugu* sp.3)	Bony fish	Tetraodontiformes	Tetraodontidae	seawater	4	1		100%	*Takifugu porphyreus*	1
146	*Takifugu* sp.4	Bony fish	Tetraodontiformes	Tetraodontidae	seawater	3834	24		96%	*Takifugu snyderi*	1

147	*Thamnaconus modestus*	Bony fish	Tetraodontiformes	Monacanthidae	seawater	1910	13	F,O,L	0%	*Thamnaconus modestus*

* Parentheses indicate that those OTUs were not subjected to the analyses of fish fauna. Those OTUs were not remained in any samples after applied read count cut-off.

** Read count, number of unique sequence and frequency of unique sequences with LOW-confidence assignment before applied read count cut-off.

*** Reason 1, assigned to genus or family level because the sequence cannot be distinguished among closely-related species; reason 2; re-assigned ambiguously assigned OTU (i.e. genus or family level) to specific species identity because only one species from the taxonomic group occurs around Maizuru Bay; reason 3, when species assigned by automated pipeline never occur around Maizuru Bay, we assigned to closely related species that is not included in reference database but is likely to occur in Maizuru Bay; reason 4, original assignment remained in spite of LOW-confident sequence when the frequency of LOW-confident sequence was low.

**** Because second candidate of ID 33 was problematic data, we determined assigned species (i.e. *Lophius litulon*) based on HIGH-confidence assignments. Some LOW-confidence assignment suggested *Grammistes sexlineatus* (accession KJ489014) but this sequence was identical to *Lophius litulon*.

Table S3. Species that have been observed by underwater visual census for 14 years.

Species name	Num. observed individuals	Detected by MiFish metabarcoding	Reference data
Trachurus japonicus	25413	Detected	
Sebastes inermis	8005		impossible to distinguish among closely related species
Plotosus japonicus	7627	Detected	
Tridentiger trigonocephalus	4593	Detected	
Engraulis japonicus	3871	Detected	
Halichoeres tenuispinis	3399	Detected	
Pterogobius zonoleucus	3384		
Chaenogobius gulosus	2466	Detected	
Girella punctata	2450	Detected	
Sphyraena pinguis	1728	Detected	
Acentrogobius pflaumii	1521	Detected	
Helichoeres poecilopterus	1491	Detected	
Pseudolabrus sieboldi	1367	Detected	
Rudarius ercodes	935	Detected	
Pagrus major	738	Detected	
Sardinops melanostictus	700	Detected	
Sebastes thompsoni	544		impossible to distinguish among closely related species
Ditrema temminckii temminckii	508	Detected	
Acanthopagrus schlegelii	499	Detected	
Pseudoblennius cottoides	417		
Takifugu poecilonotus	333		impossible to distinguish among closely related species
Lutjanus ophuysenii	129		
Hexagrammos otakii	126	Detected	impossible to distinguish among closely related species
Takifugu niphobles	126		
Rhynchopelates oxyrhynchus	115	Detected	
Paracentropogon rubripinnis	112	Detected	
Hexagrammos agrammus	89		impossible to distinguish among closely related species
Istigobius hoshinonis	79		
Stephanolepis cirrhifer	79	Detected	
Omobranchus elegans	74	Detected	
Parapristipoma trilineatum	73	Detected	
Mugil cephalus	68	Detected	
Gymnogobius heptacanthus	52		not included in reference database
Epinephelus awoara	47	Detected	
Semicossyphus reticulatus	42		
Istigobius campbelli	42	Detected	

Myersina filifer	42	Detected	
Epinephelus akaara	41	Detected	
Chromis notata notata	41		
Oplegnathus fasciatus	41	Detected	
Suezichthys gracilis	38		not included in reference database
Upeneus japonicus	36	Detected	
Sebastiscus marmoratus	30	Detected	
Petroscirtes breviceps	26		
Dictyosoma burgeri	19	Detected	
Apogon semilineatus	15		
Sillago japonica	14	Detected	
Pleuronichthys cornutus	12	Detected	
Hippocampus coronatus	10		
Sebastes pachycephalus	9		impossible to distinguish among closely related species
Saurida sp. (MAESO)	8		not included in reference database
Lateolabrax japonicus	8	Detected	
Trimma grammistes	7		
Repomucenus curvicornis	7		
Scorpaenodes littoralis	6		not included in reference database
Inegocia japonica	5		not included in reference database
Furcina ishikawae	5		not included in reference database
Sebastes oblongus	5		impossible to distinguish among closely related species
Thamnaconus modestus	5	Detected	
Goniistius quadricornis	4		not included in reference database
Parablennius yatabei	4	Detected	
Paramonacanthus japonicus	4		
Paralichthys olivaceus	4	Detected	
Eviota abax	3	Detected	
Choerodon azurio	2		
Syngnathus schlegeli	2		
Inimicus japonicus	1		
Furcina osimae	1		
Parapercis snyderi	1		
Dasyatis akajei	1		
Platycephalus sp. (MAGOCHI)	1	Detected	
Seriola dumerili	1		
Nuchequula nuchalis	1	Detected	
Chaetodontoplus septentrionalis	1		

Abudefduf vaigiensis	1	
Oplegnathus punctatus	1	
Pterogobius elapoides	1	
Favonigobius gymnauchen	1	
Siganus fuscescens	1	
Takifugu pardalis	1	impossible to distinguish among closely related species

Table S4. Detected species and its number of MiSeq read from negative controls.

PCR round	Negative control	Detected species and number of Miseq reads							
		Acanthopagrus sivicolus	Amblychaeturichthys hexanema	Cyprinus carpio Hyogo	Ditrema temminckii pacificum	Engraulis japonicus	Gadus chalcogramma	Halichoeres tenuispinis	Lateolabrax japonicus
First round	Equipment 1	0	0	0	0	4	315	0	0
Second round	Equipment 1	0	0	0	0	4	0	0	0
	Equipment 2	11	0	0	0	126	0	0	0
	Equipment 3	0	0	0	29	201	0	0	0
	Equipment 4	0	0	0	0	84	0	0	6
	Equipment 7	0	0	0	0	4	0	0	0
	Equipment 8	0	0	4	0	0	0	0	0
	Equipment 9	0	4	0	0	4	0	0	0
	PCR blank	0	0	0	0	624	0	12	0
Third round	Equipment 1	0	0	0	0	6	0	0	0
	Equipment 4	0	0	0	0	0	0	0	0
	Equipment 6	0	0	0	0	4	0	0	0

Table S4. (continued)

PCR round	Negative control	Detected species and number of Miseq reads						
		Nuchequula nuchalis	Oncorhynchus keta	Scomberomorus niphonius	Sebastes aleutianus	Strongylura anastomella	Takifugu niphobles	Takifugu pardalis
First round	Equipment 1	0	0	0	0	0	0	0
Second round	Equipment 1	0	0	0	0	0	0	0
	Equipment 2	45	0	0	4	0	76	8
	Equipment 3	0	0	0	0	11	0	0
	Equipment 4	6	0	0	0	0	0	0
	Equipment 7	0	686	0	0	0	0	0
	Equipment 8	0	0	0	0	0	0	0
	Equipment 9	0	0	0	0	0	0	0
	PCR blank	4	0	30	0	0	0	51
Third round	Equipment 1	0	0	0	0	0	0	0
	Equipment 4	0	1415	0	0	0	0	0
	Equipment 6	0	0	0	0	0	0	0

Fig.S1_Acanthocepola_krusensternii

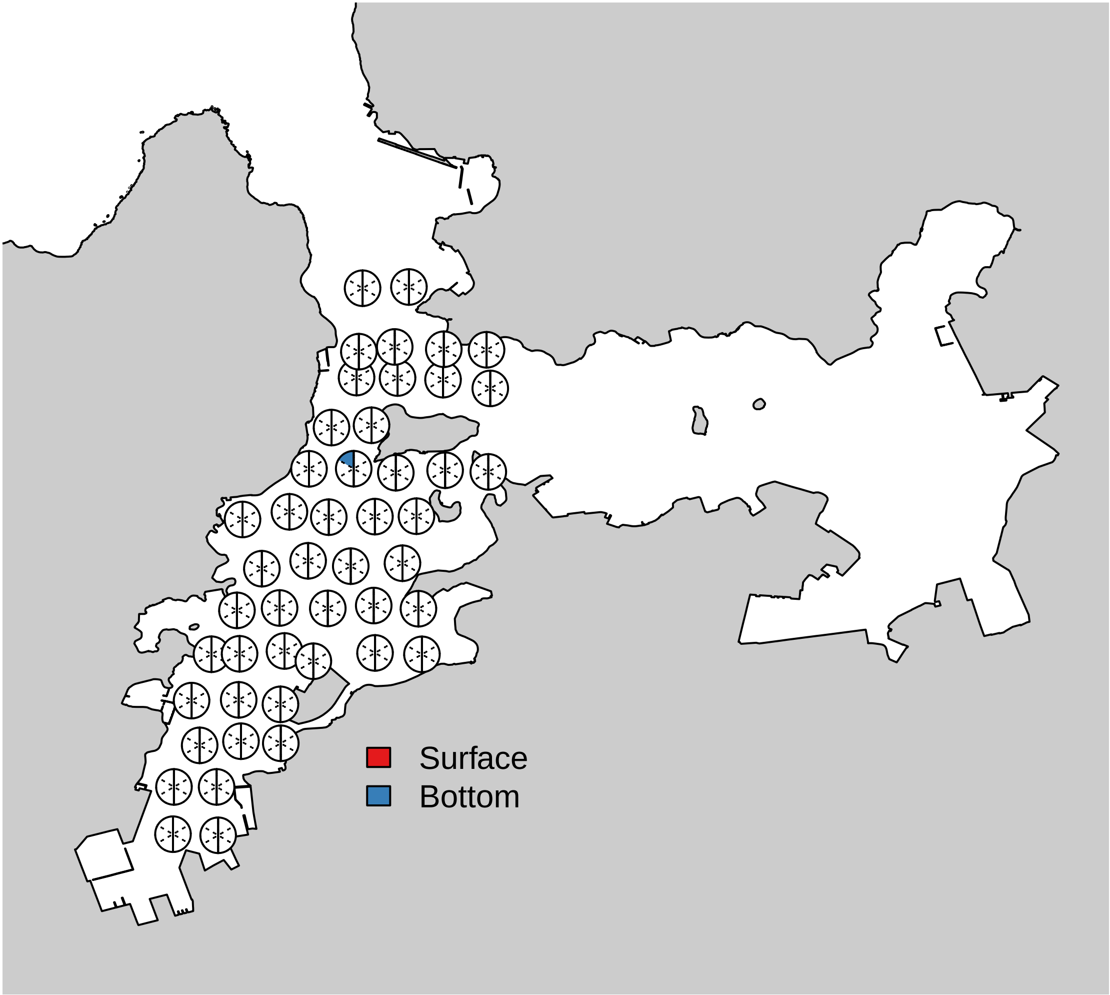

Fig.S1_Acanthogobius_flavimanus

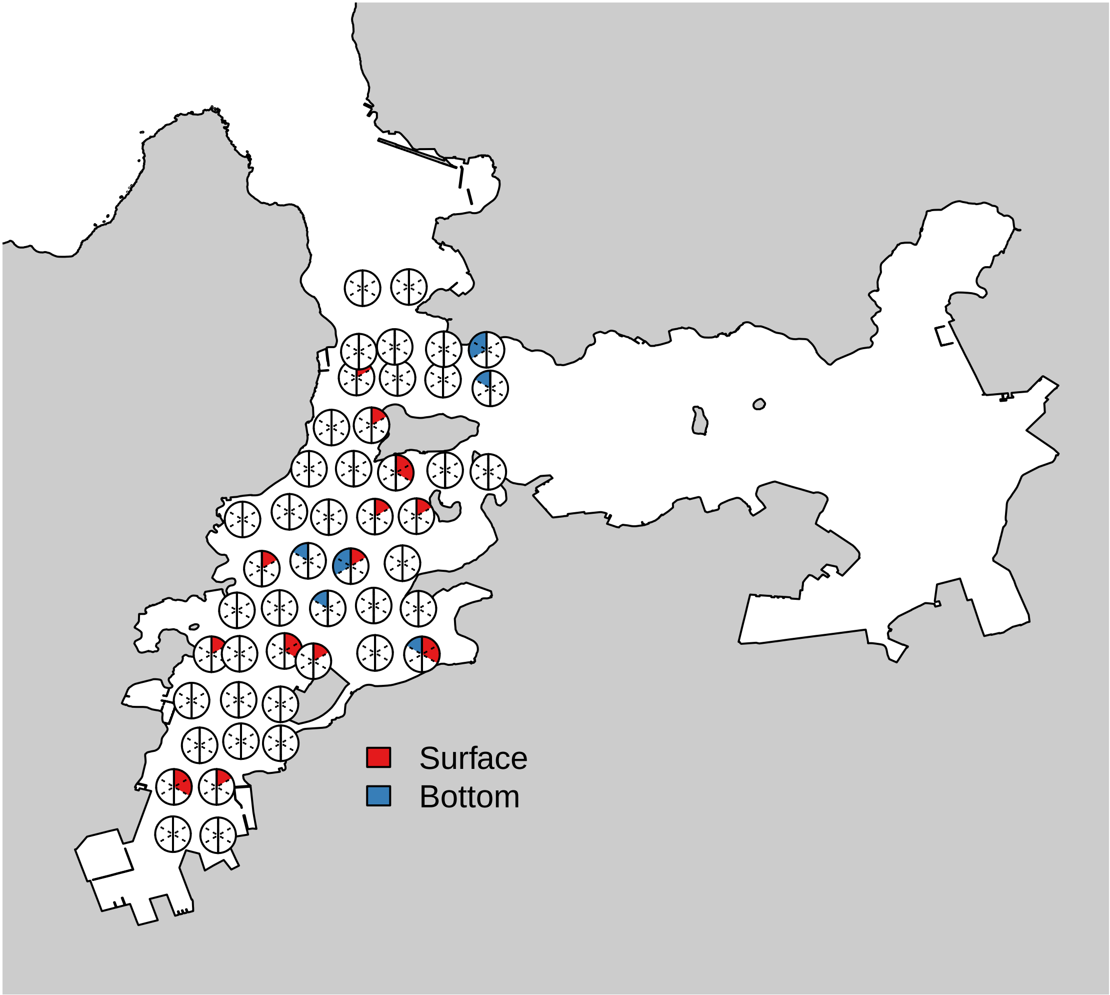

Fig.S1_Acanthopagrus_schlegelii

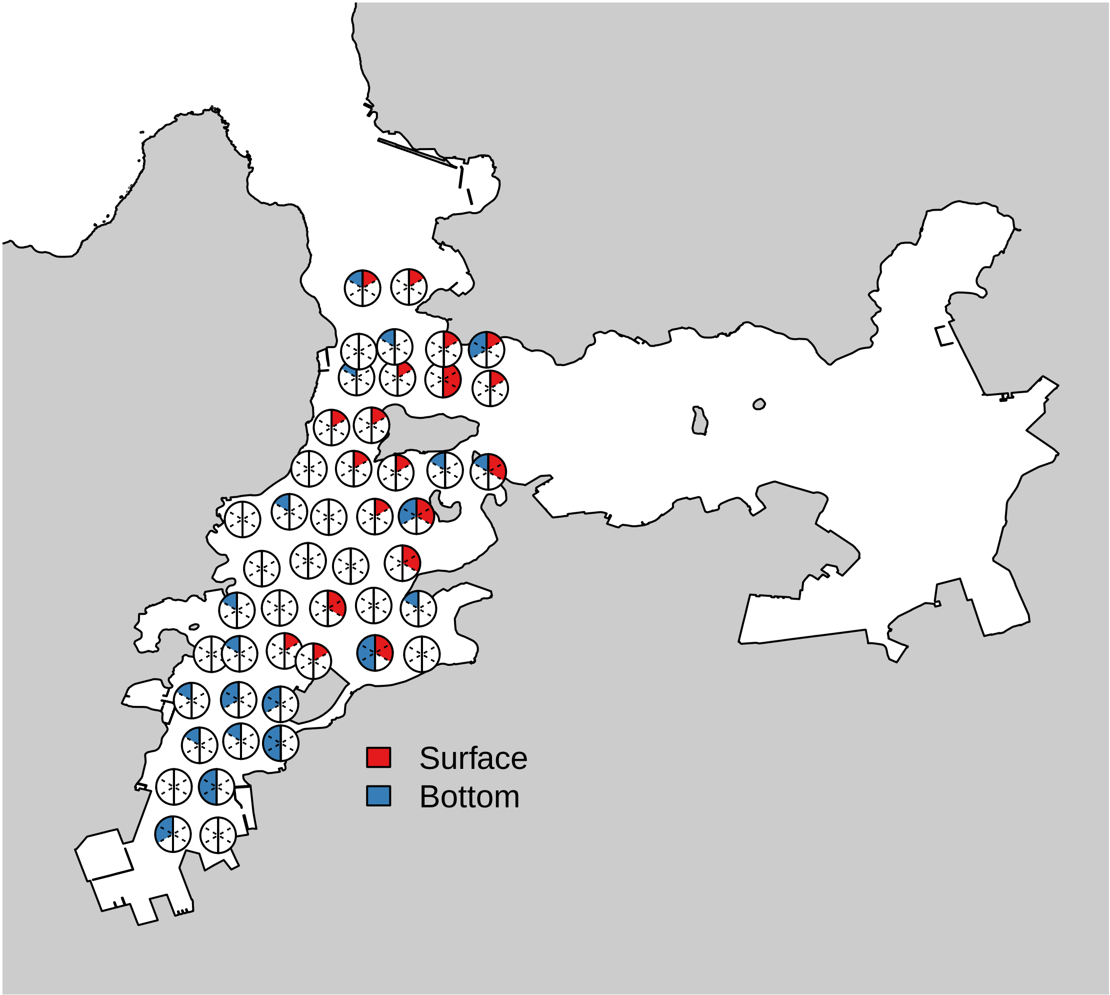

Fig.S1_Acentrogobius_pflaumii

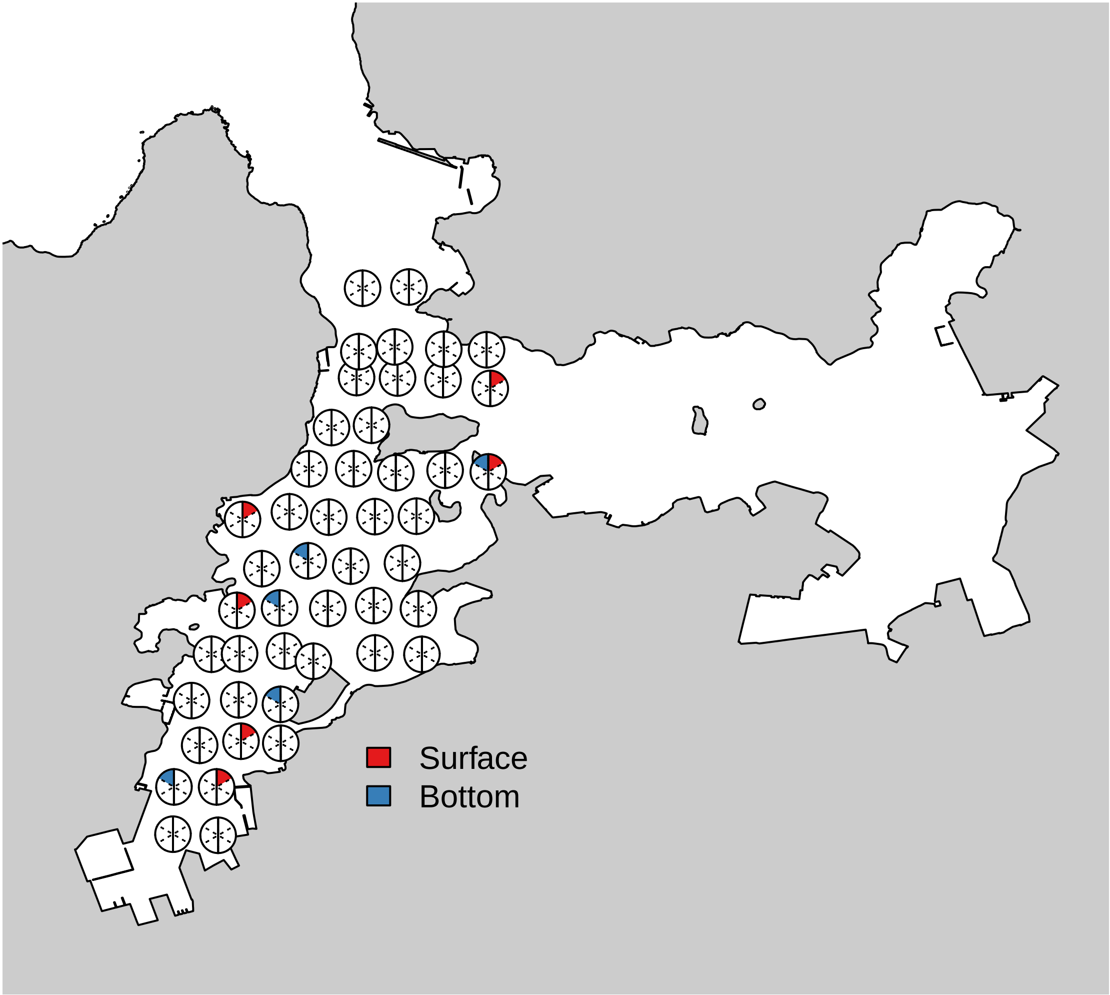

Fig.S1_Acentrogobius_virgatulus_species_complex

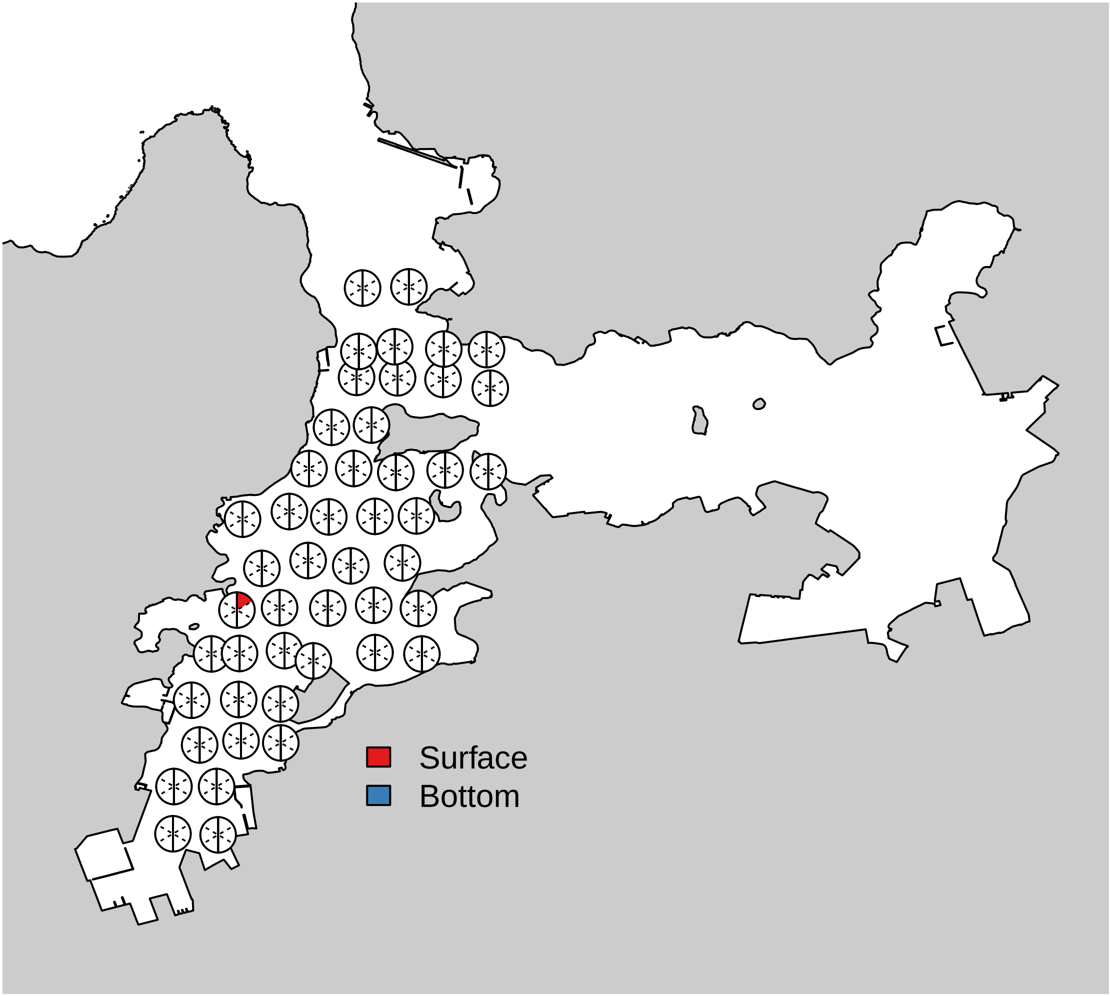

Fig.S1_Amblychaeturichthys_sciistius

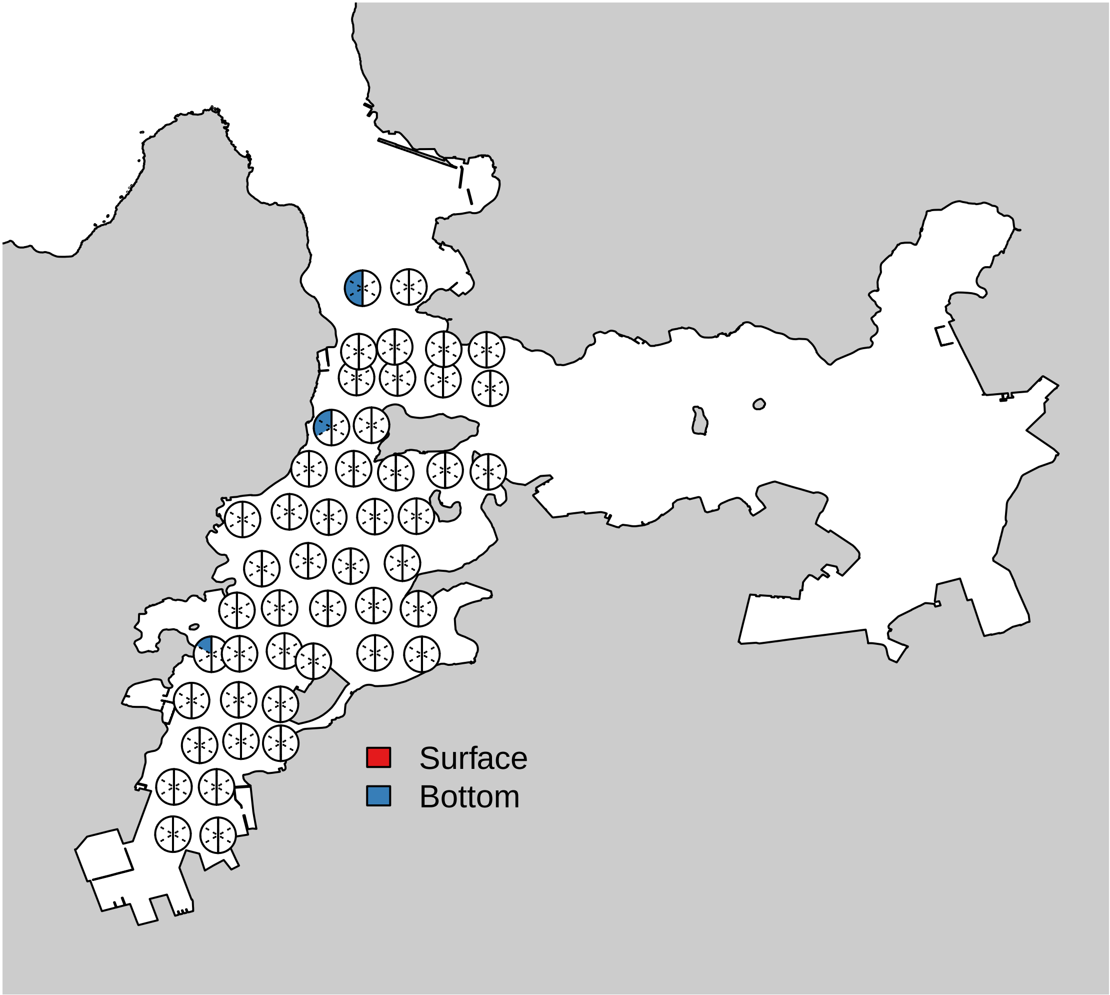

Fig.S1_Ammodytes_japonicus

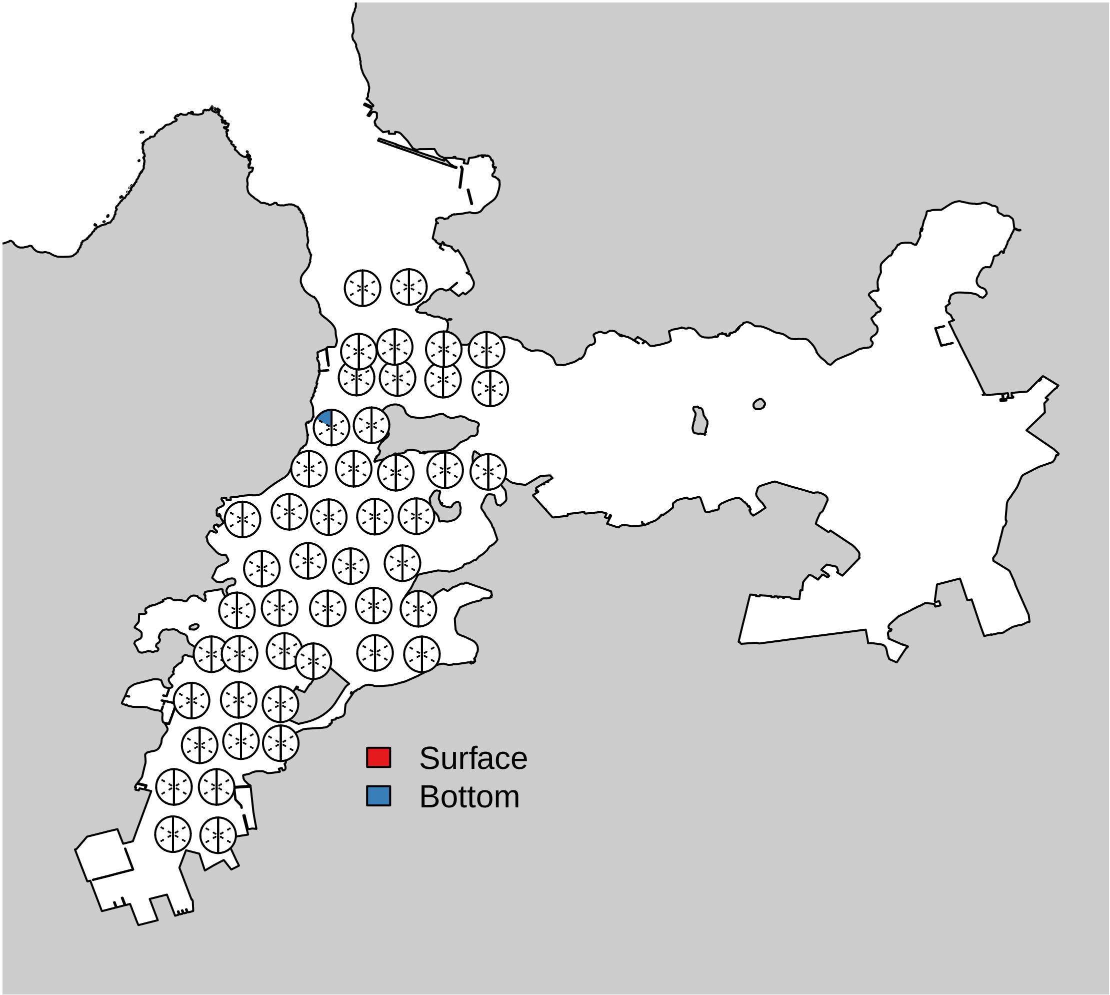

Fig.S1_Artediellus_fuscimentus

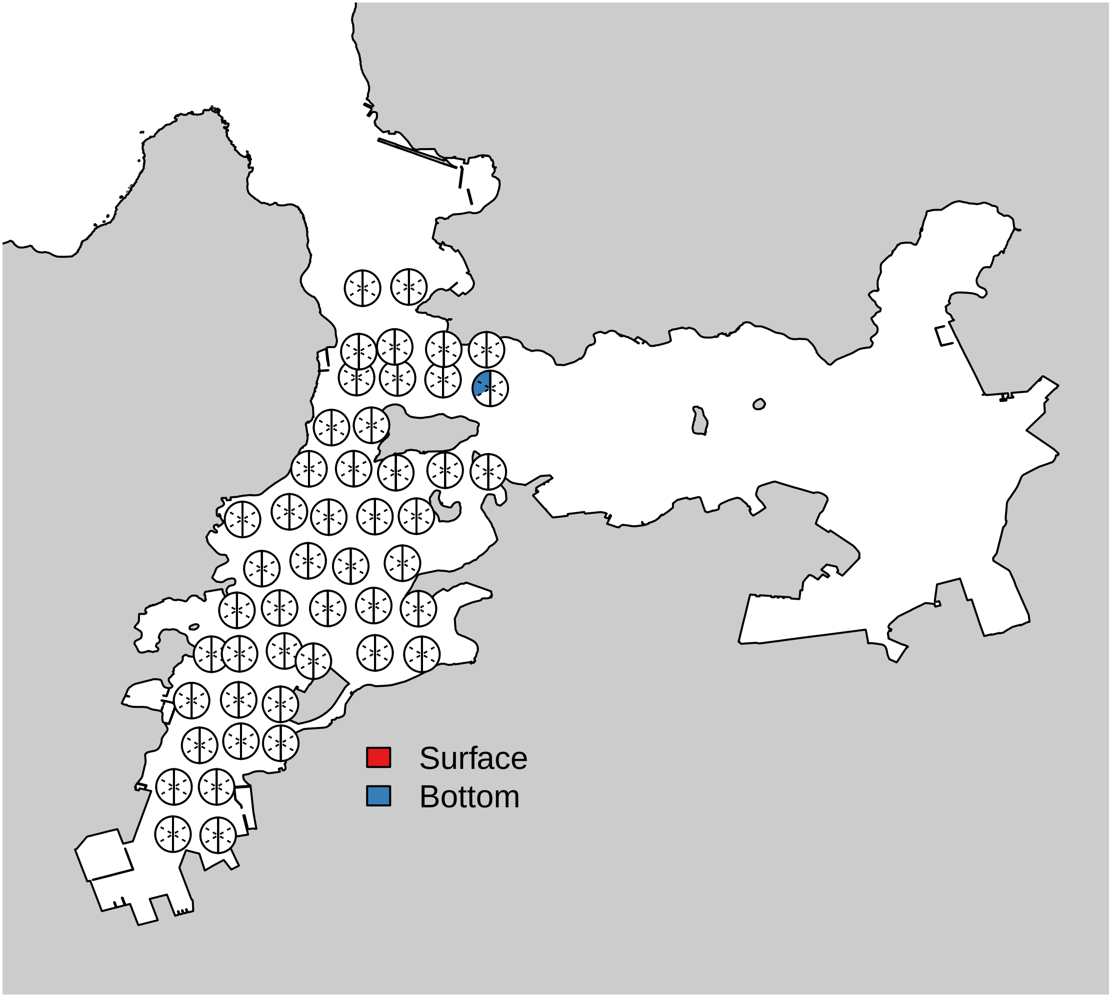

Fig.S1_Branchiostegus_japonicus

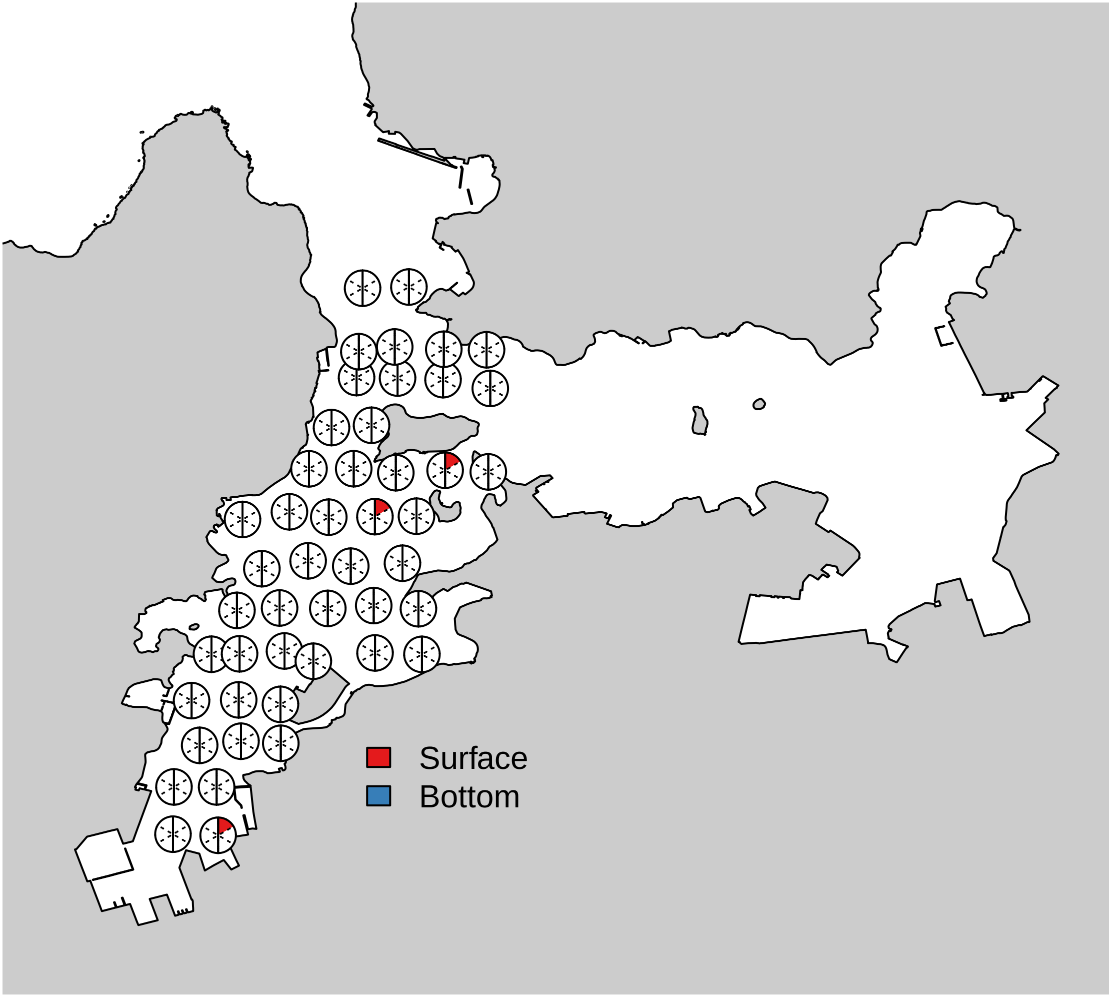

Fig.S1_Callionymid_species

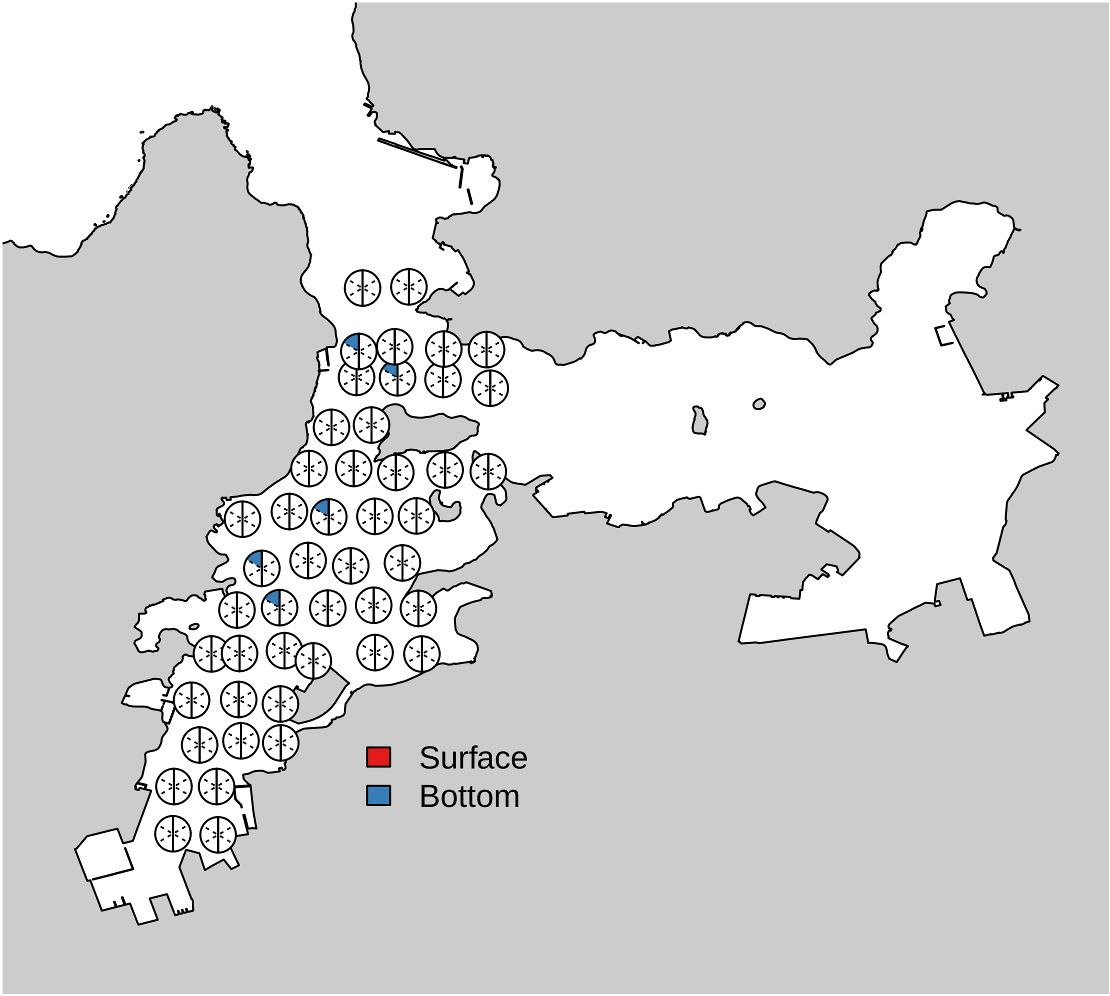

Fig.S1_Carassius_auratus

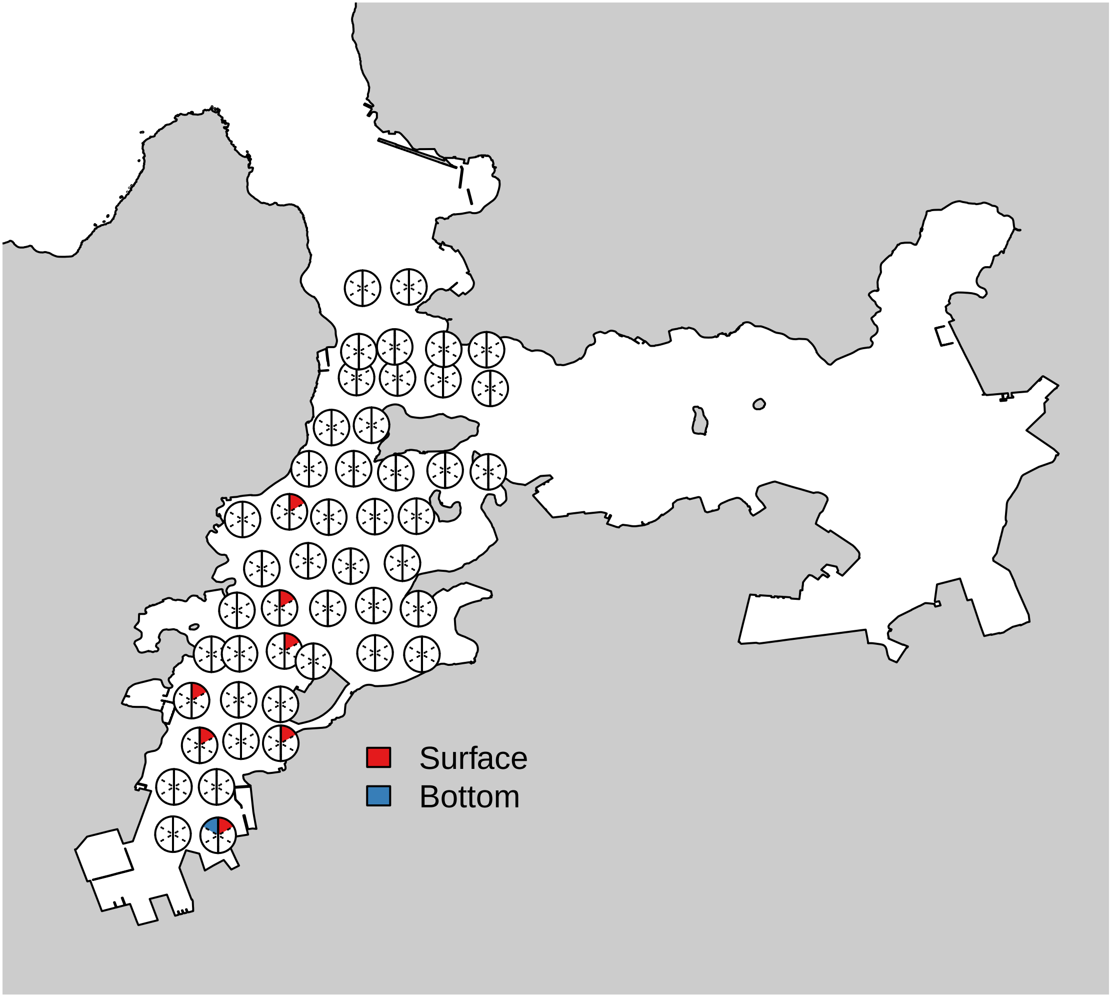

Fig.S1_Chaenogobius_gulosus

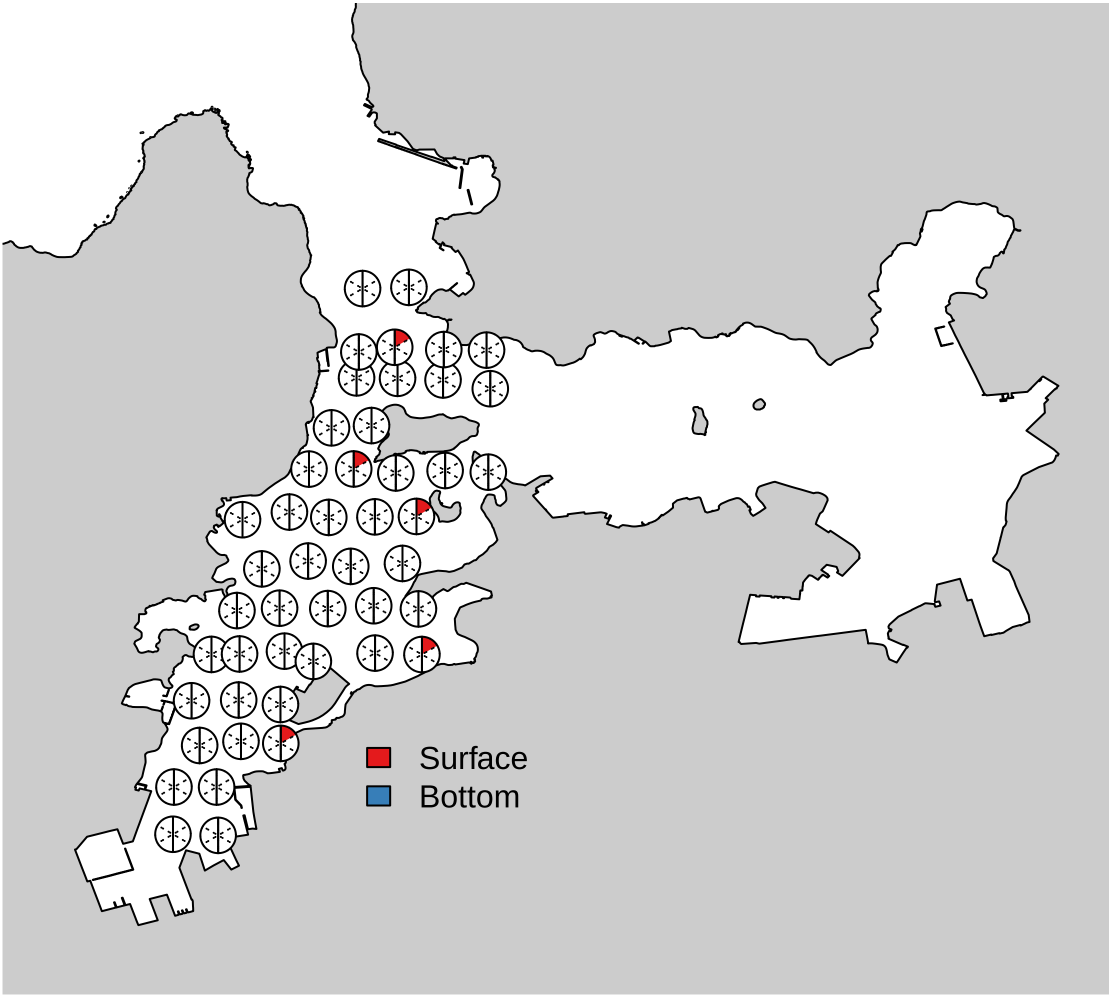

Fig.S1_Chelidonichthys_spinosus

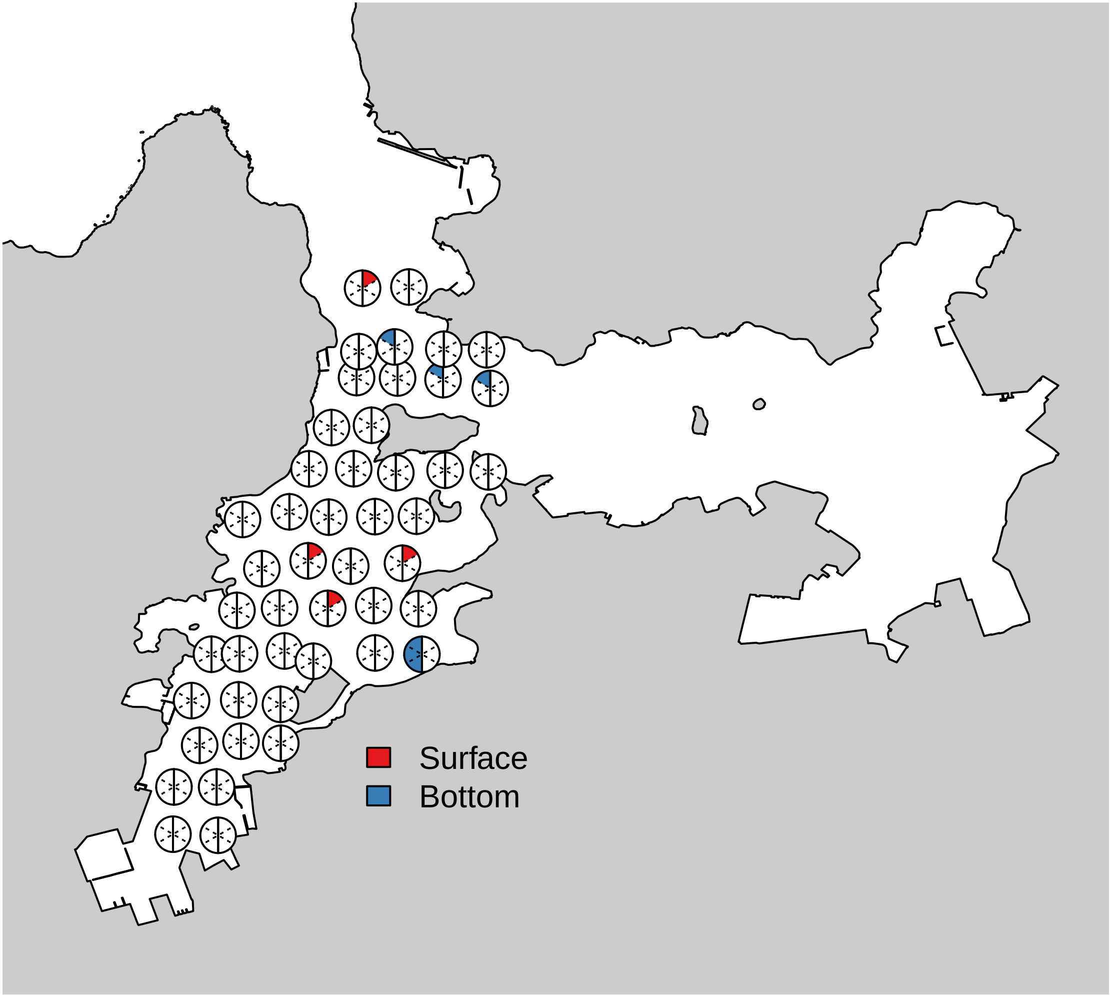

Fig.S1_Chelon_affinis

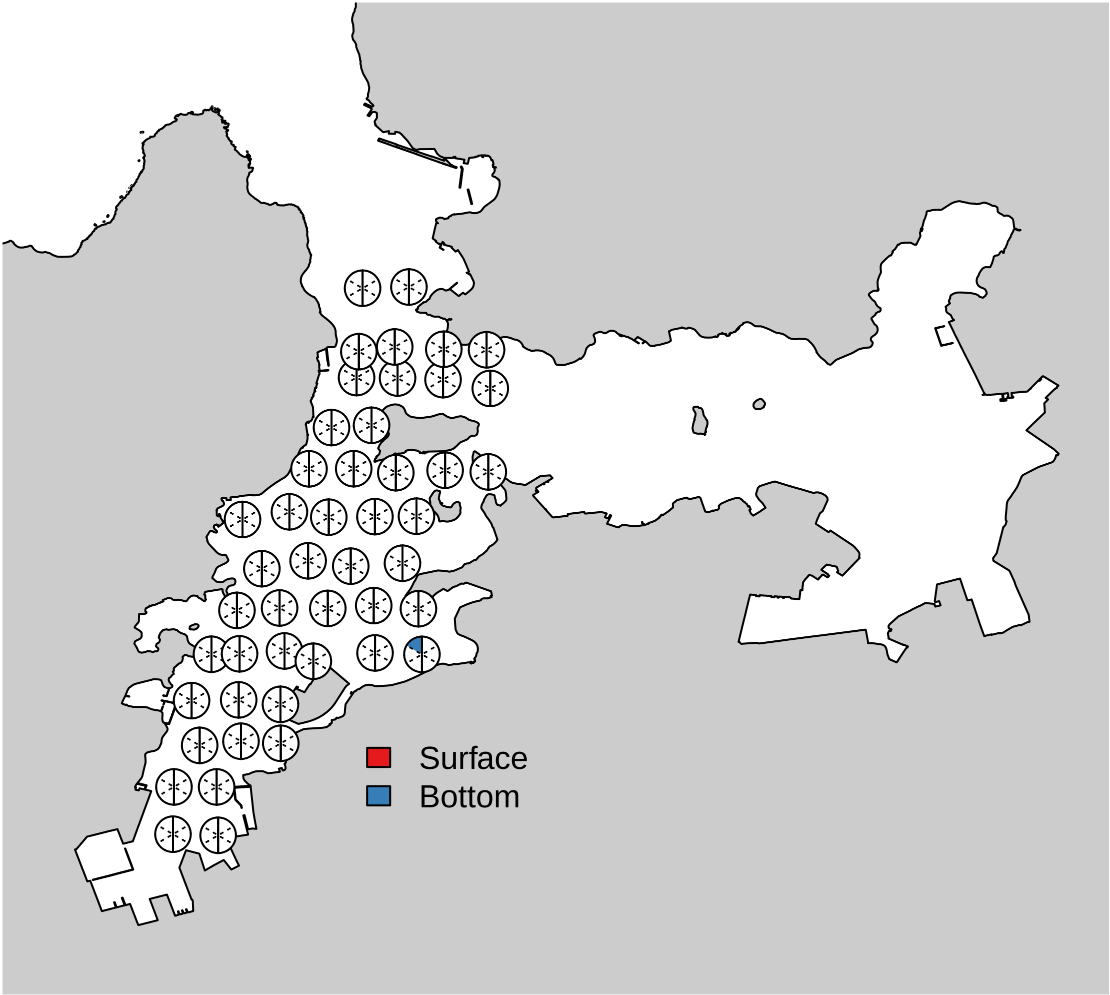

Fig.S1_Chelon_haematocheilus

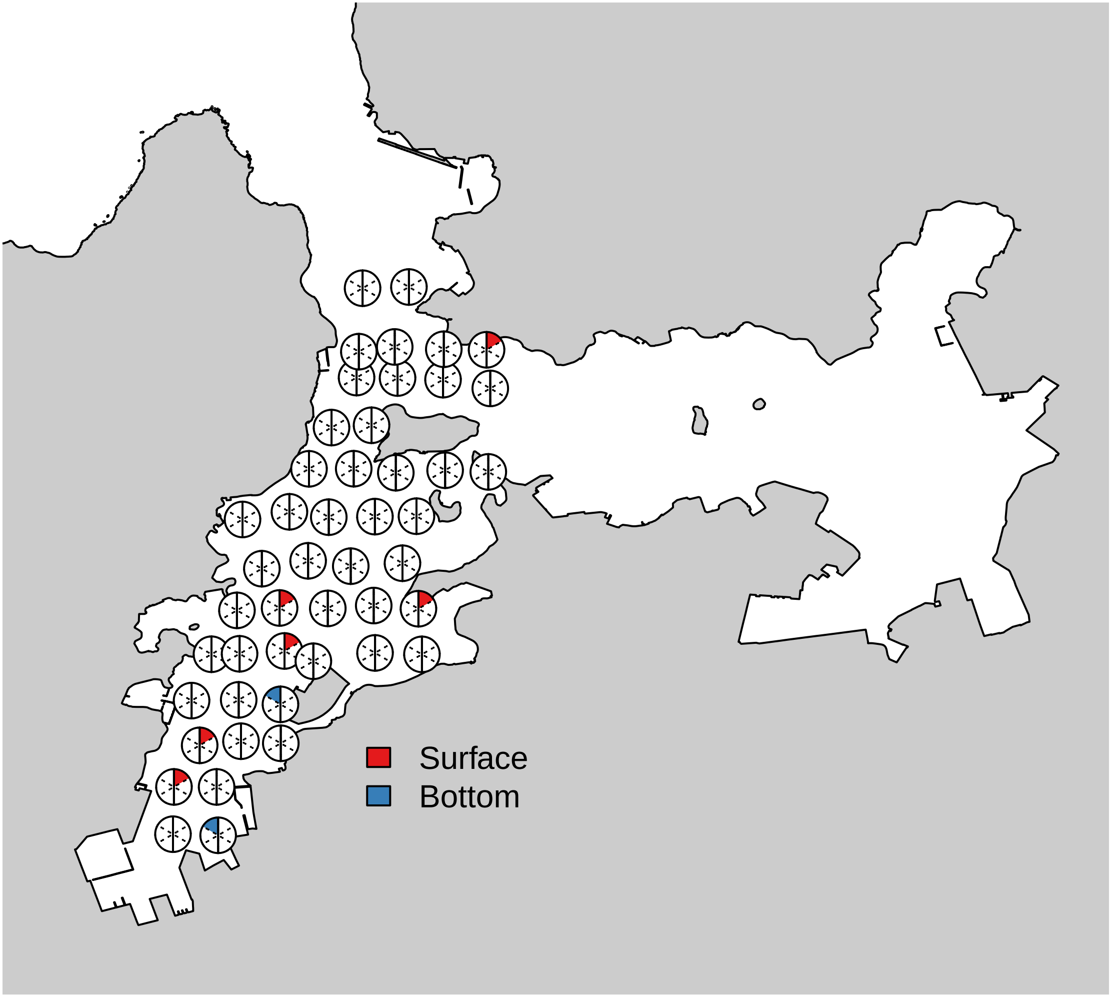

Fig.S1_Cottiusculus_nihonkaiensis

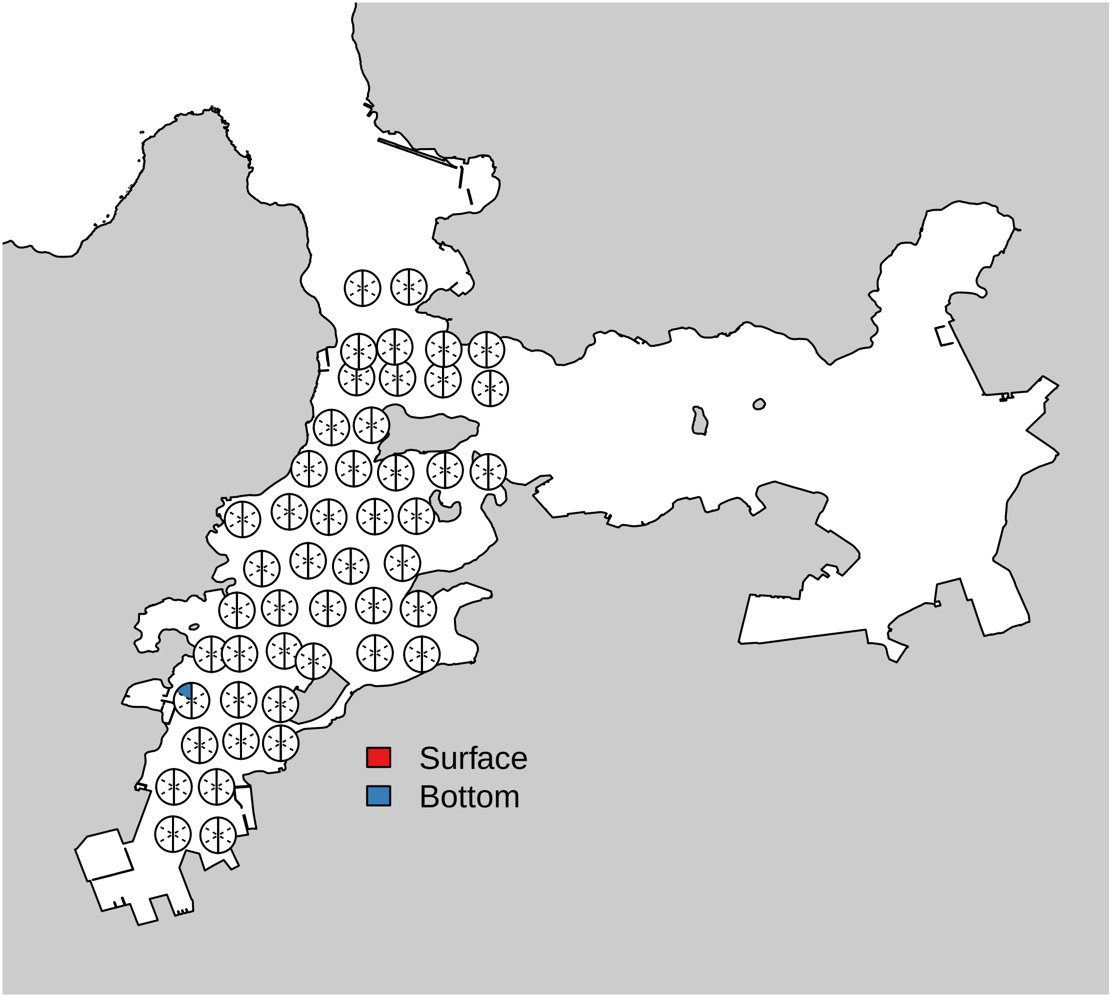

Fig.S1_Cyprinus_carpio

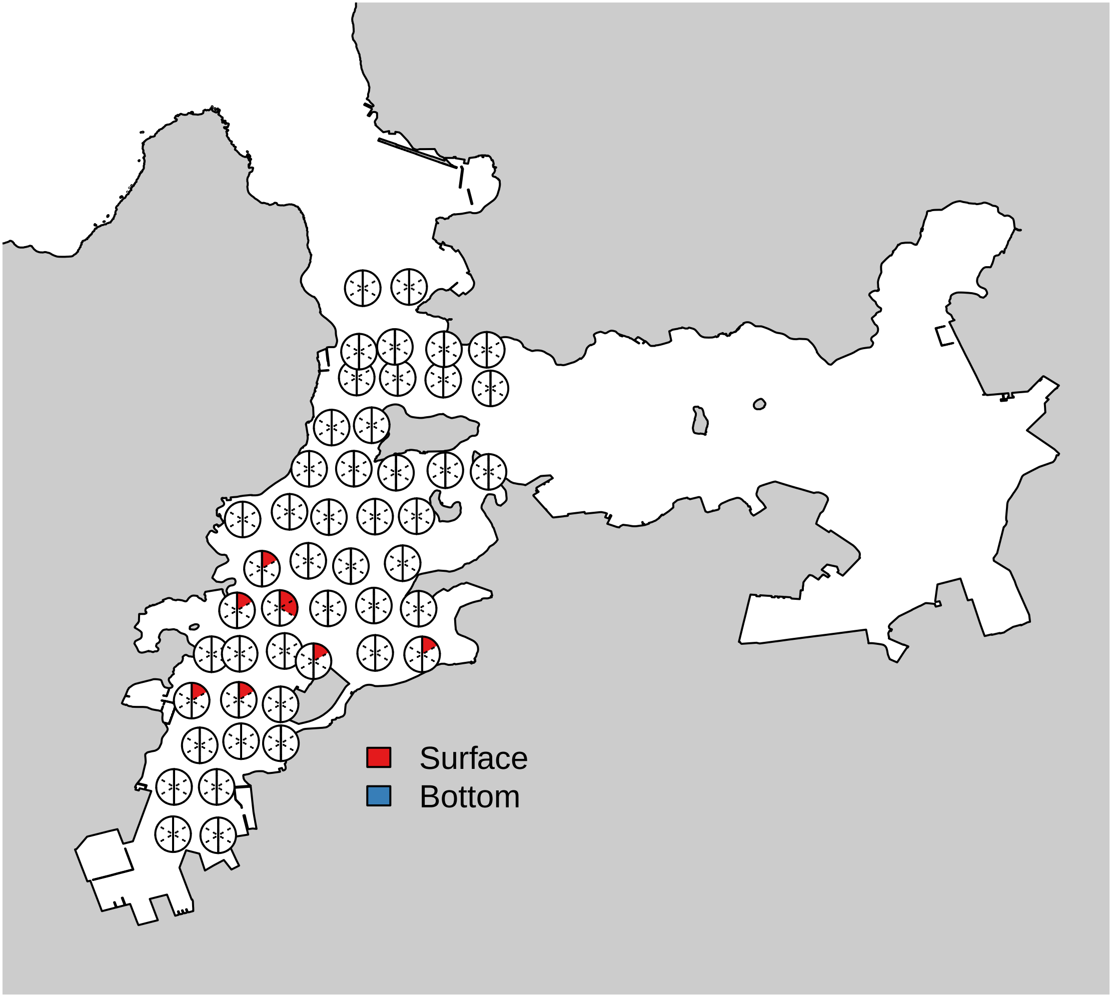

Fig.S1_Cypselurus_poecilopterus

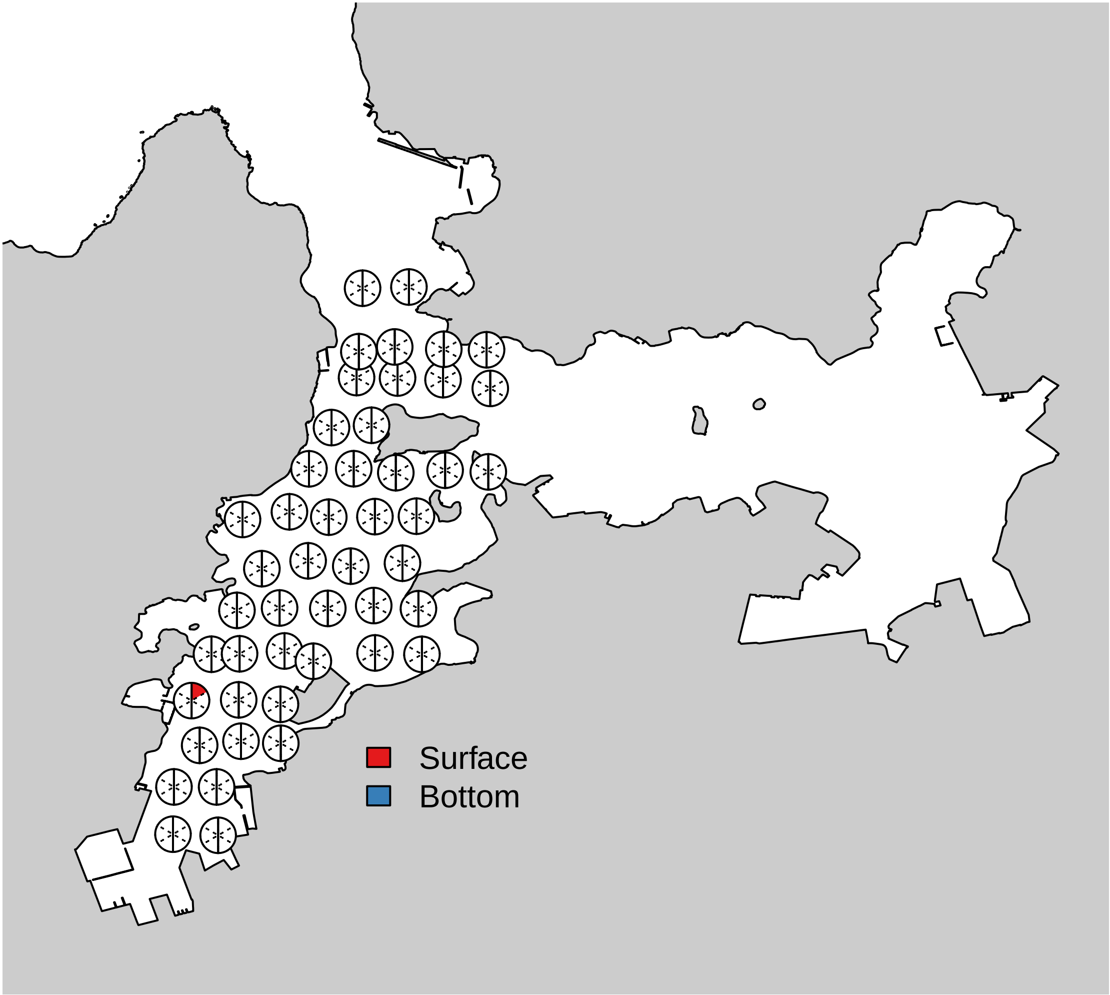

Fig.S1_Cypselurus_sp.1

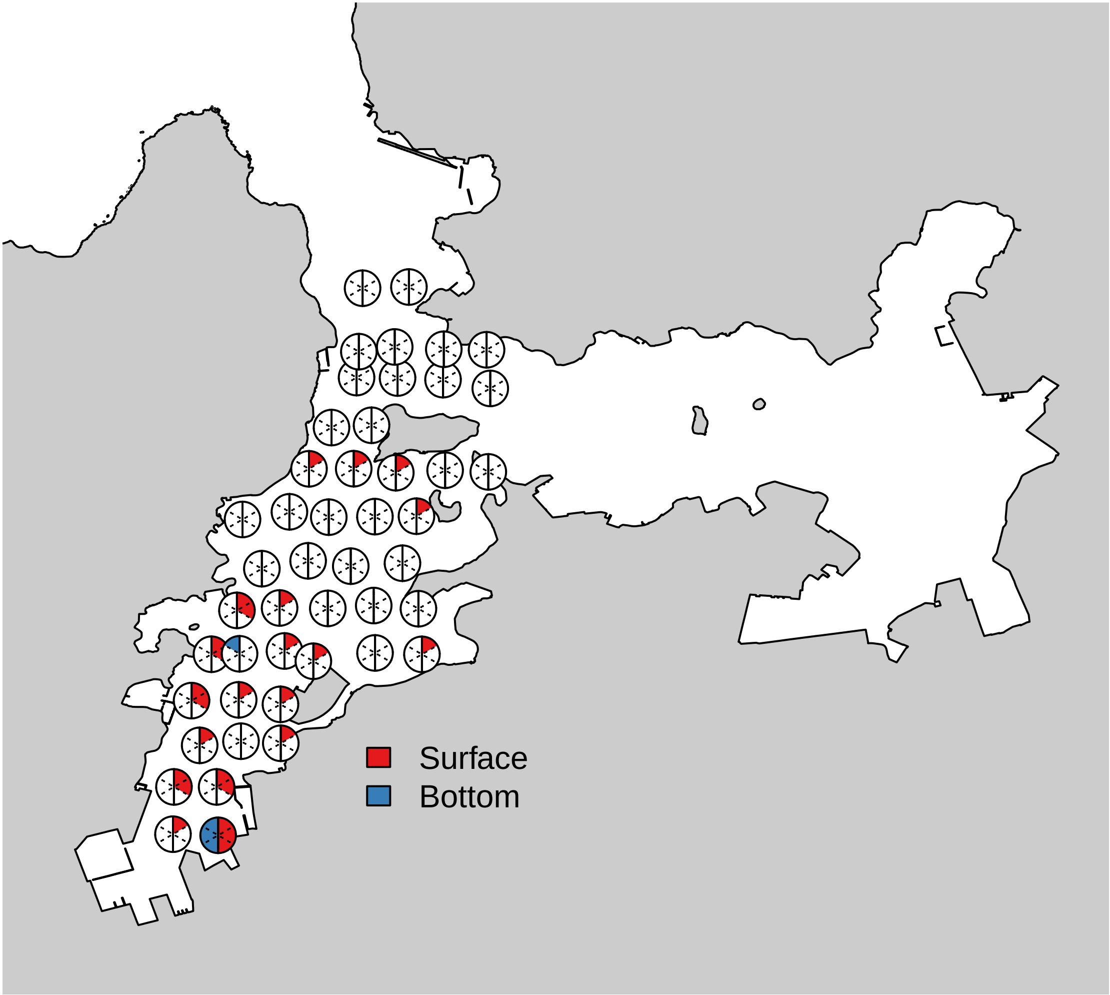

Fig.S1_Cypselurus_sp.2

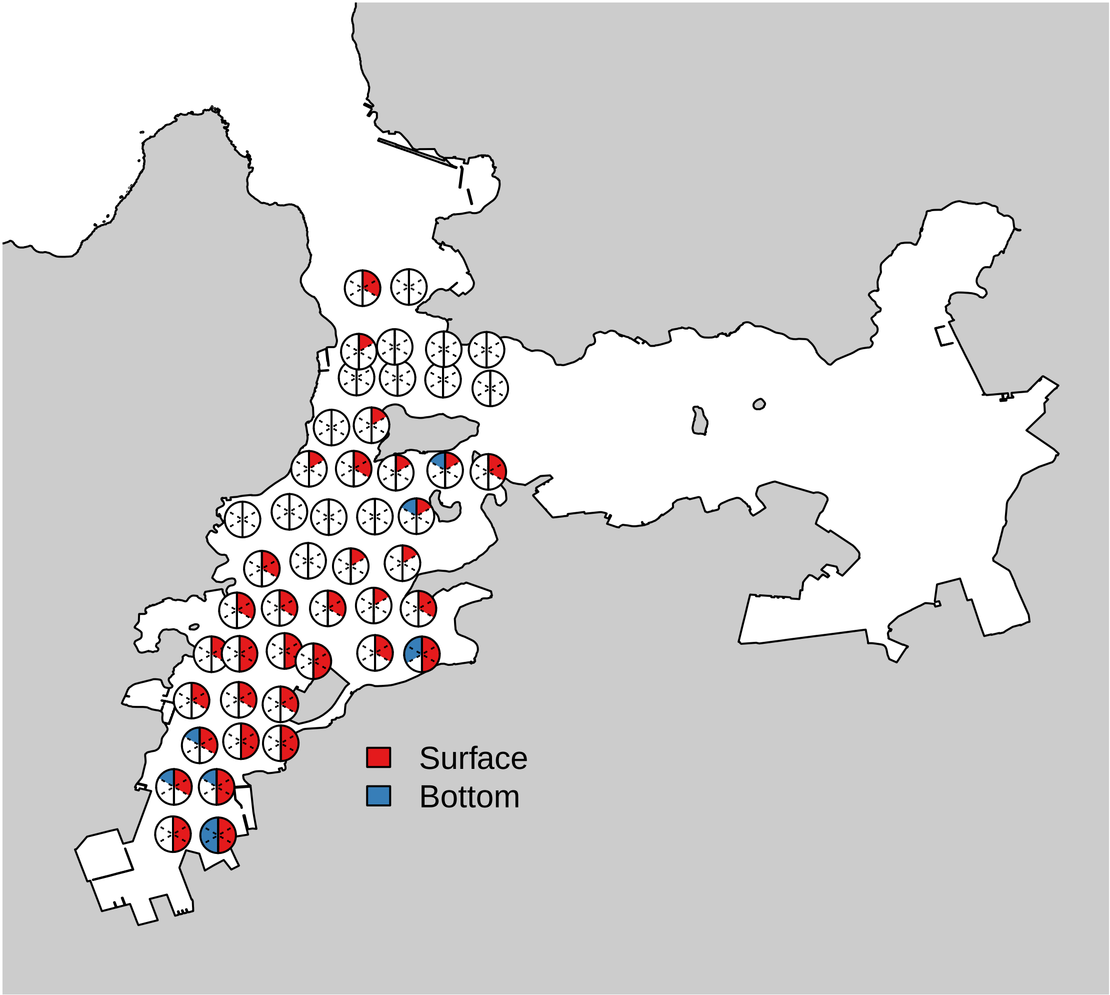

Fig.S1_Davidijordania_poecilimon

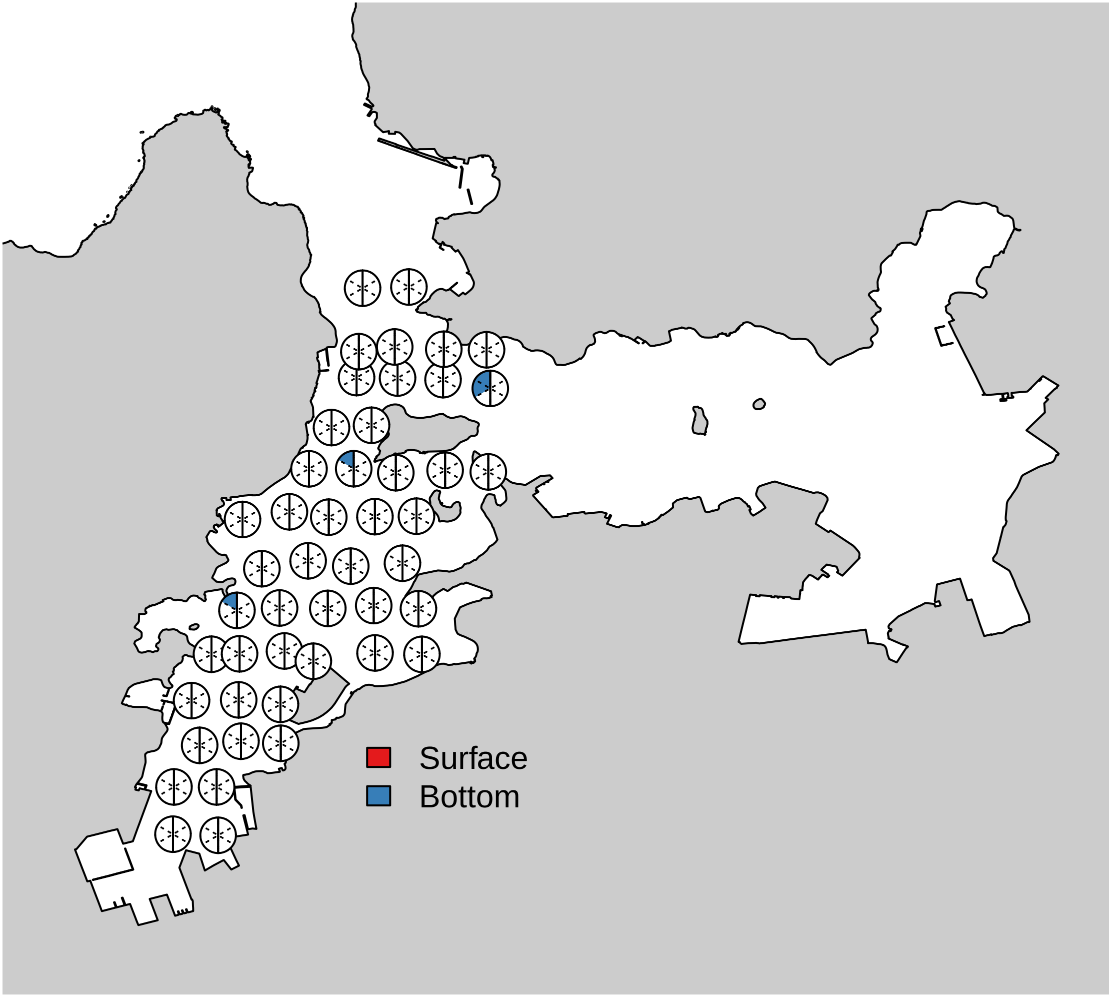

Fig.S1_Decapterus_maruadsi

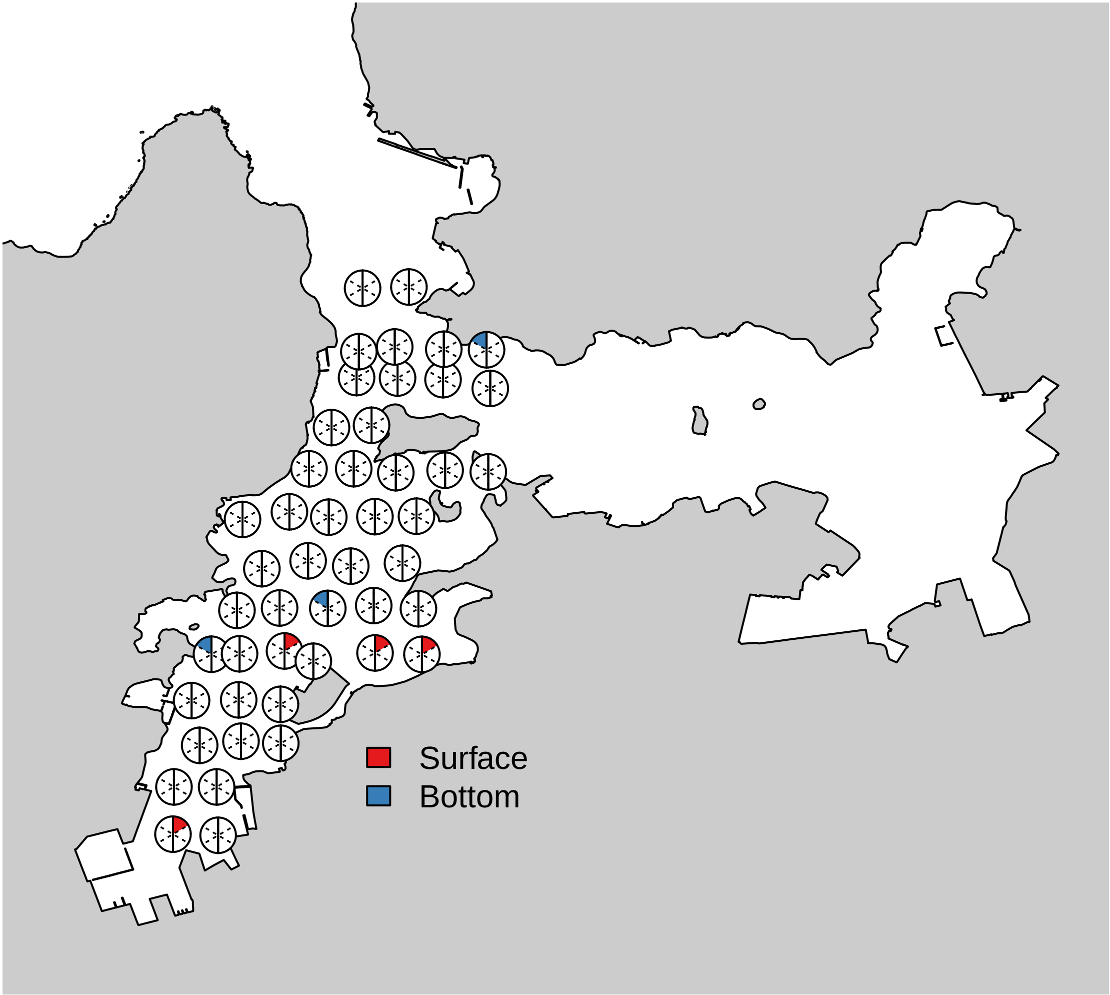

Fig.S1_Dentex_hypselosomus

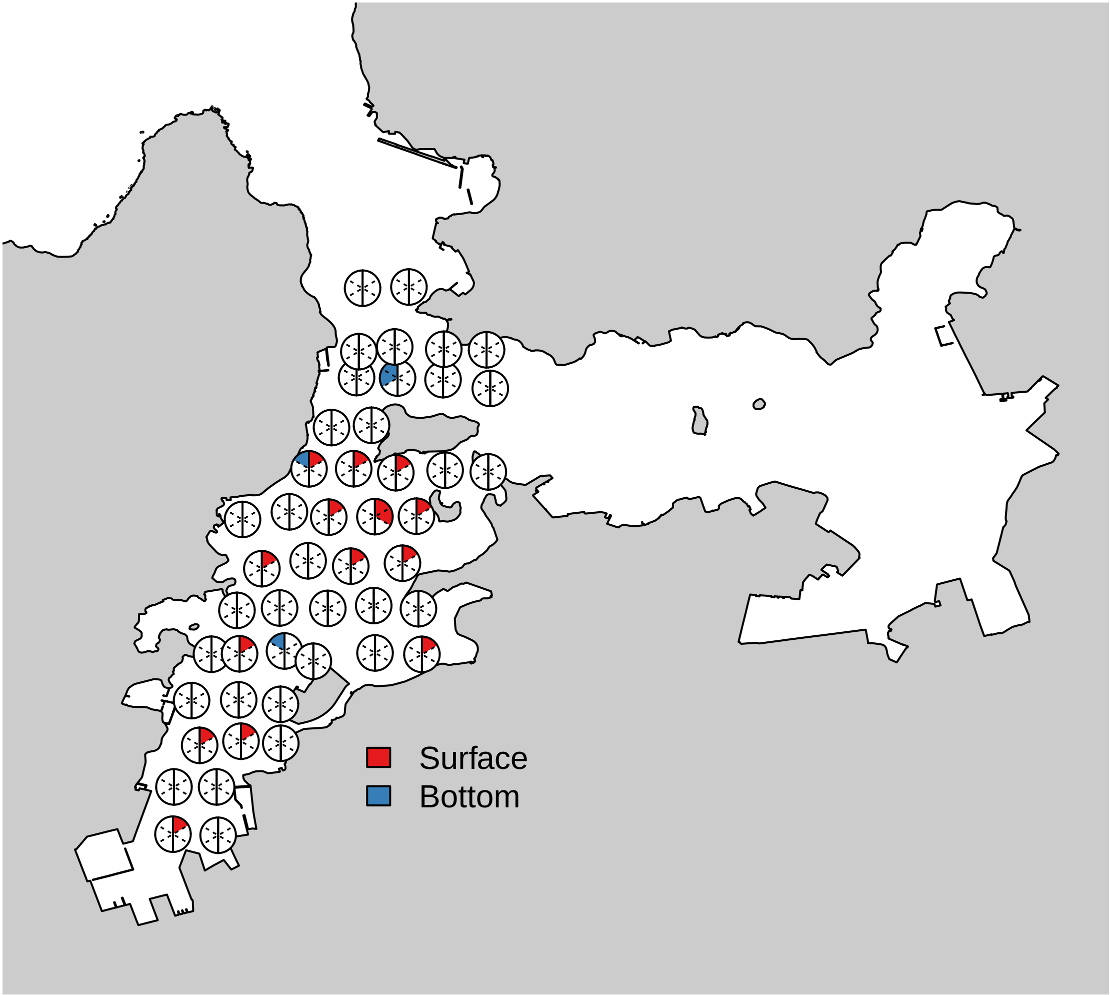

Fig.S1_Dictyosoma_burgeri

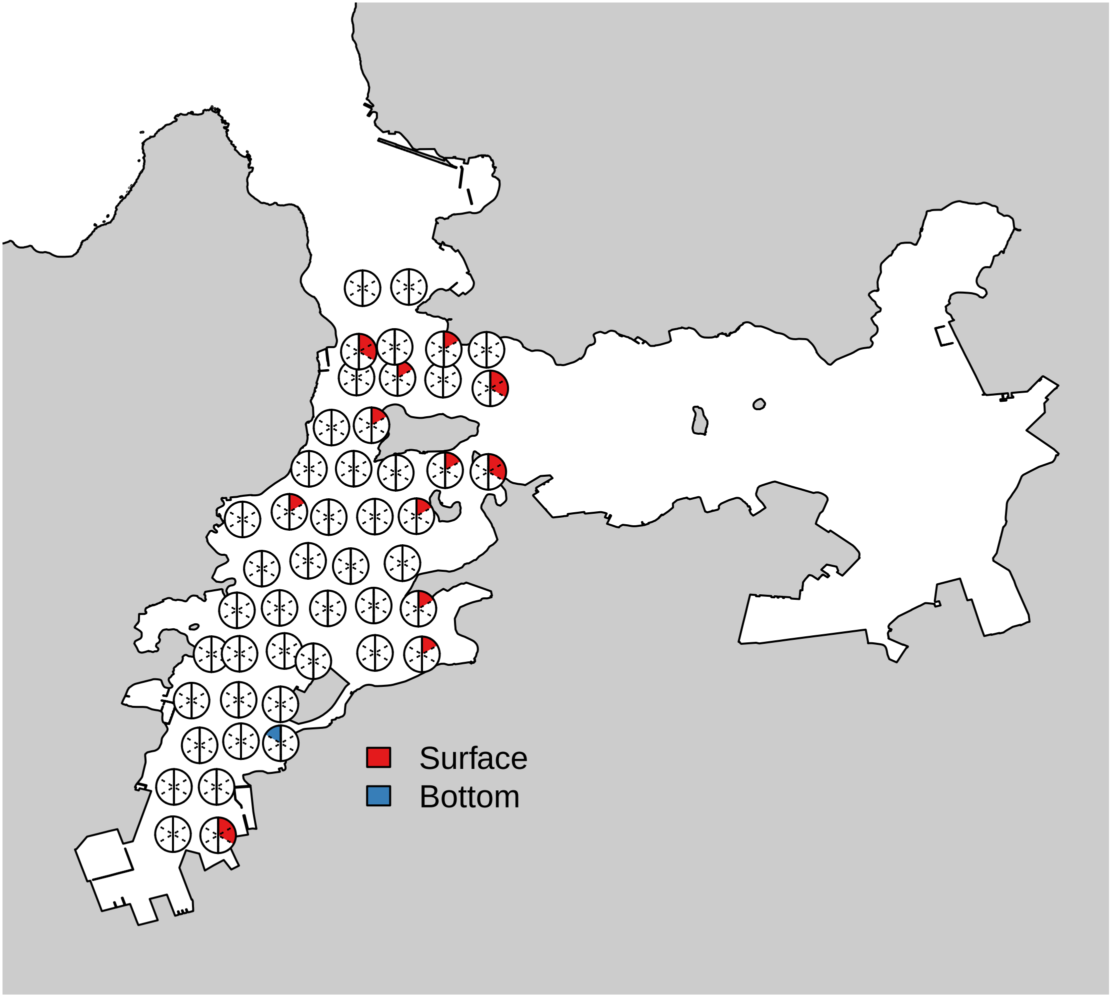

Fig.S1_Dipturus_kwangtungensis

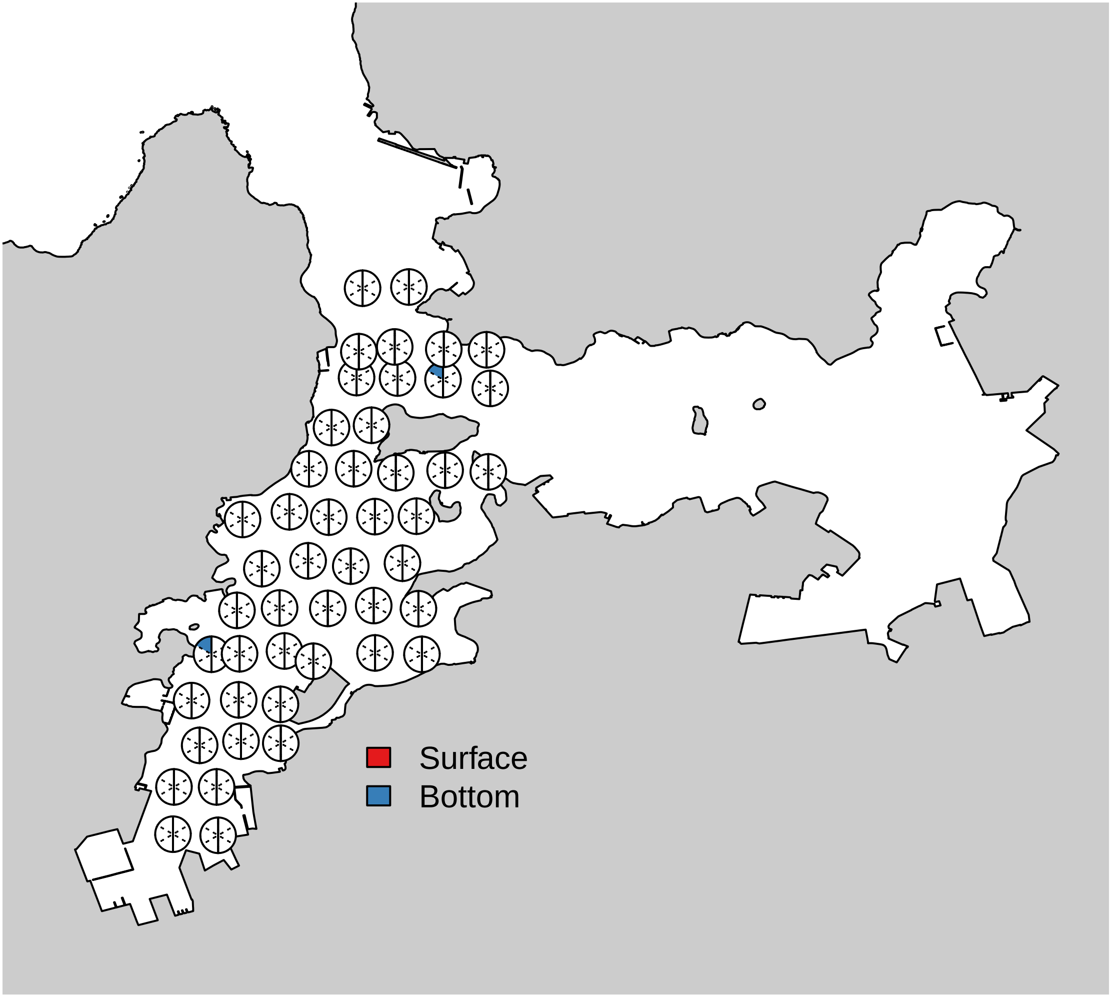

Fig.S1_Ditrema_temminckii_temminckii

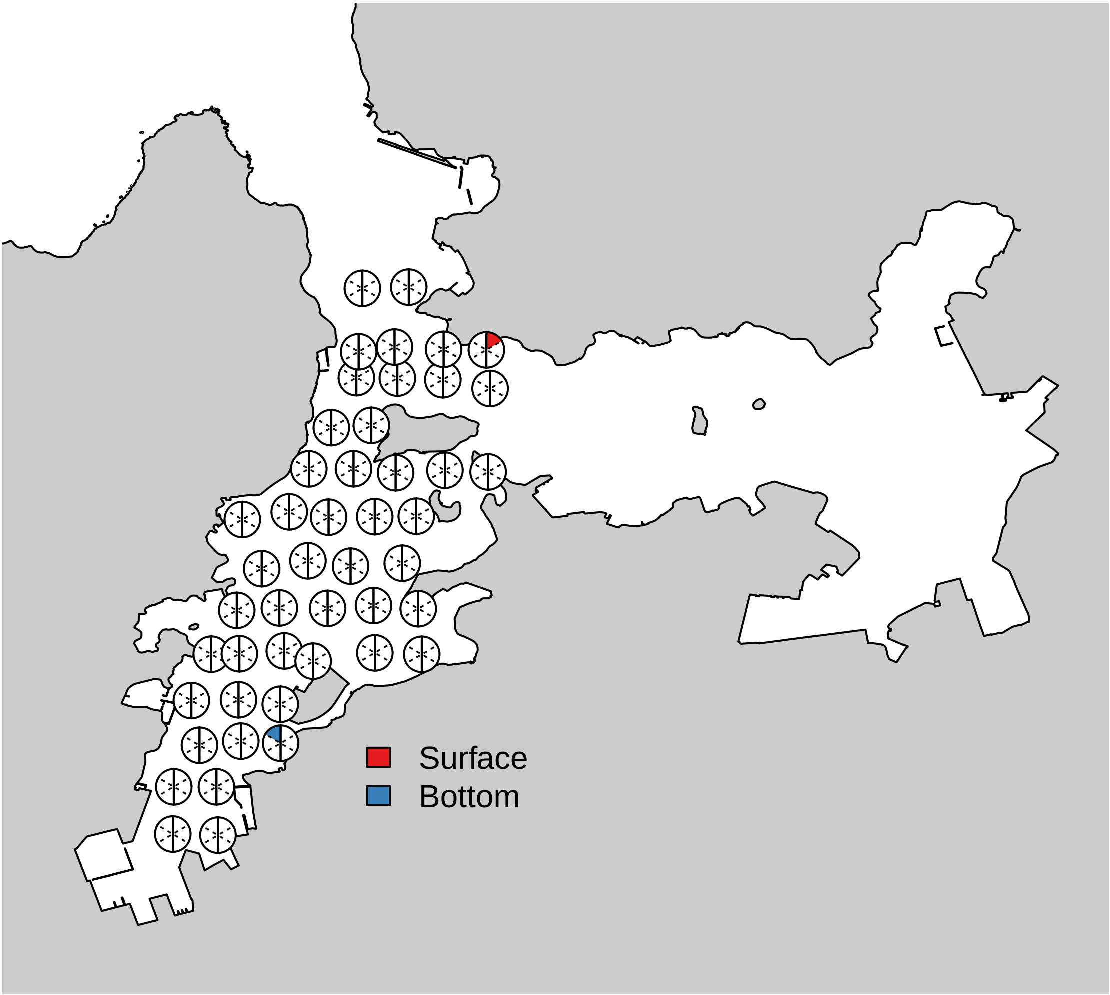

Fig.S1_Engraulis_japonicus

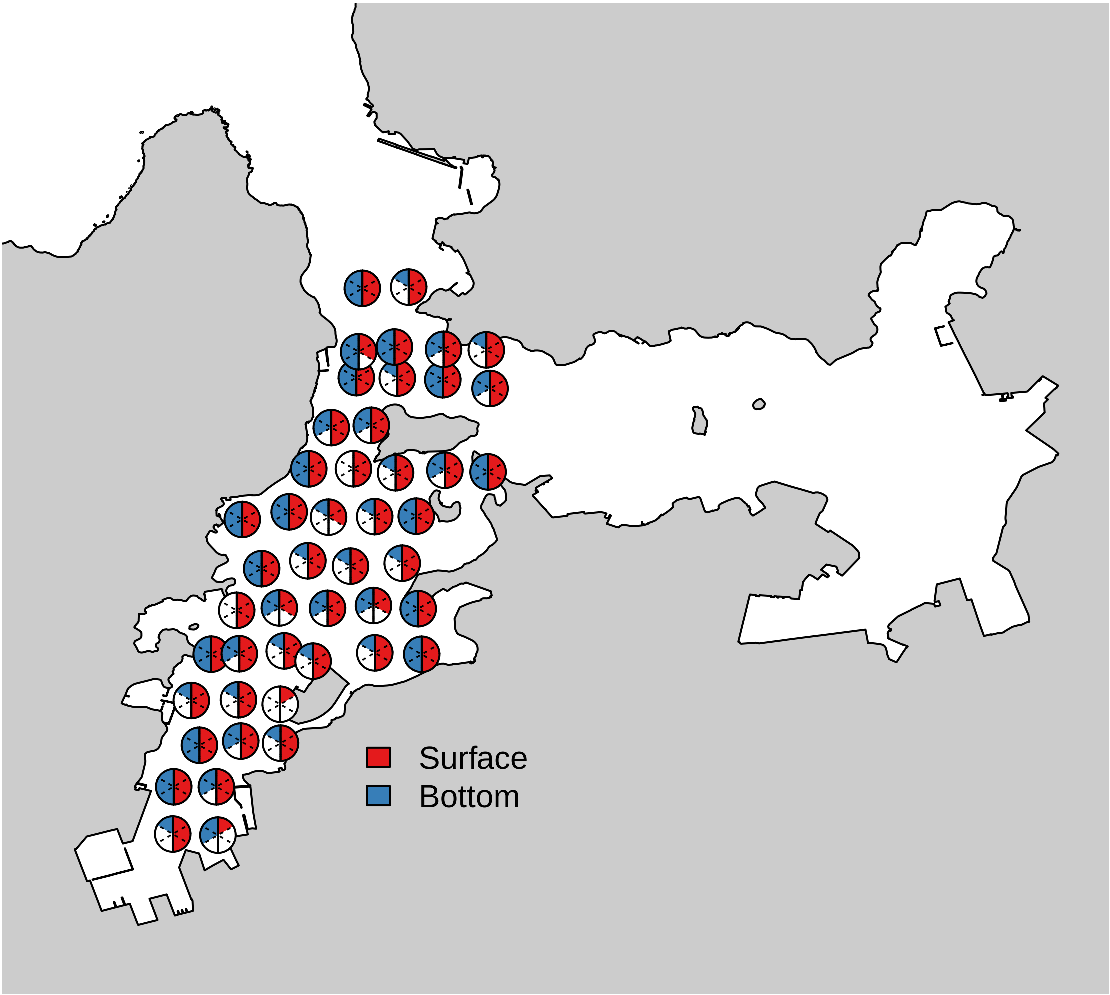

Fig.S1_Epinephelus_akaara

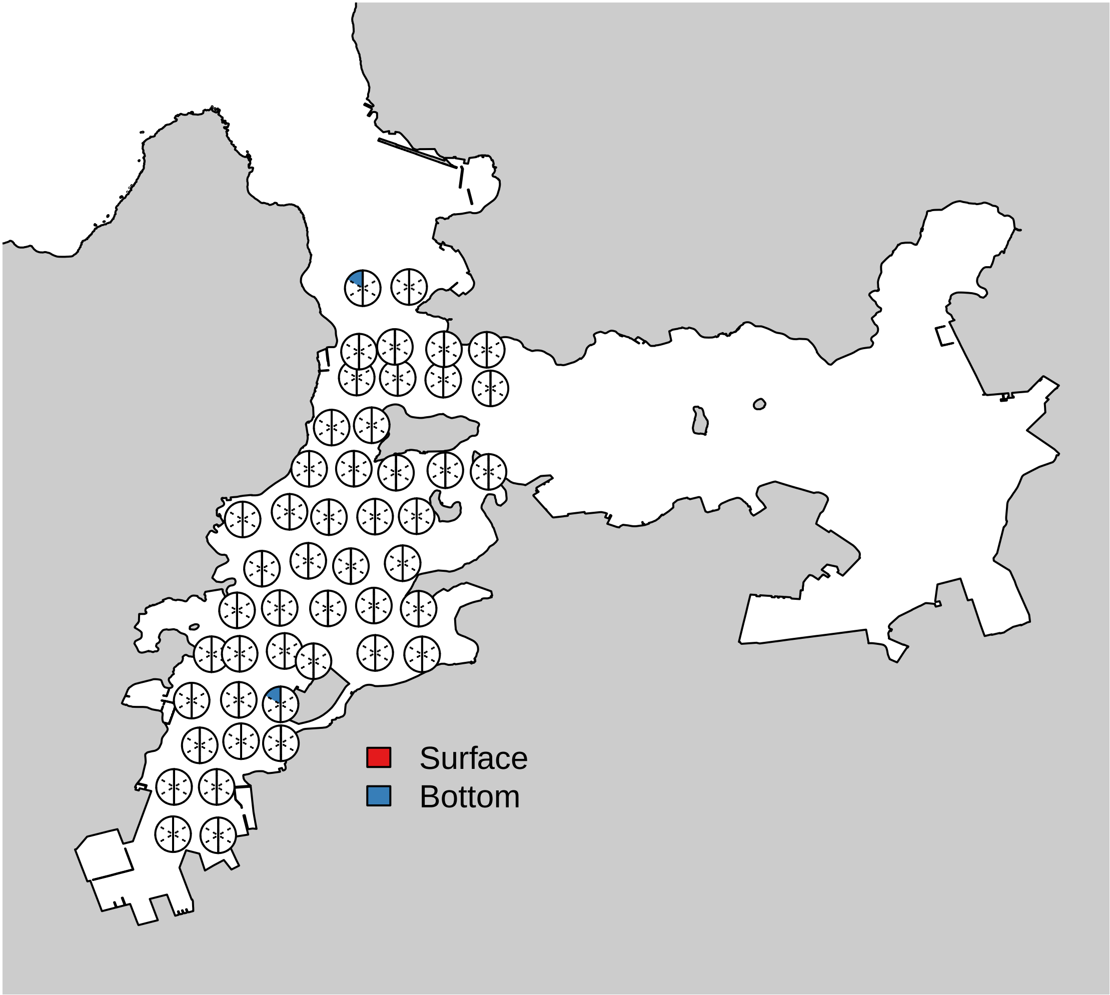

Fig.S1_Epinephelus_awoara

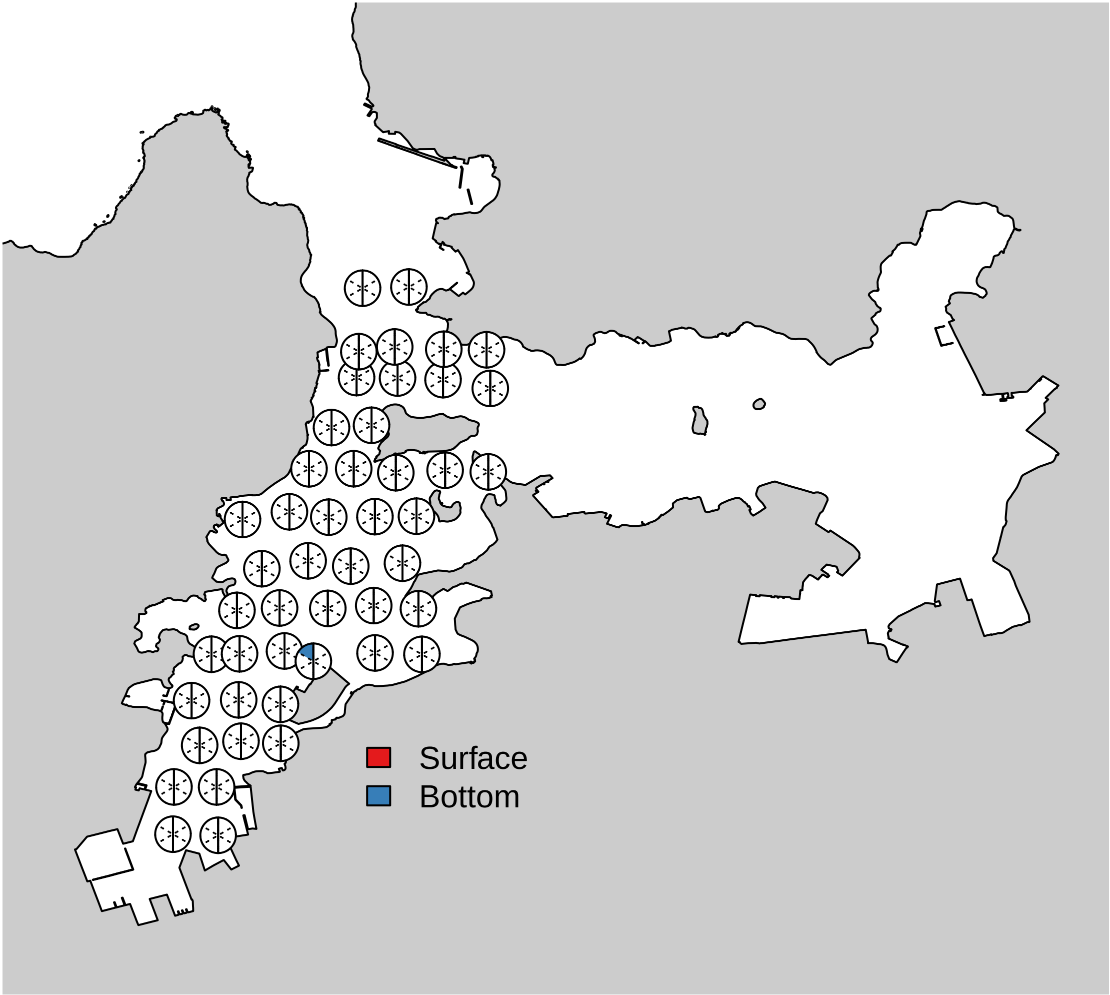

Fig.S1_Ernogrammus_hexagrammus

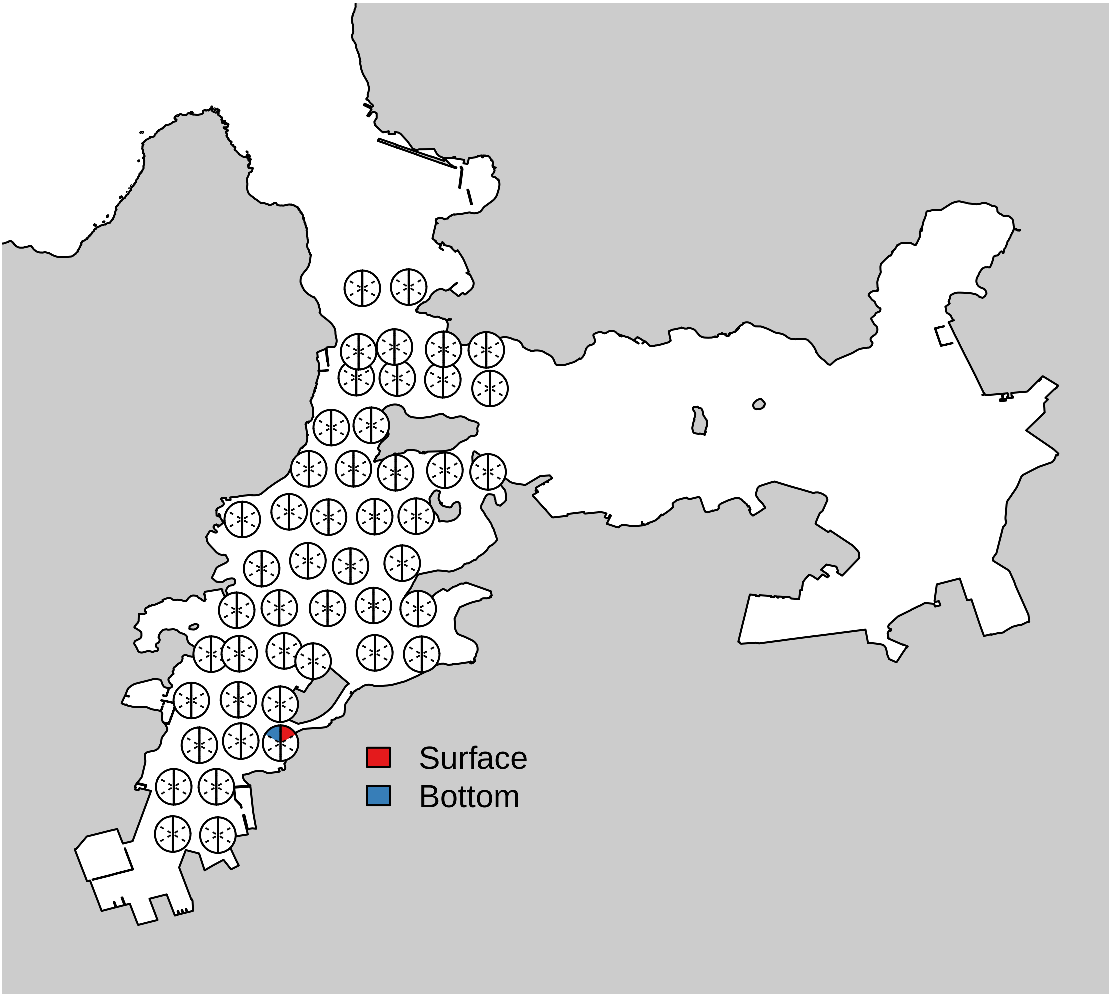

Fig.S1_Etrumeus_teres

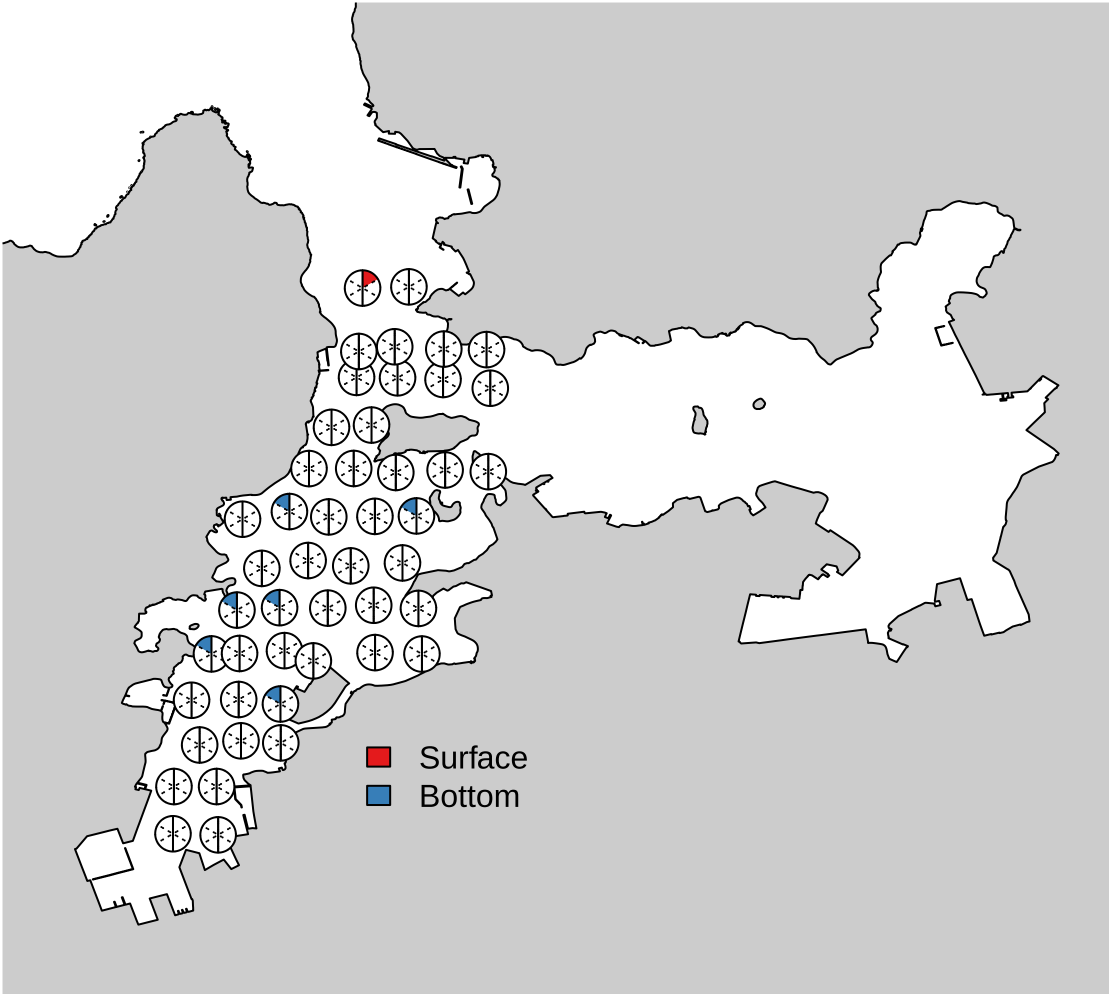

Fig.S1_Eviota_abax

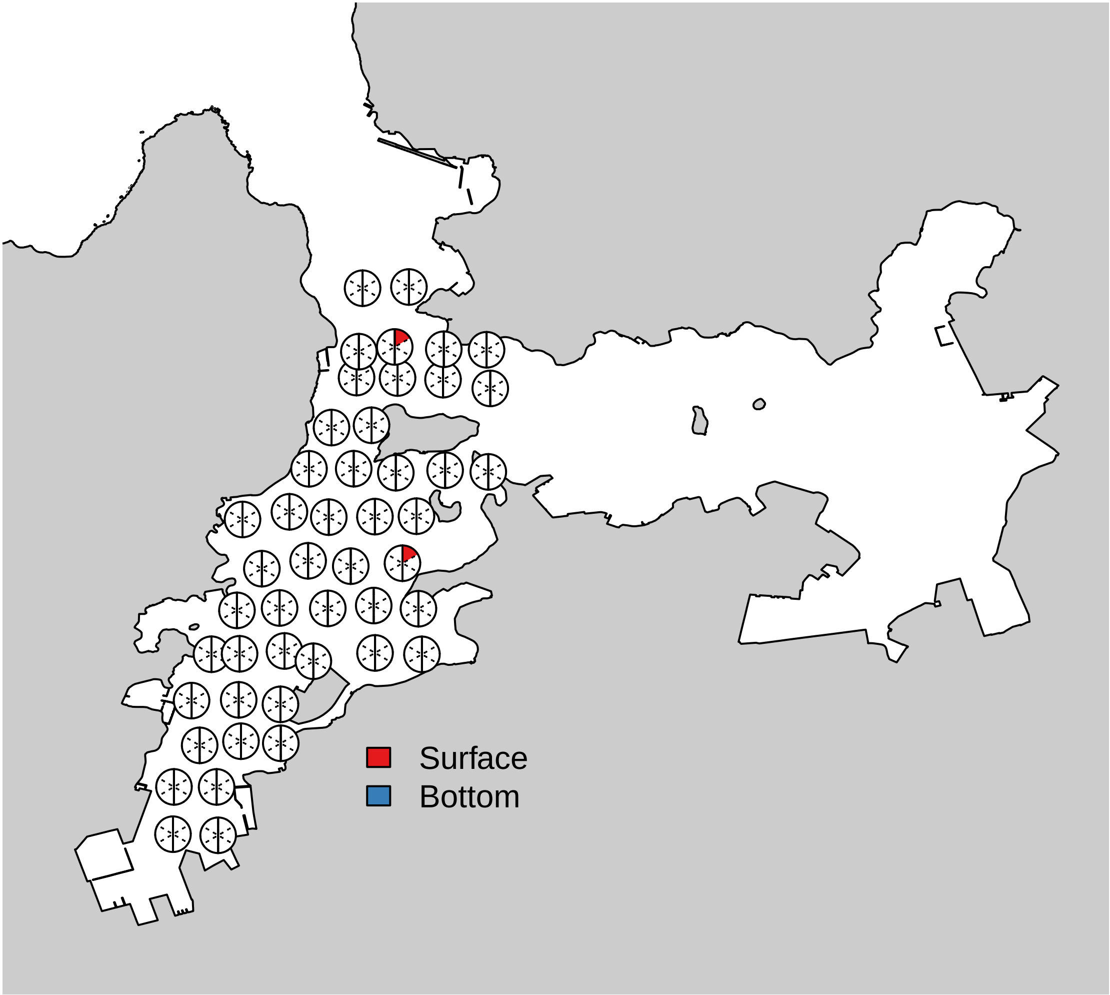

Fig.S1_Gadus_chalcogrammus

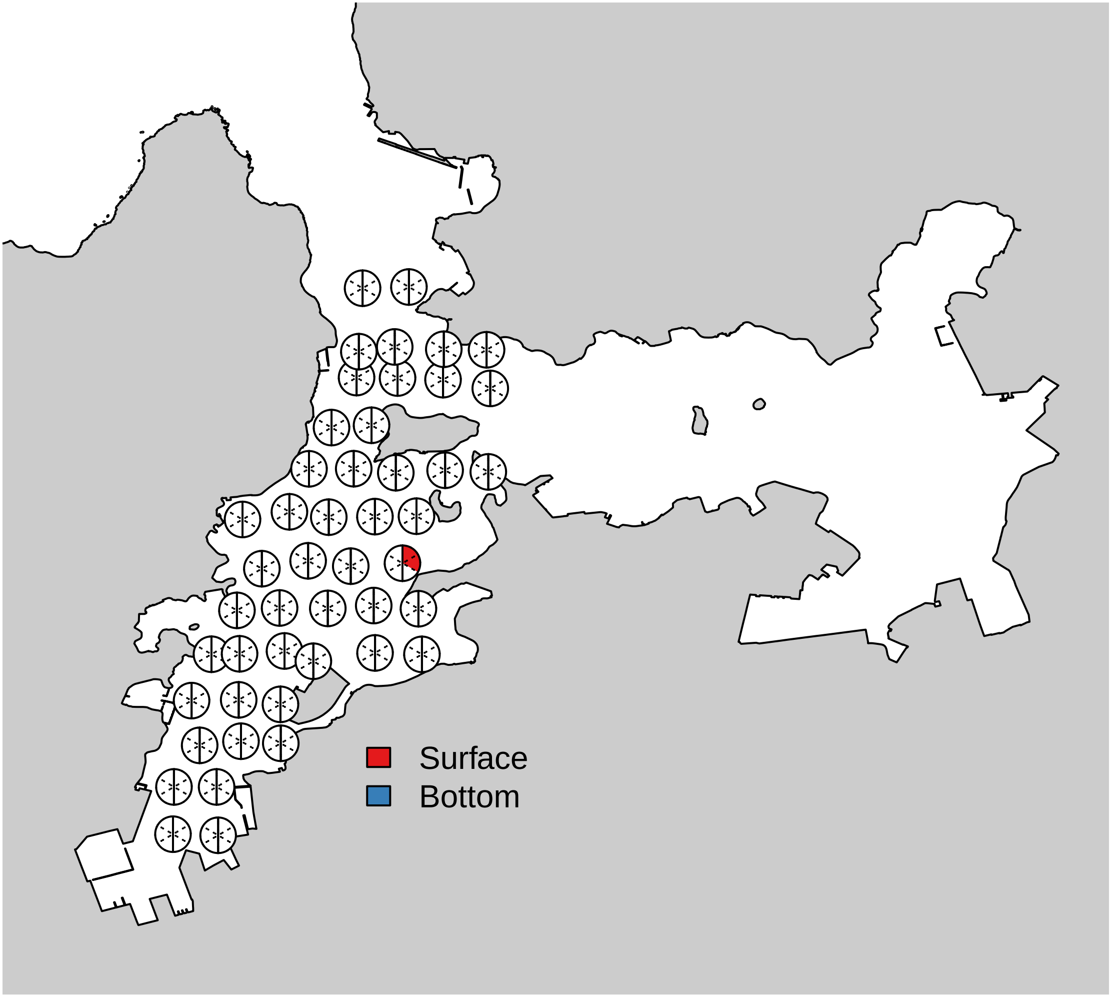

Fig.S1_Girella_punctata

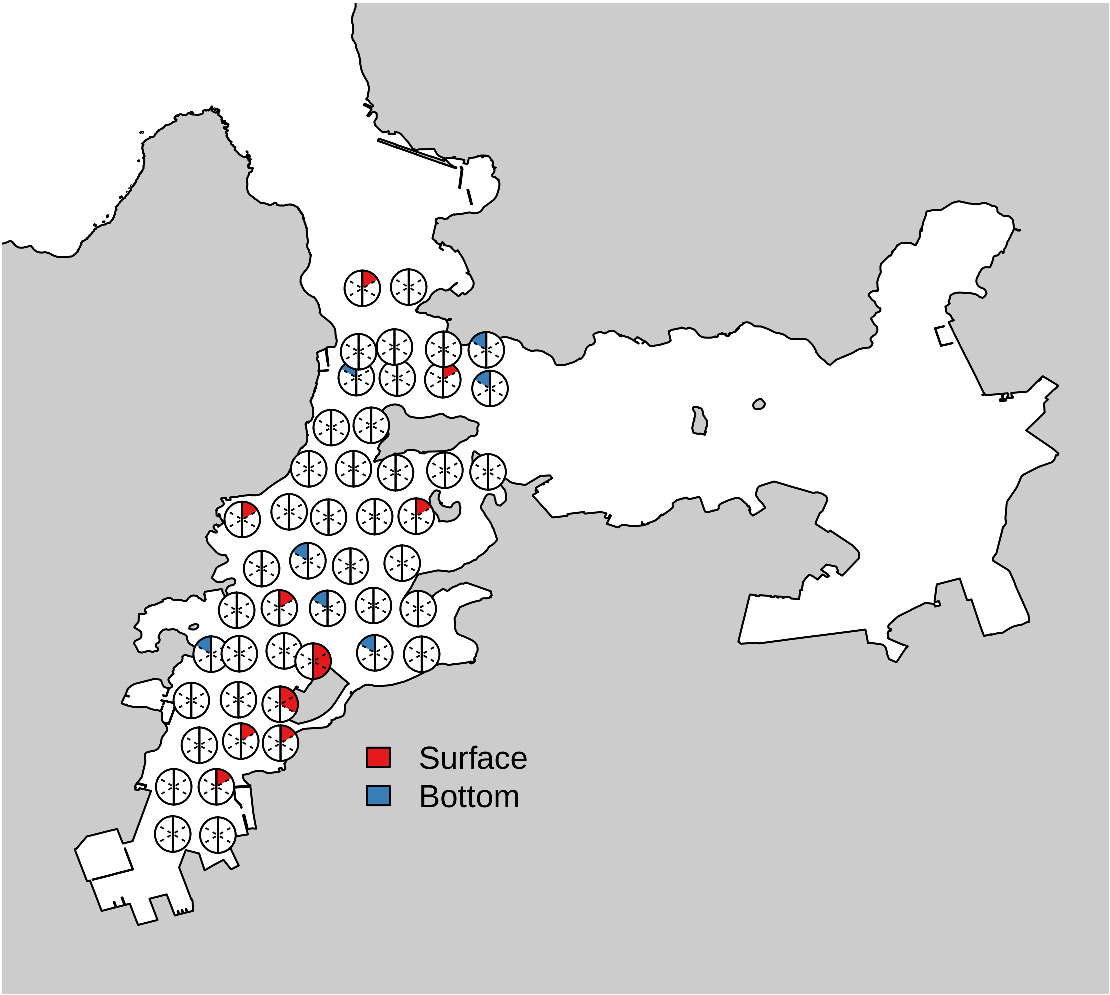

Fig.S1_Glossogobius_olivaceus

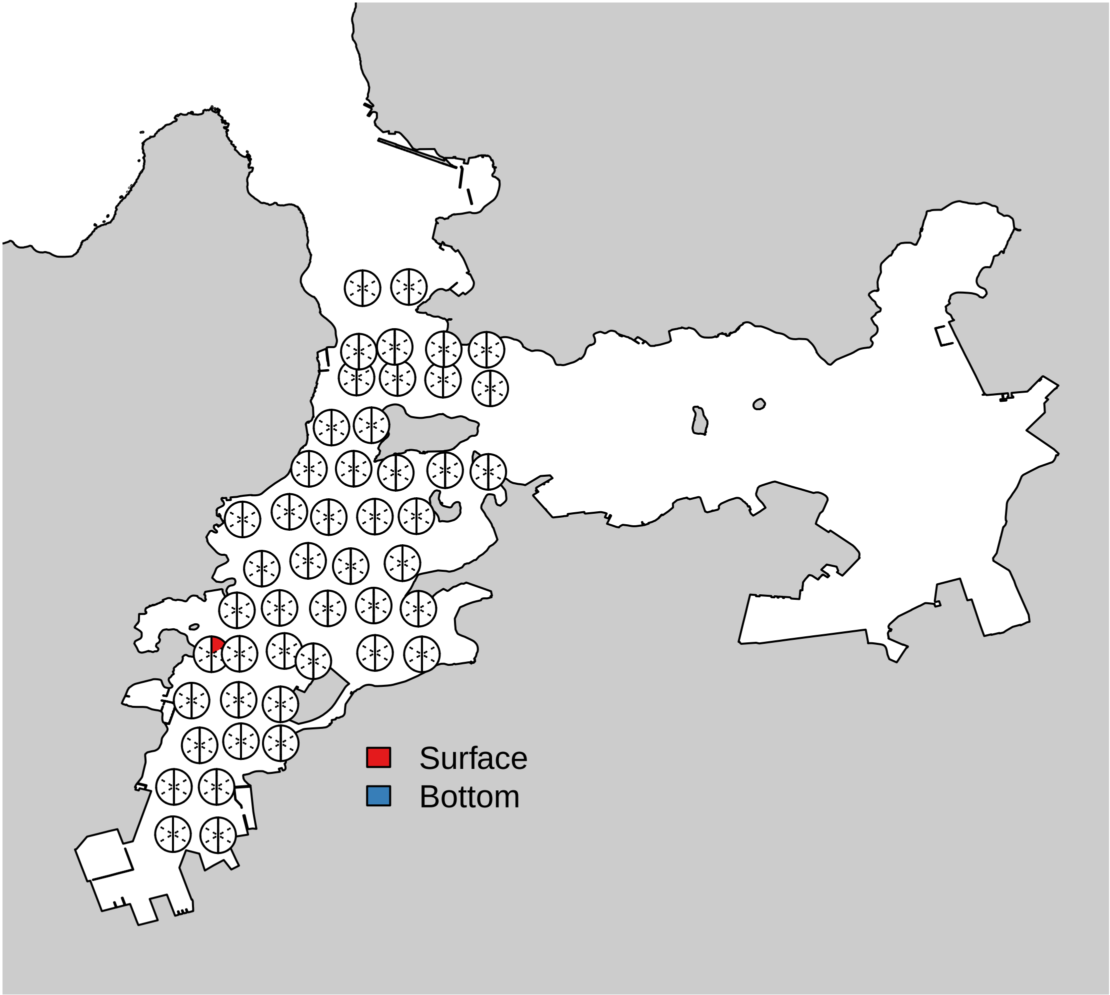

Fig.S1_Glyptocephalus_stelleri

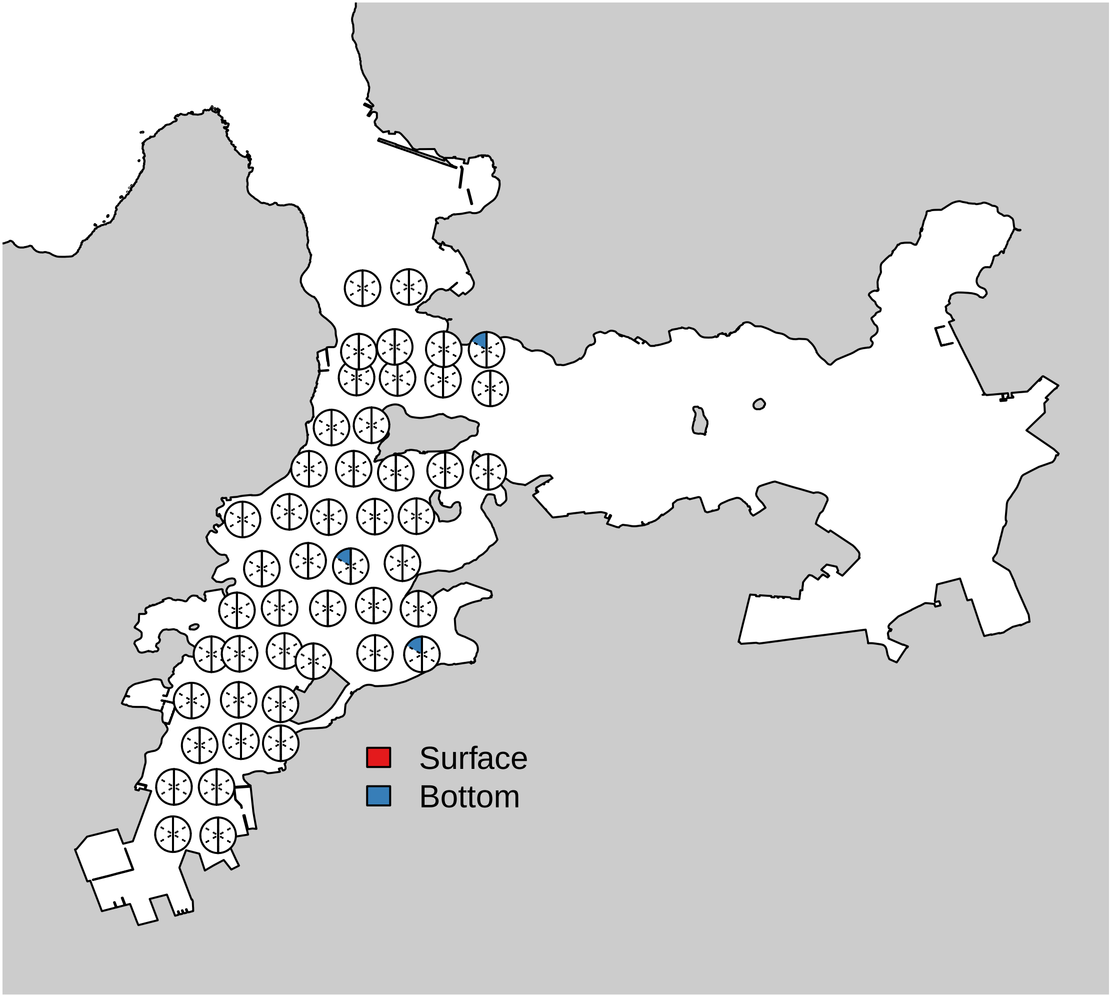

Fig.S1_Gnathopogon_elongatus_elongatus

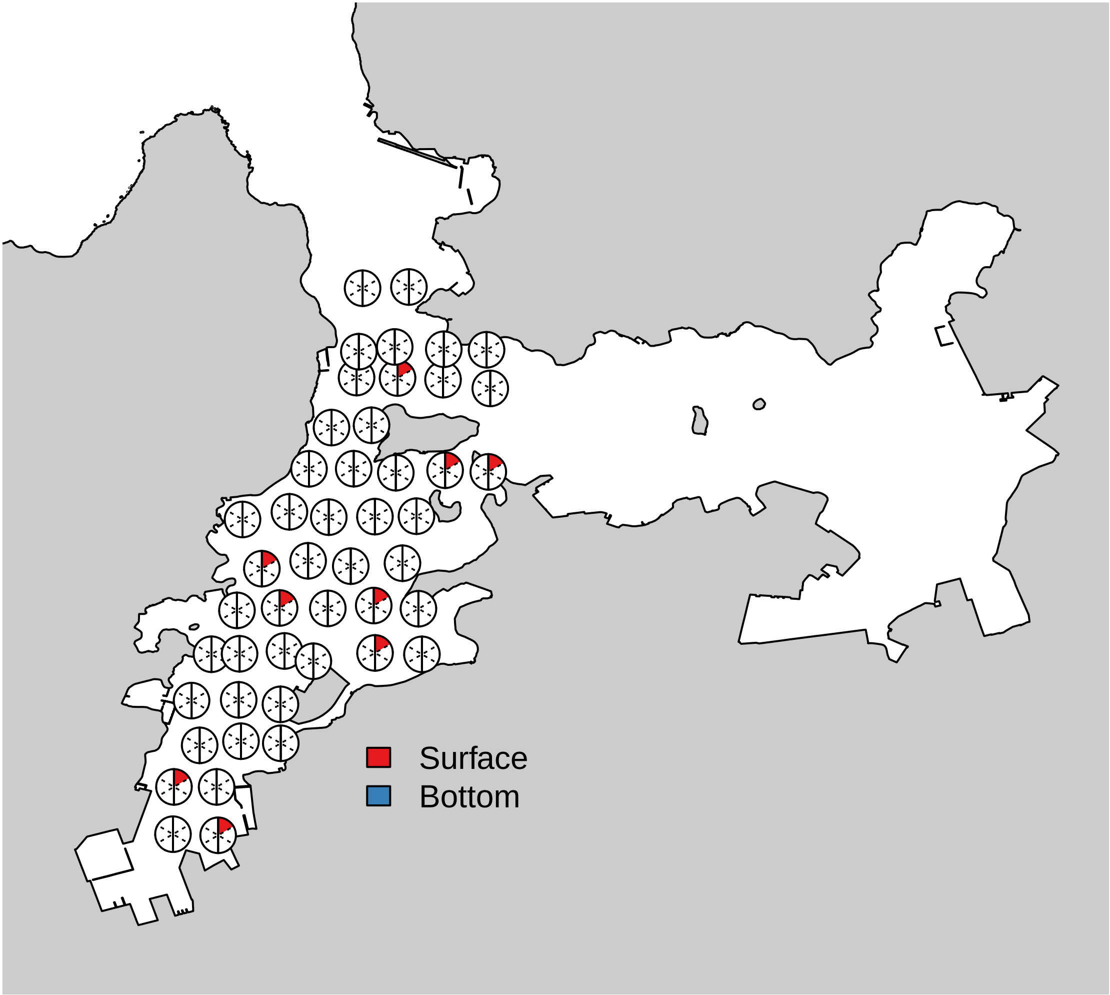

Fig.S1_Gobiid_species

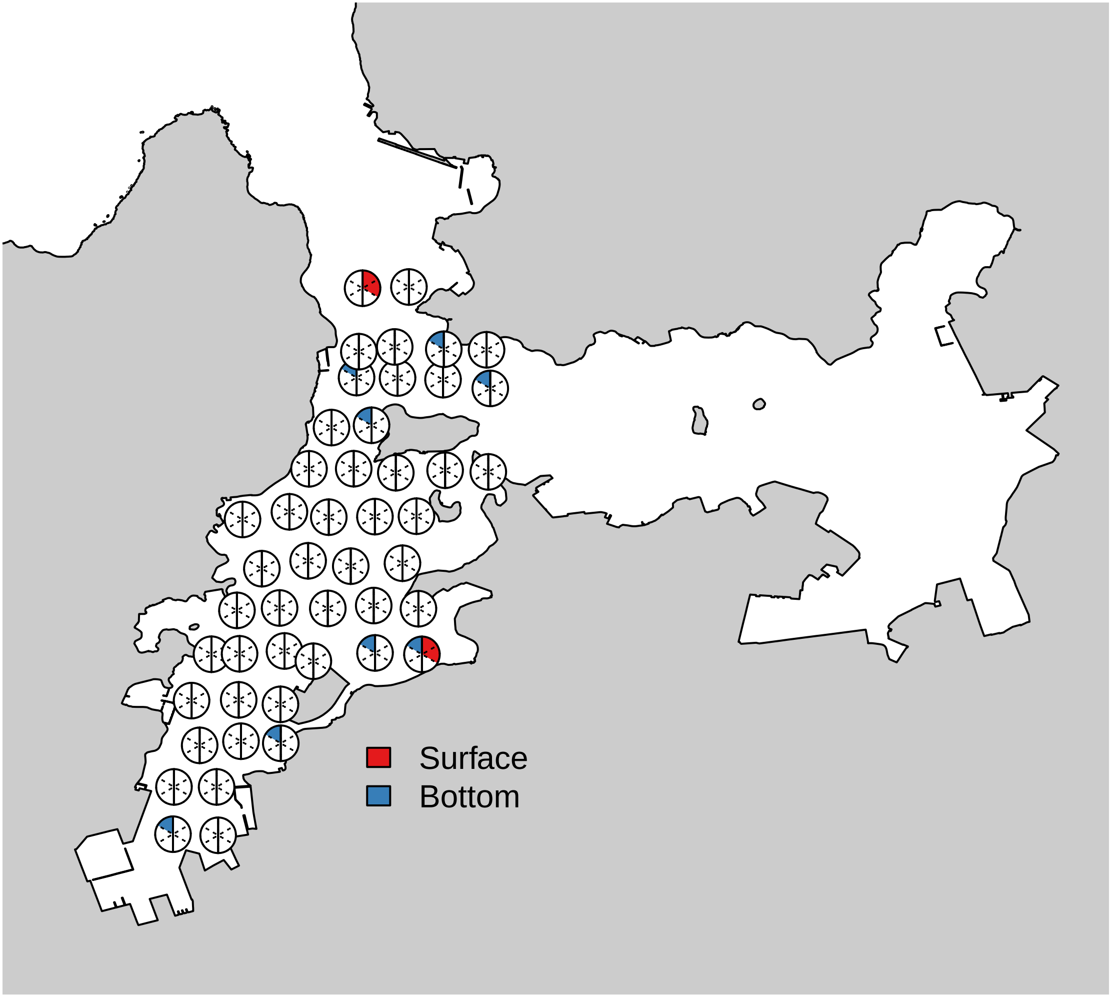

Fig.S1_Gymnogobius_breunigii

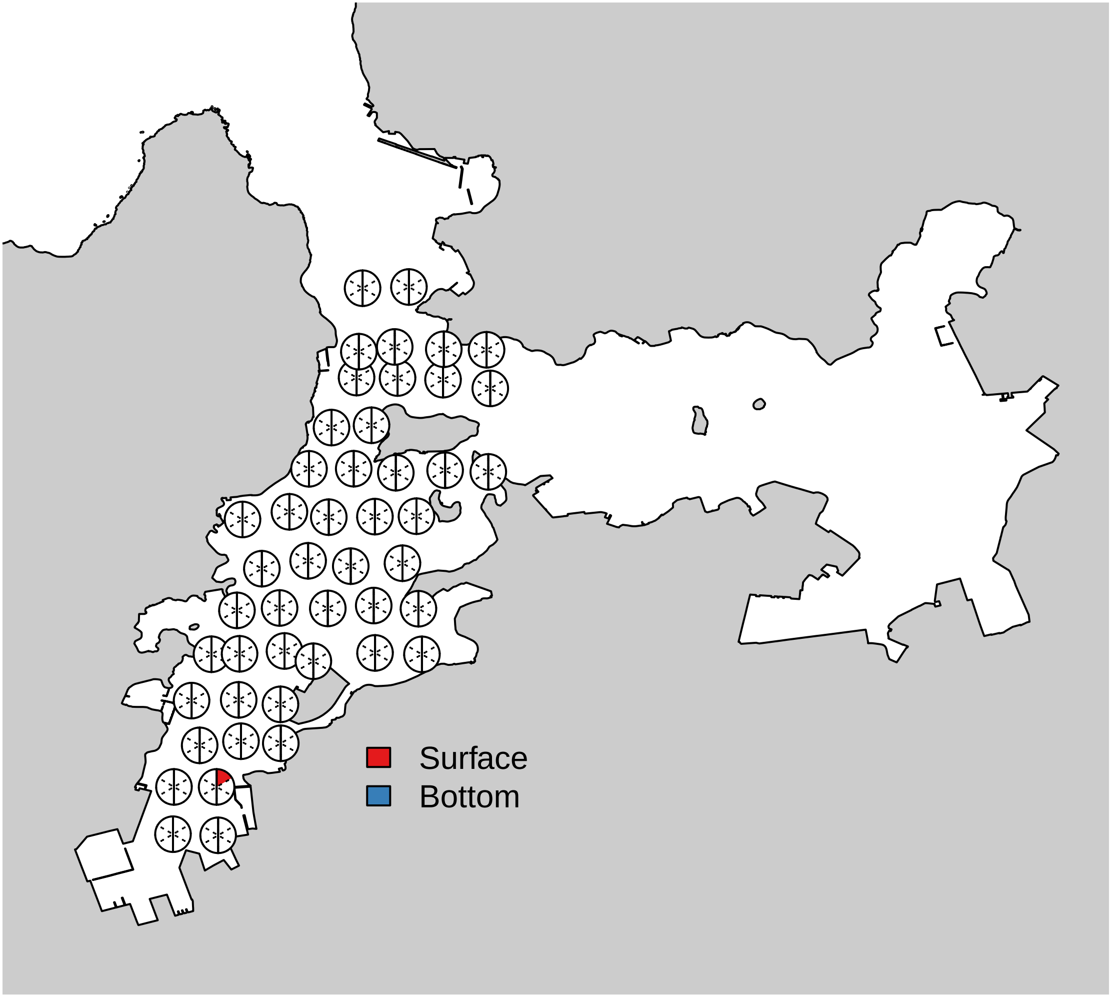

Fig.S1_Halichoeres_tenuispinis

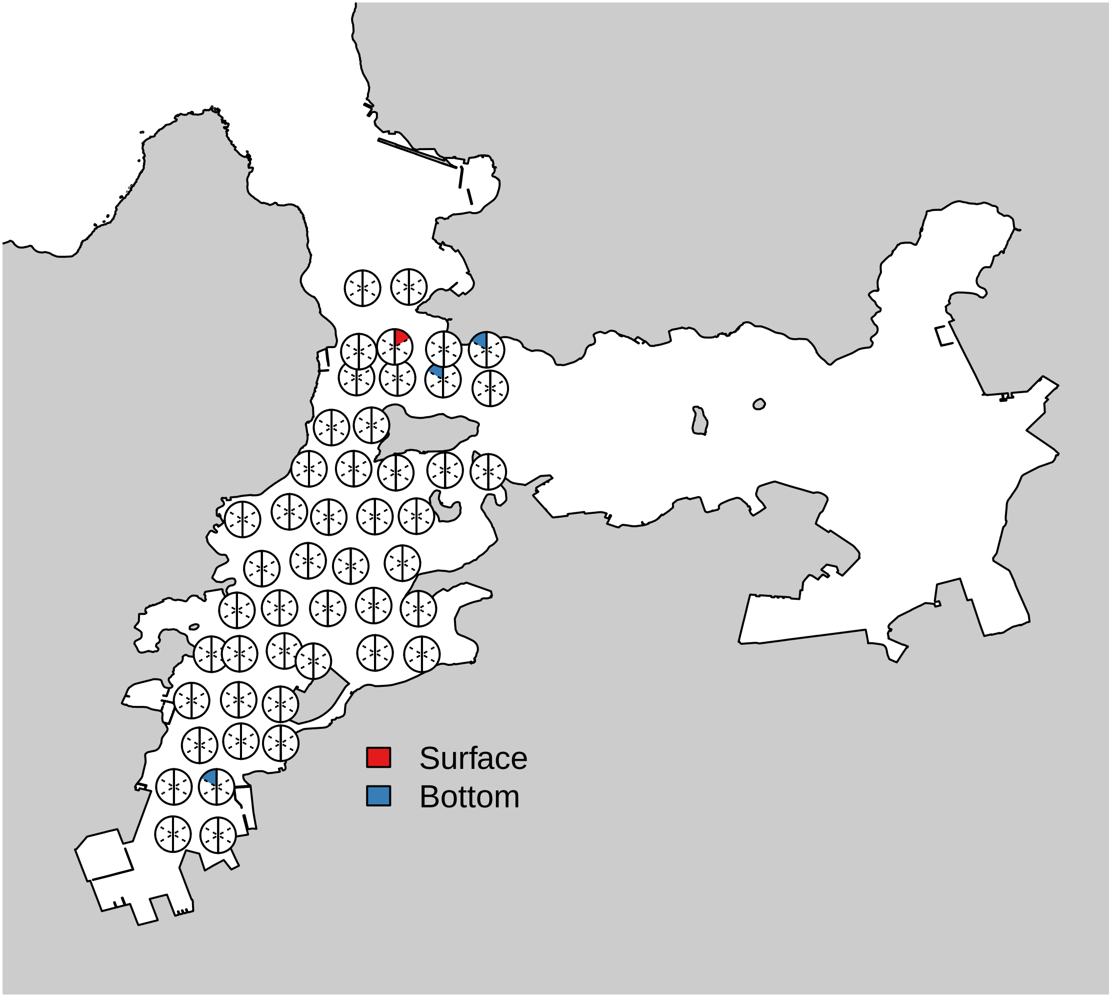

Fig.S1_Helichoeres_poecilopterus

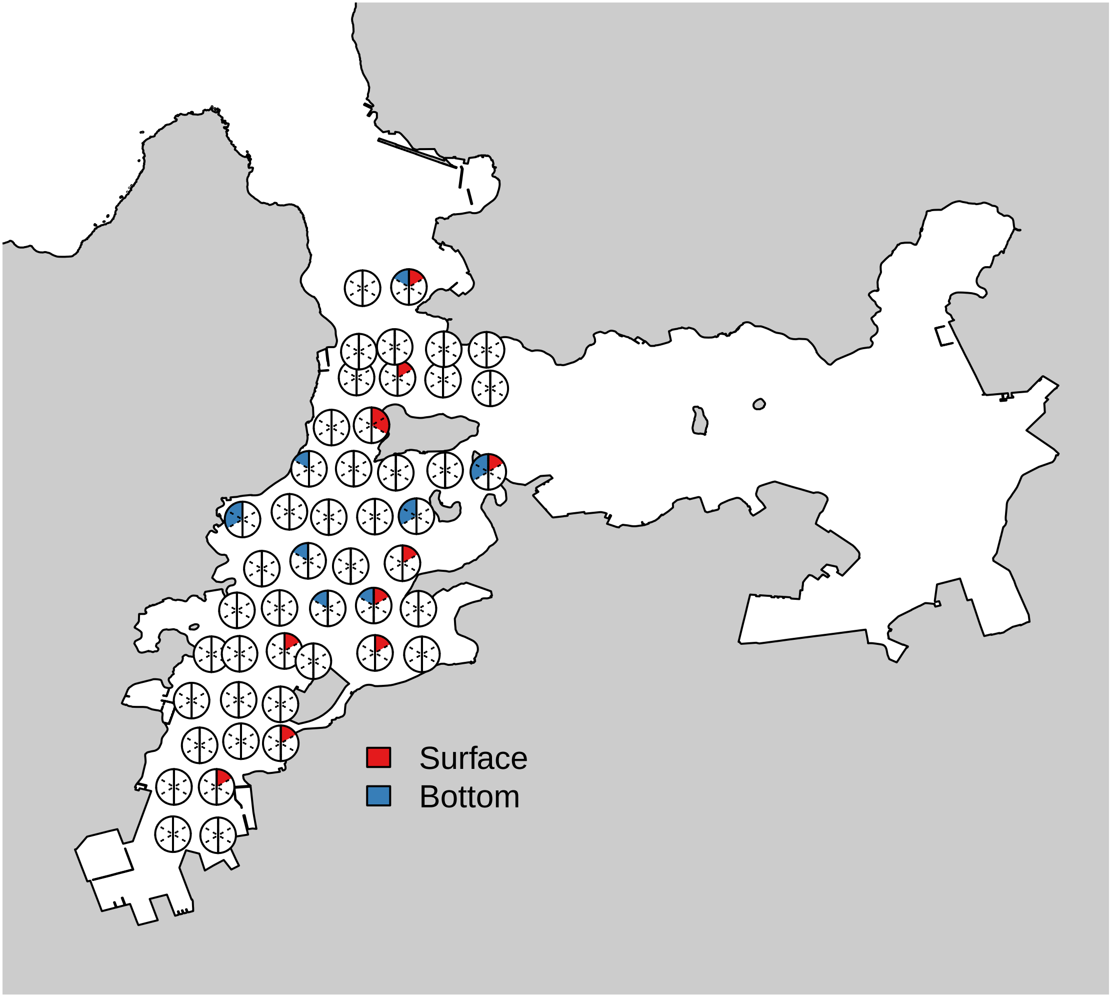

Fig.S1_Hemibarbus_longirostris

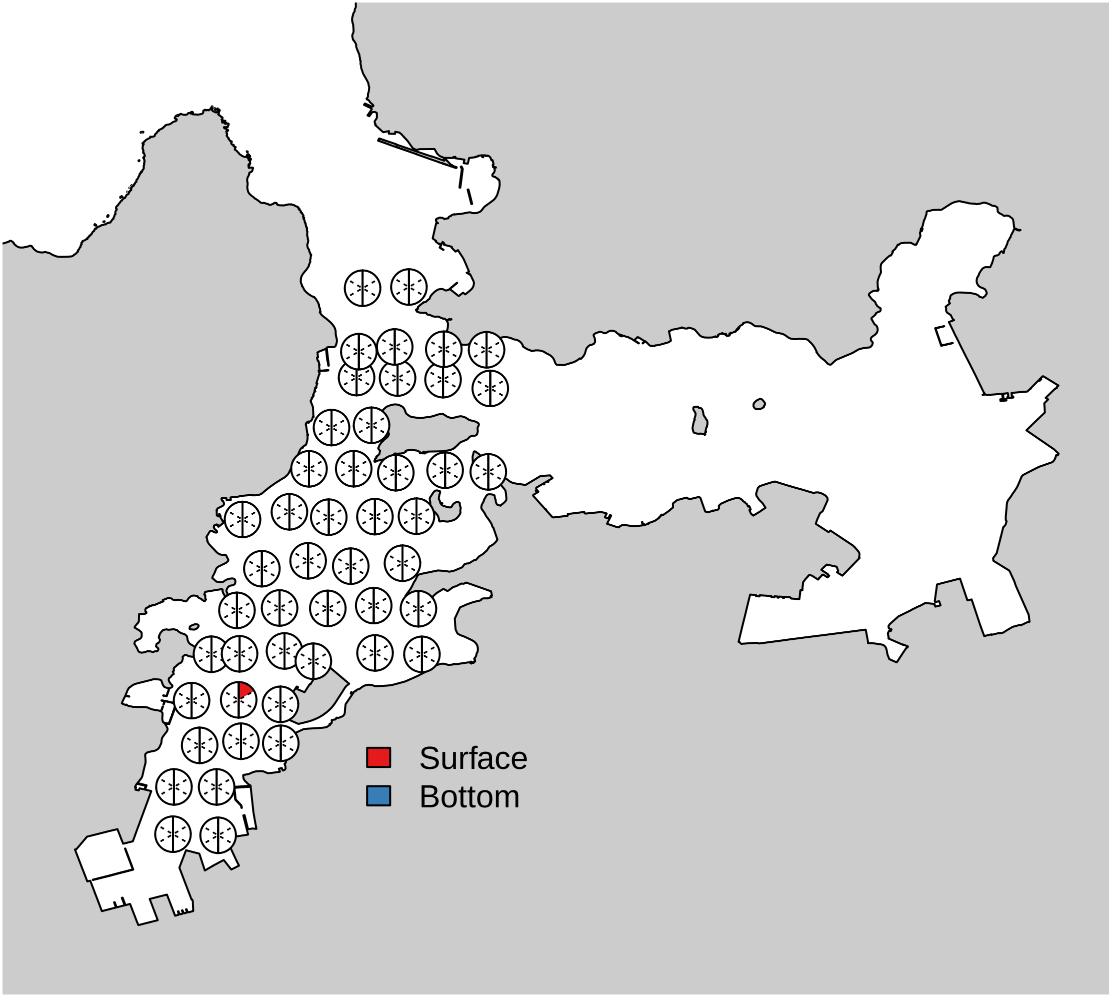

Fig.S1_Hexagrammos_otakii

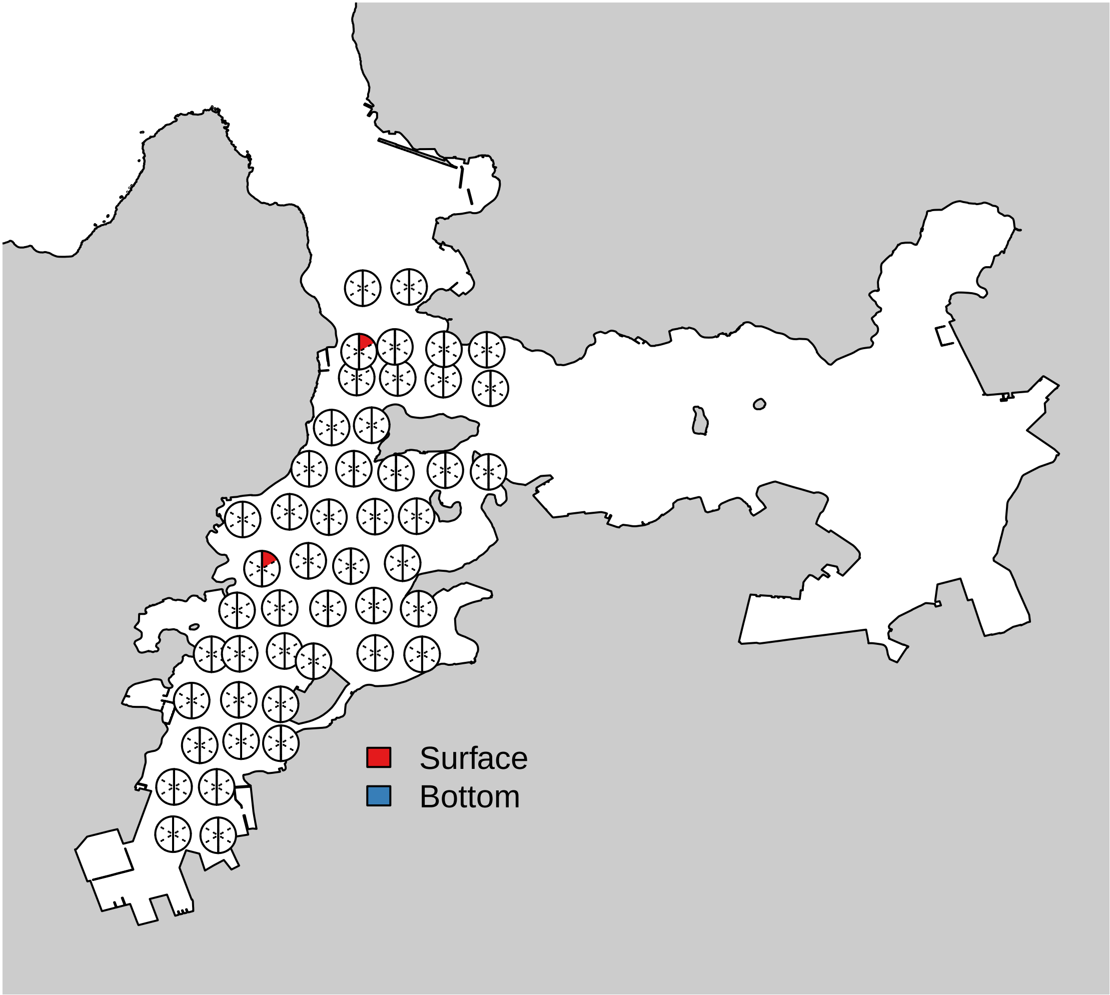

Fig.S1_Hexagrammos_sp.

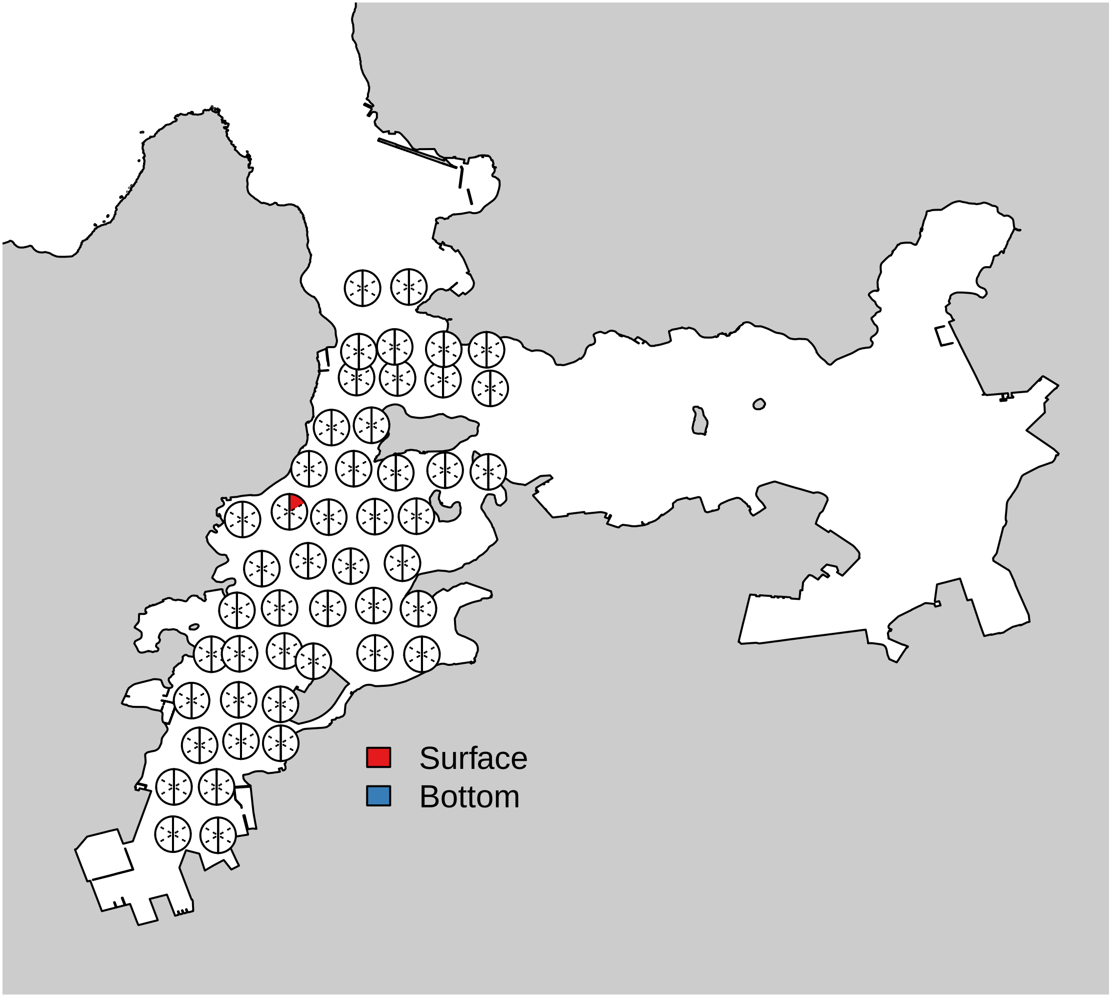

Fig.S1_Hippoglossoides_sp.

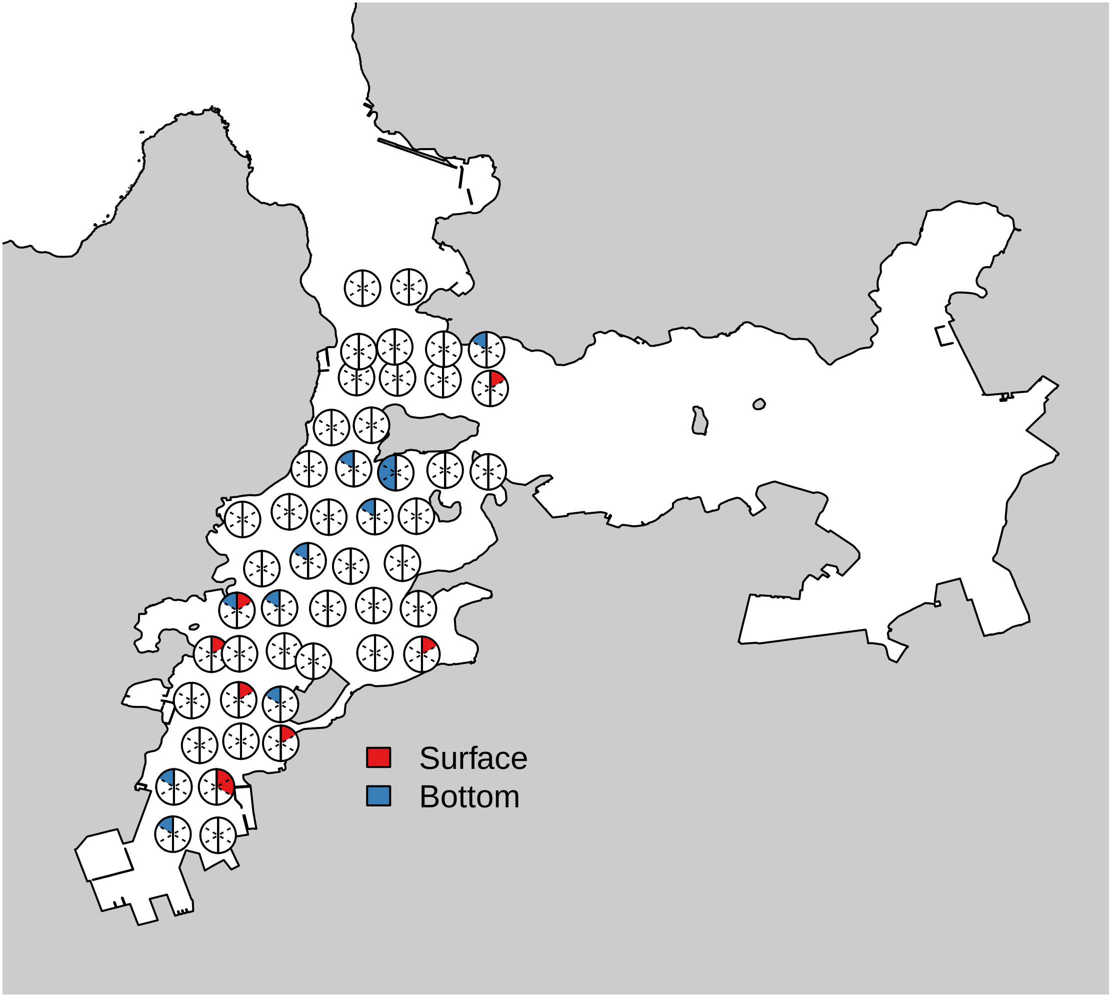

Fig.S1_Hyperoglyphe_japonica

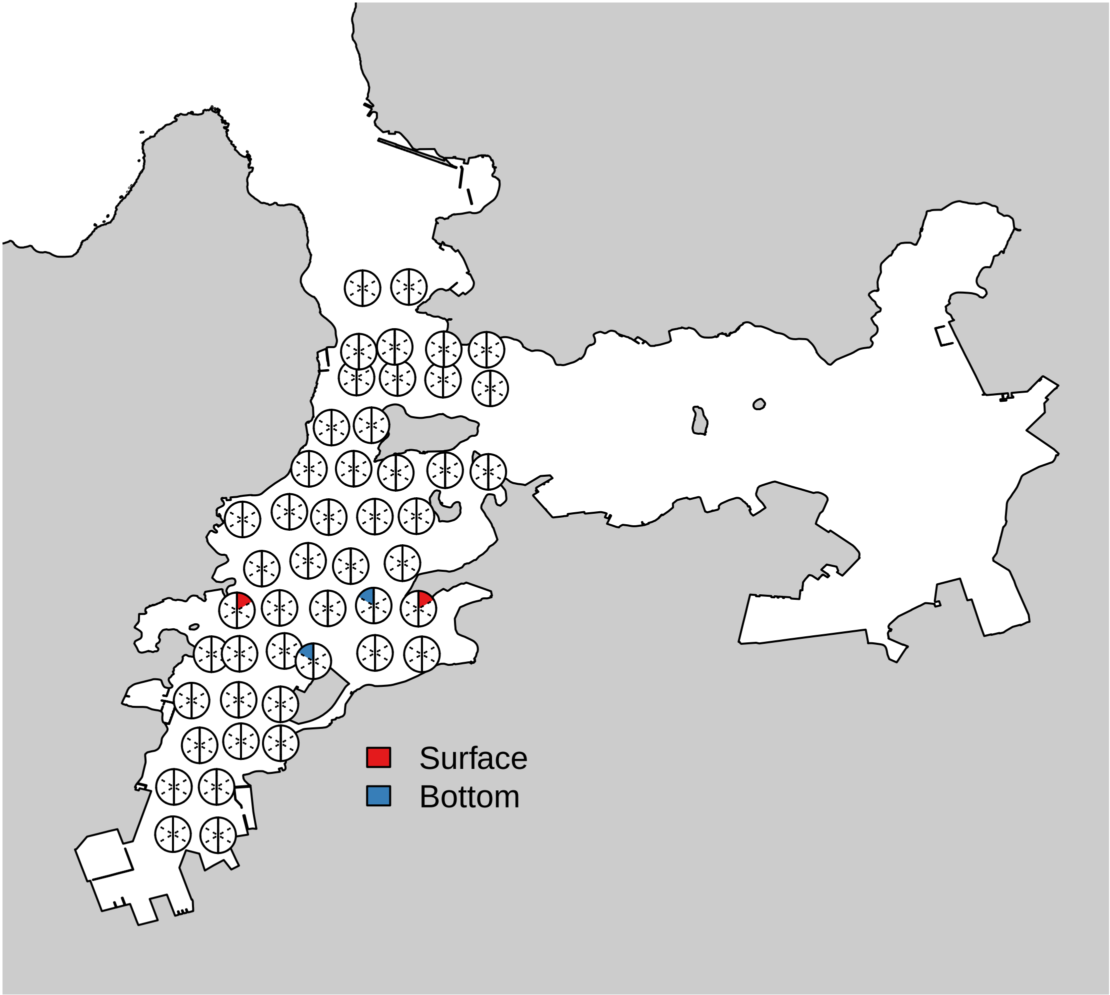

Fig.S1_Hyporhamphus_sajori

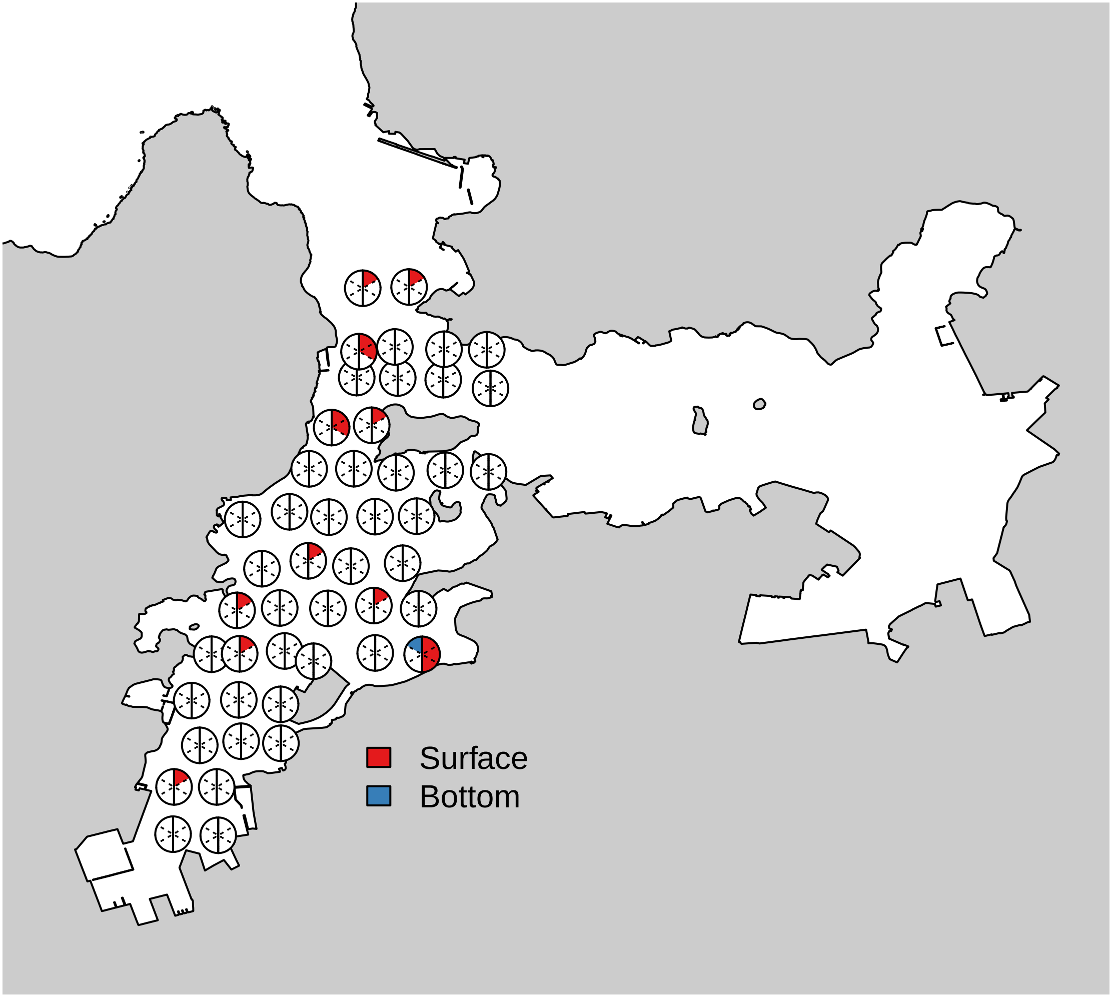

Fig.S1_Istigobius_campbelli

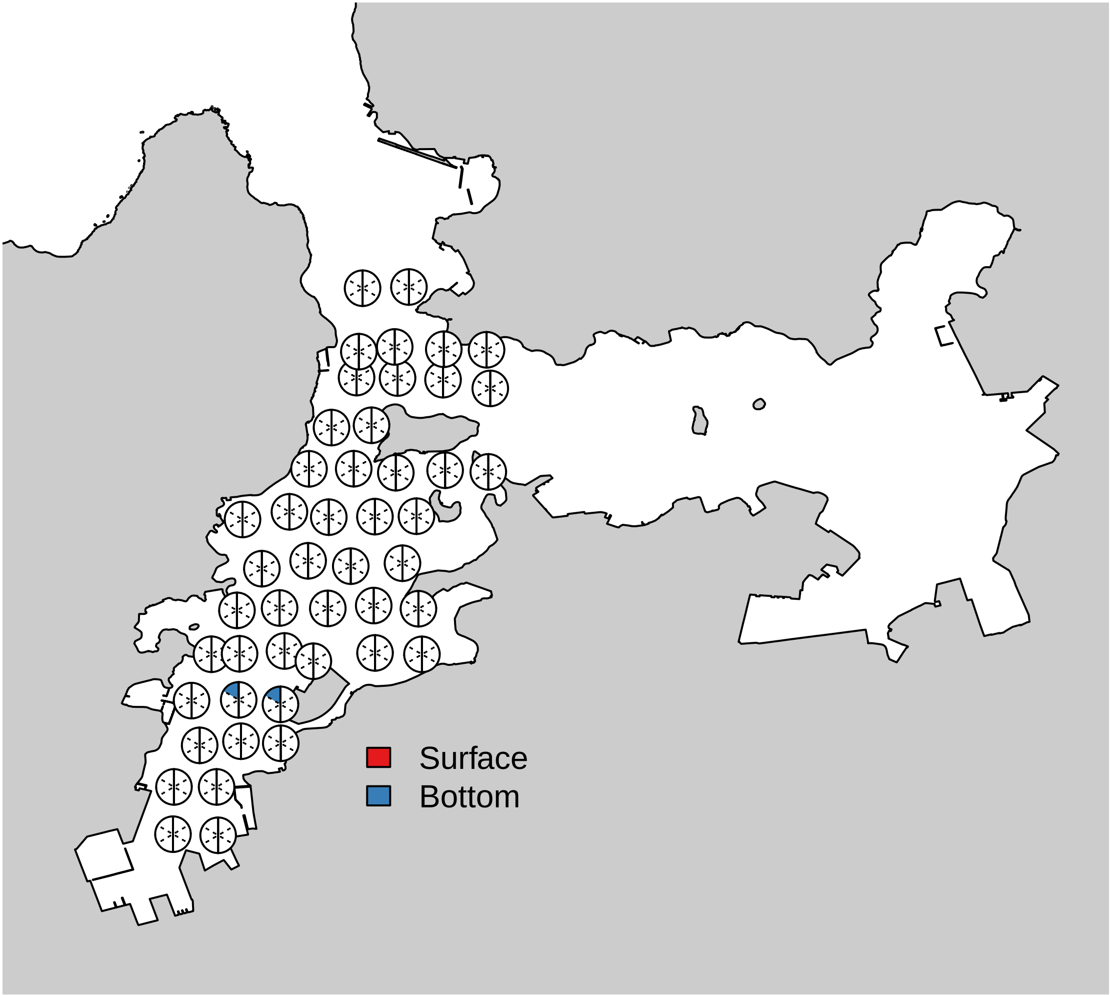

Fig.S1_Jaydia_lineata

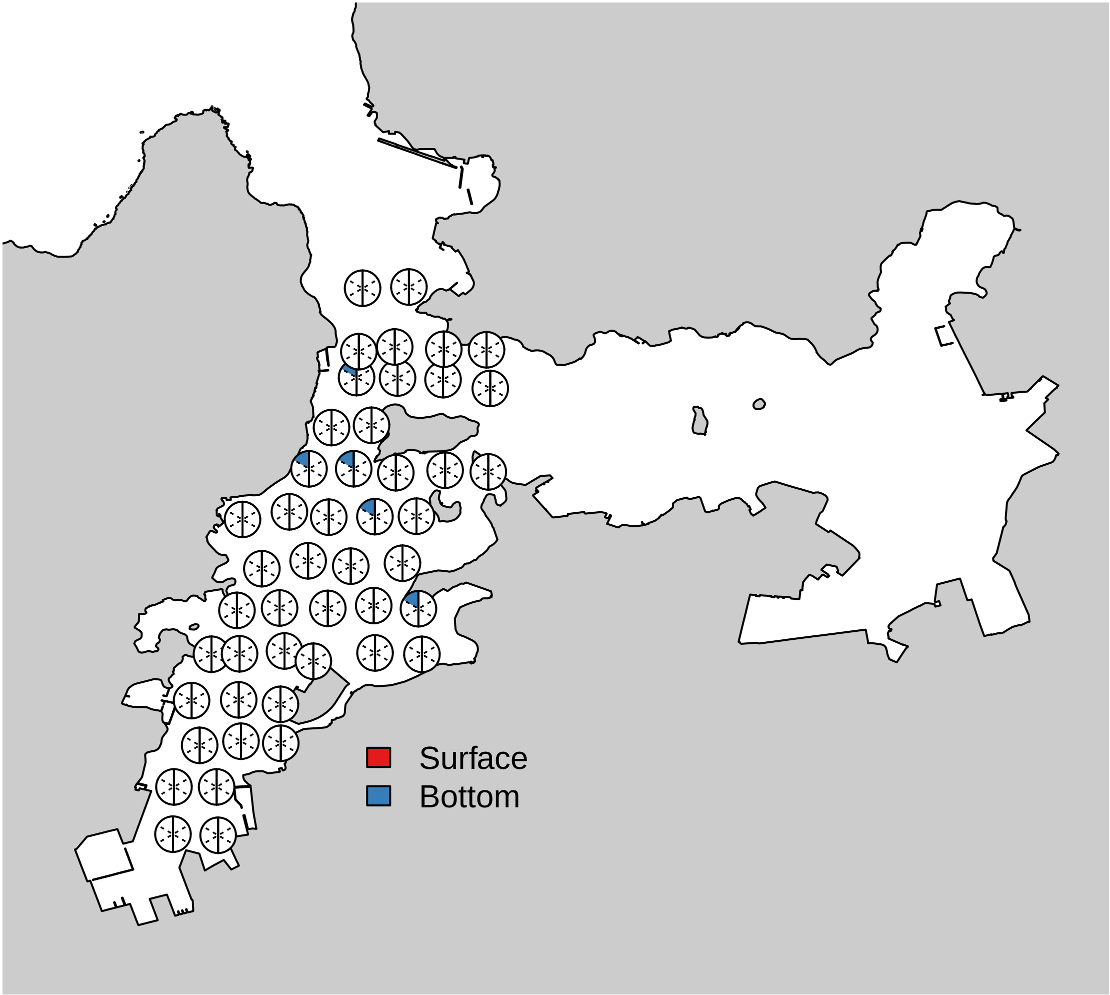

Fig.S1_Konosirus_punctatus

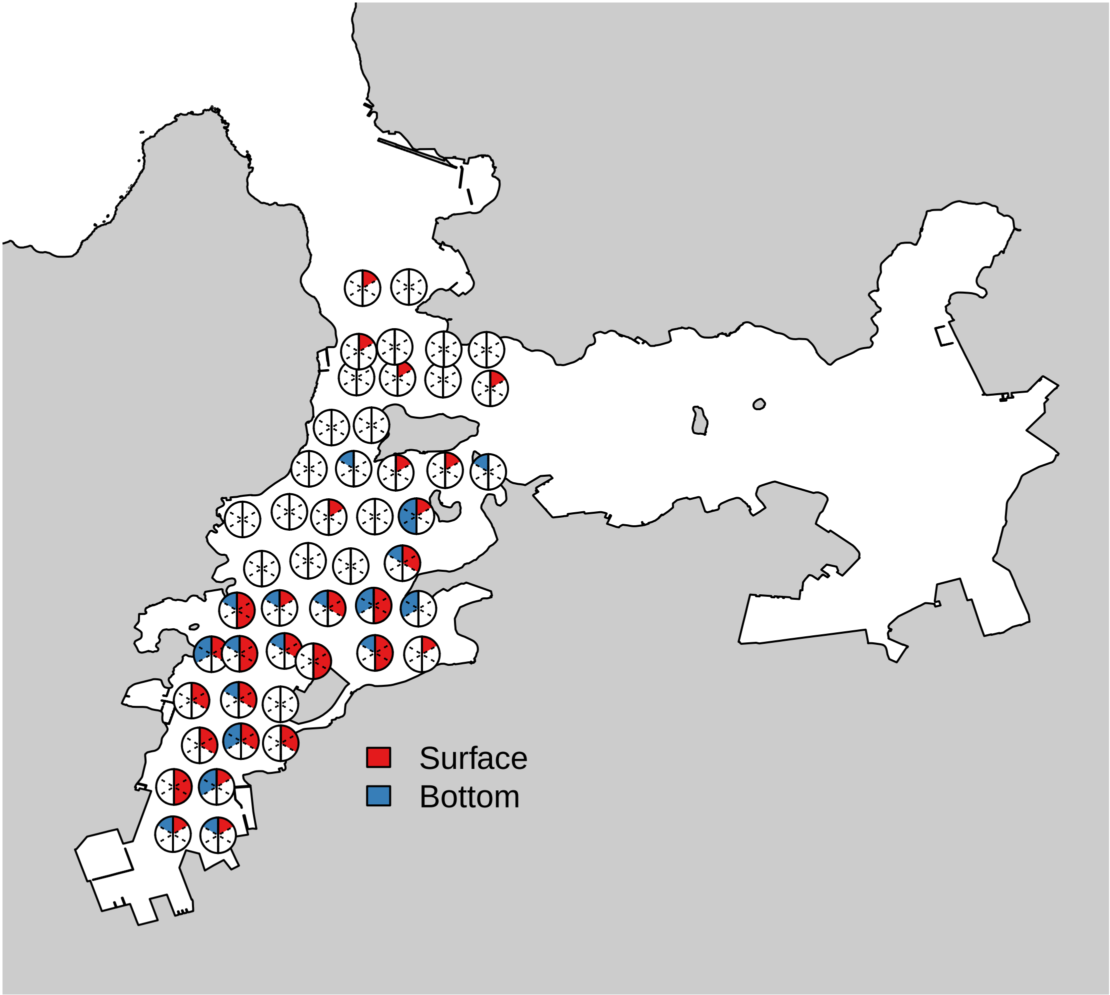

Fig.S1_Lateolabrax_japonicus

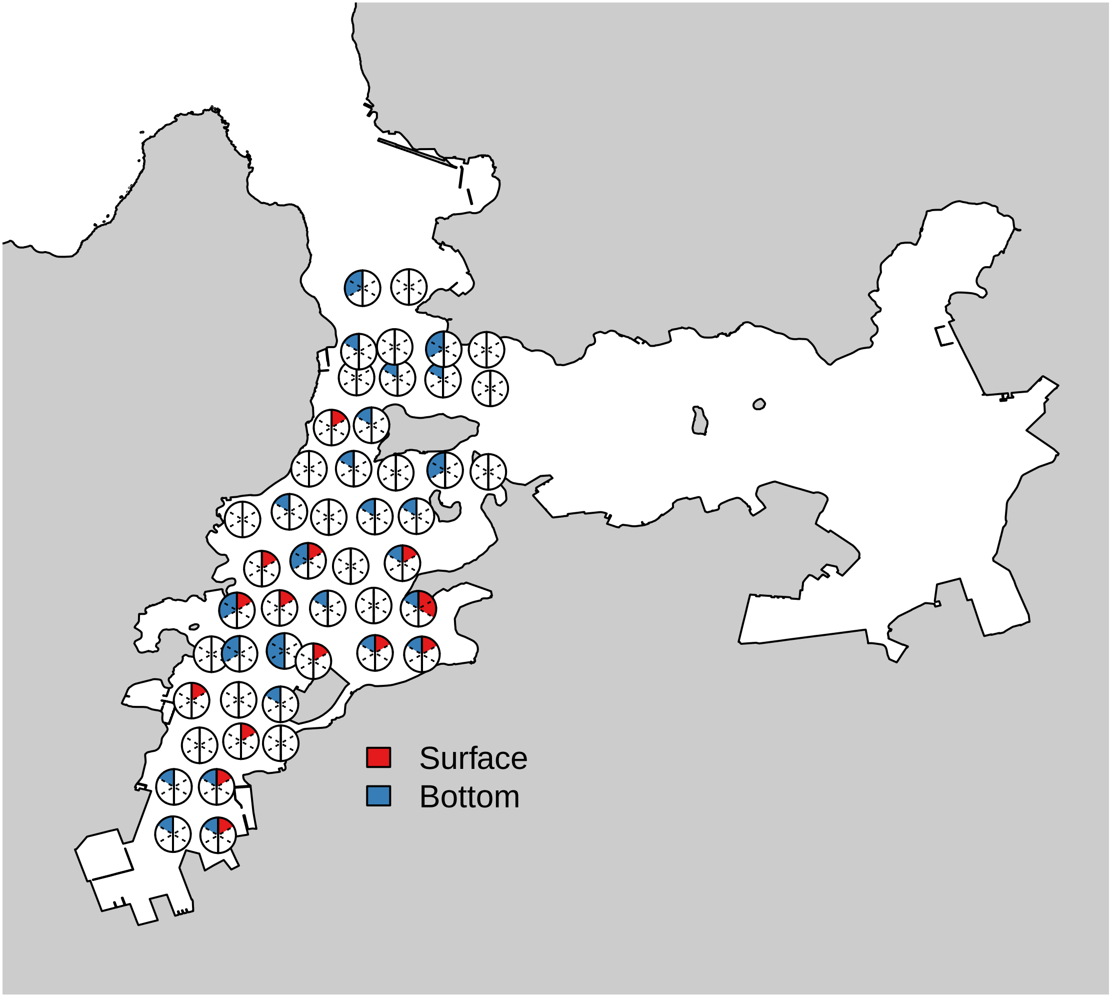

Fig.S1_Lepidotrigla_microptera

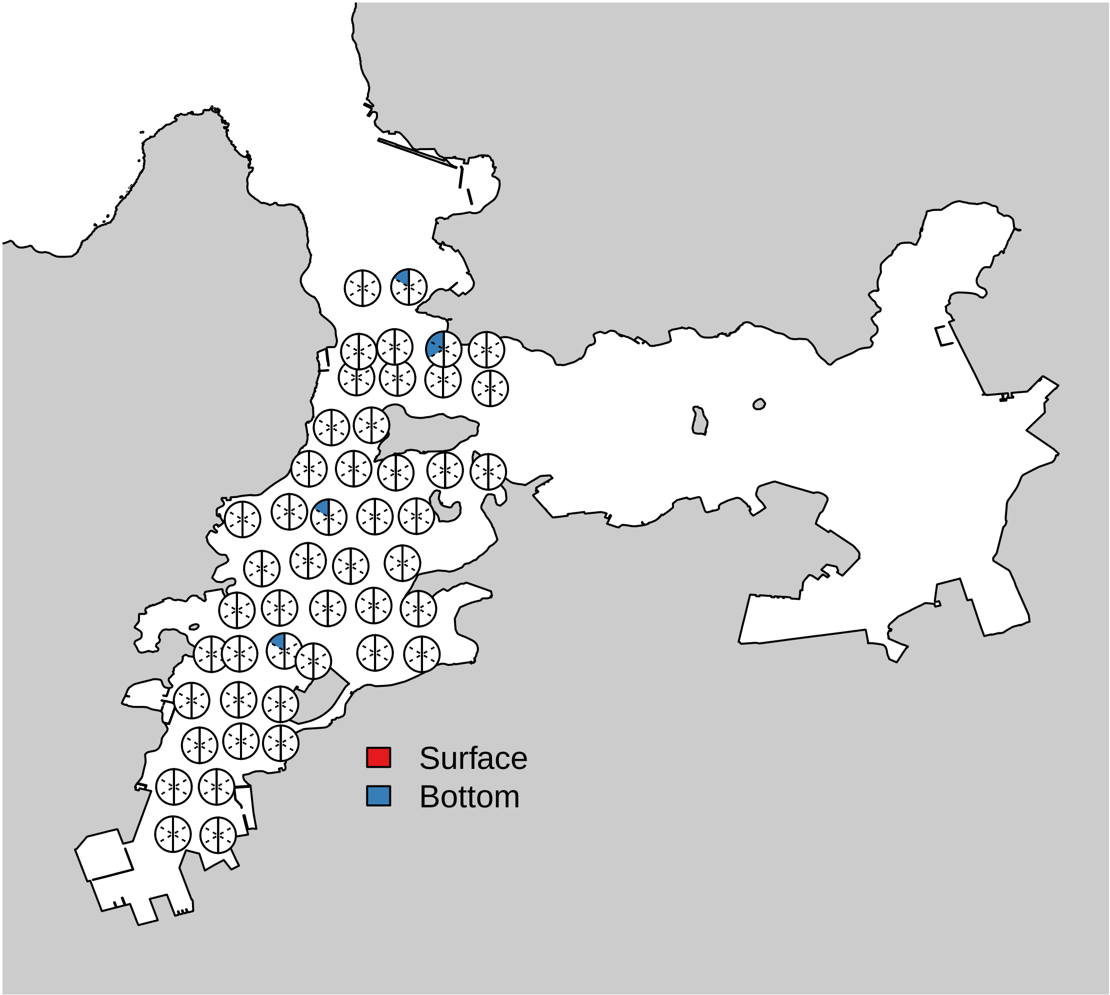

Fig.S1_Leucopsarion_petersii

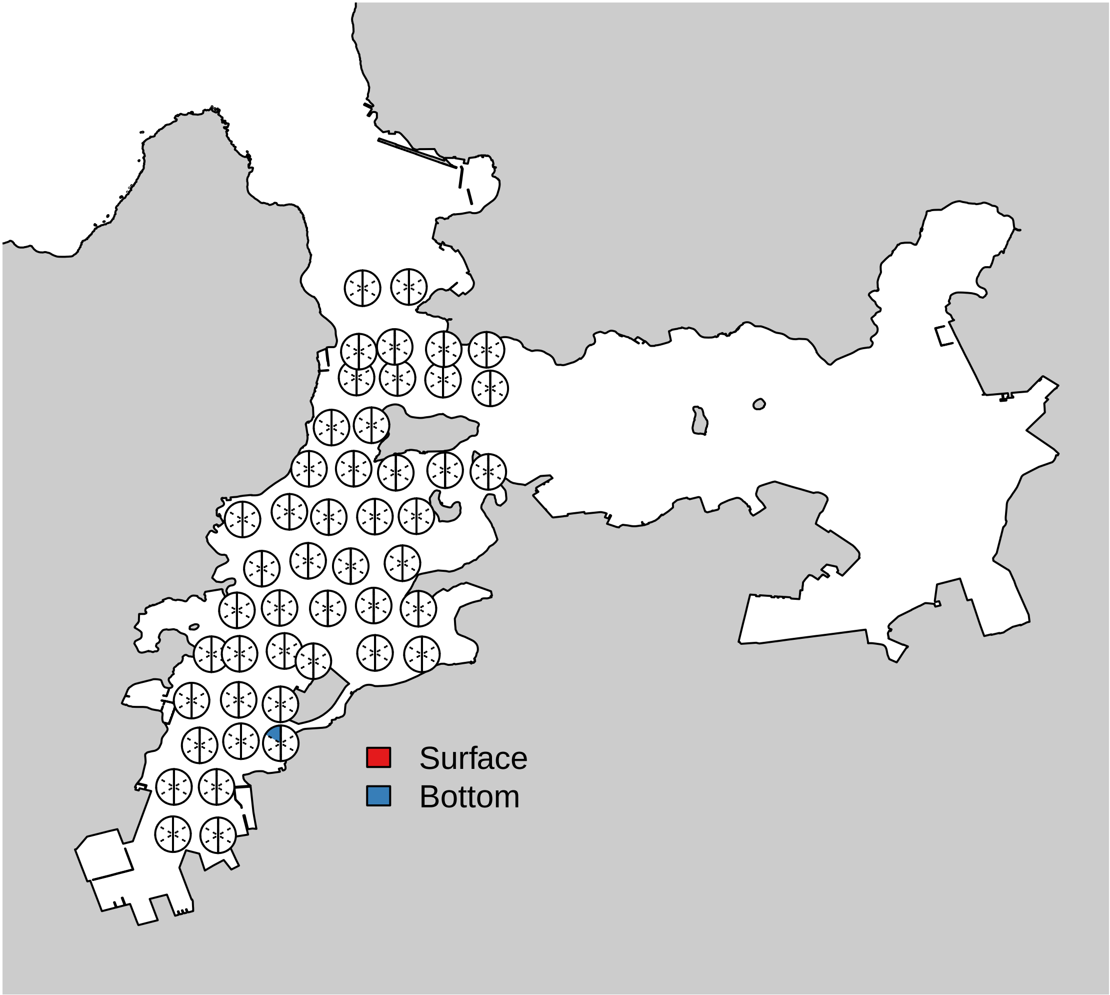

Fig.S1_Liobagrus_reini

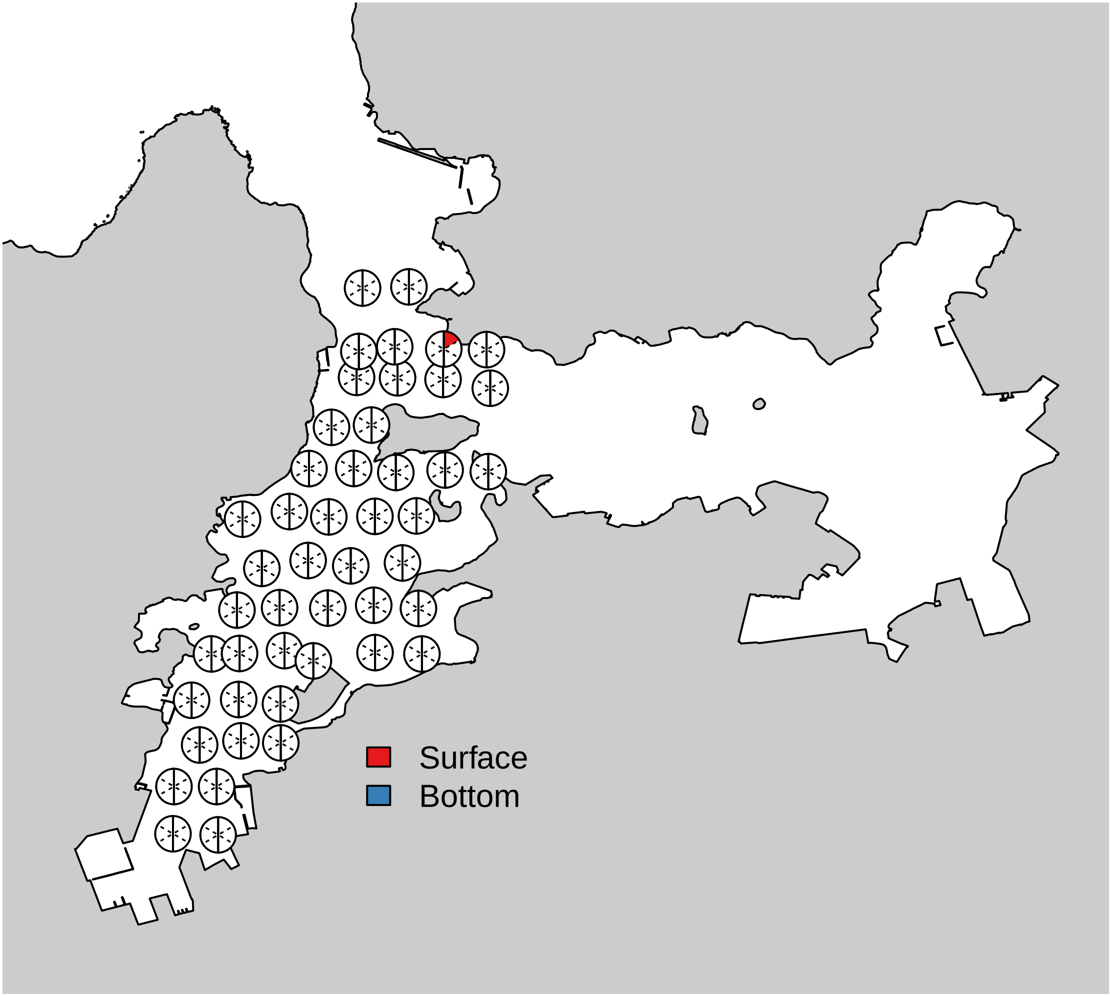

Fig.S1_Liparis_tessellatus

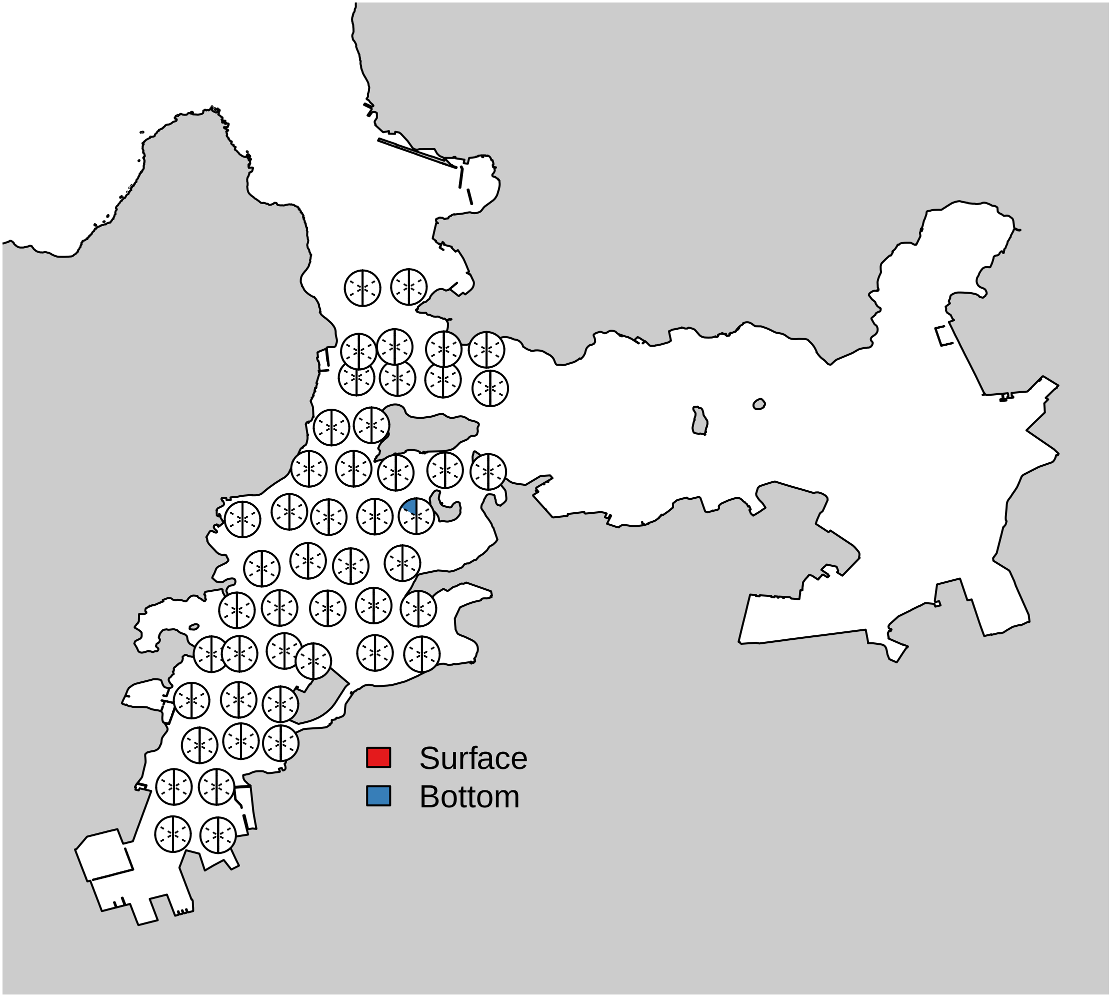

Fig.S1_Lophius_litulon

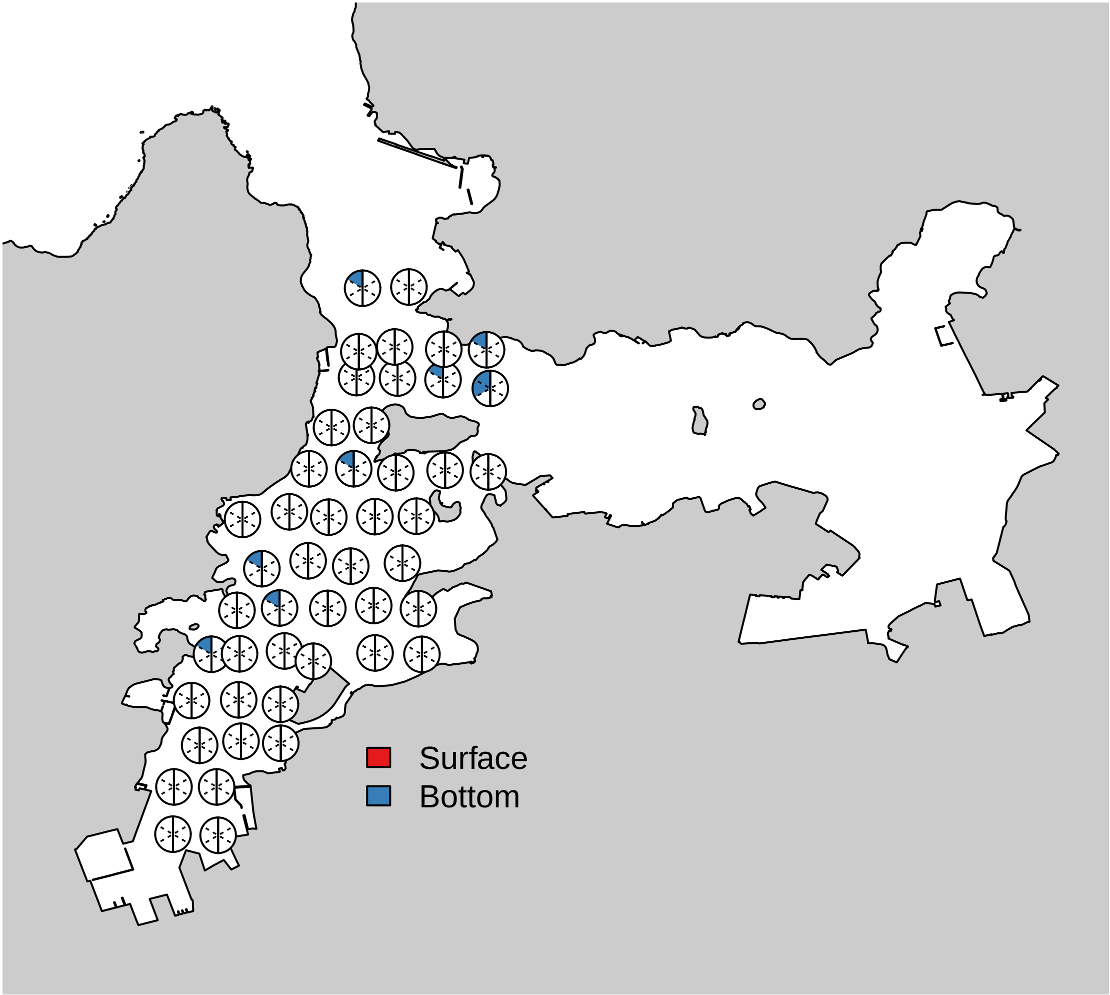

Fig.S1_Misgurnus_anguillicaudatus

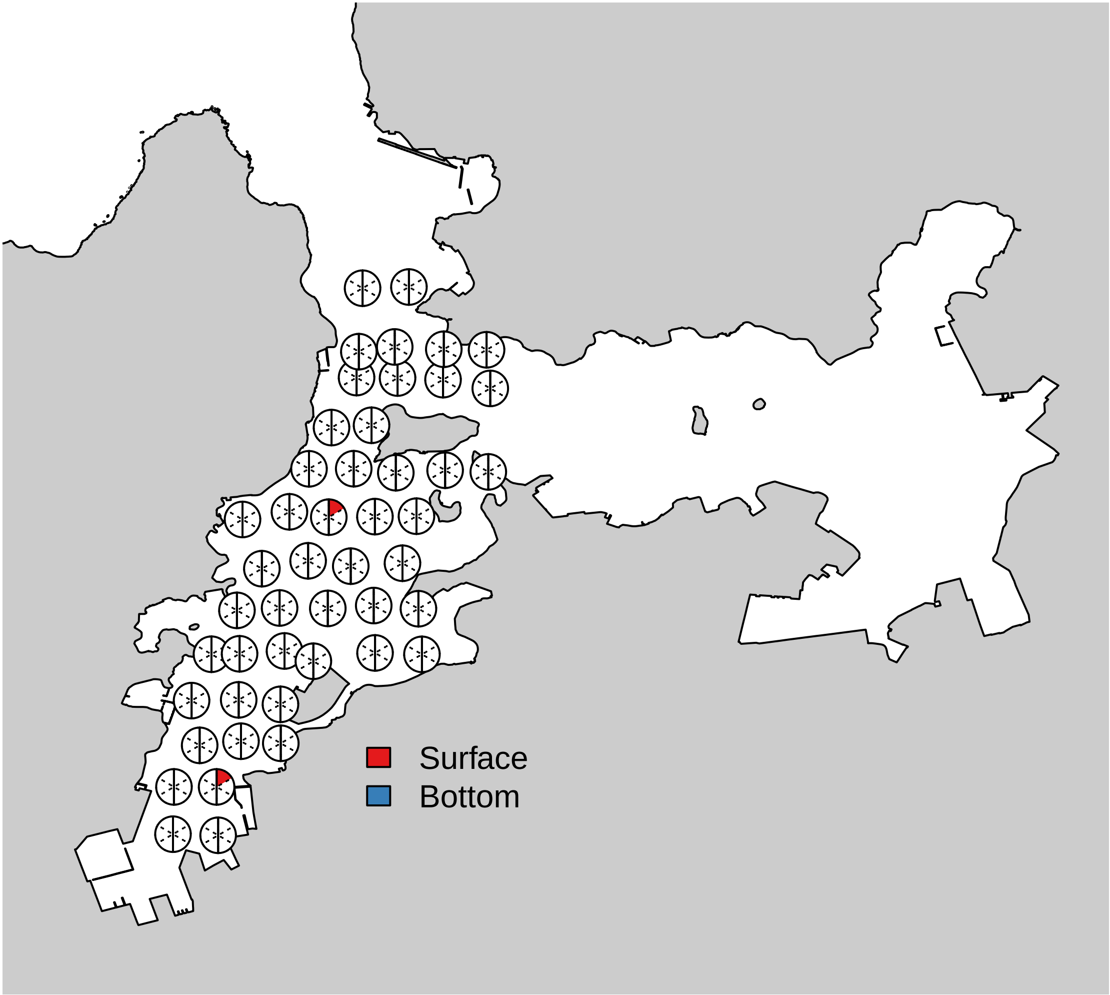

Fig.S1_Mugil_cephalus

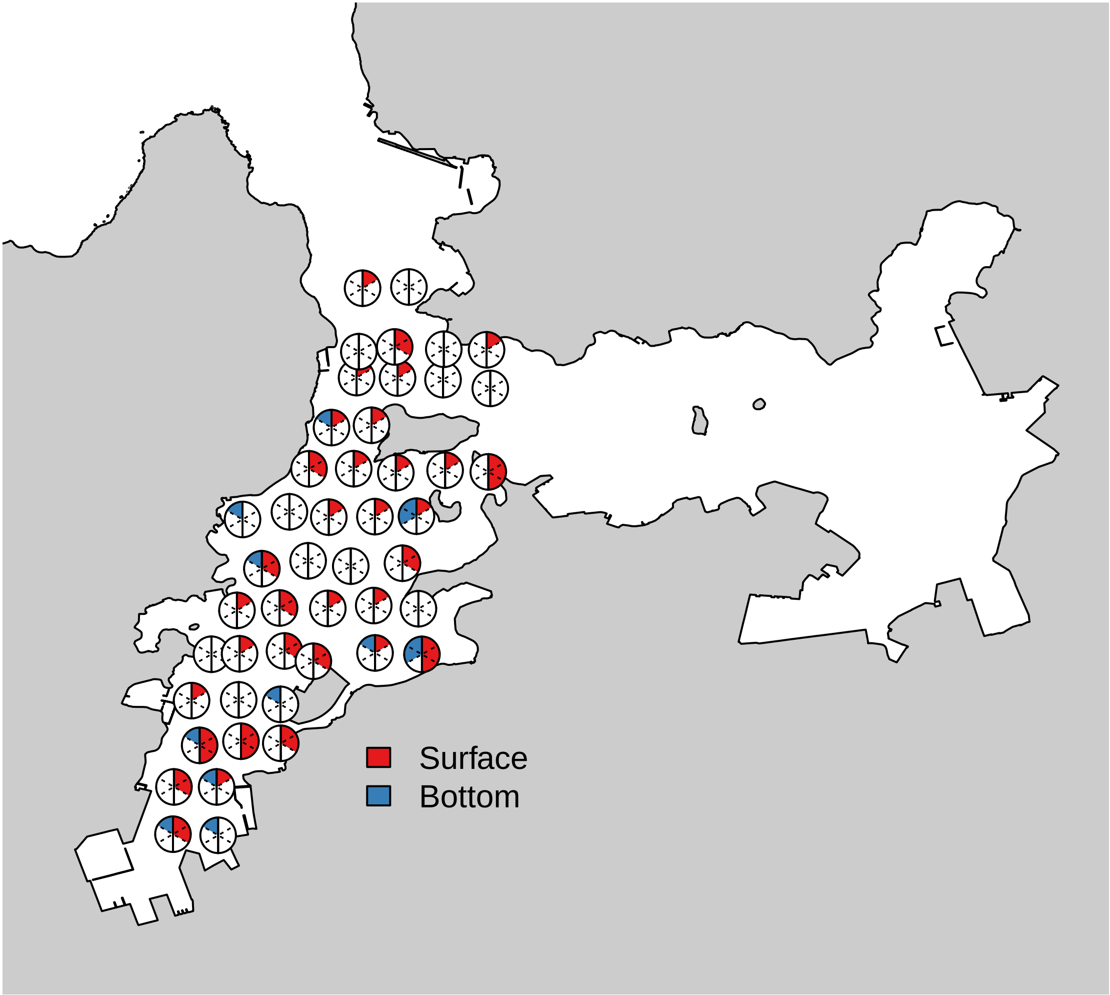

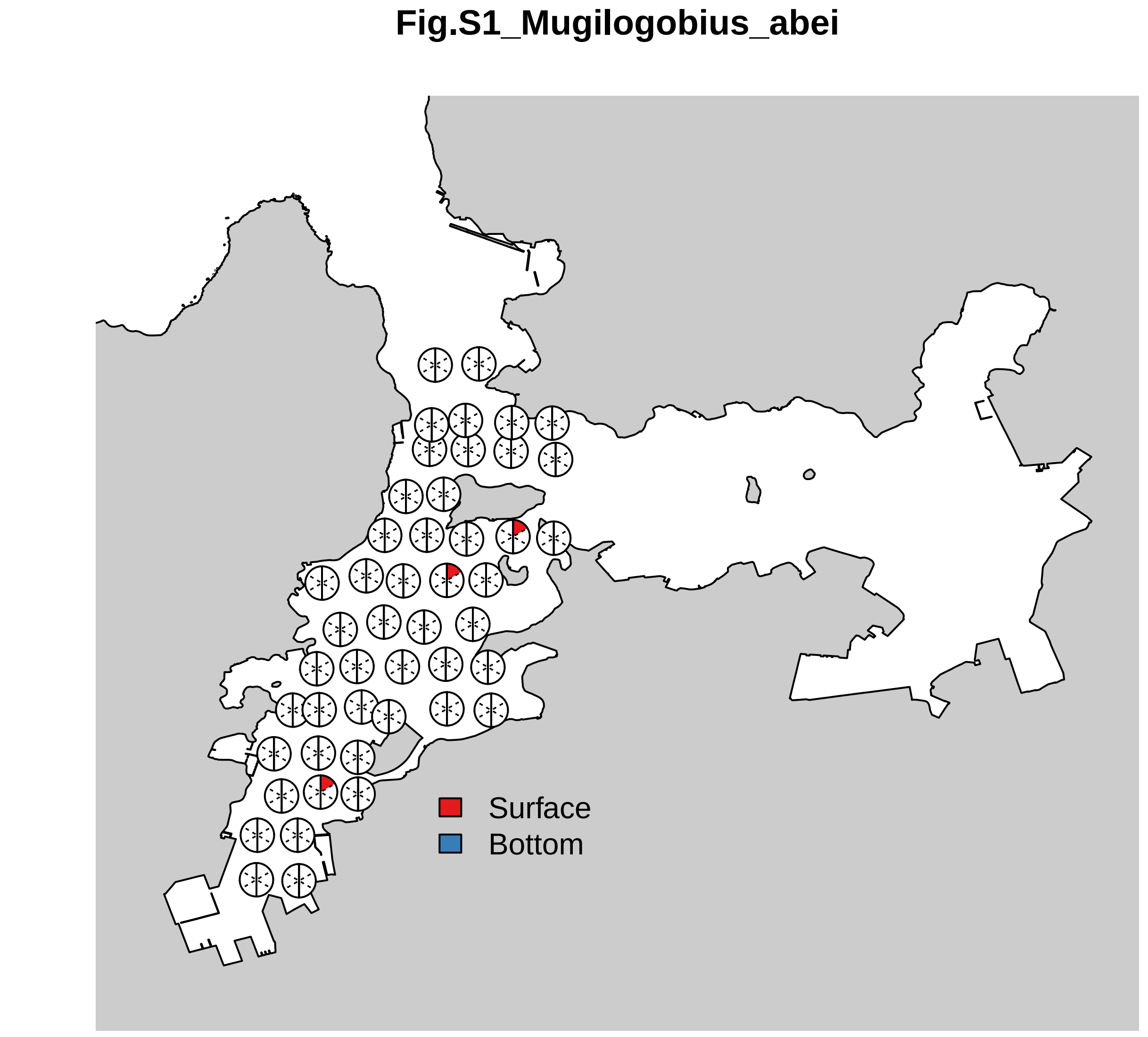
Fig.S1_Mugilogobius_abei
Surface
Bottom

Fig.S1_Muraenesox_cinereus

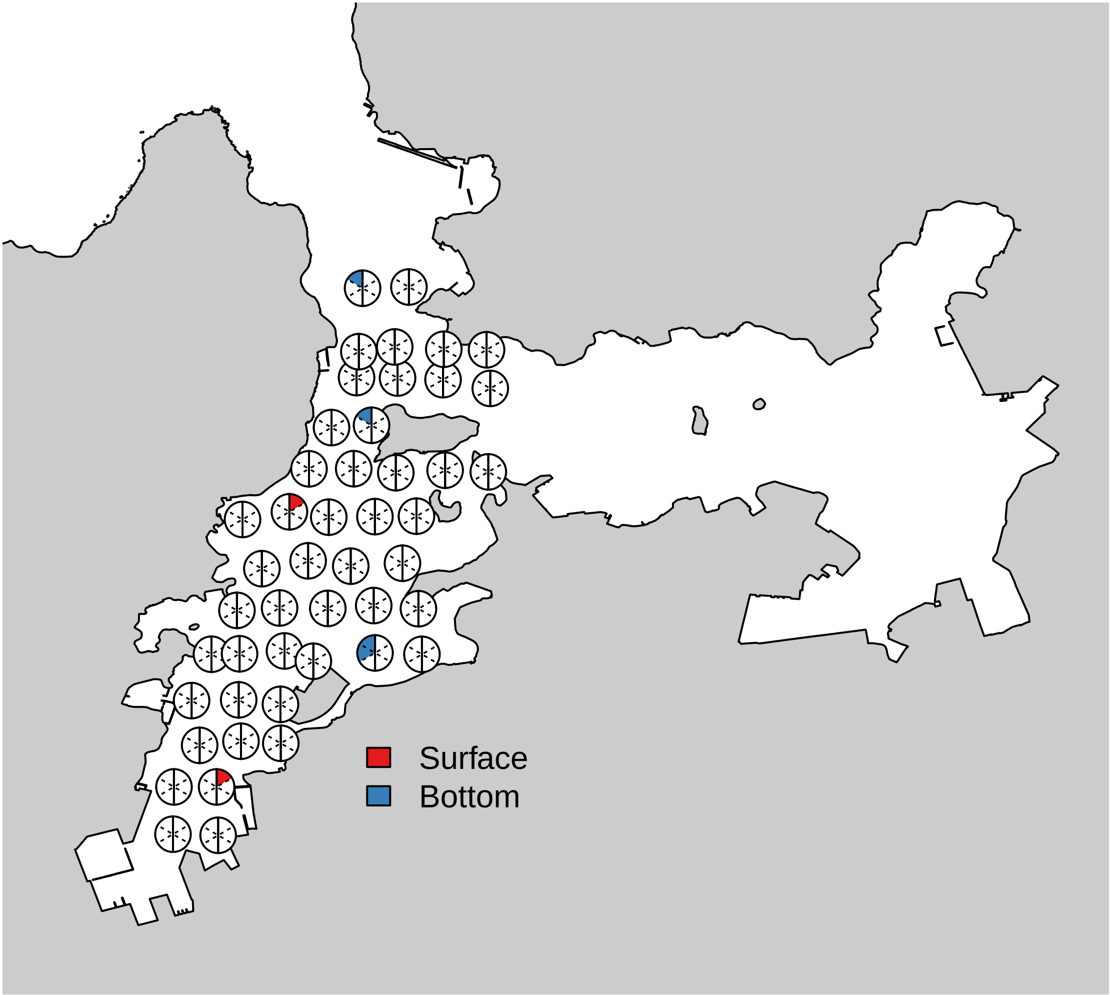

Fig.S1_Myersina_filifer

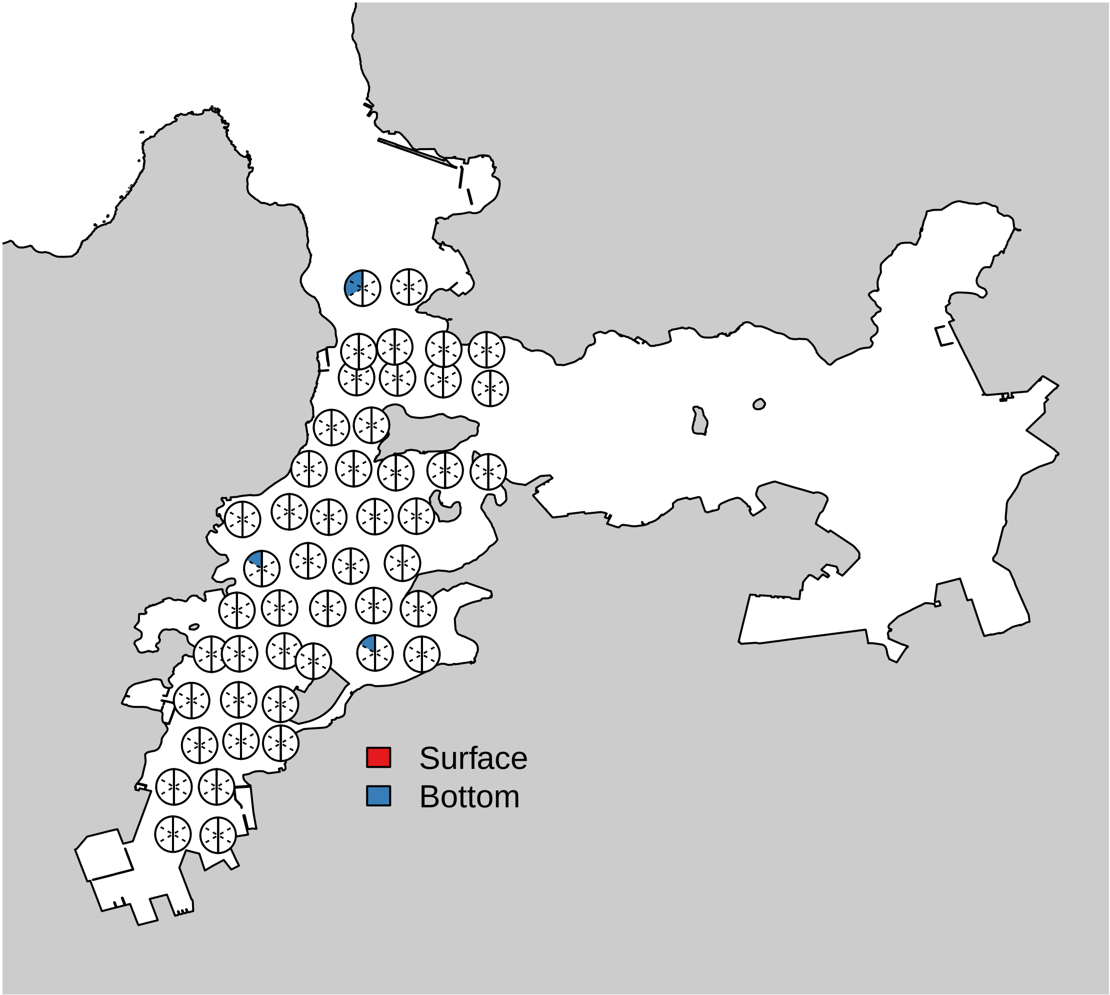

Fig.S1_Nipponocypris_temminckii

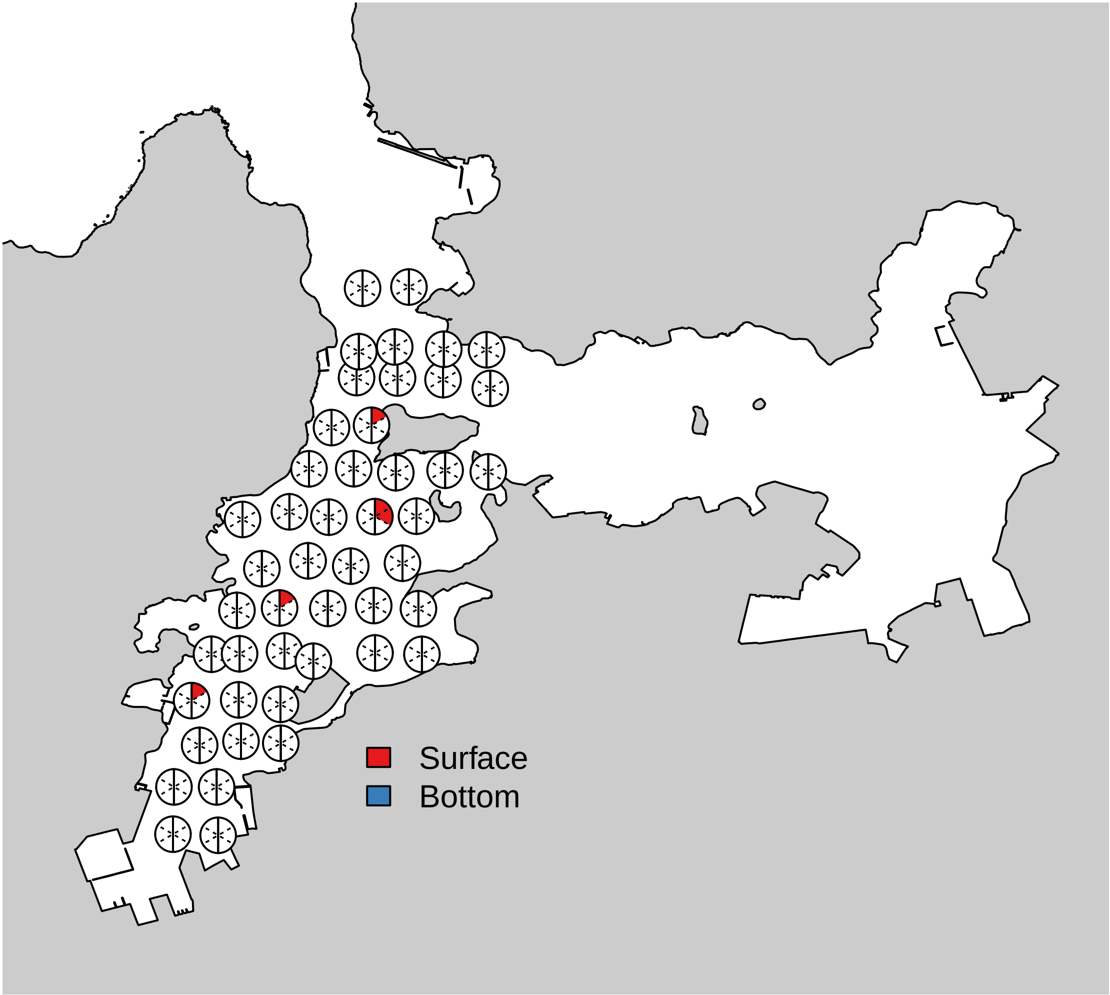

Fig.S1_Nuchequula_nuchalis

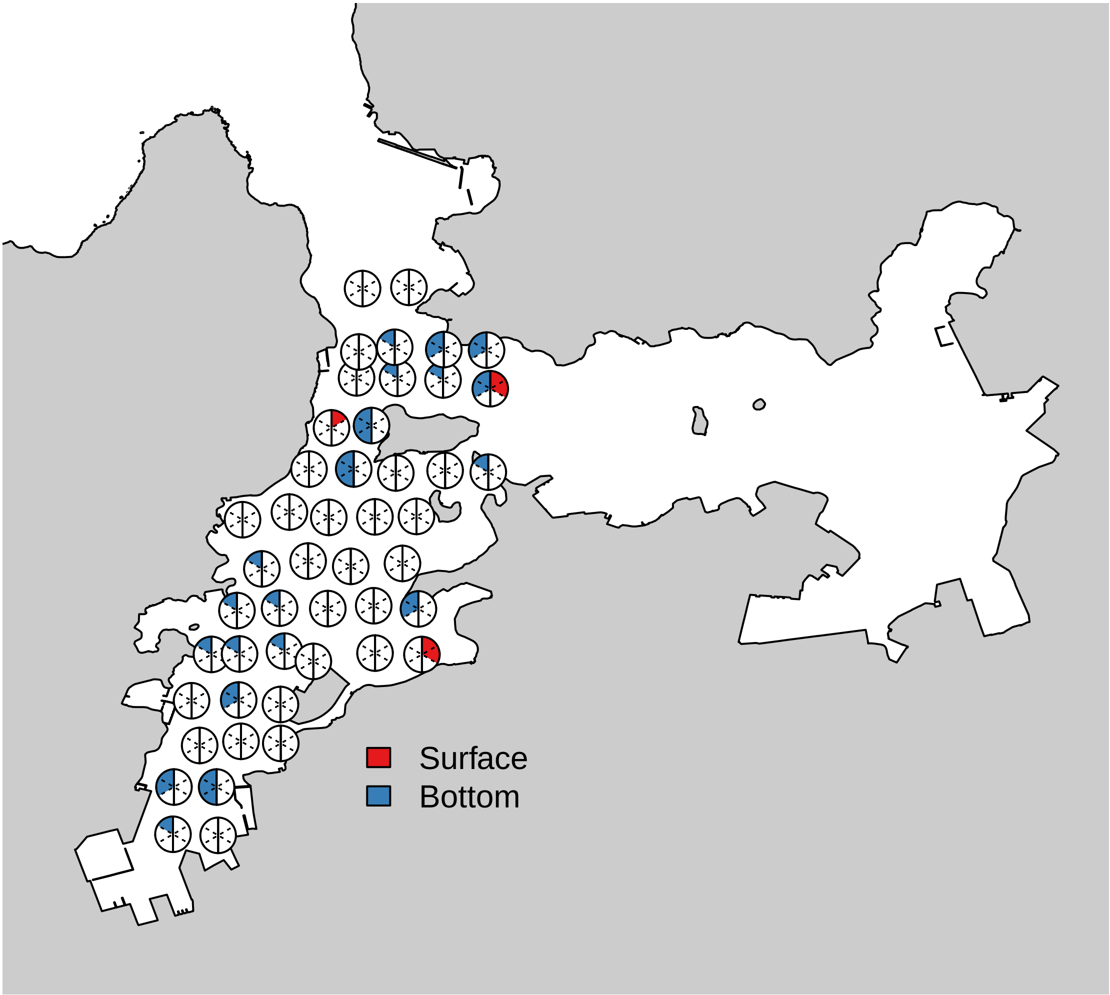

Fig.S1_Odontobutis_obscura

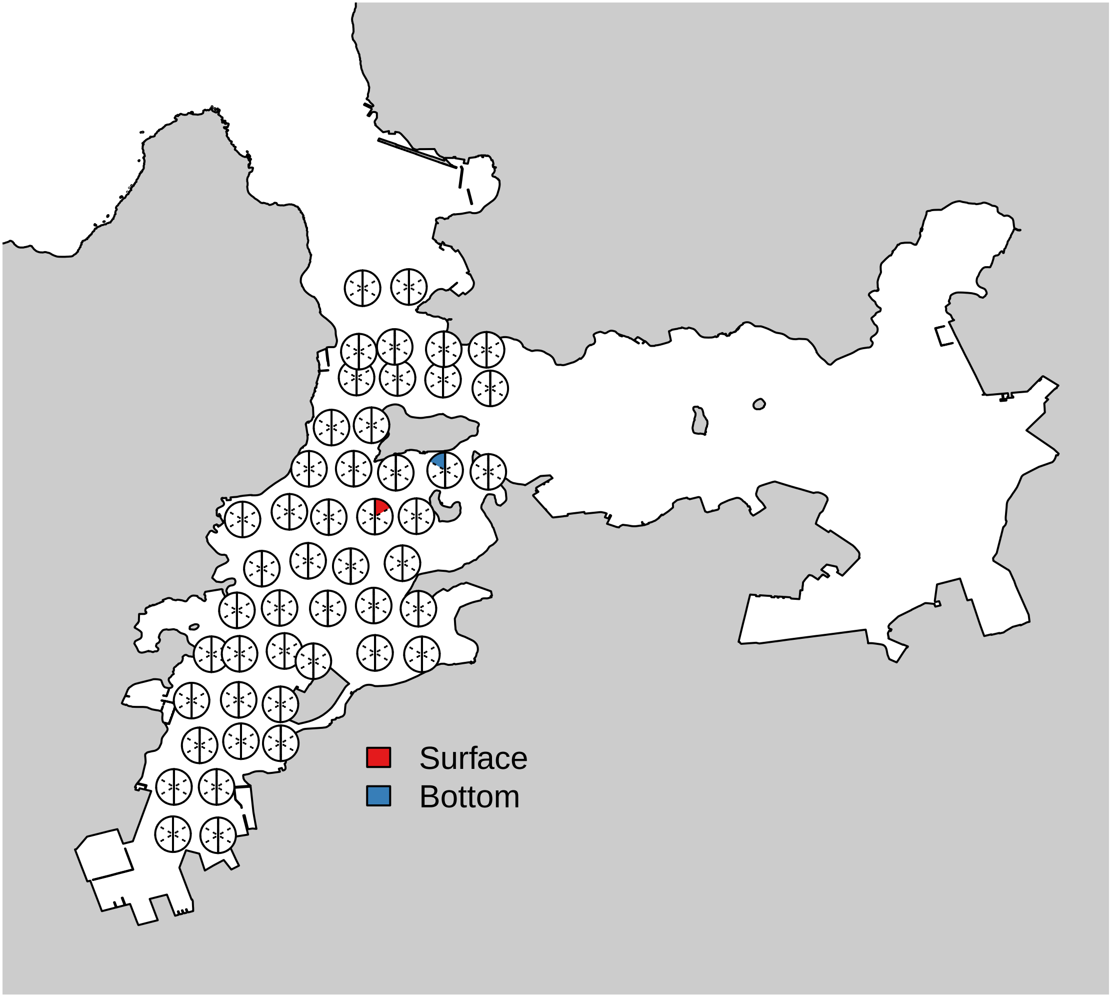

Fig.S1_Omobranchus_elegans

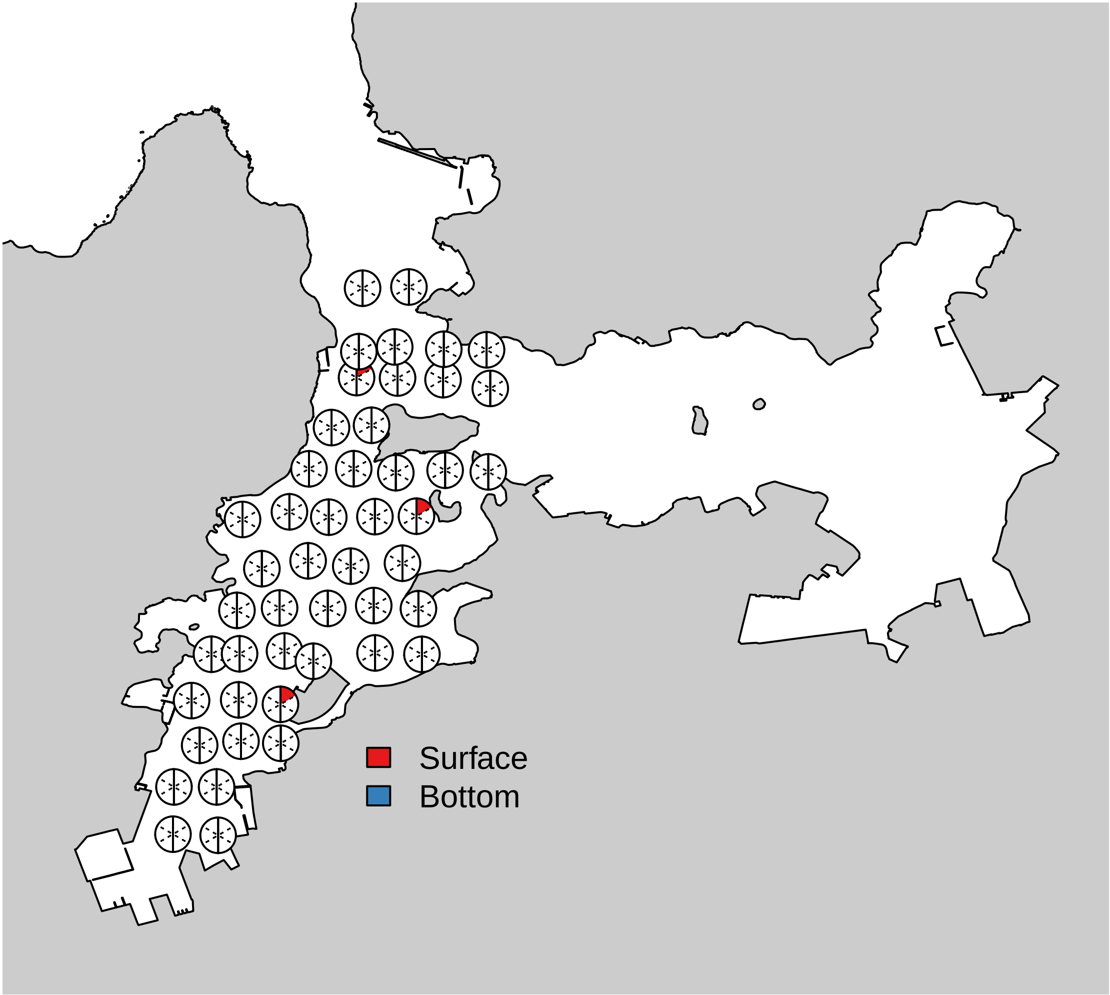

Fig.S1_Omobranchus_fasciolatoceps

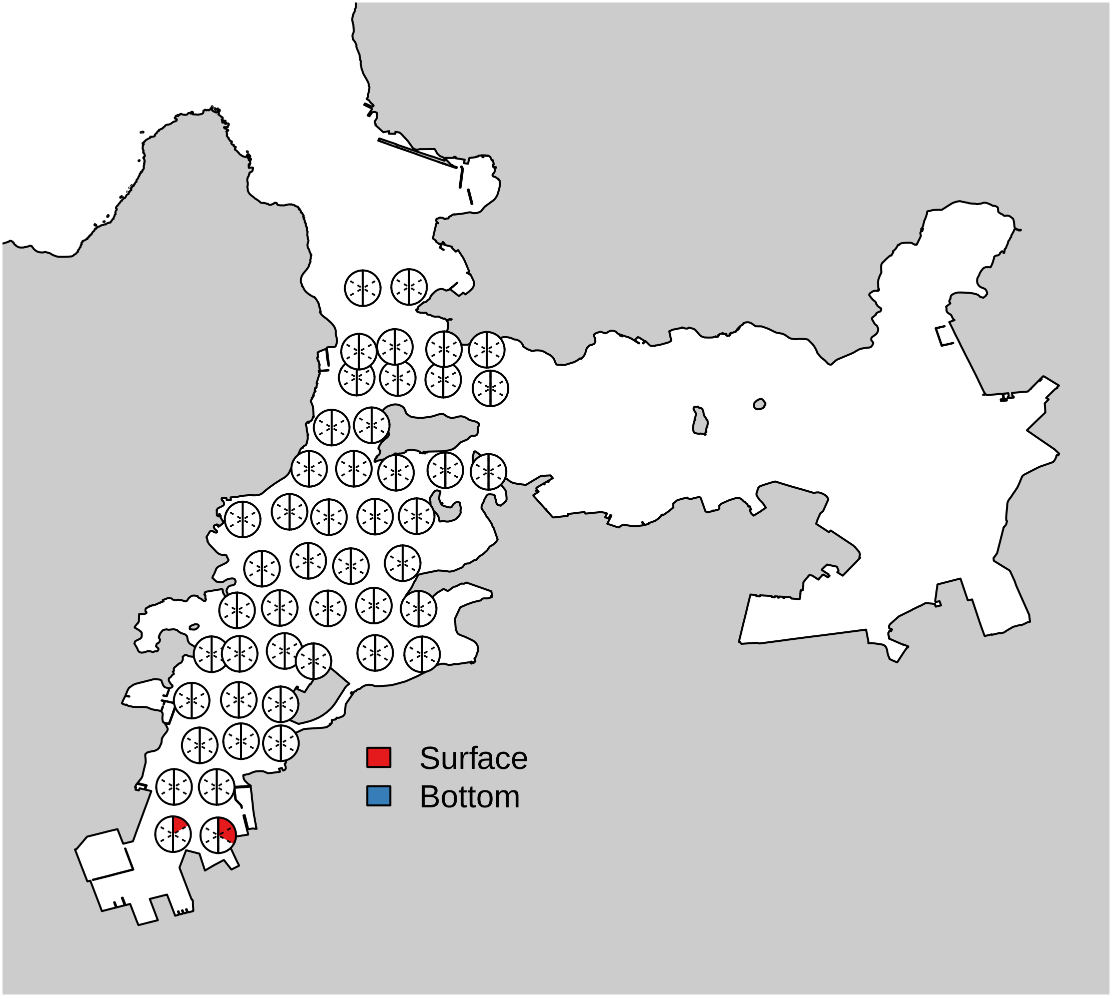

Fig.S1_Omobranchus_punctatus

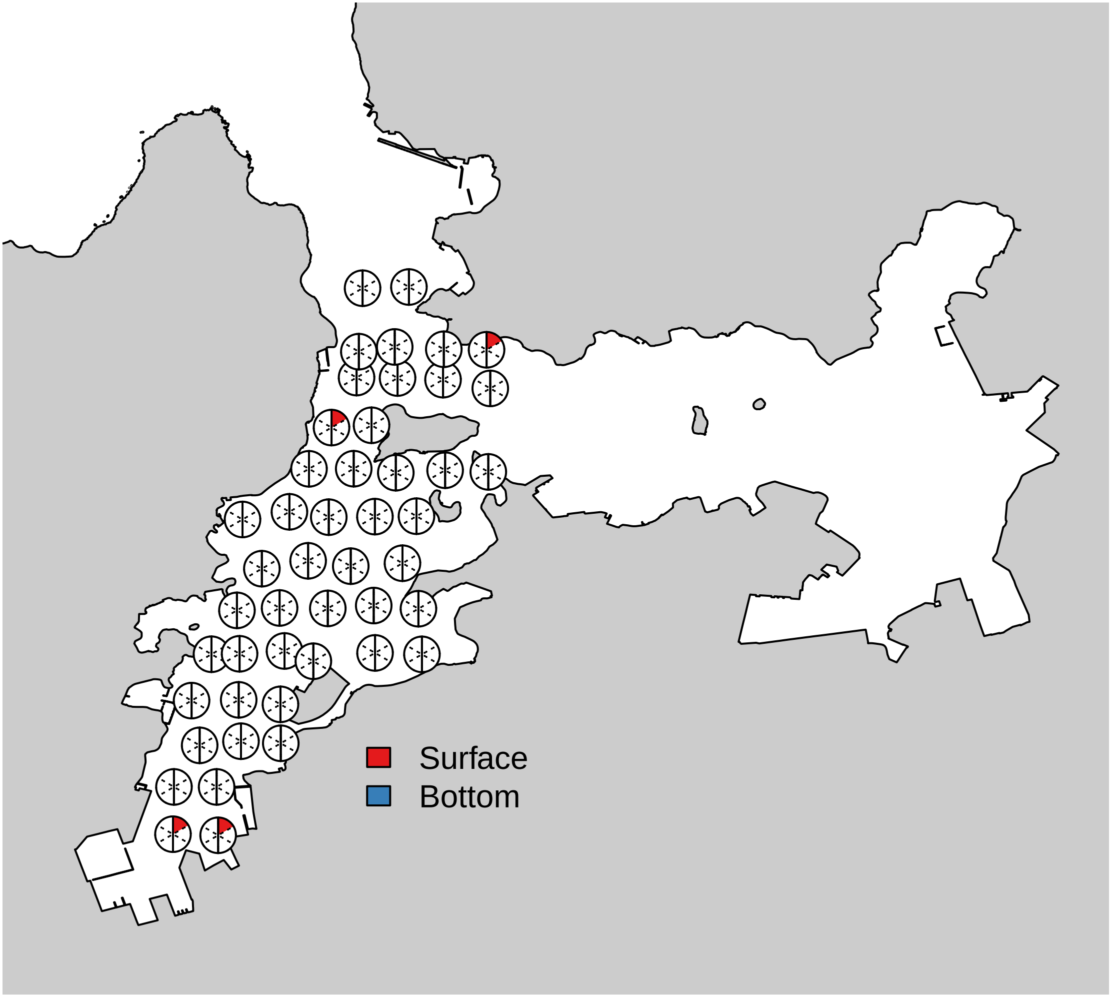

Fig.S1_Oncorhynchus_nerka

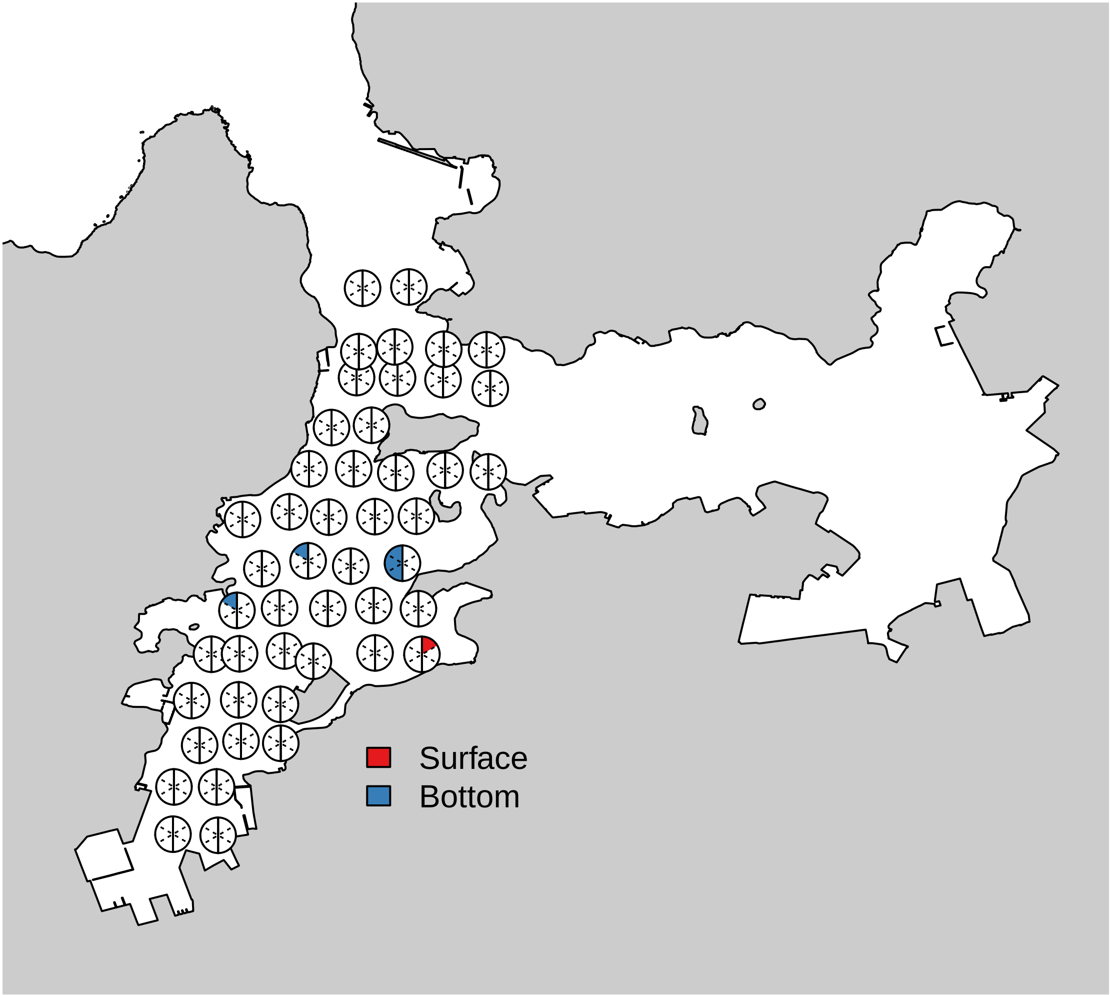

Fig.S1_Oplegnathus_fasciatus

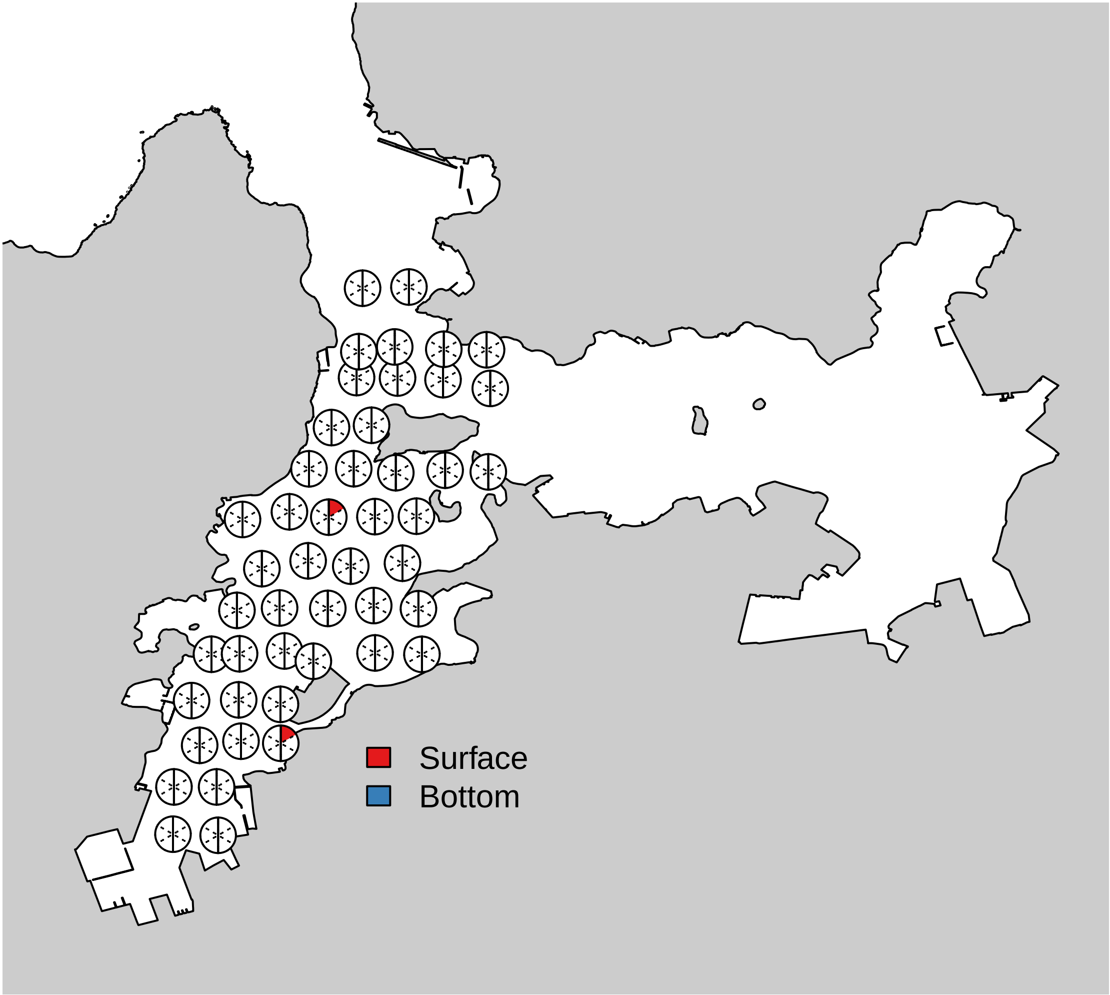

Fig.S1_Pagrus_major

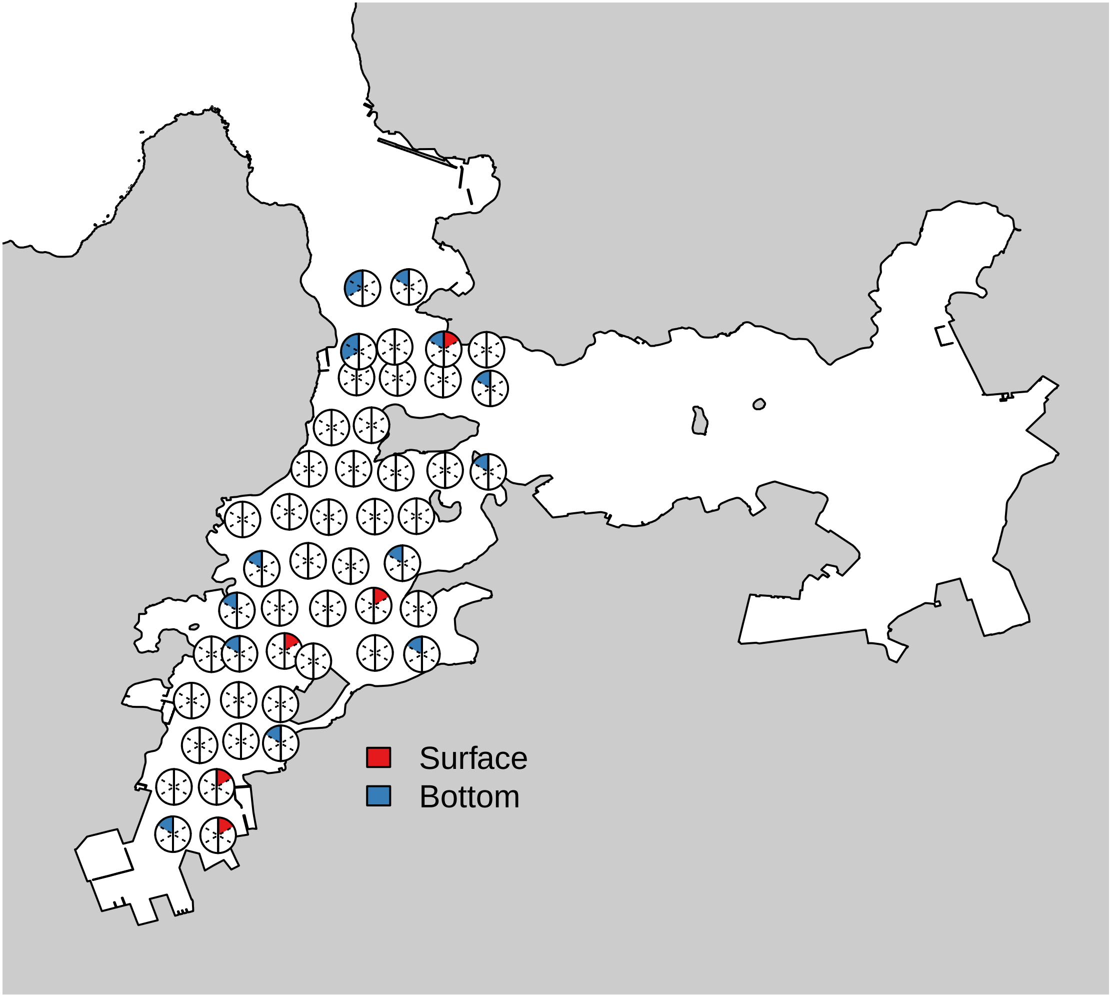

Fig.S1_Parablennius_yatabei

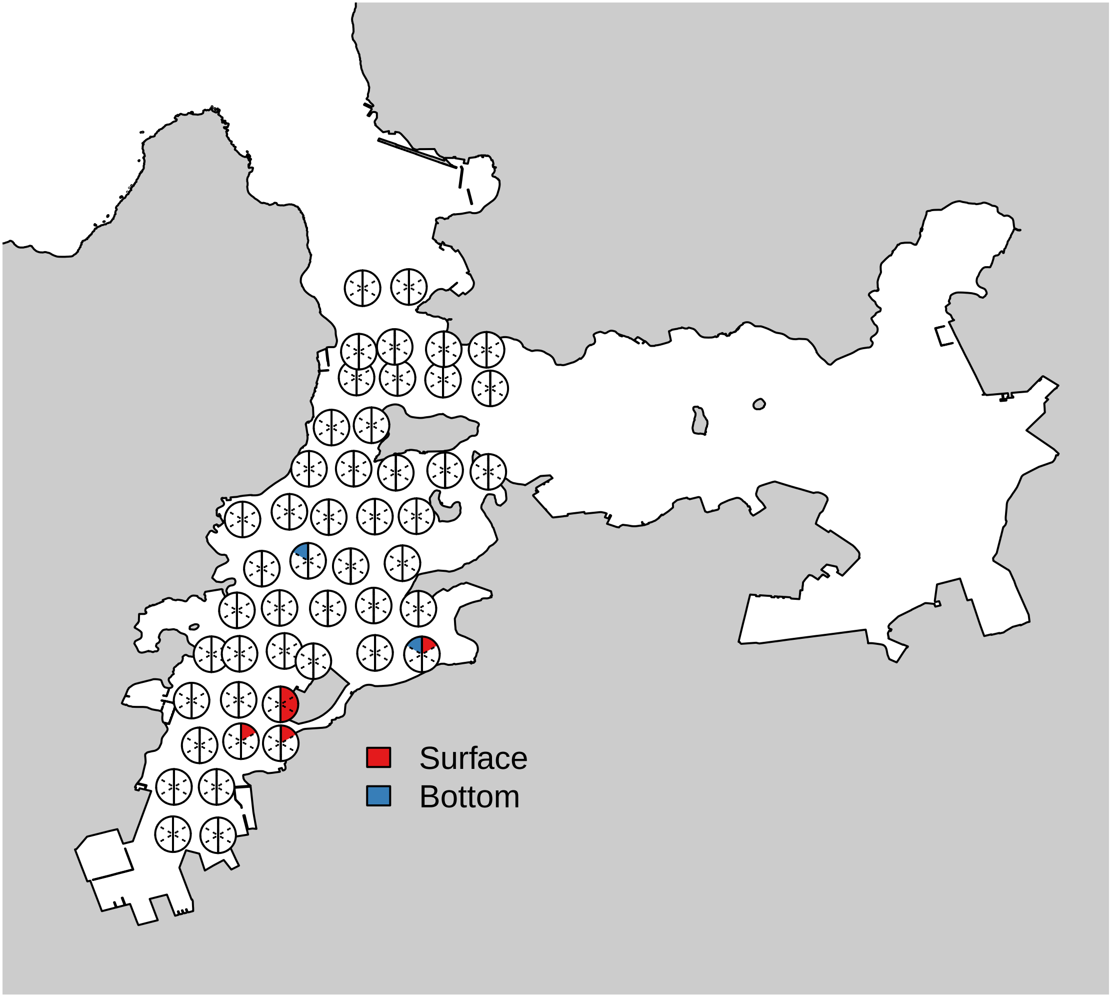

Fig.S1_Paracentropogon_rubripinnis

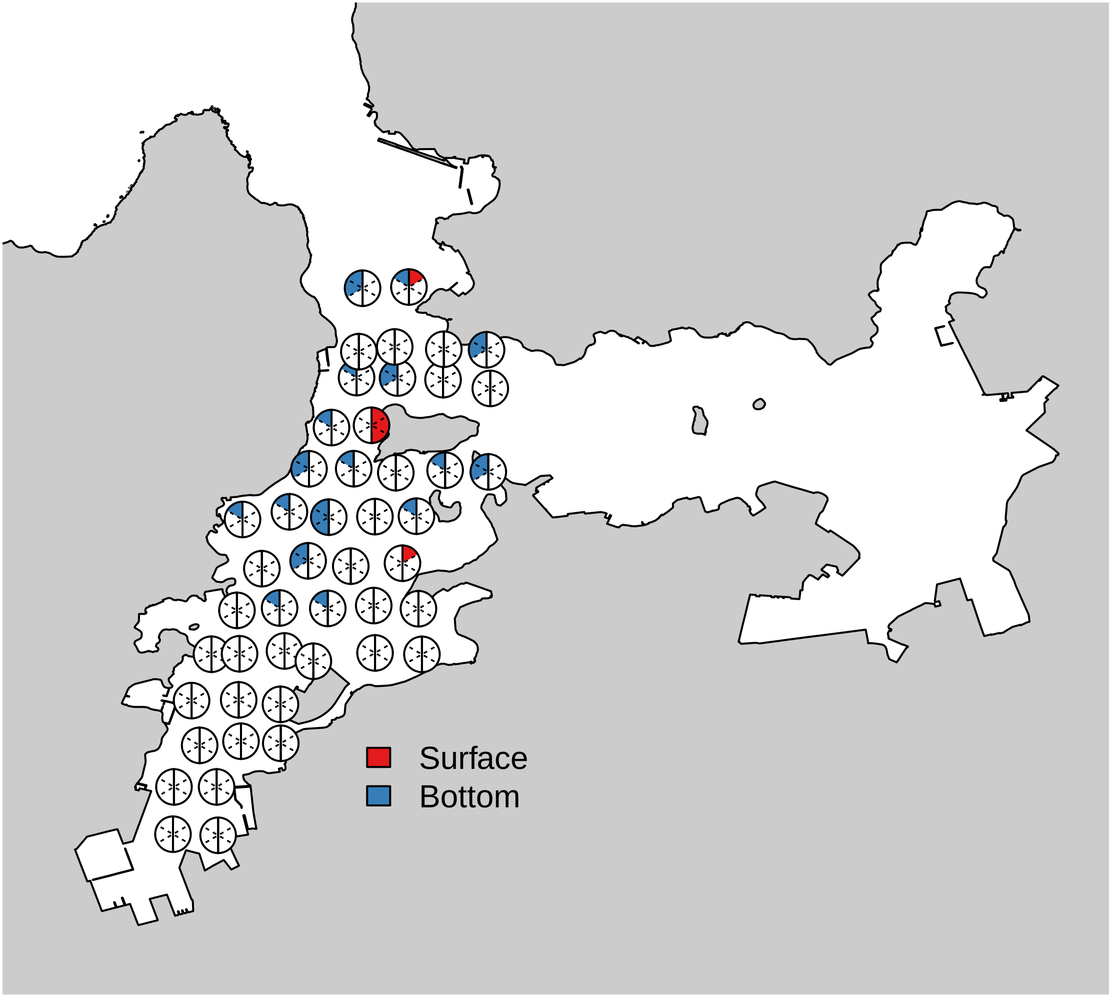

Fig.S1_Paralichthys_olivaceus

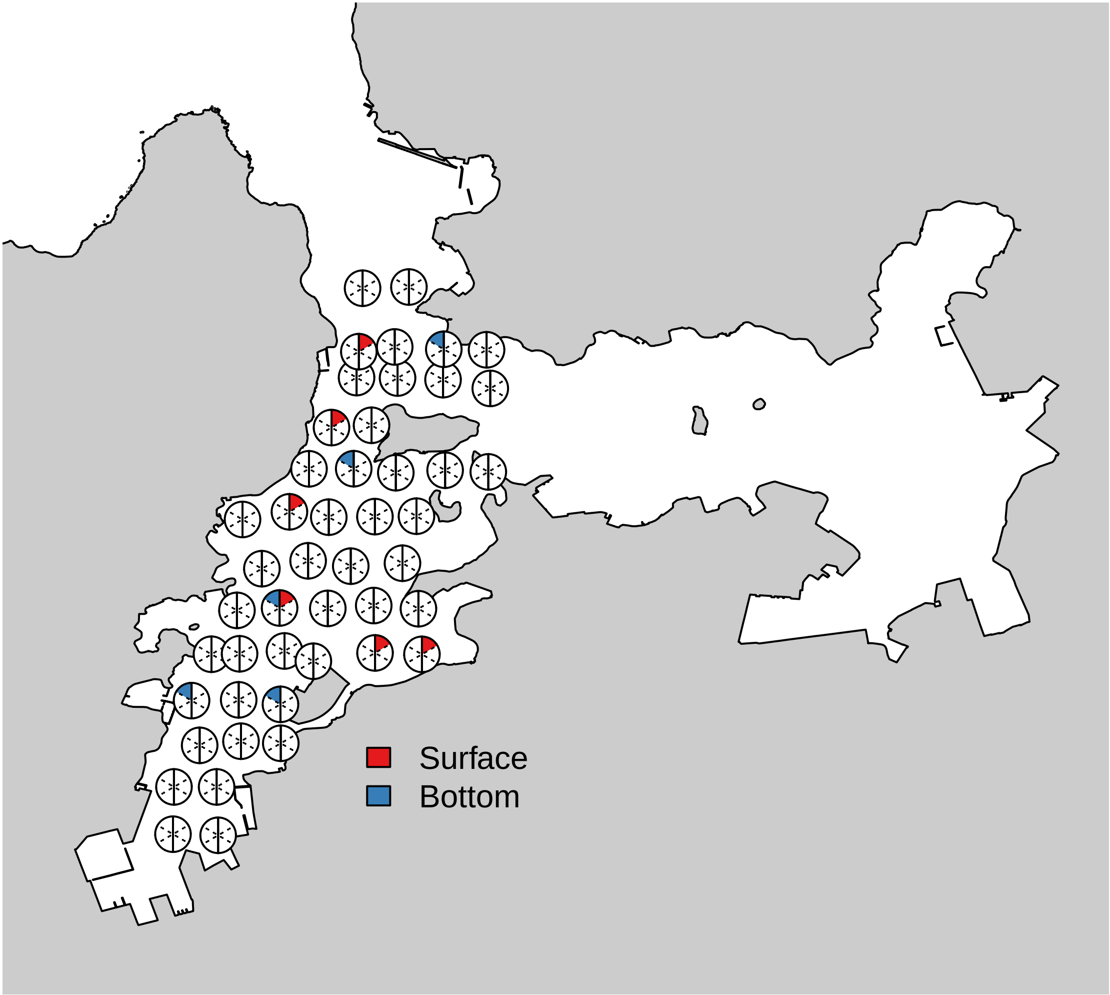

Fig.S1_Parapristipoma_trilineatum

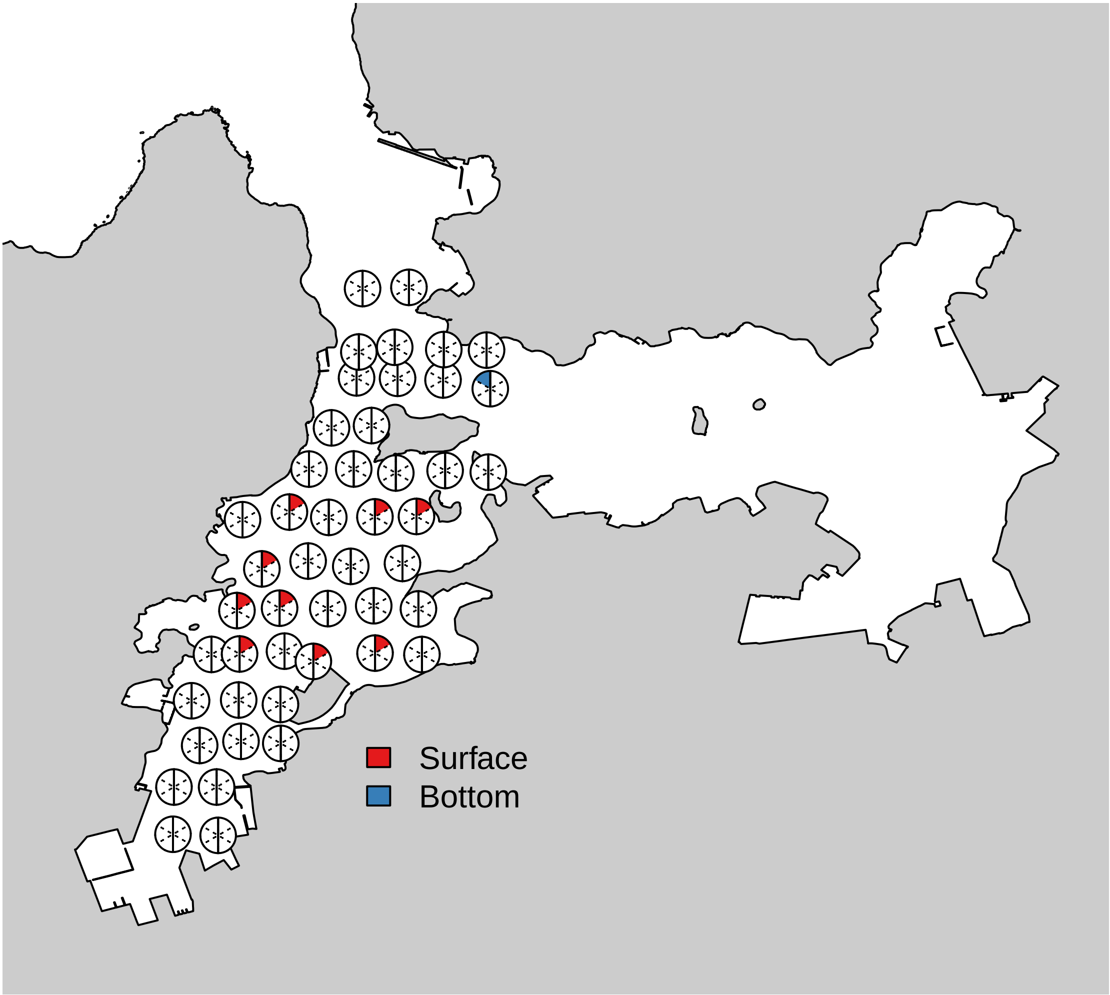

Fig.S1_Paratrypauchen_microcephalus

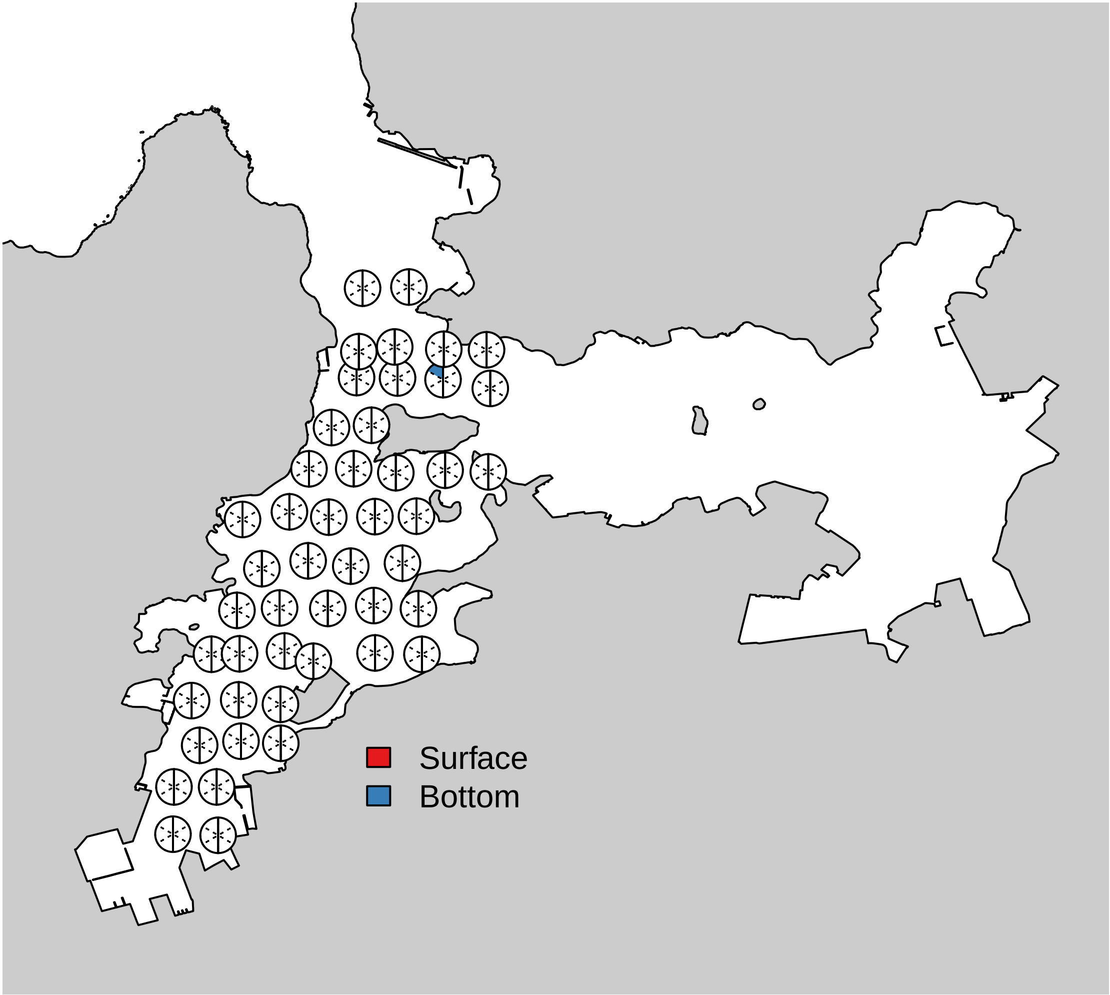

Fig.S1_Pennahia_argentata

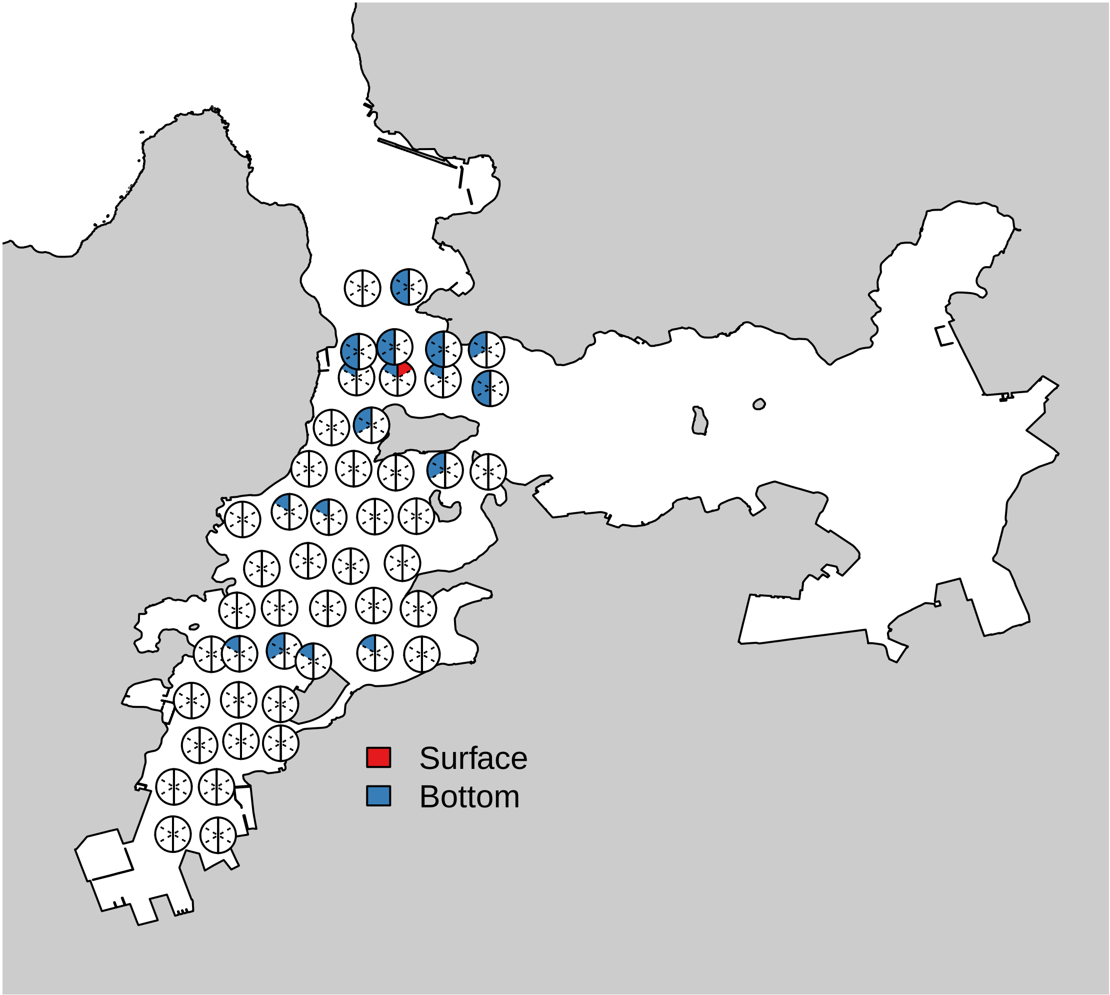

Fig.S1_Pholis_fangi

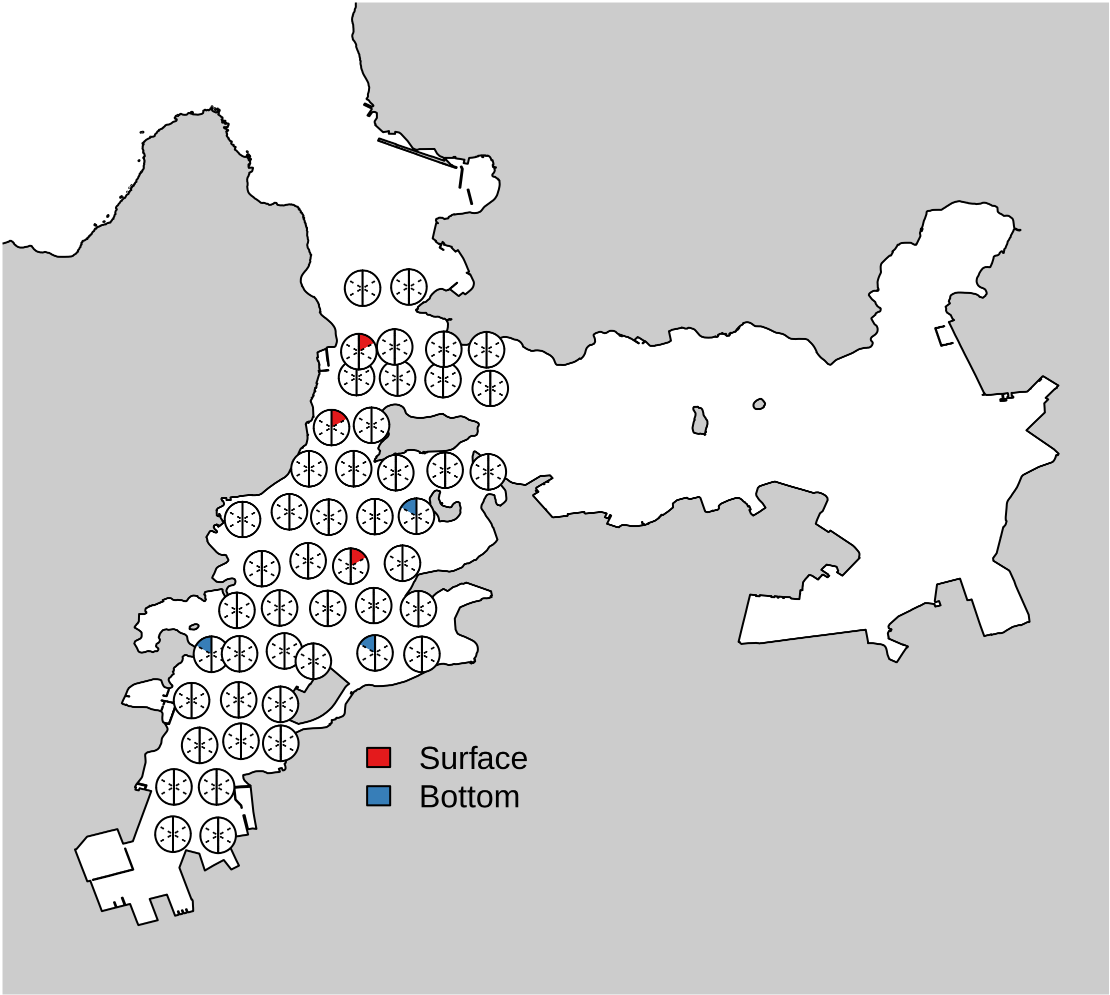

Fig.S1_Pholis_nebulosa

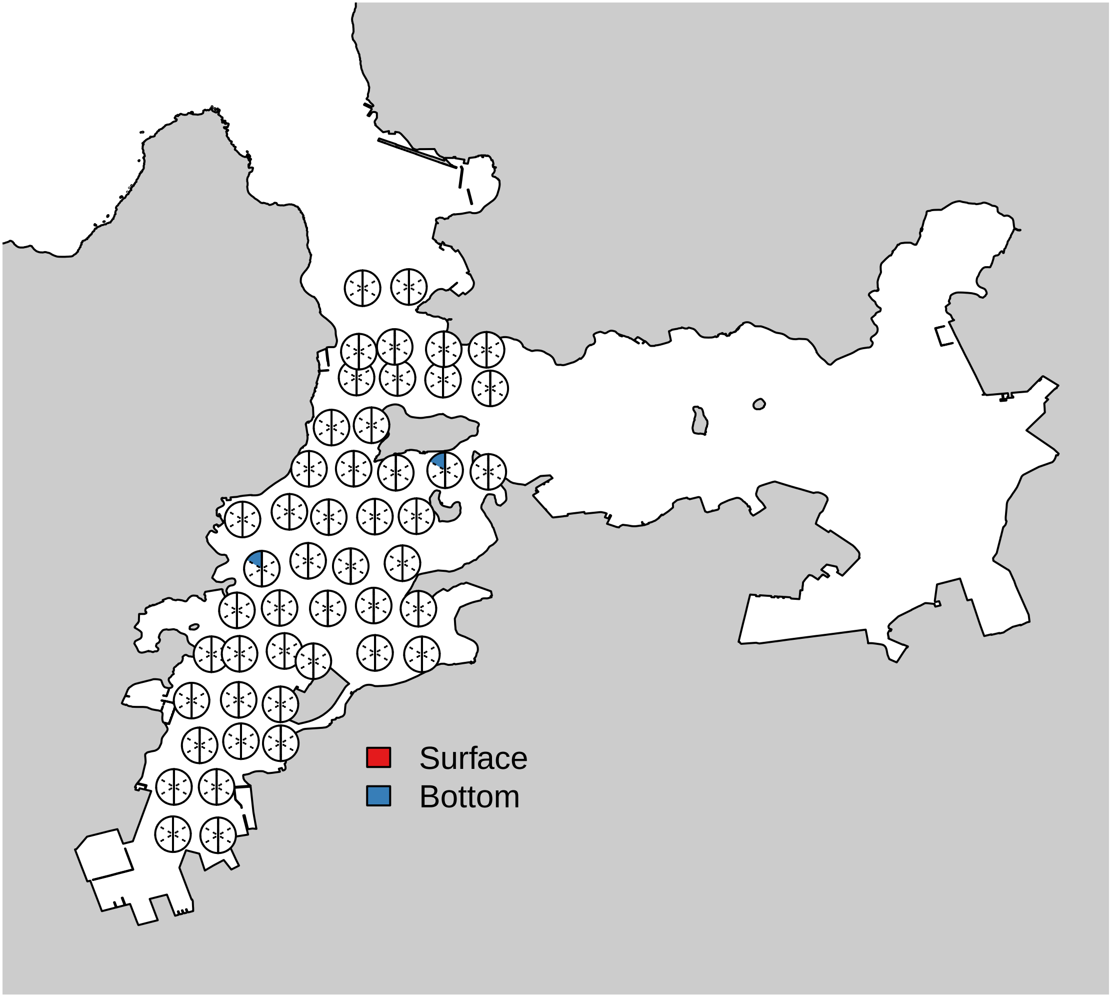

Fig.S1_Platycephalus_sp.MAGOCHI

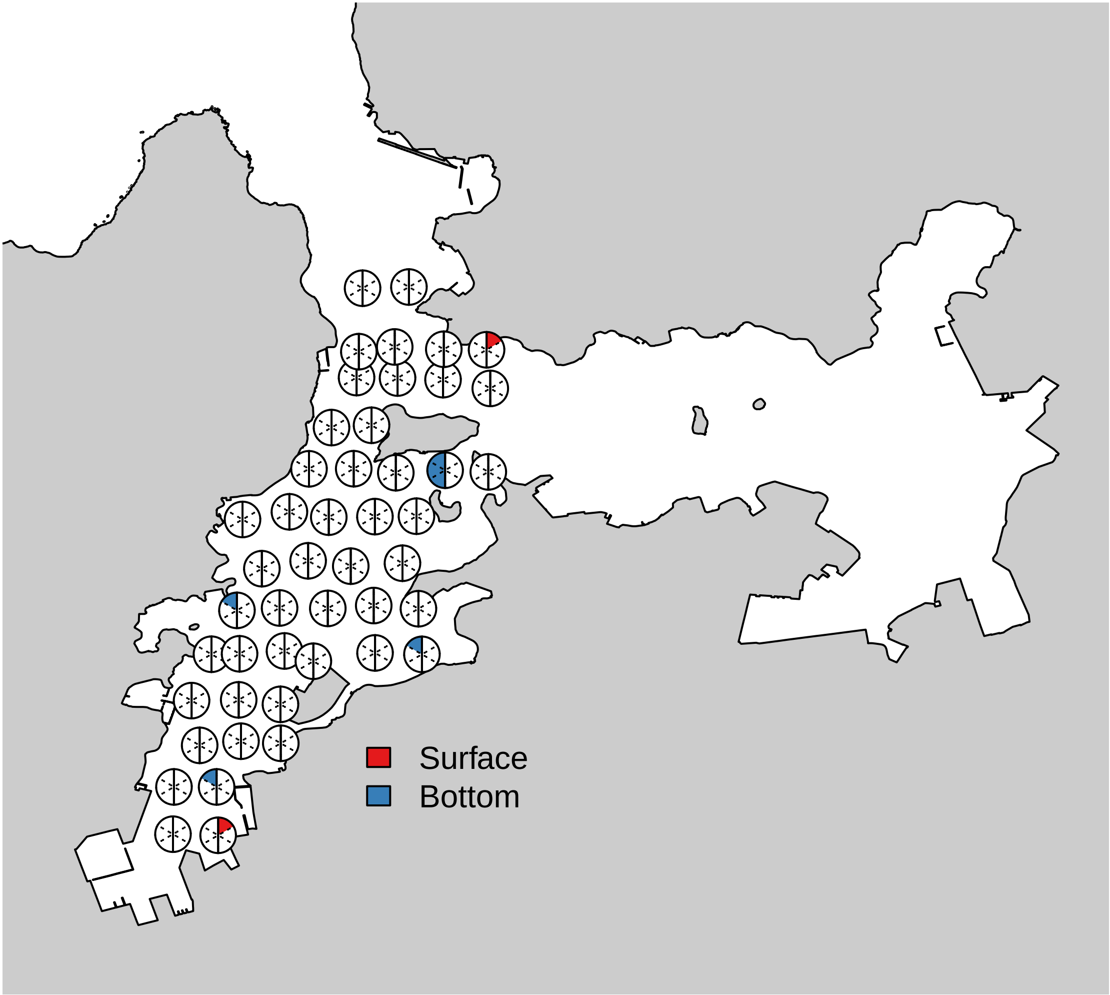

Fig.S1_Plecoglossus_altivelis_altivelis

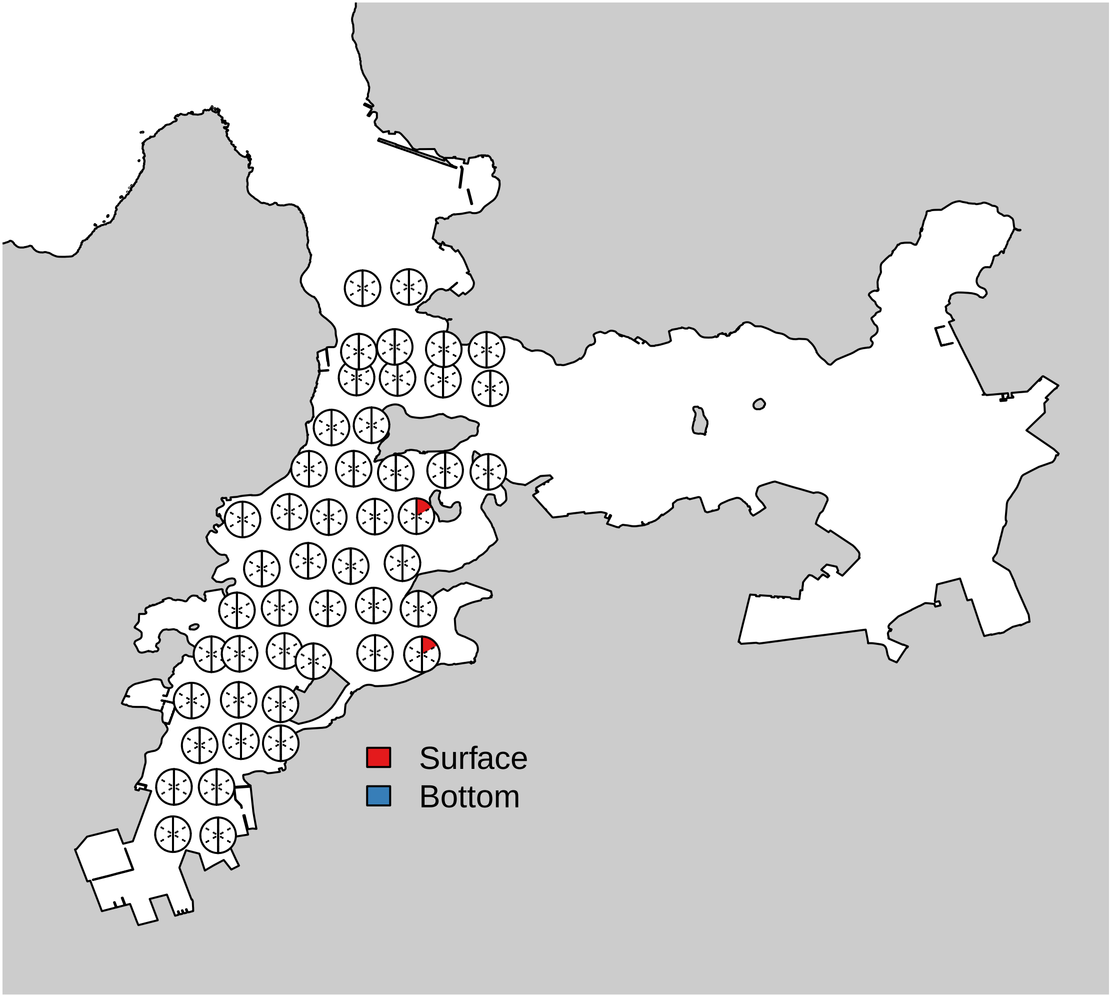

Fig.S1_Plectorhinchus_cinctus

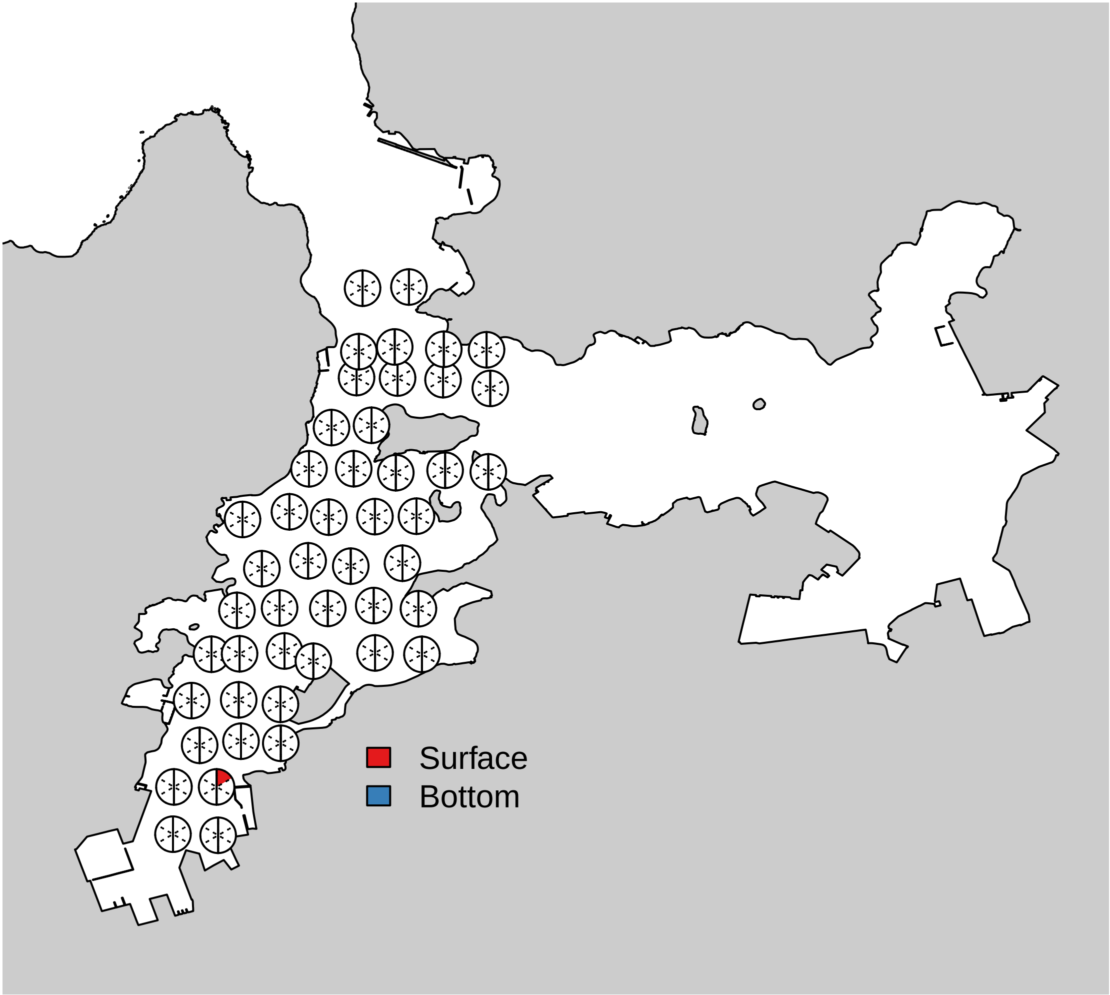

Fig.S1_Pleuronectes_sp.

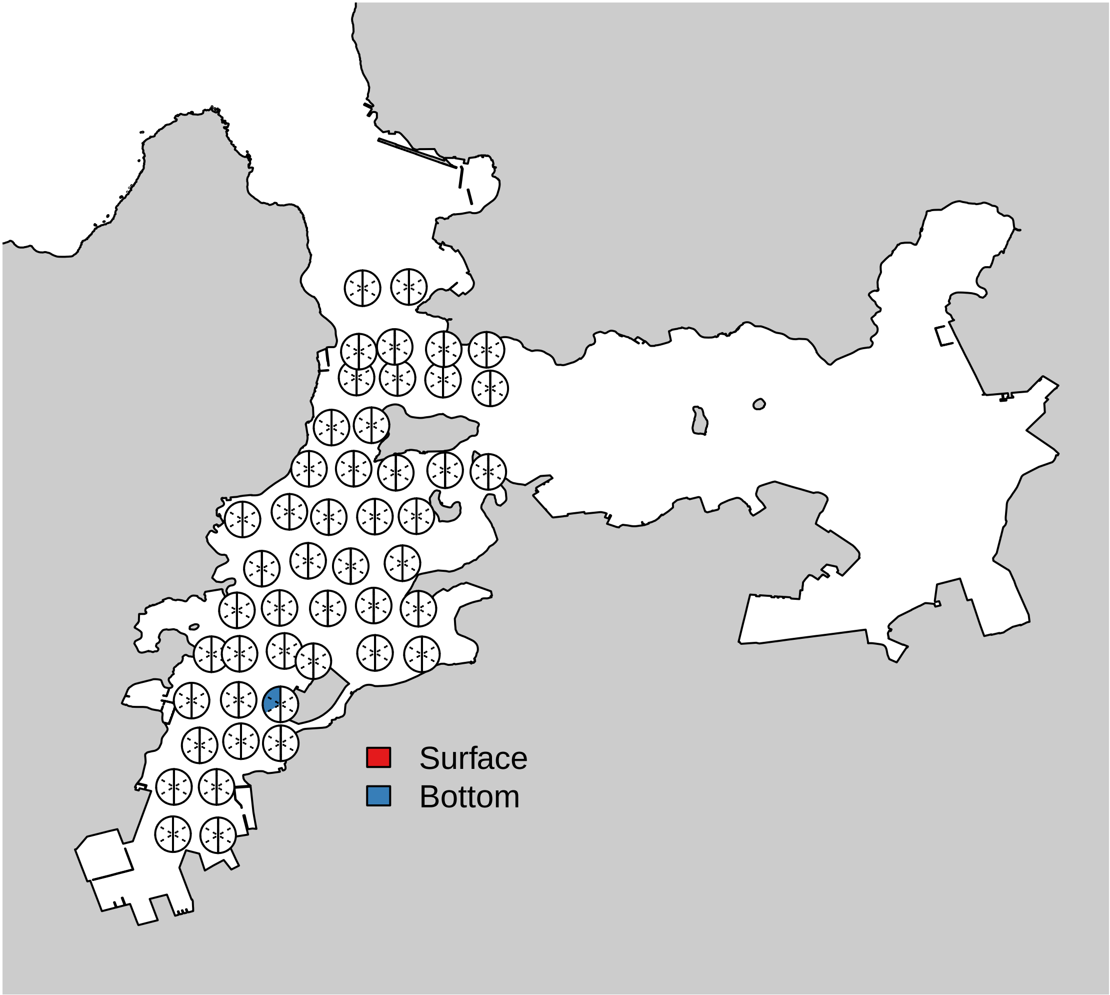

Fig.S1_Pleuronichthys_cornutus

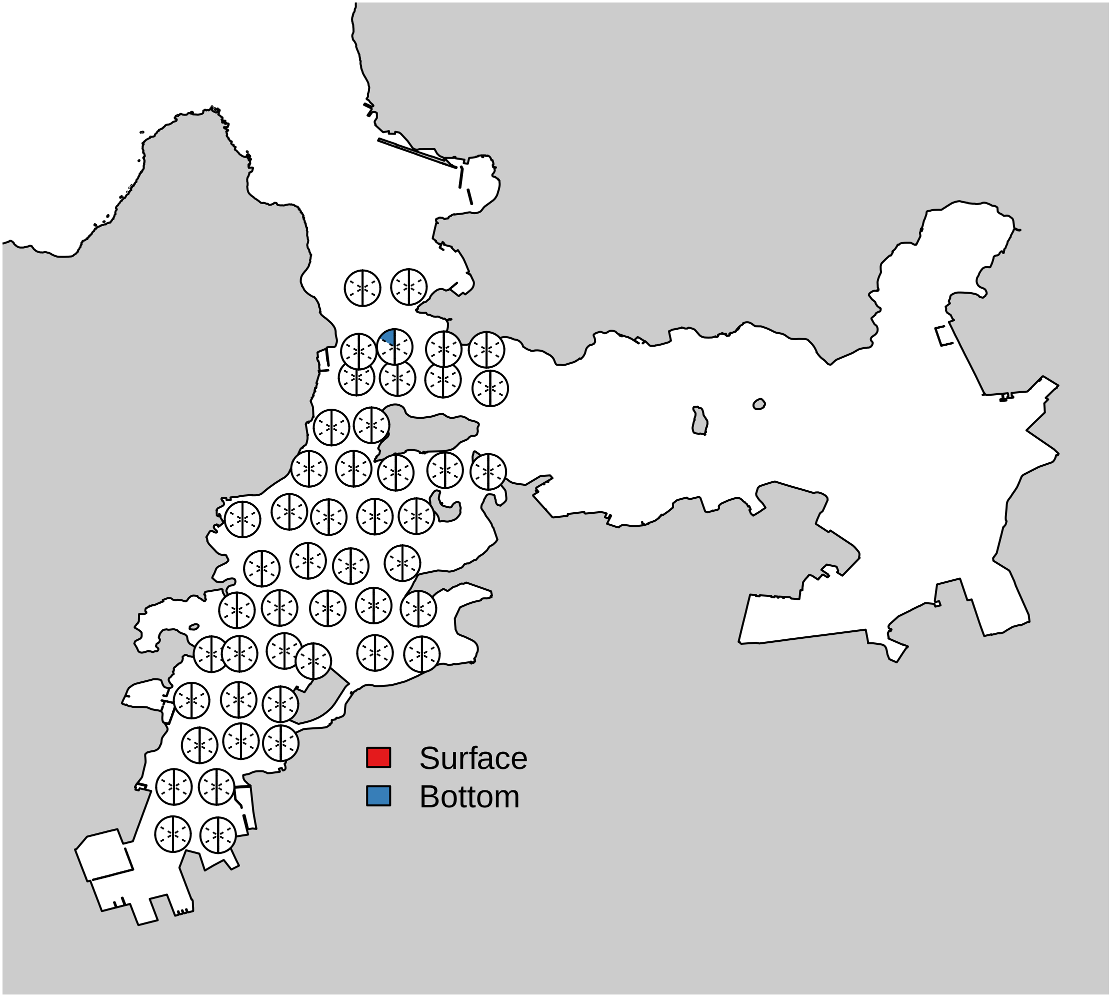

Fig.S1_Plotosus_japonicus

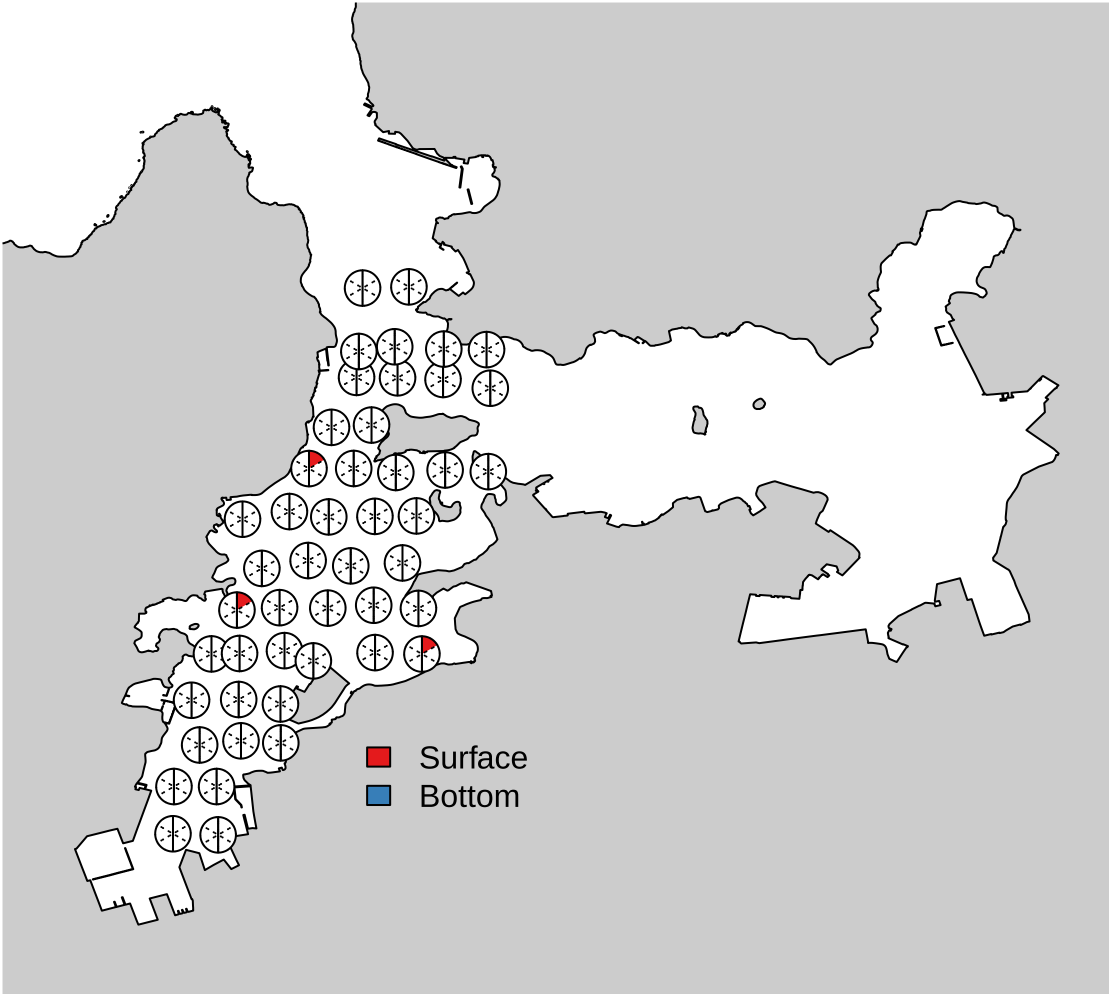

Fig.S1_Pseudaesopia_japonica

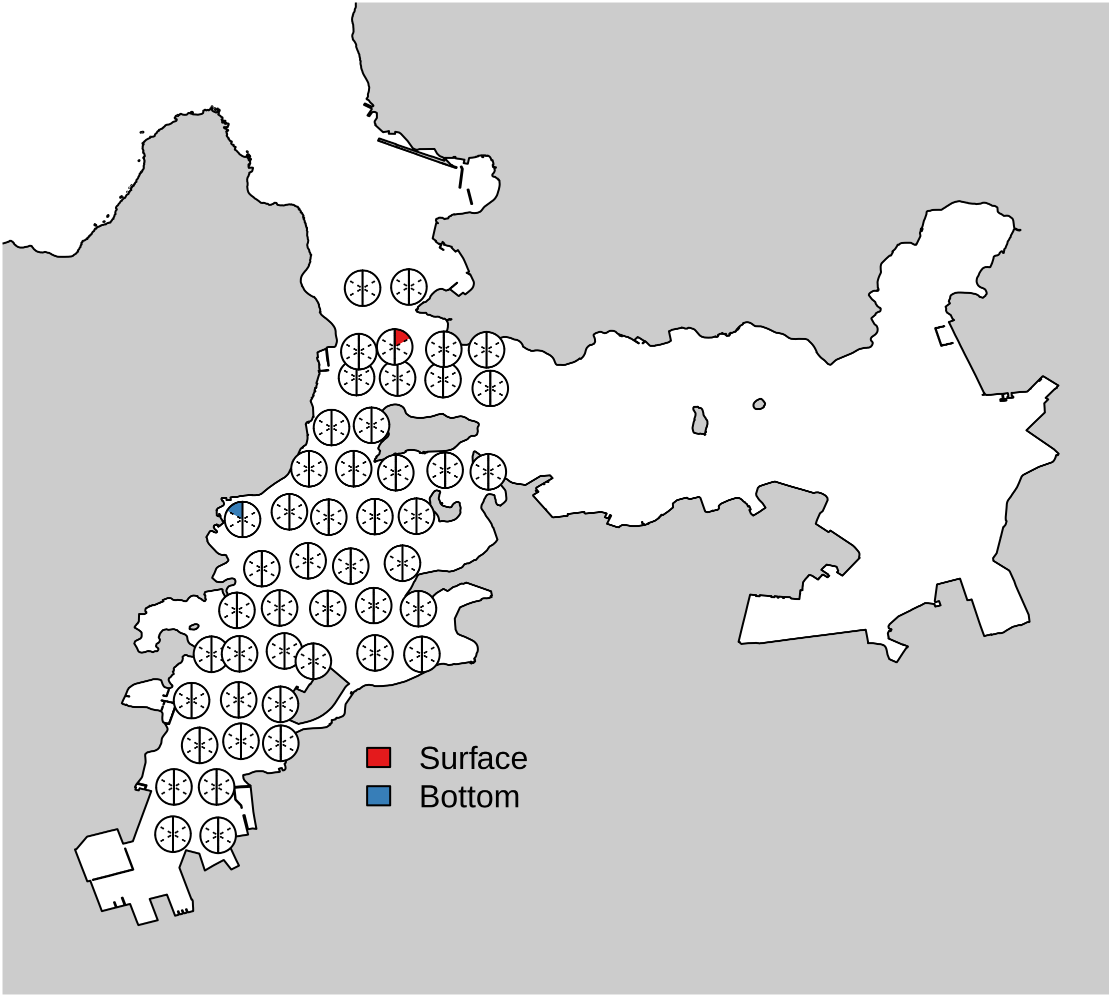

Fig.S1_Pseudogobio_esocinus

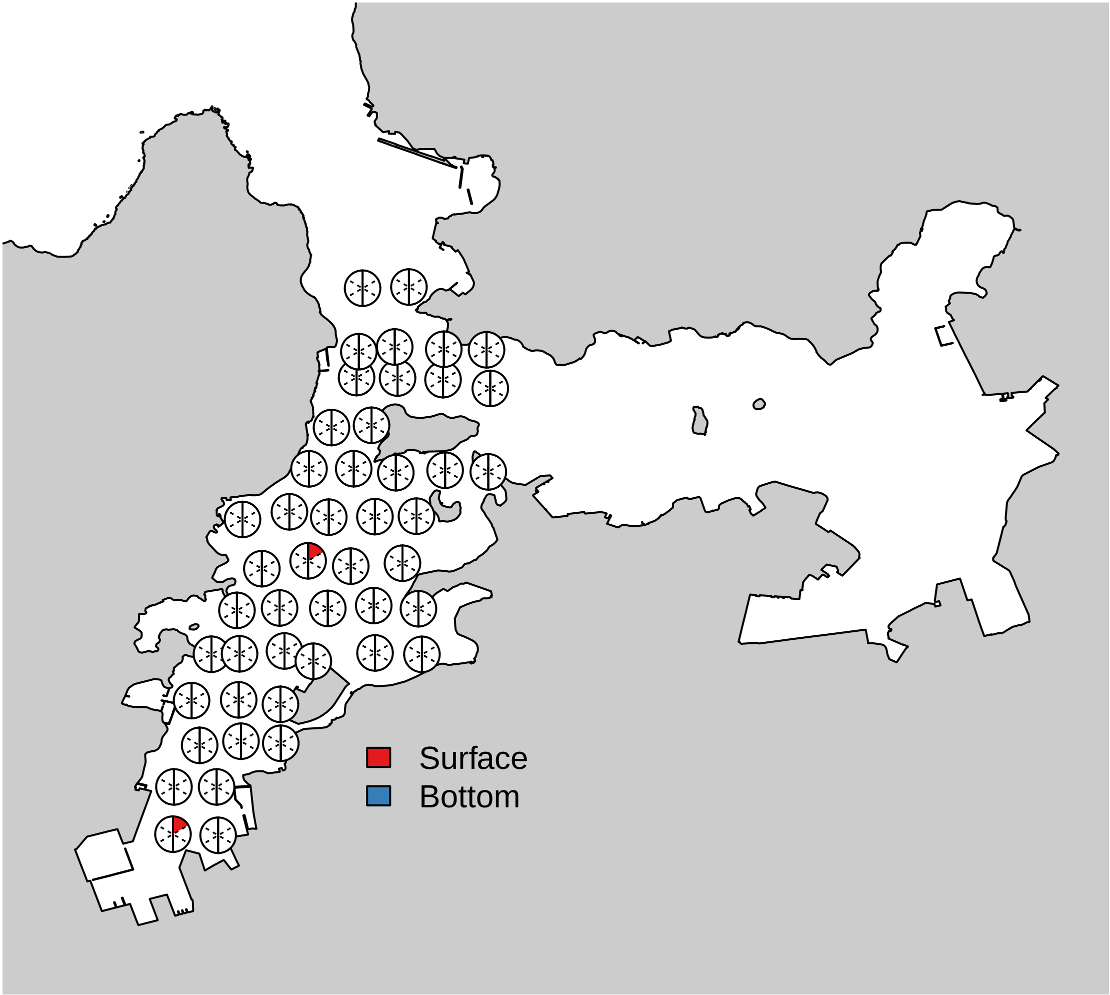

Fig.S1_Pseudolabrus_sieboldi

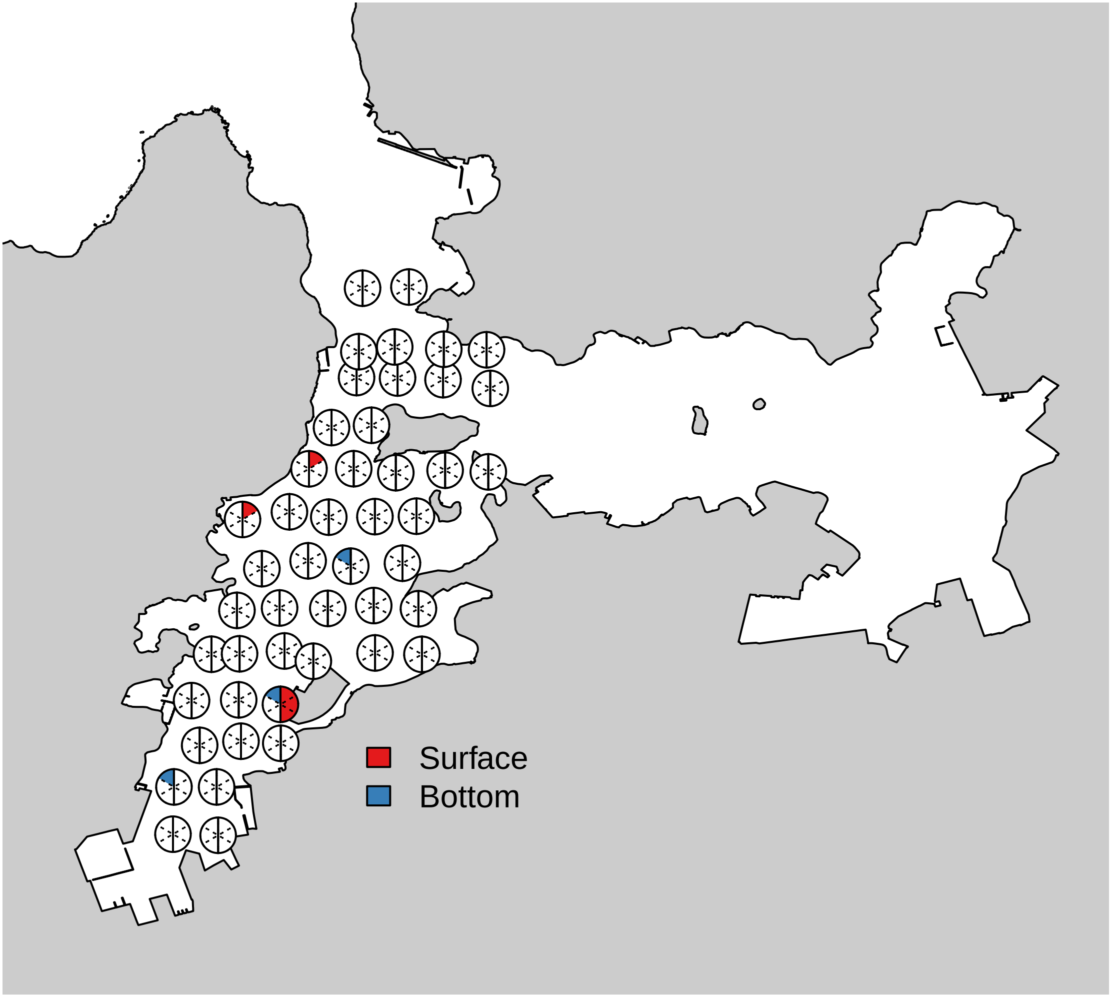

Fig.S1_Pseudorhombus_sp.

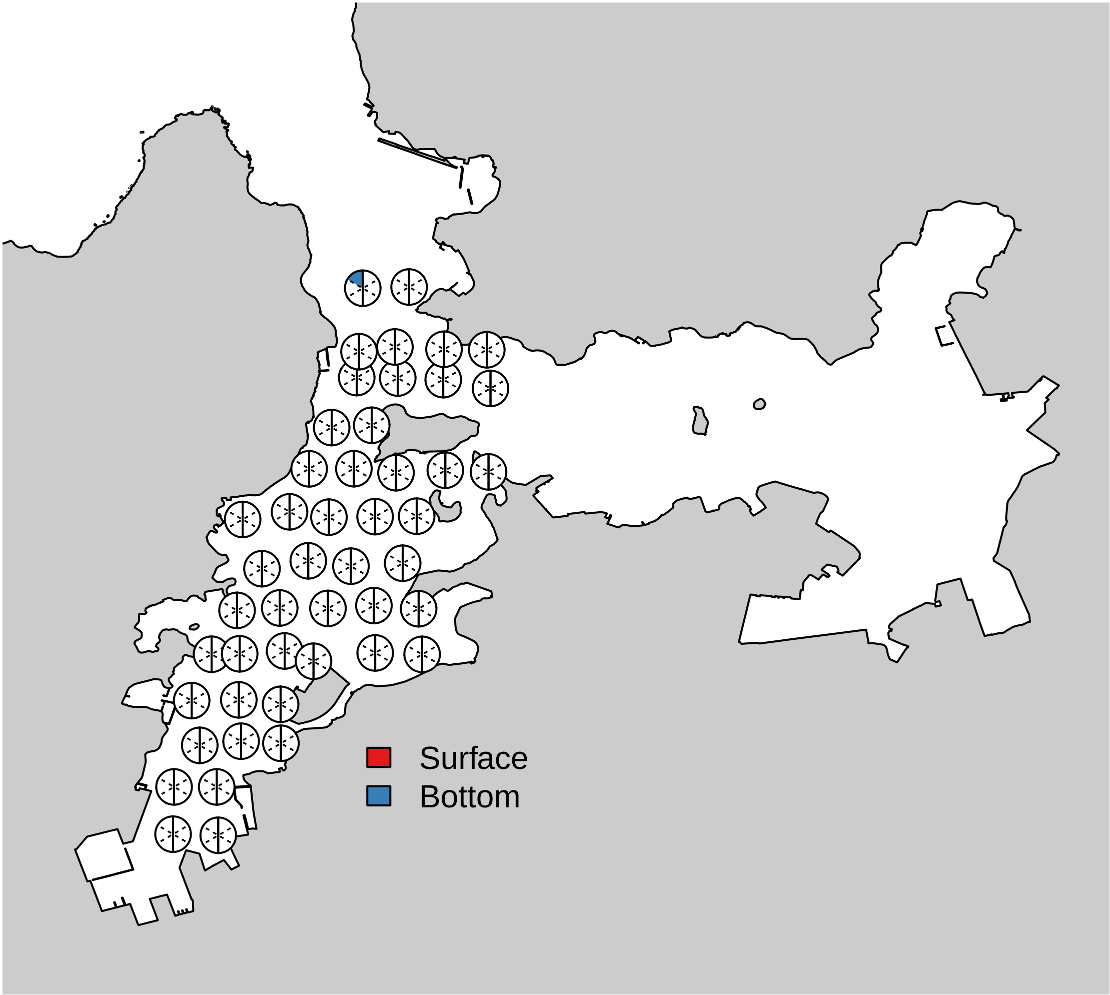

Fig.S1_Pungtungia_herzi

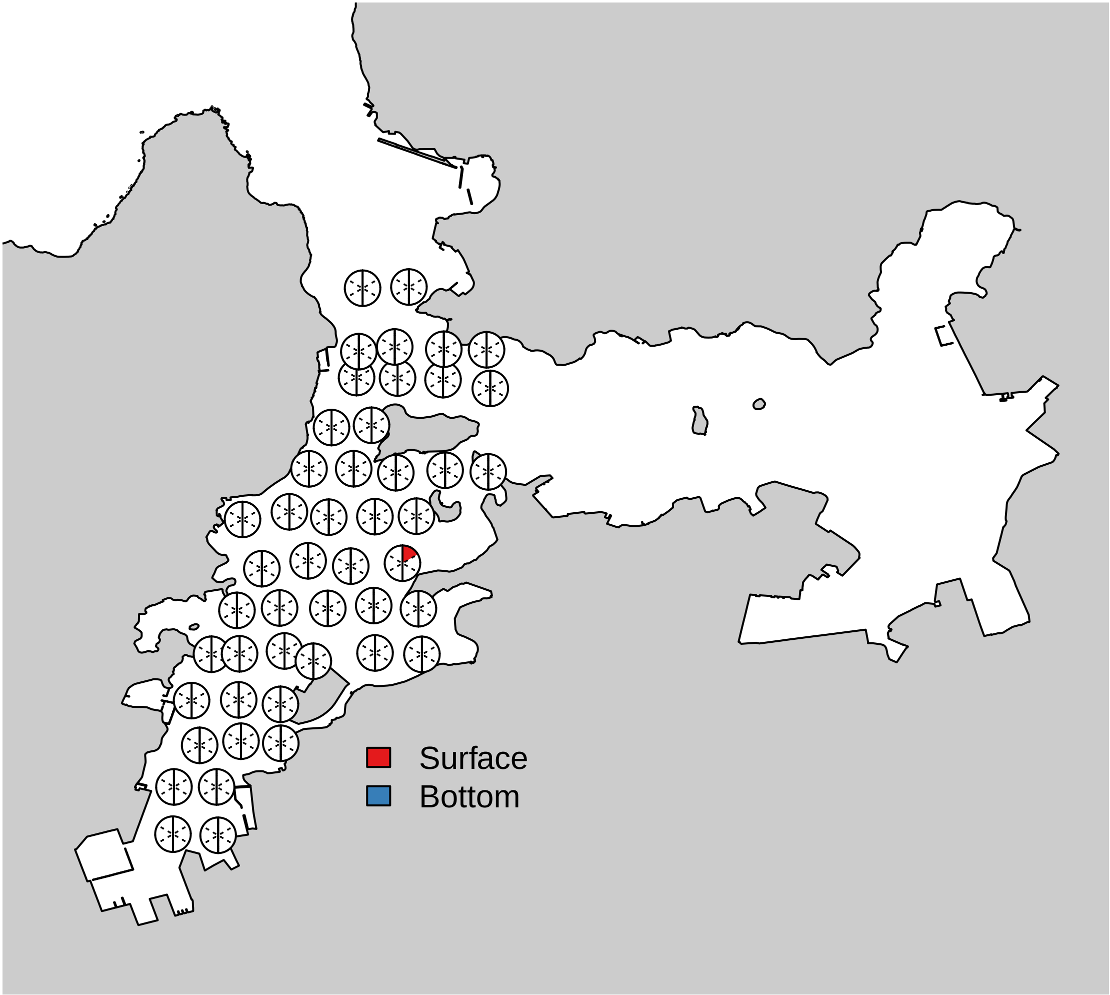

Fig.S1_Repomucenus_valenciennei

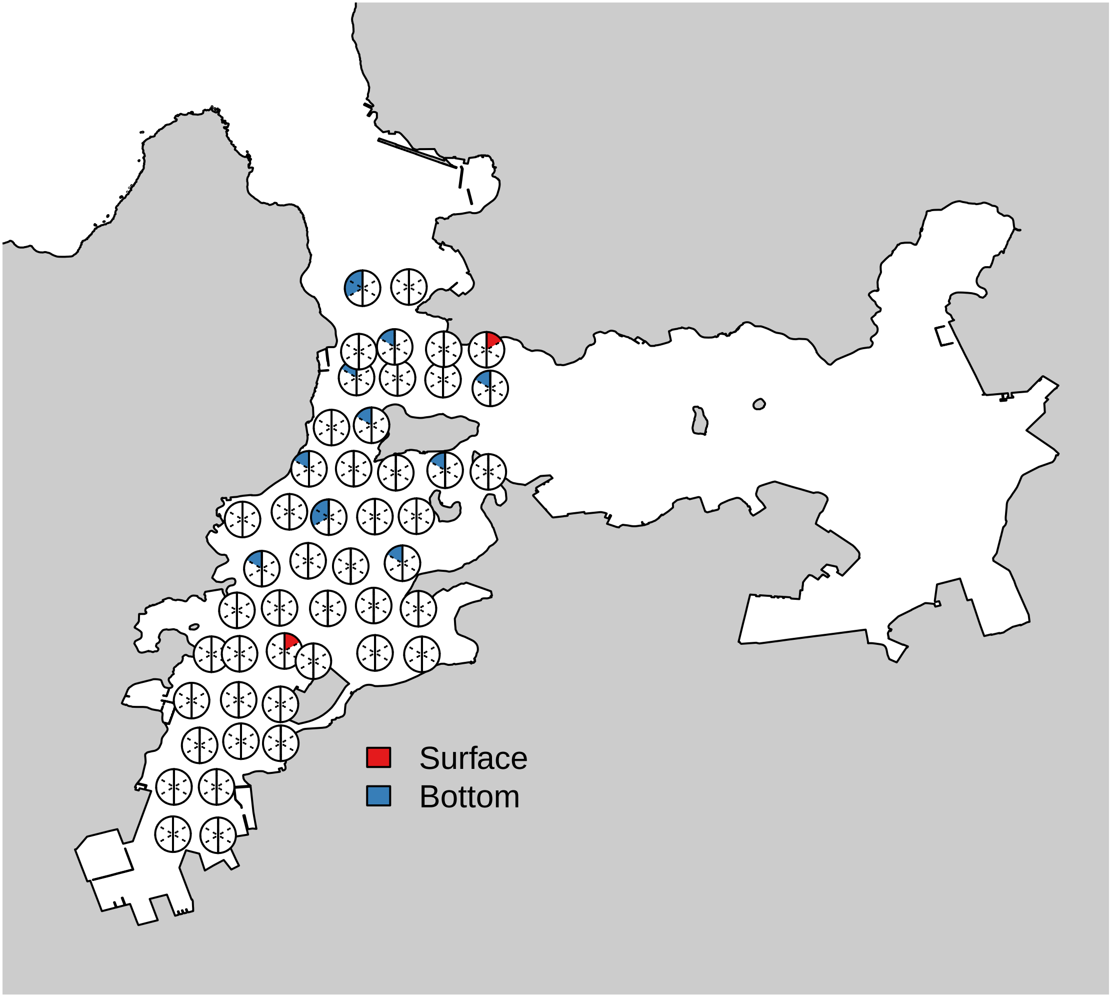

Fig.S1_Rhinogobius_flumineus

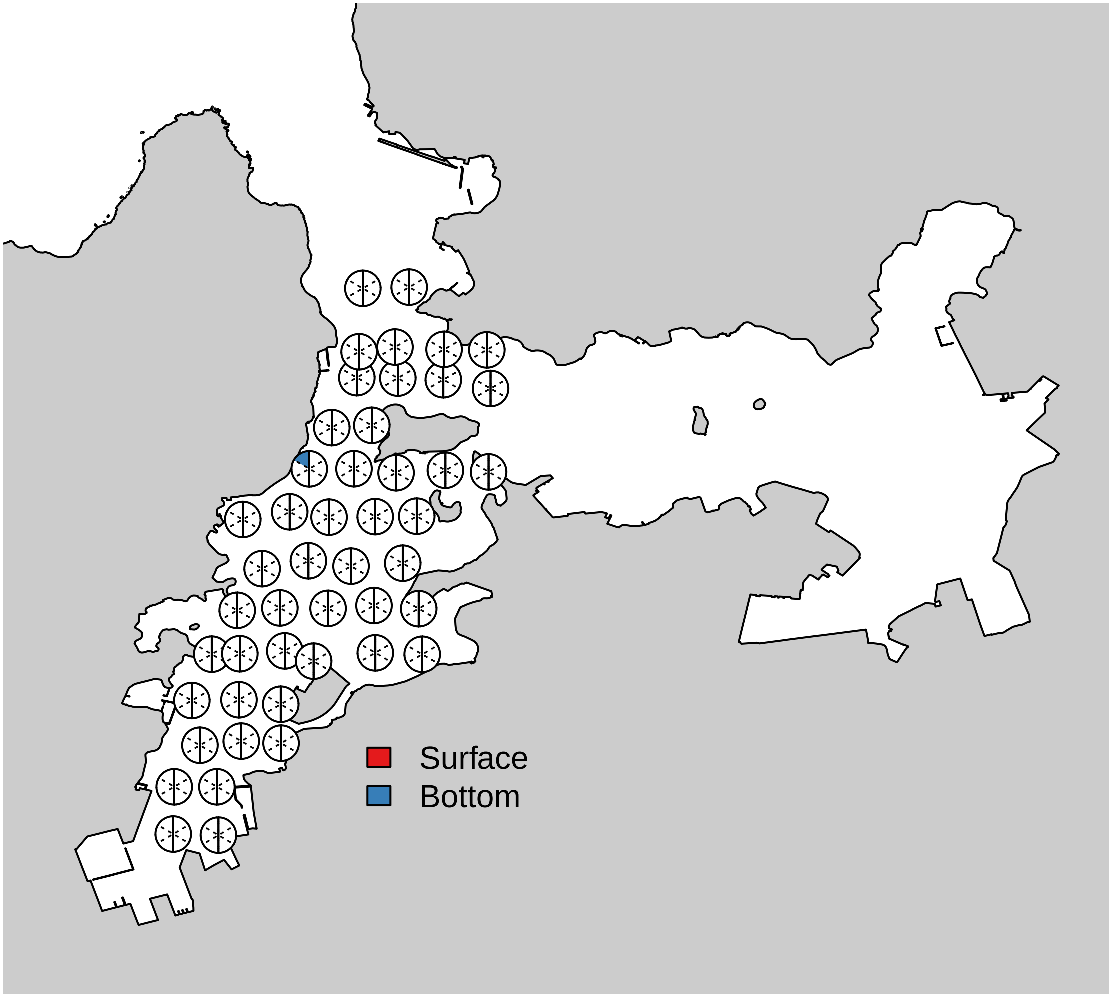

Fig.S1_Rhinogobius_giurinus

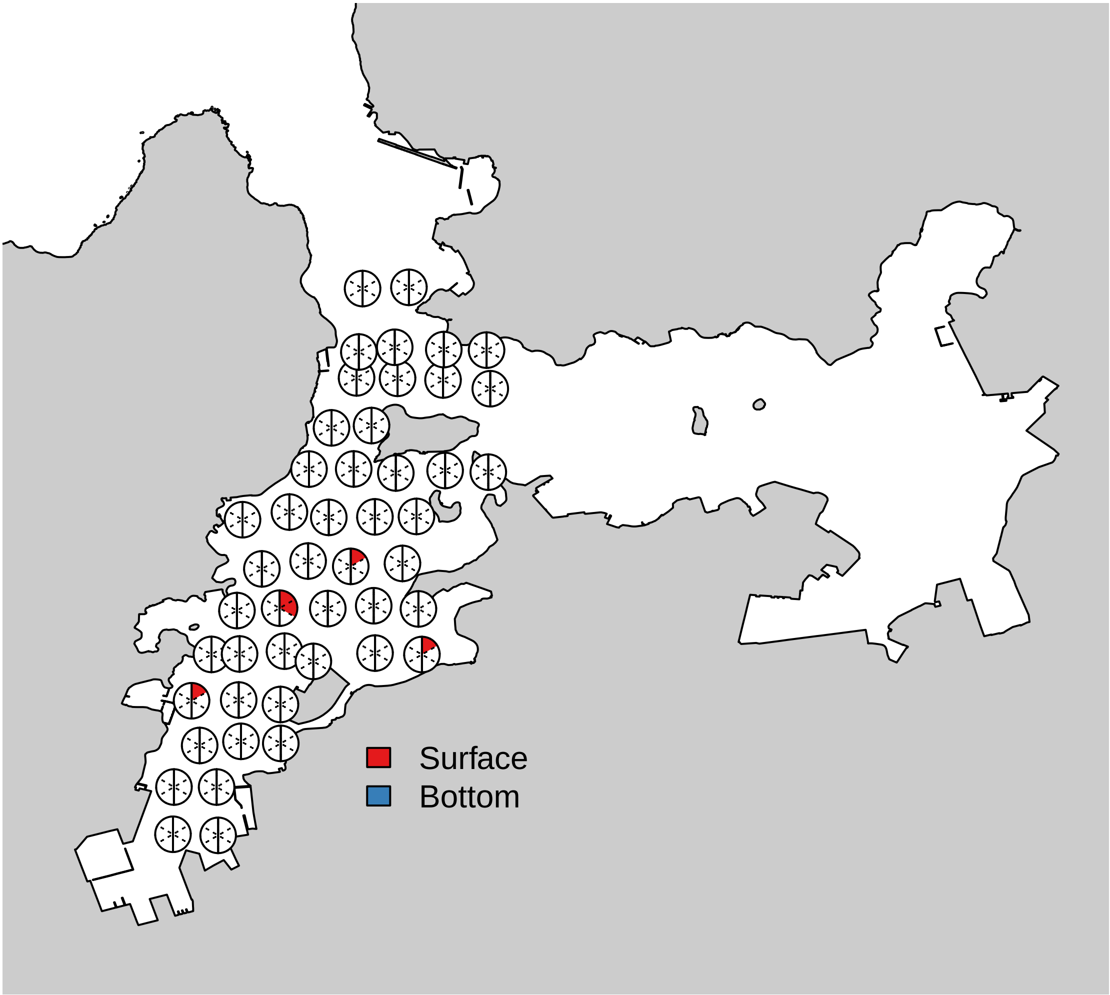

Fig.S1_Rhynchopelates_oxyrhynchus

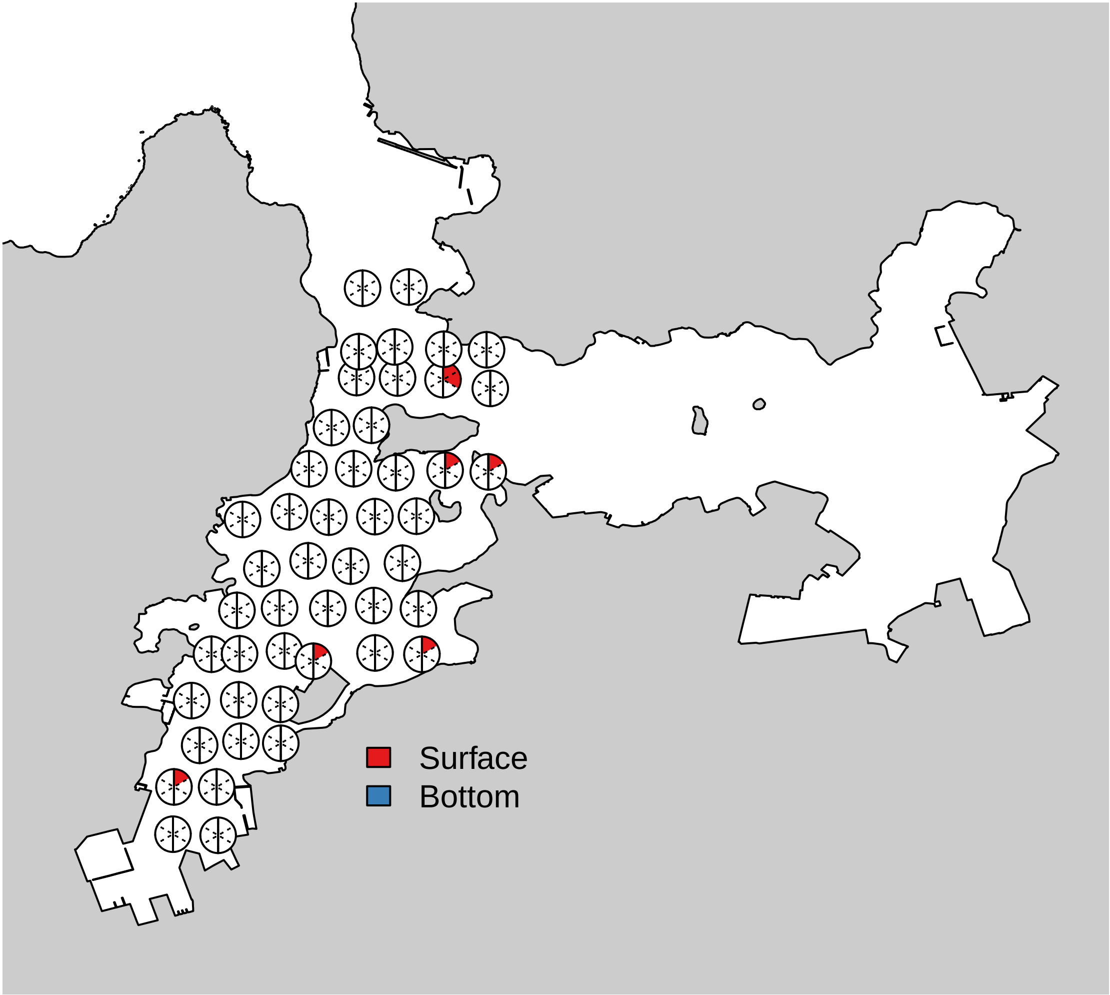

Fig.S1_Rudarius_ercodes

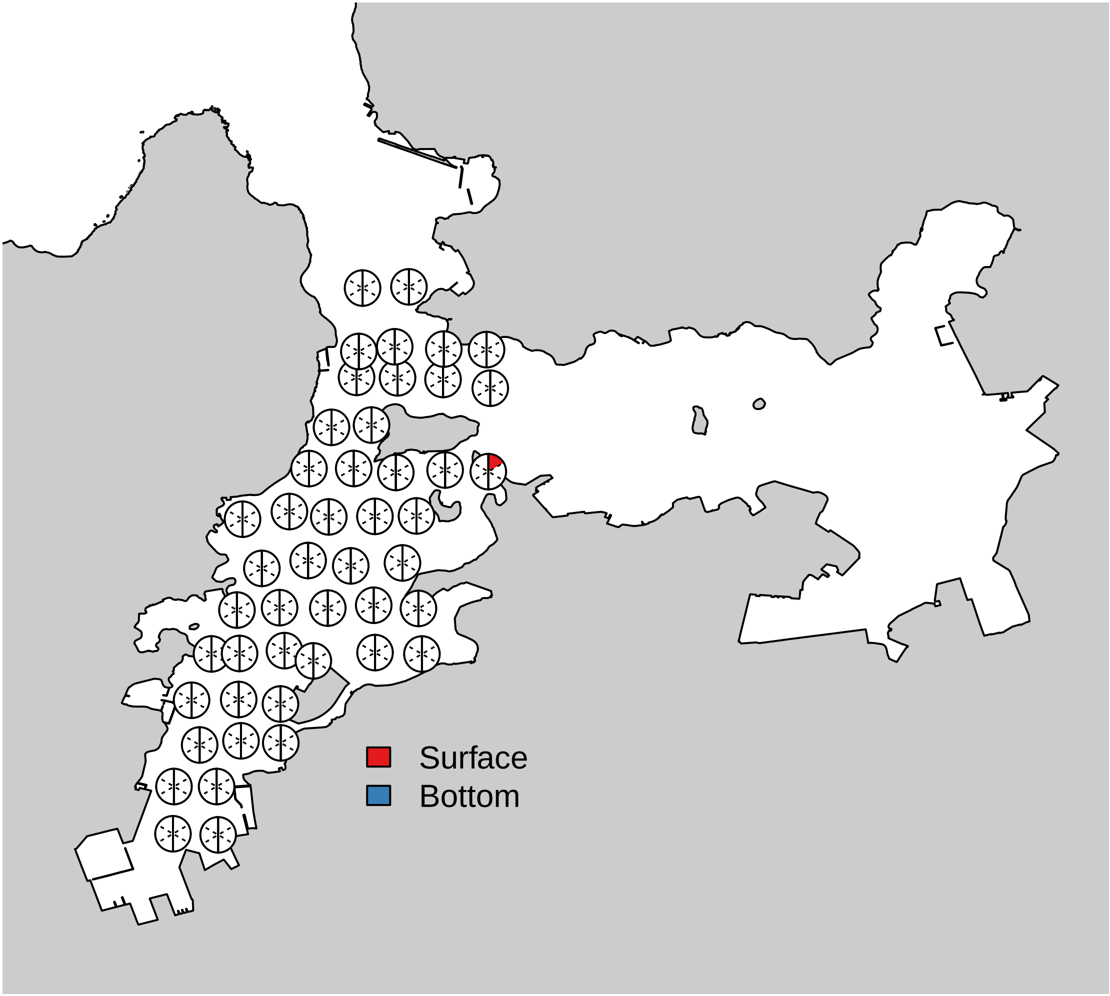

Fig.S1_Sarda_orientalis

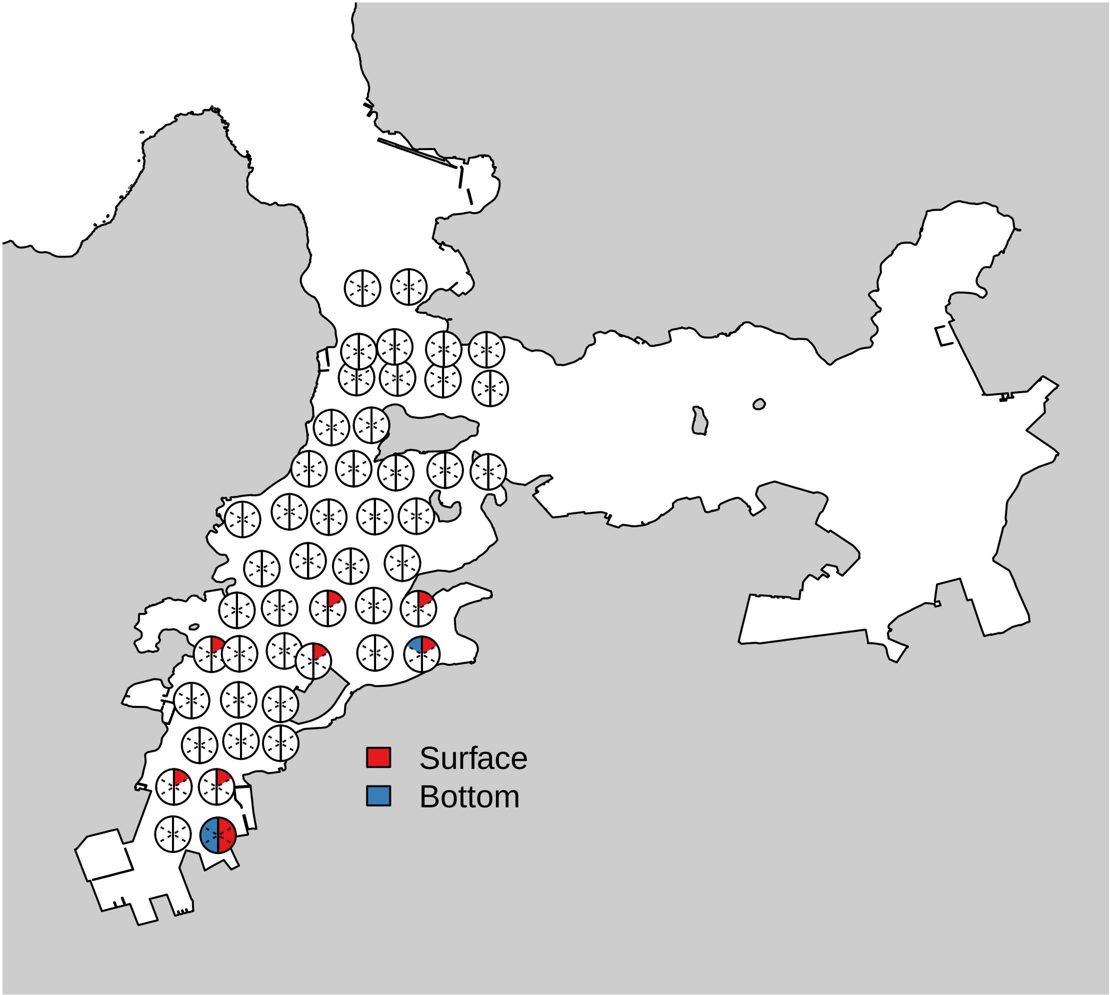

Fig.S1_Sardinops_melanostictus

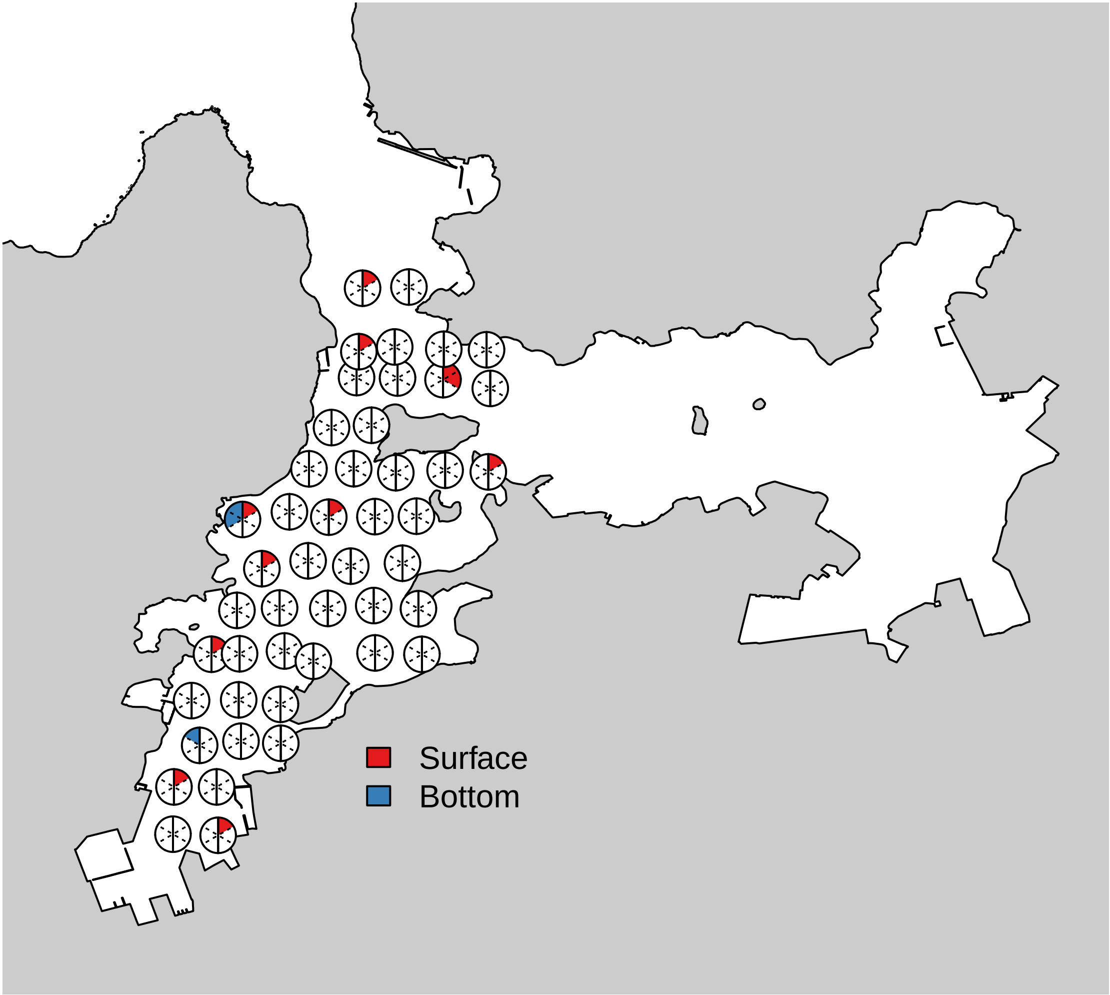

Fig.S1_Saurida_sp.

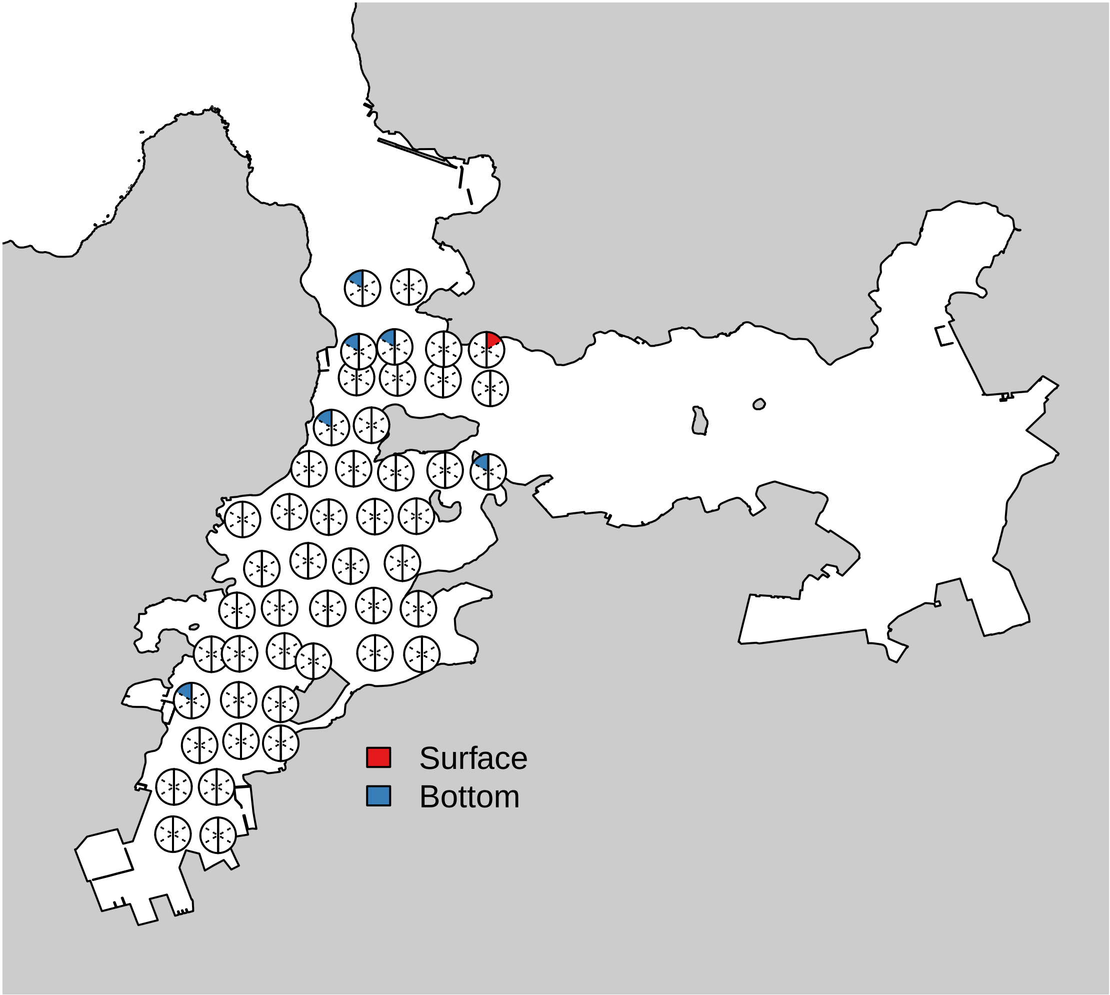

Fig.S1_Saurida_wanieso

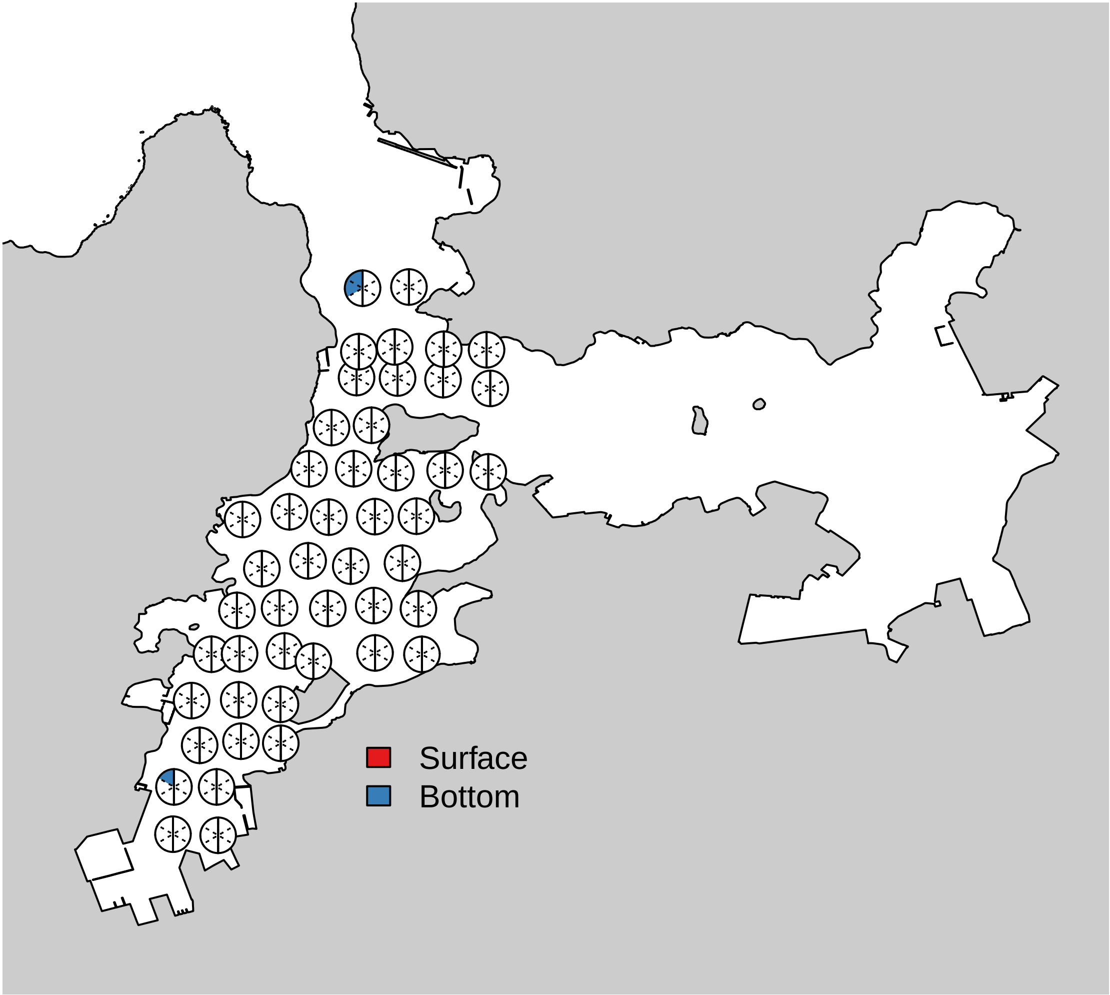

Fig.S1_Scomber_scombrus

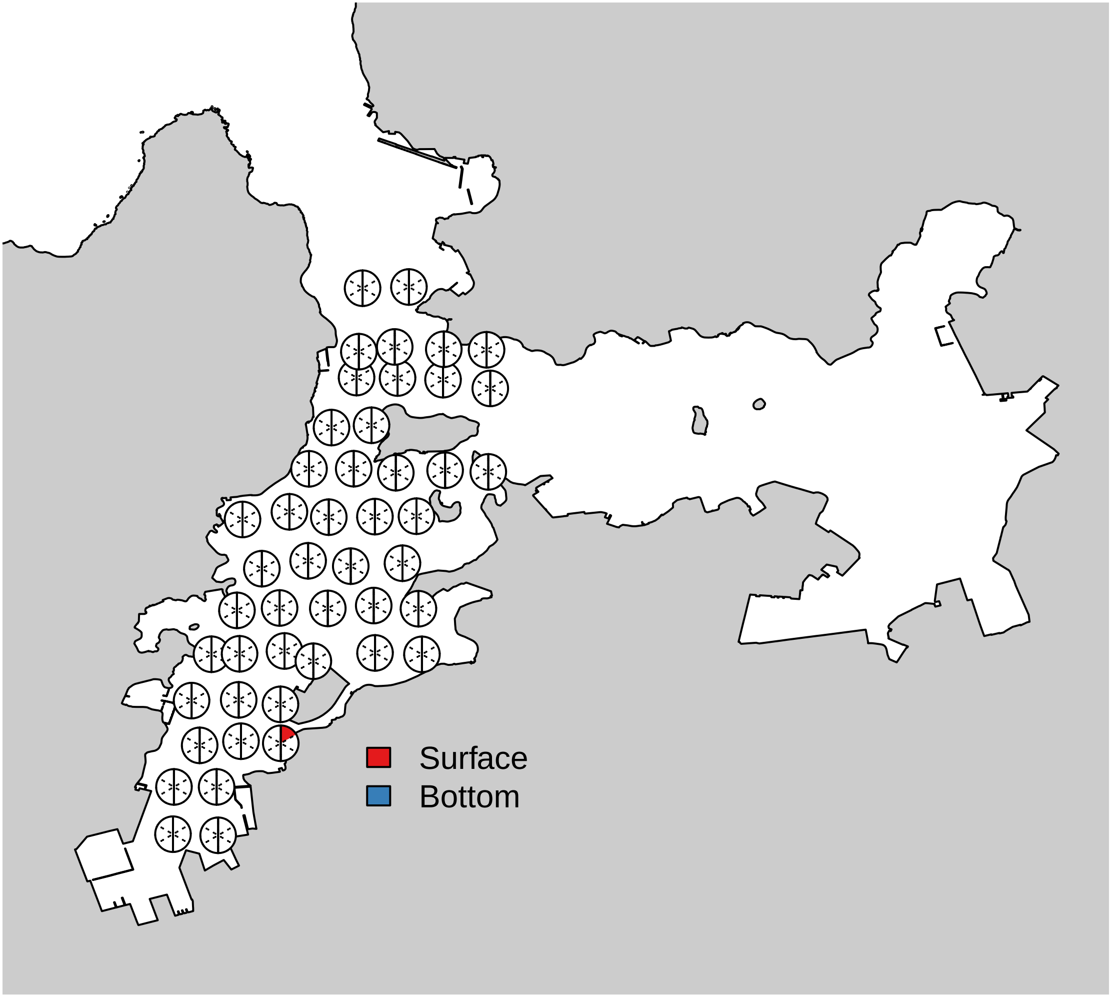

Fig.S1_Scomber_sp.

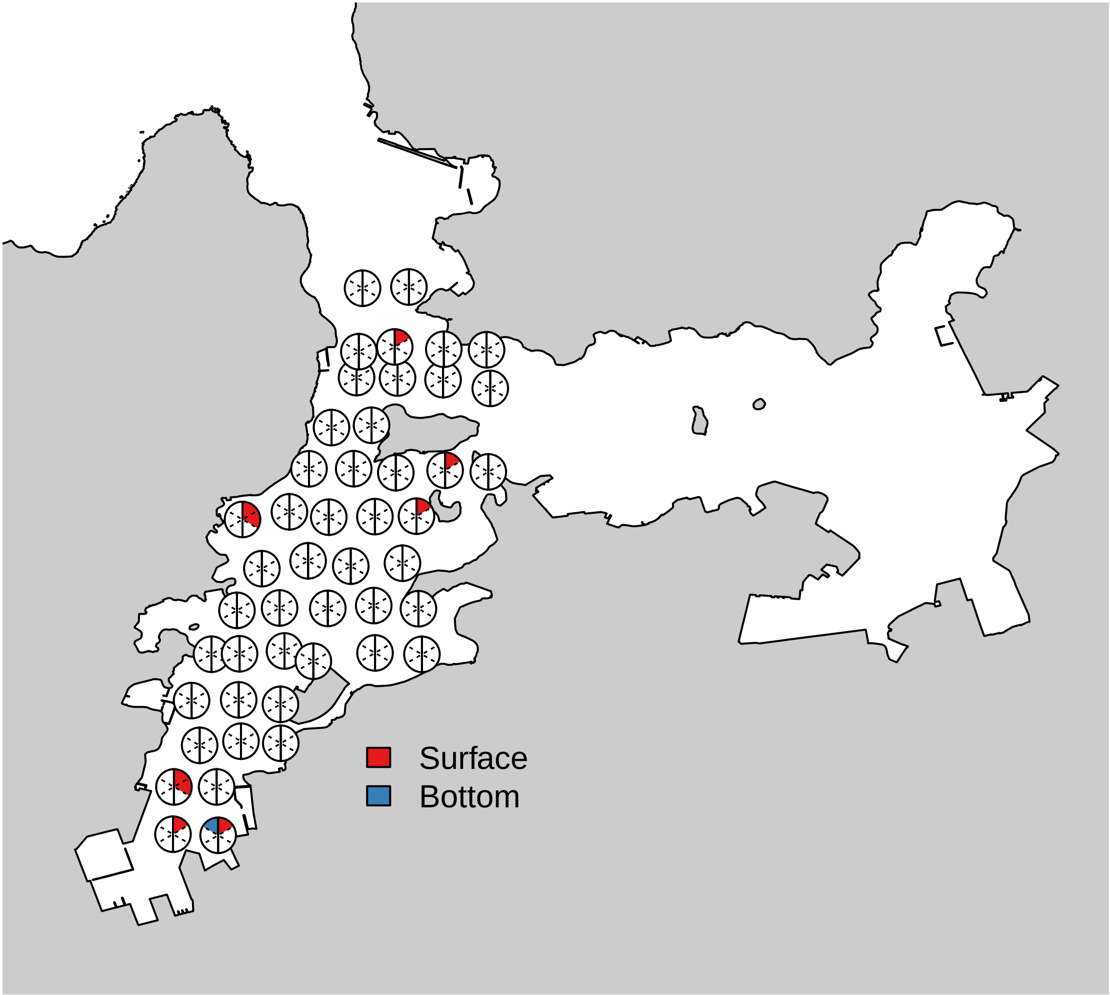

Fig.S1_Scomberomorus_niphonius

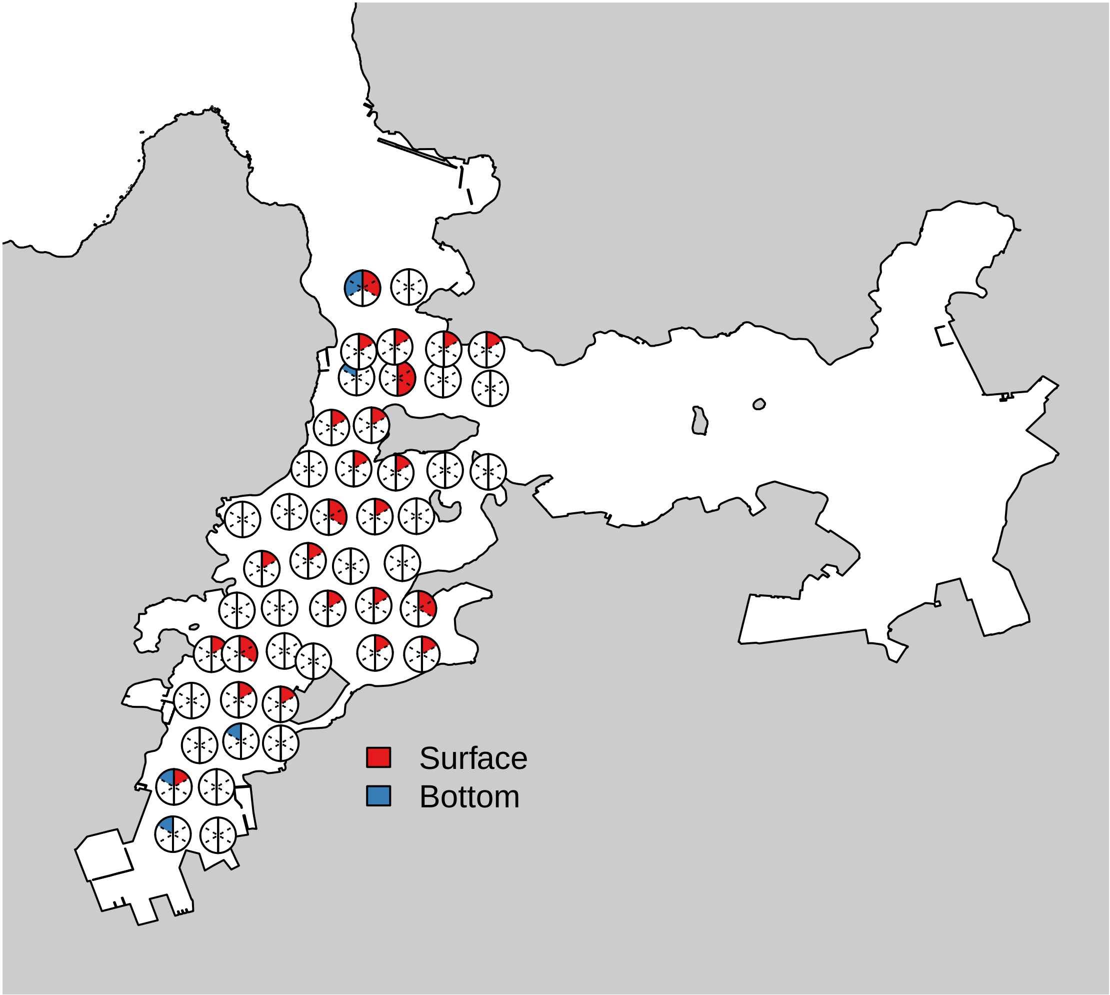

Fig.S1_Sebastes_sp.

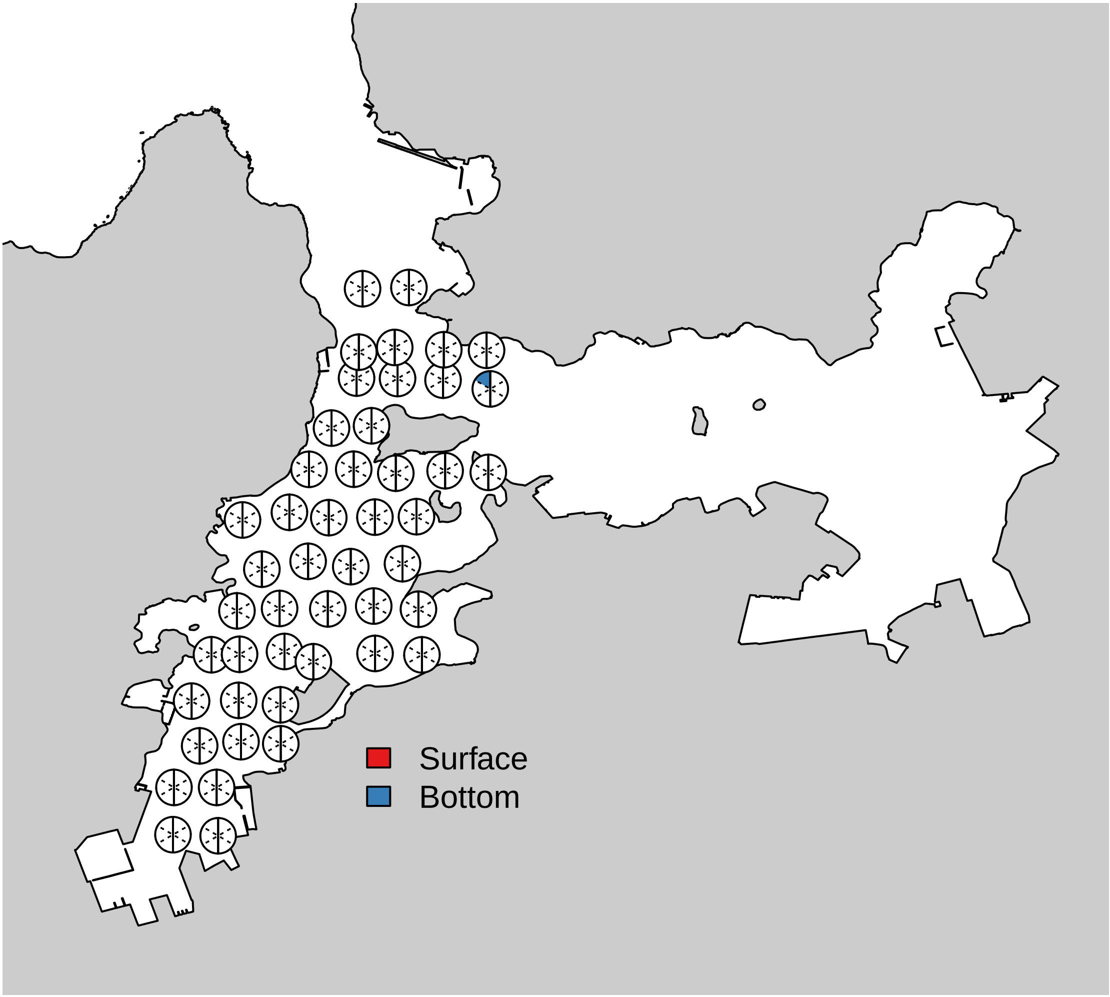

Fig.S1_Sebastiscus_marmoratus

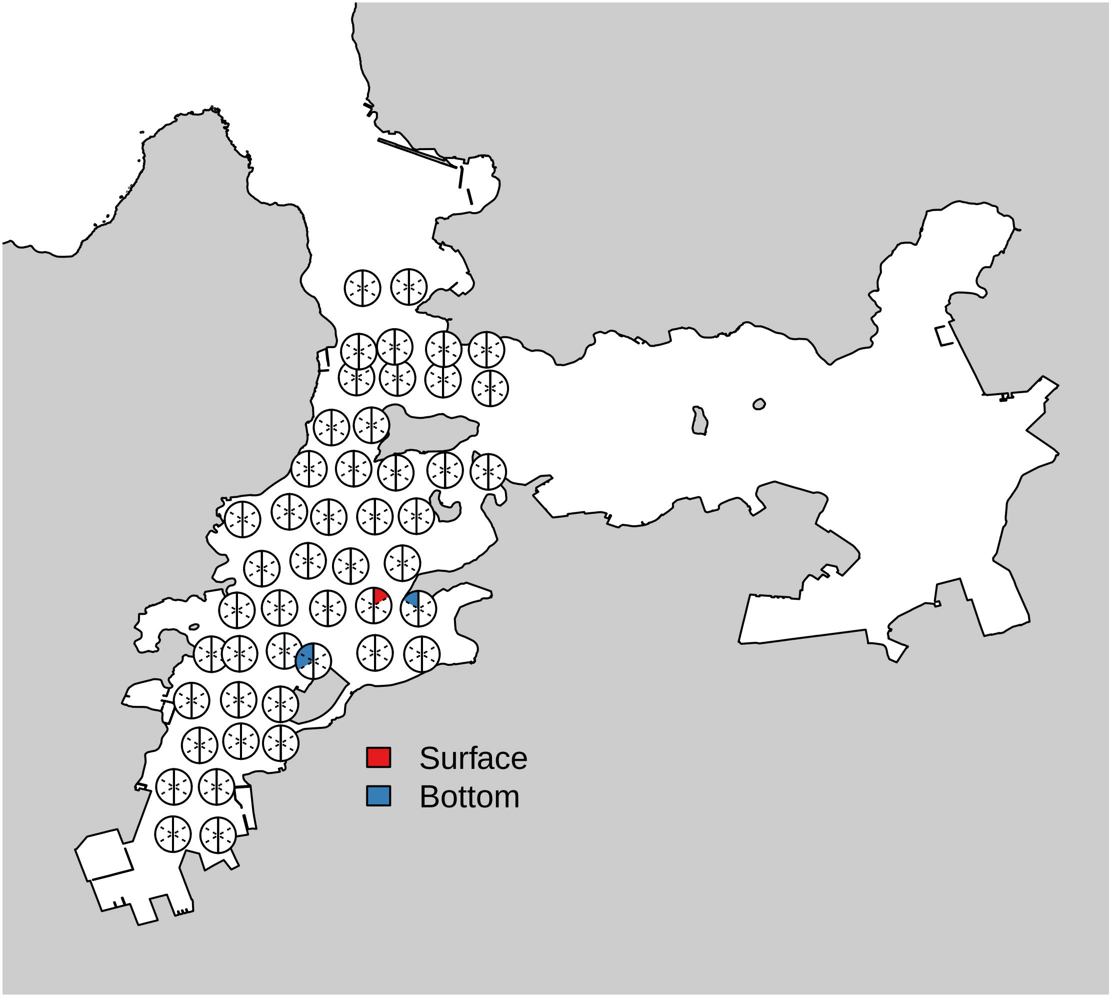

Fig.S1_Sebastiscus_tertius

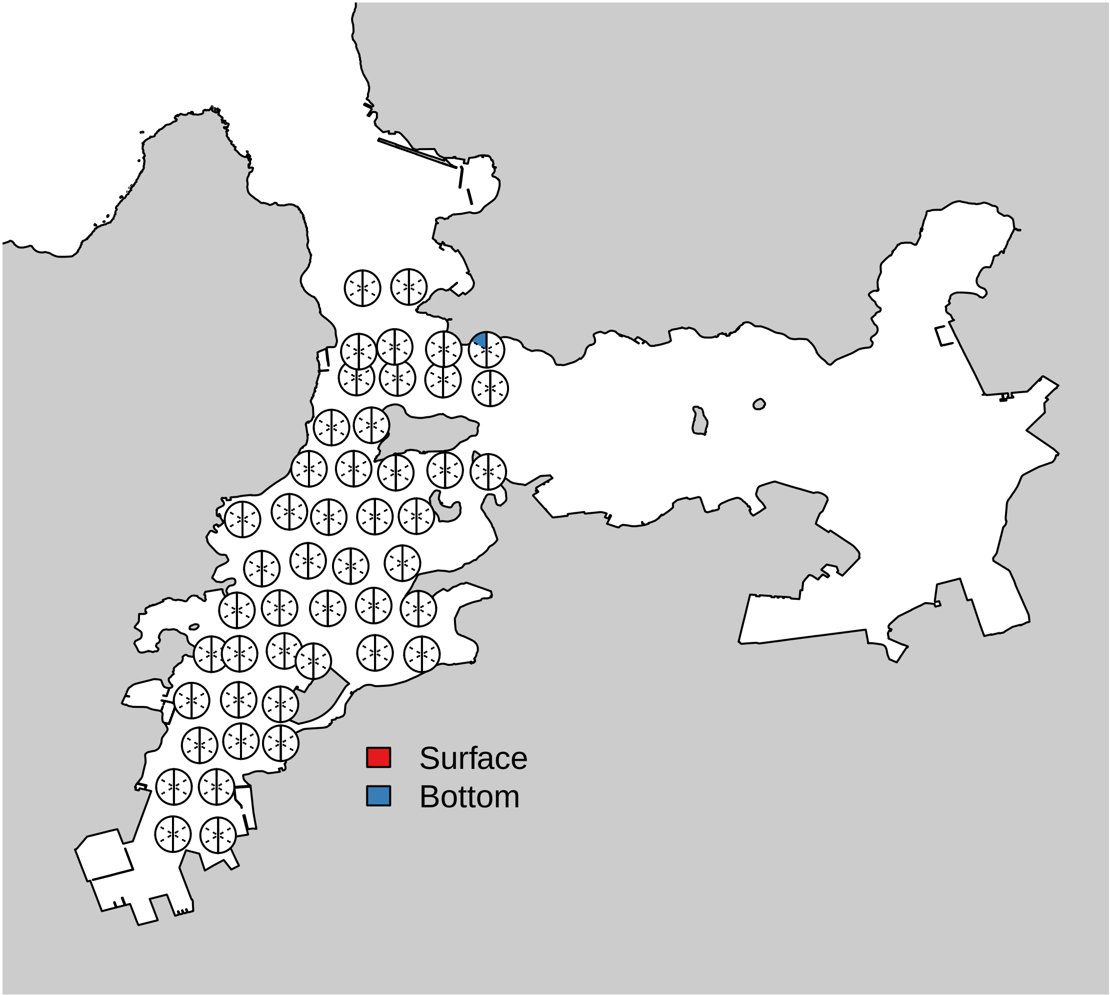

Fig.S1_Seriola_lalandi

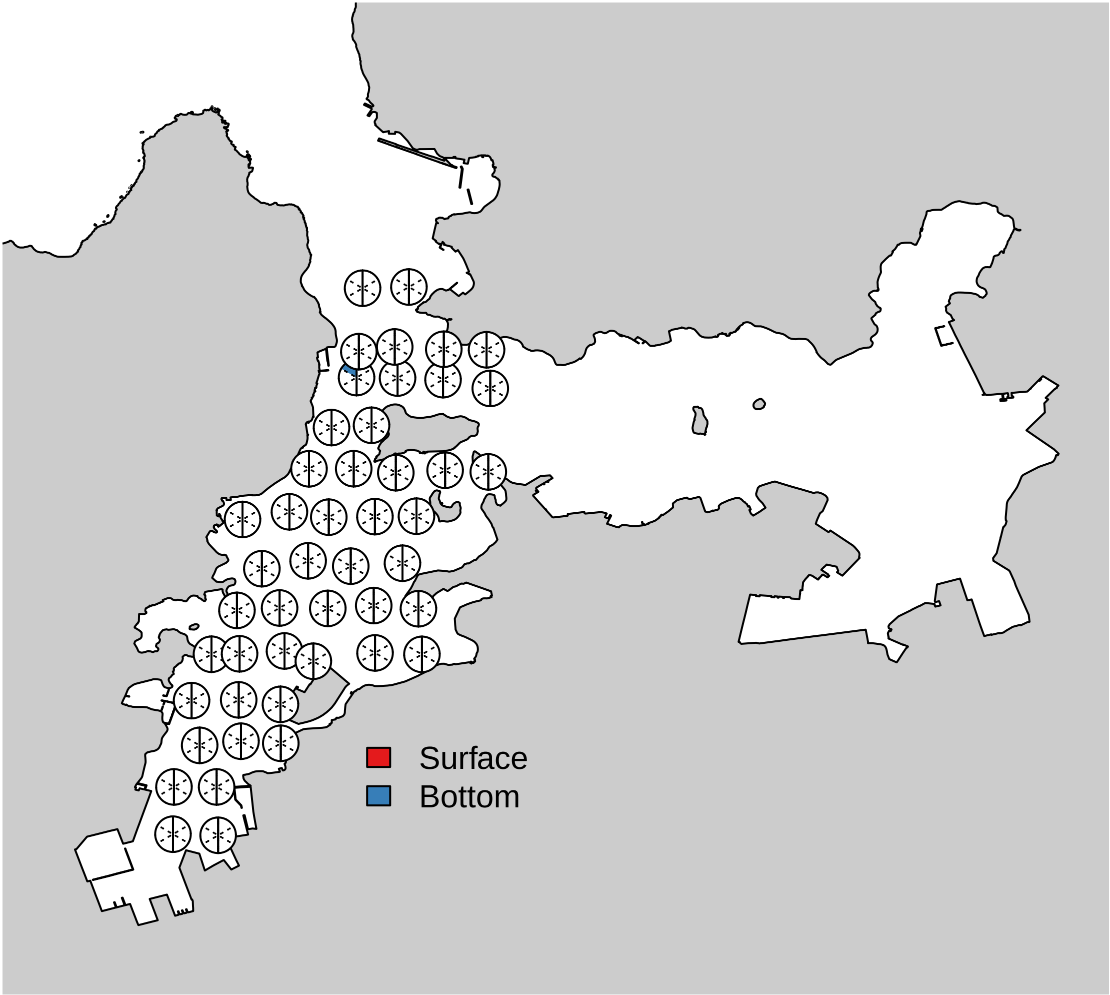

Fig.S1_Seriola_quinqueradiata

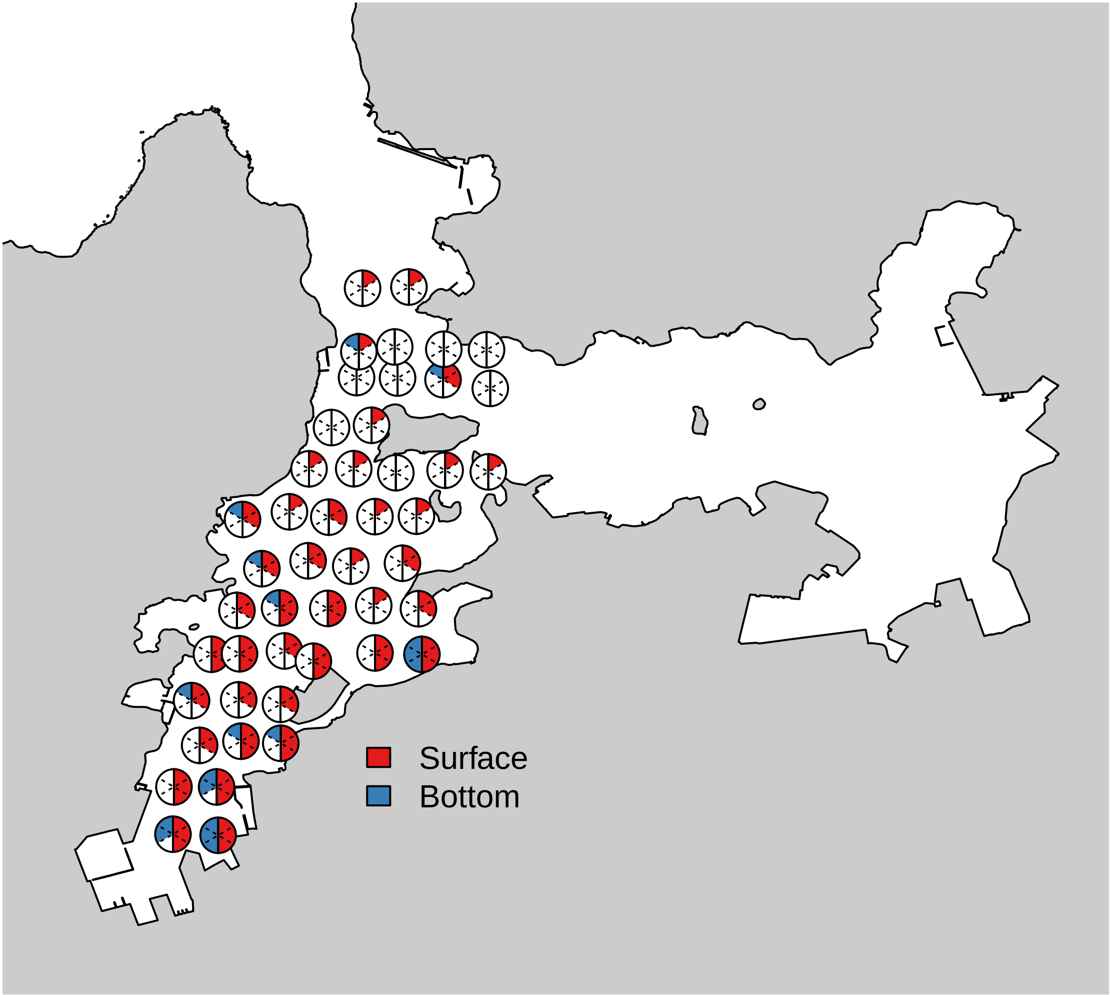

Fig.S1_Sillago_japonica

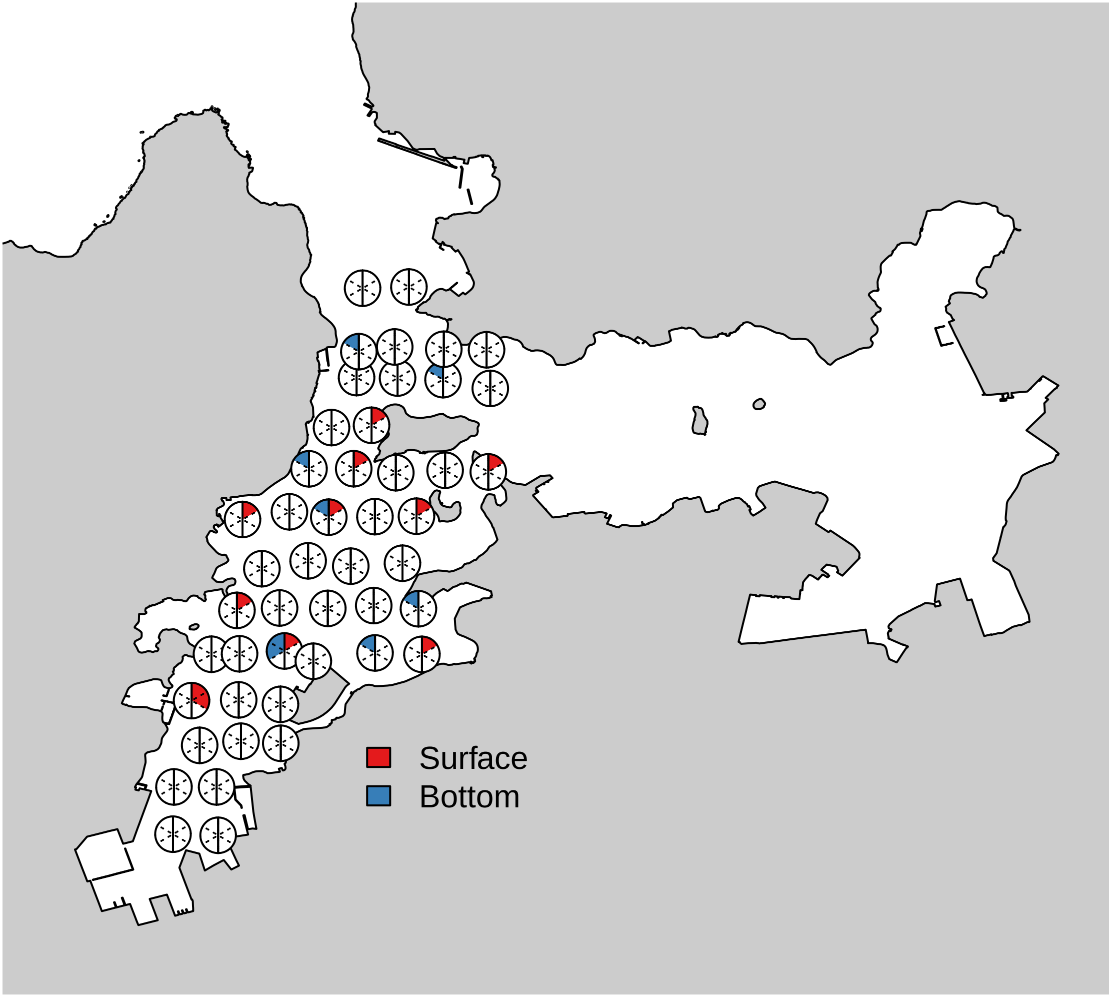

Fig.S1_Sphyraena_pinguis

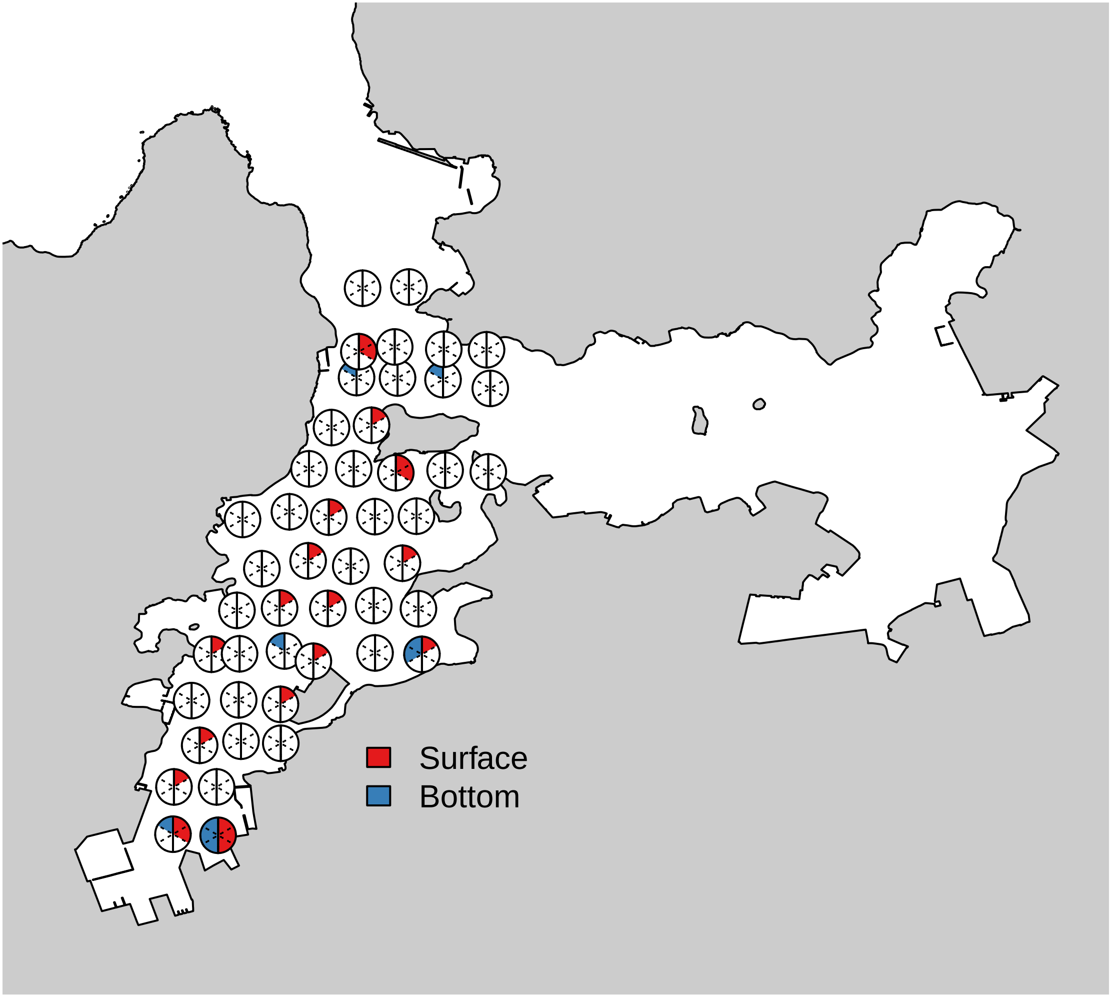

Fig.S1_Stephanolepis_cirrhifer

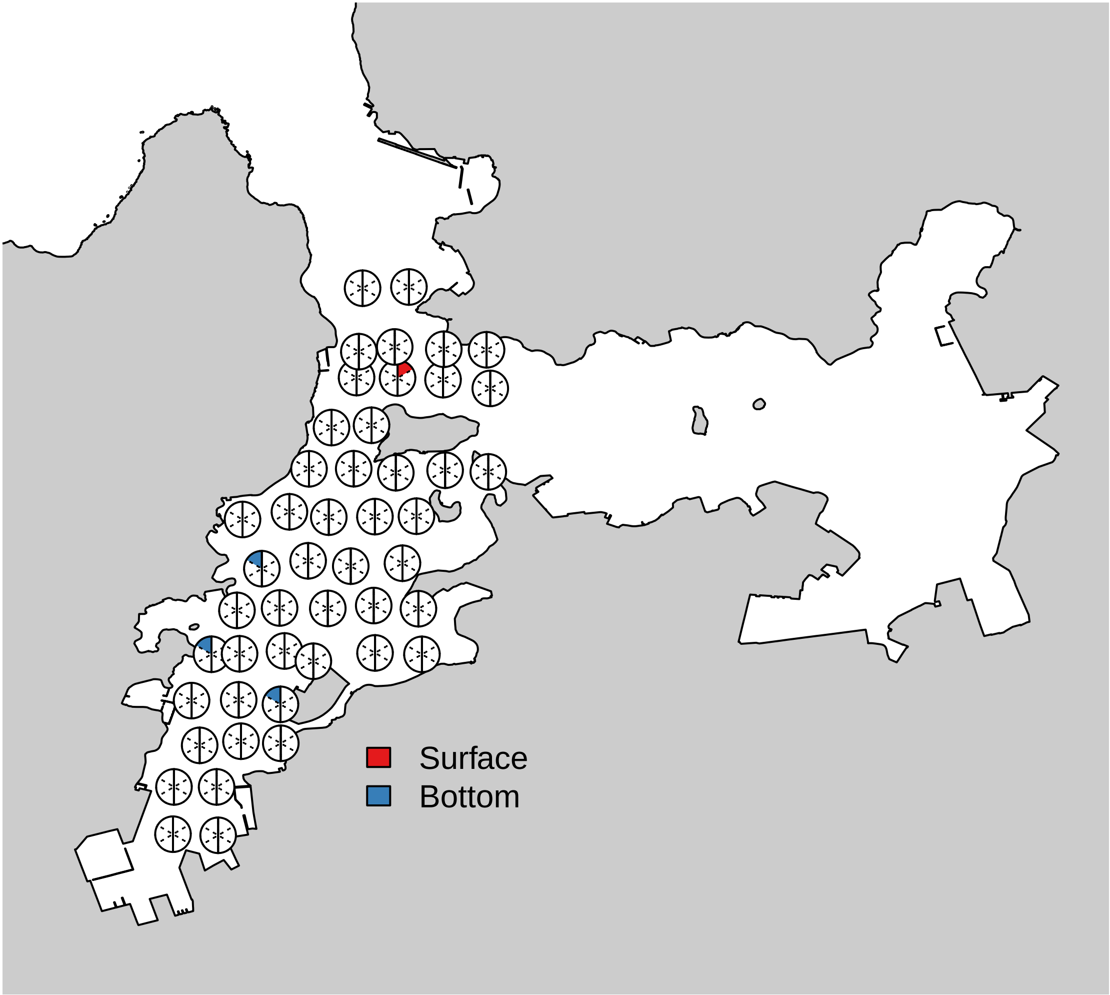

Fig.S1_Strongylura_anastomella

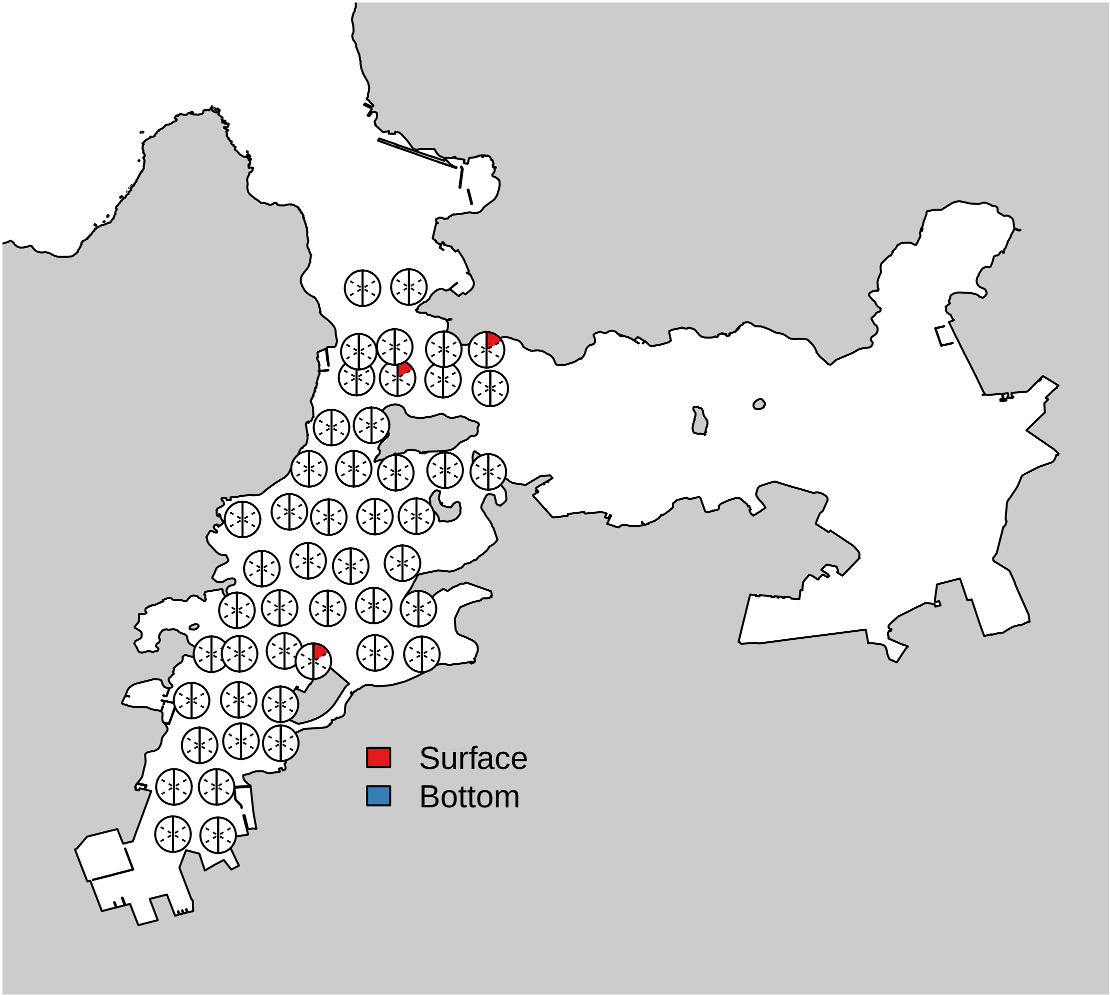

Fig.S1_Takifugu_sp.1

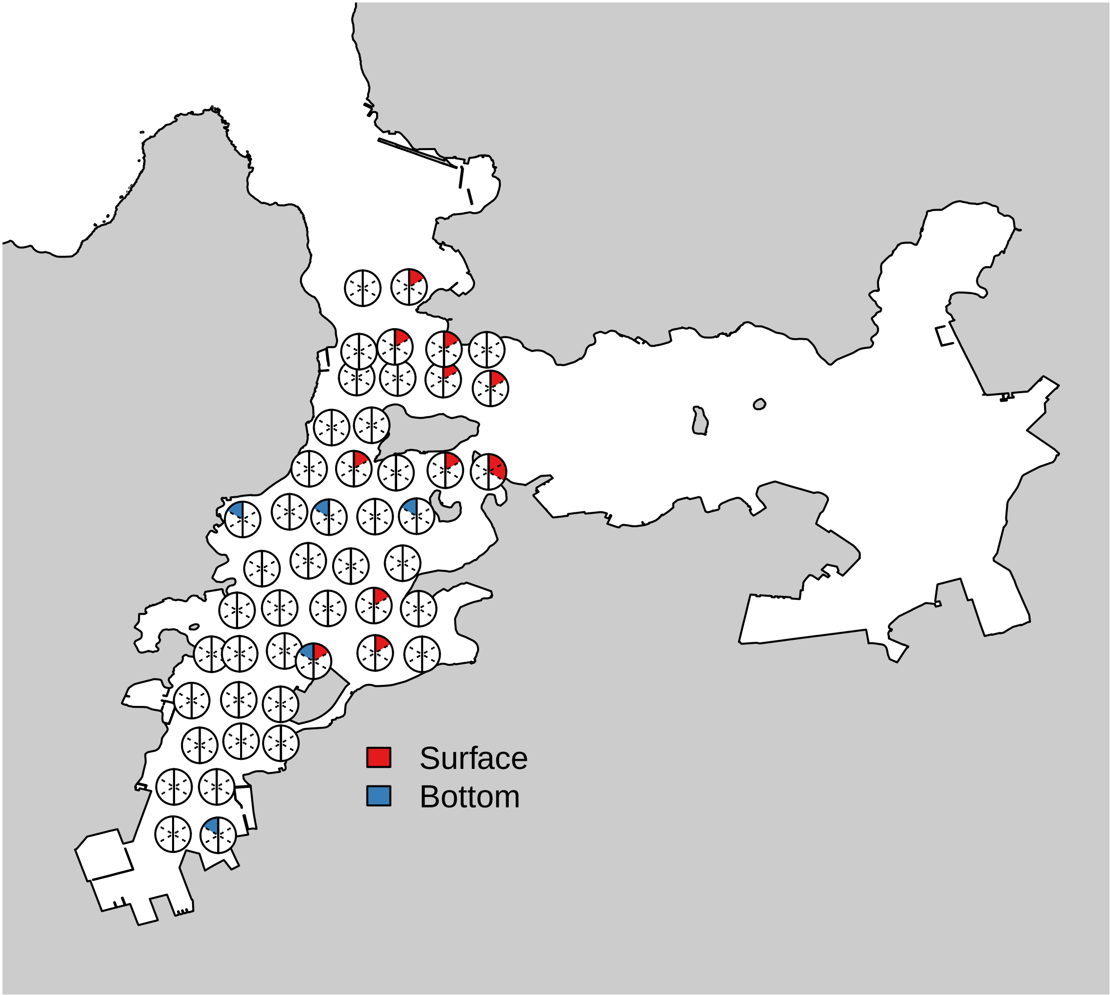

Fig.S1_Takifugu_sp.2

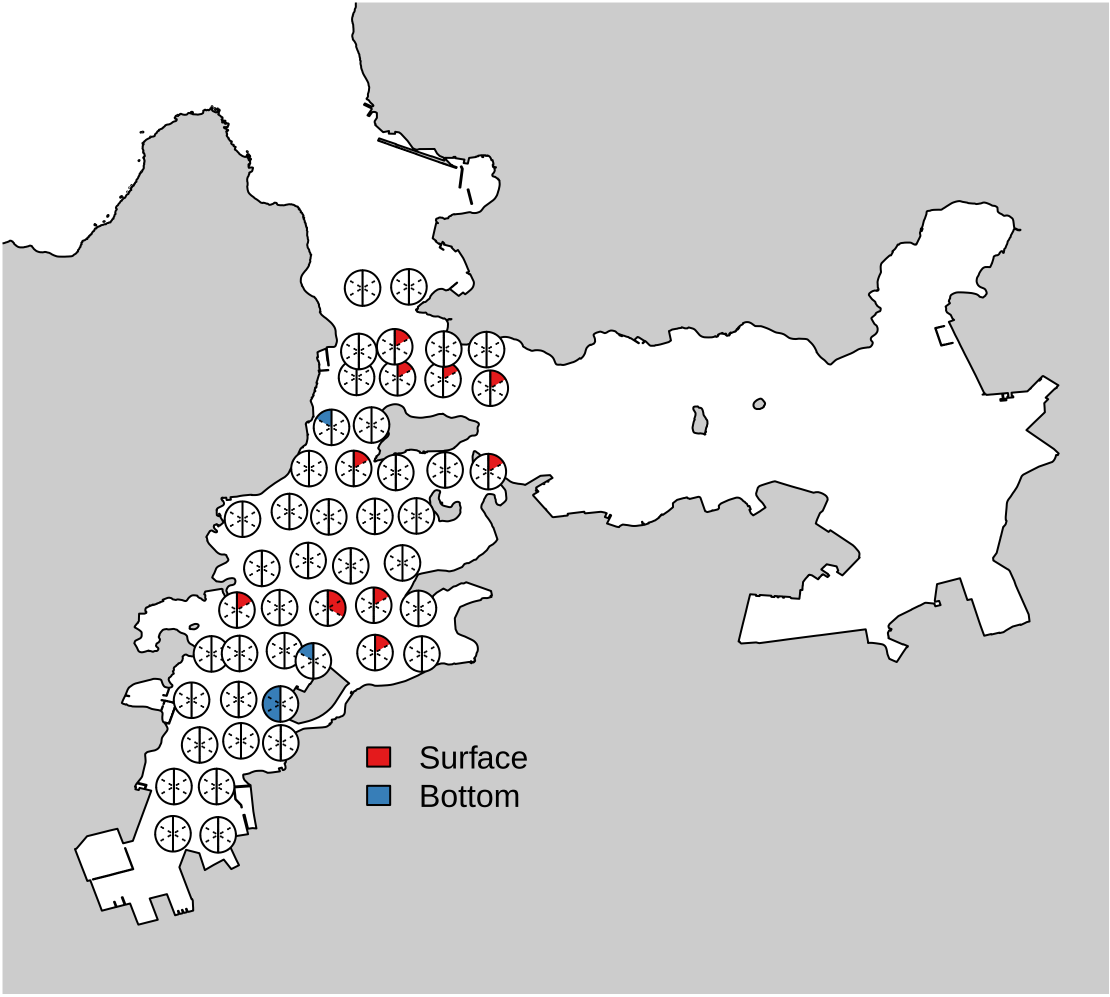

Fig.S1_Takifugu_sp.4

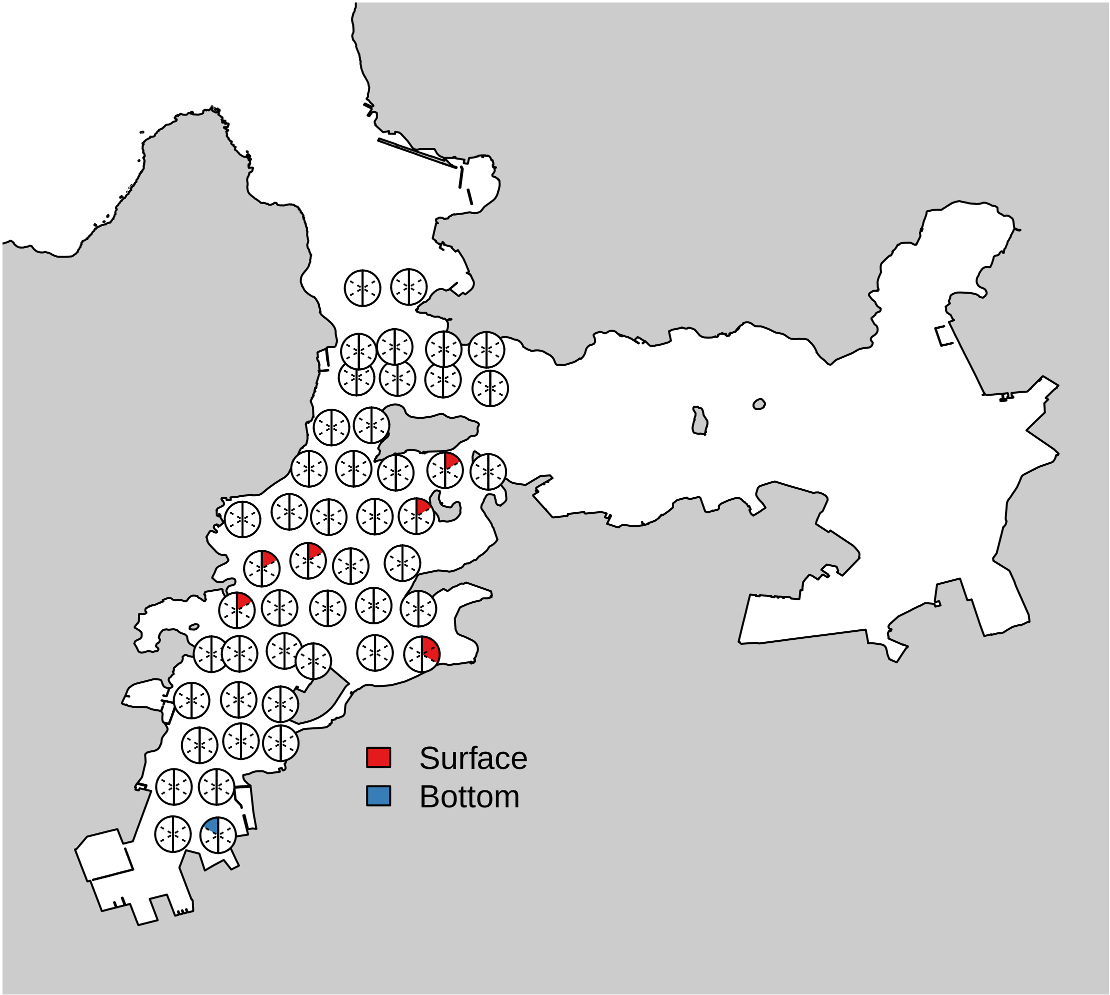

Fig.S1_Tanakius_kitaharae

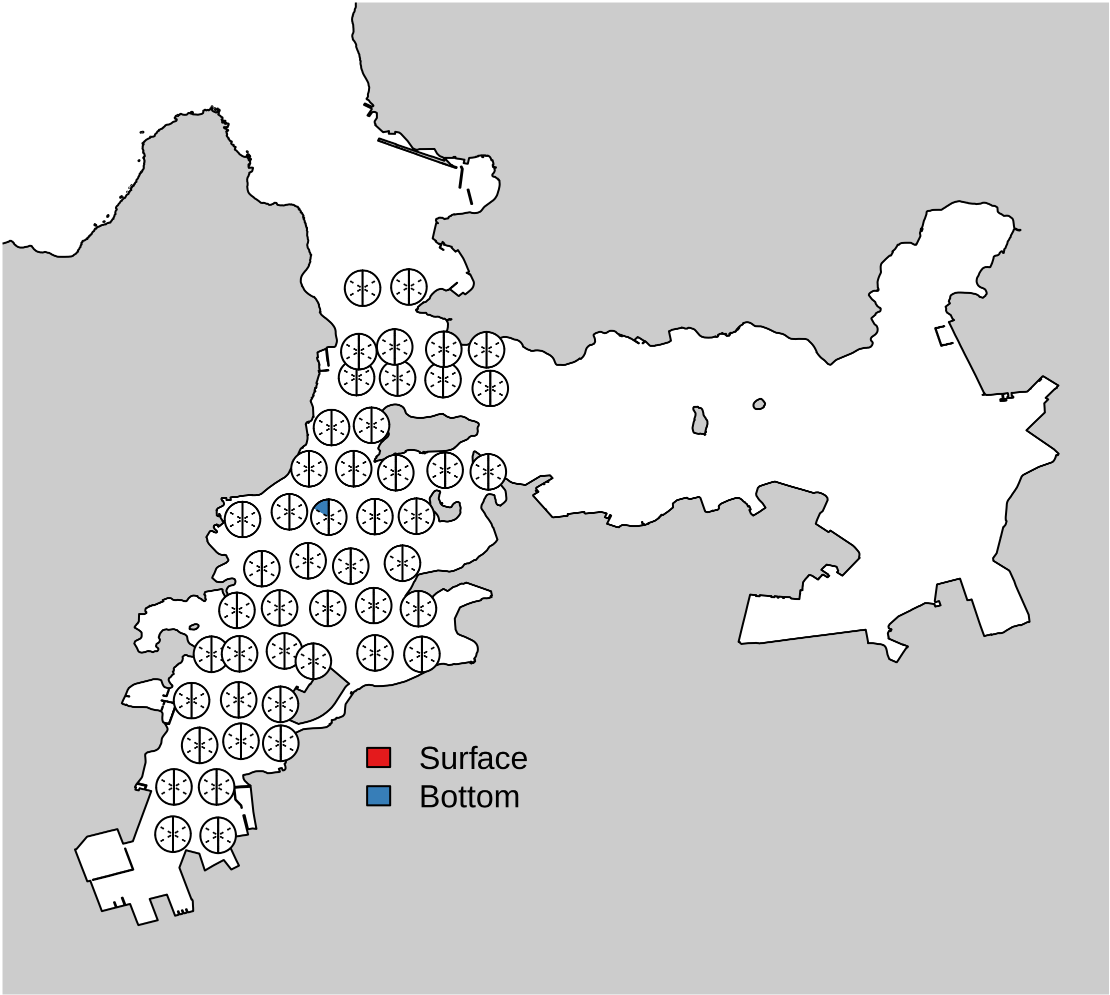

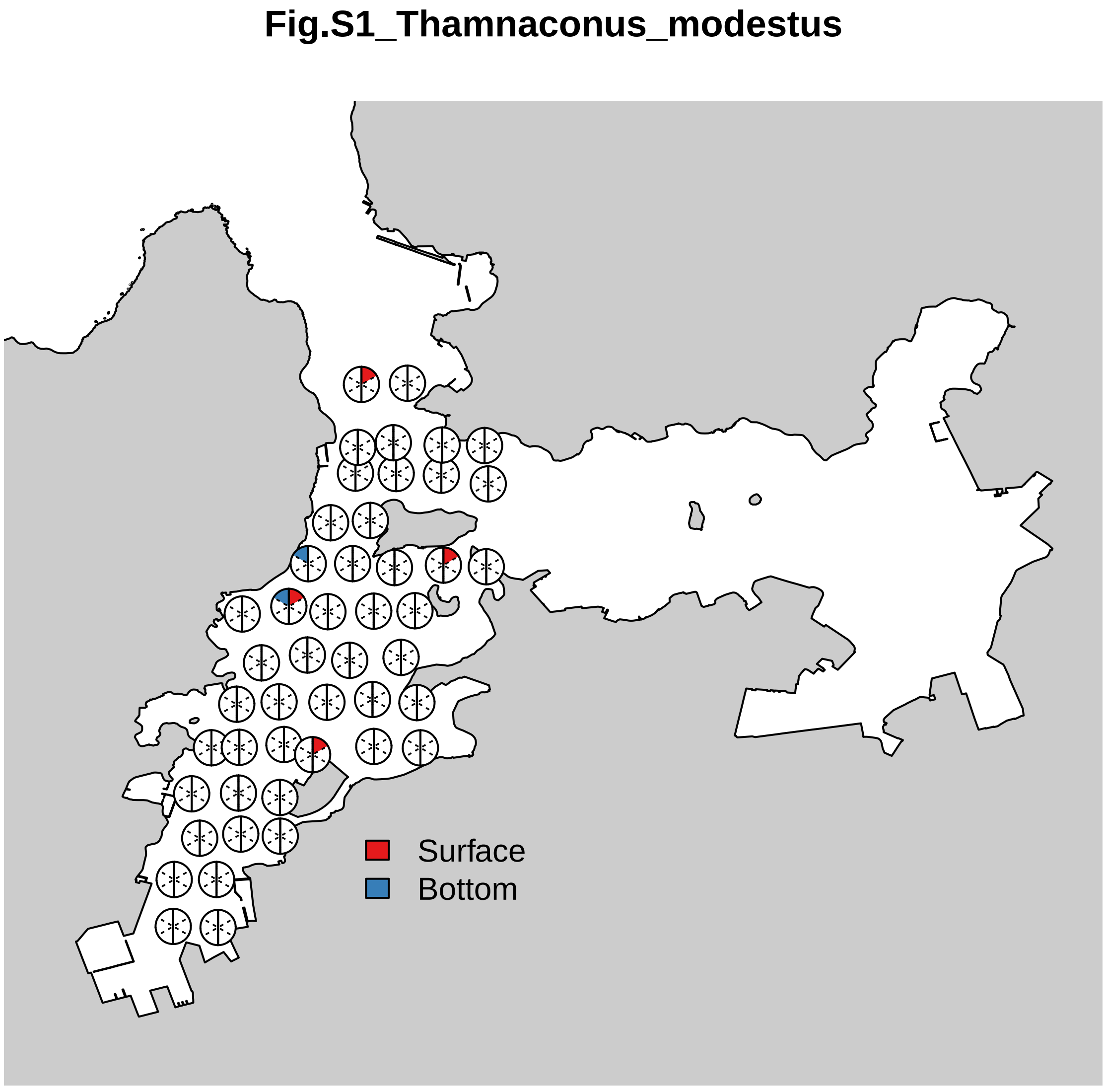
Fig.S1_Thamnaconus_modestus
Surface
Bottom

Fig.S1_Thunnus_orientalis

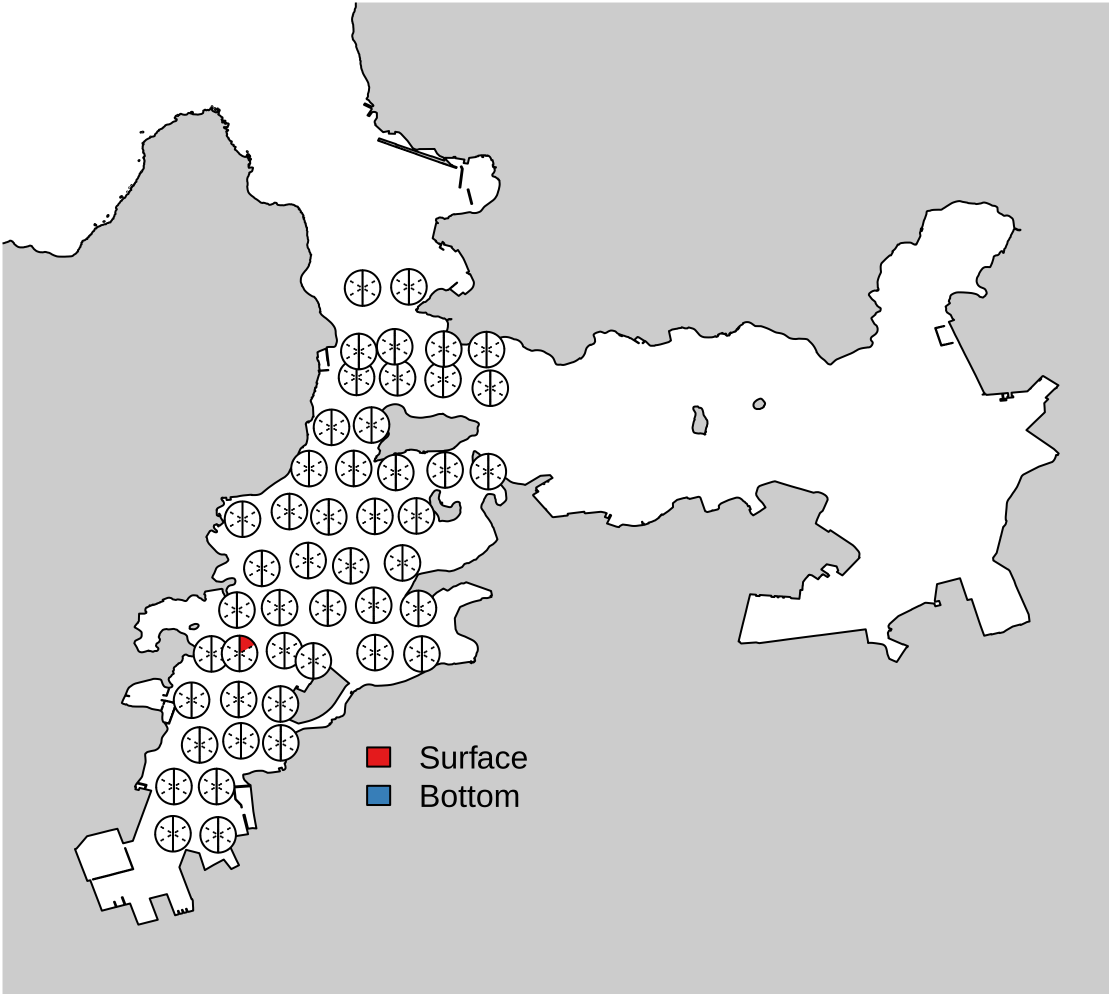

Fig.S1_Thunnus_sp.

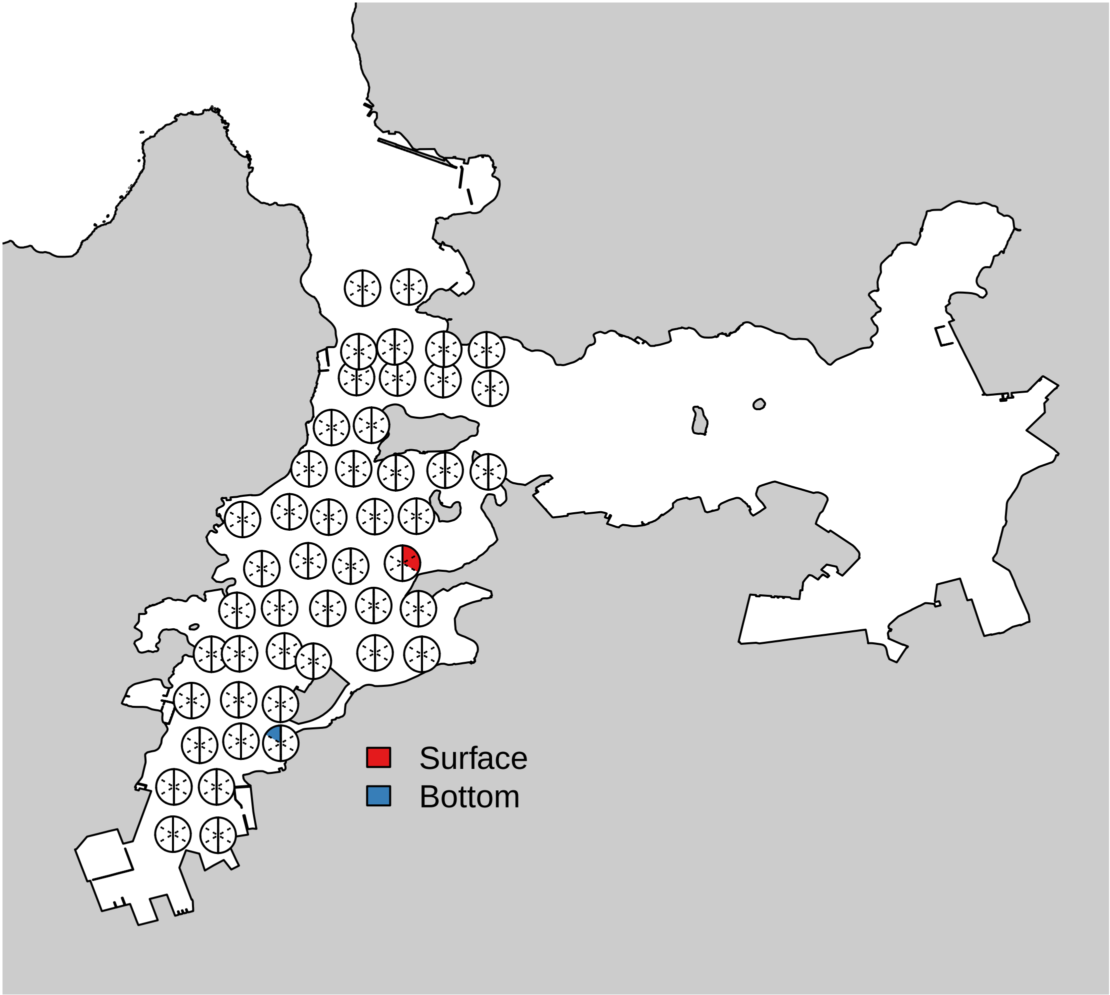

Fig.S1_Trachurus_japonicus

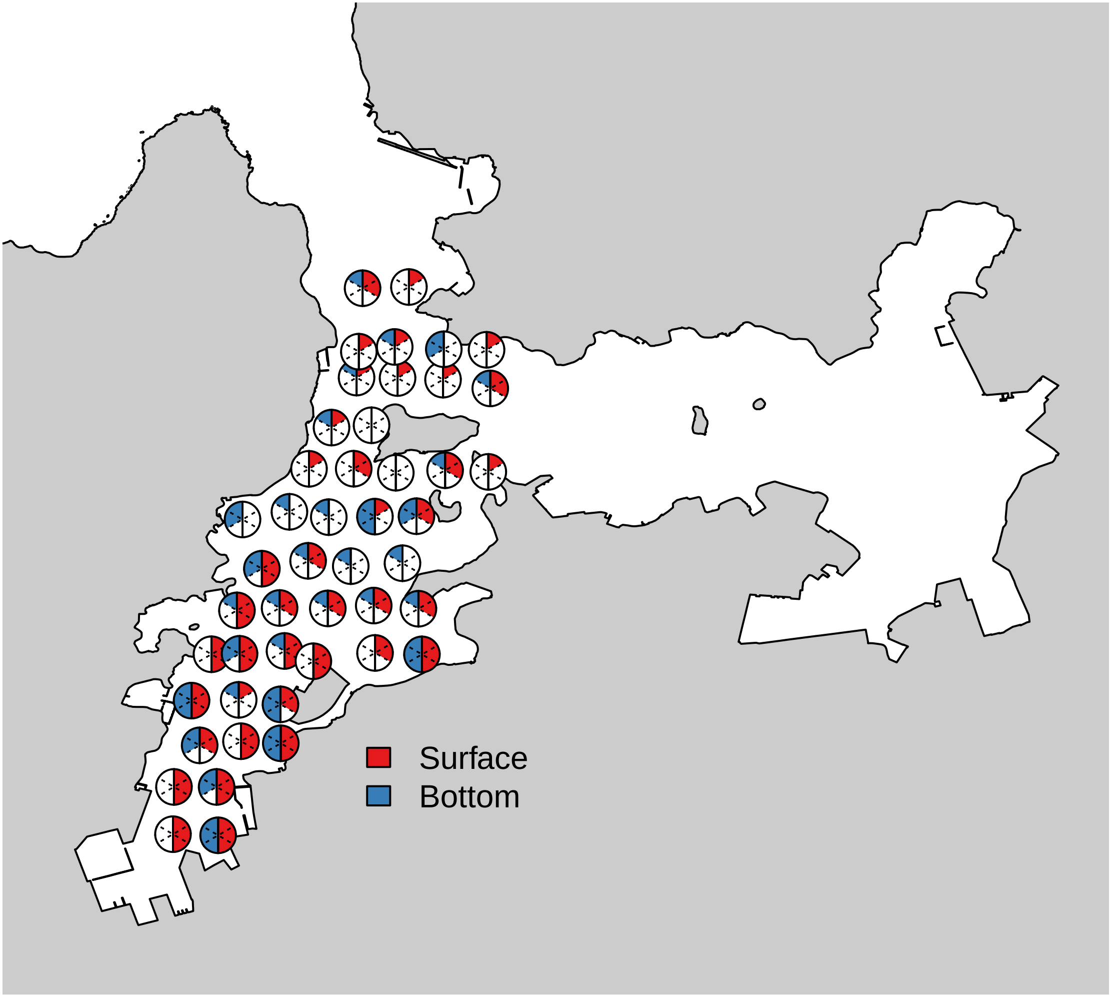

Fig.S1_Trachurus_sp.

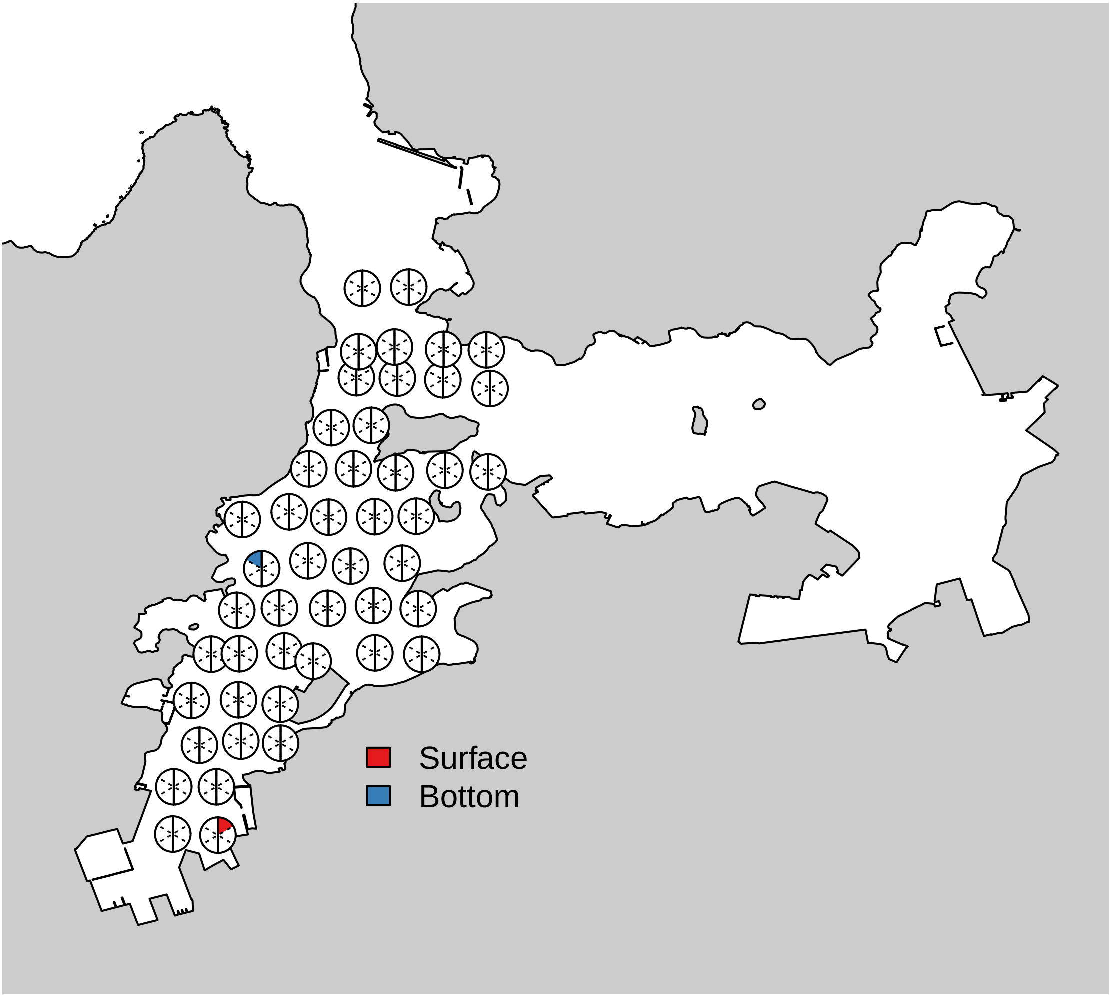

Fig.S1_Trachurus_trachurus

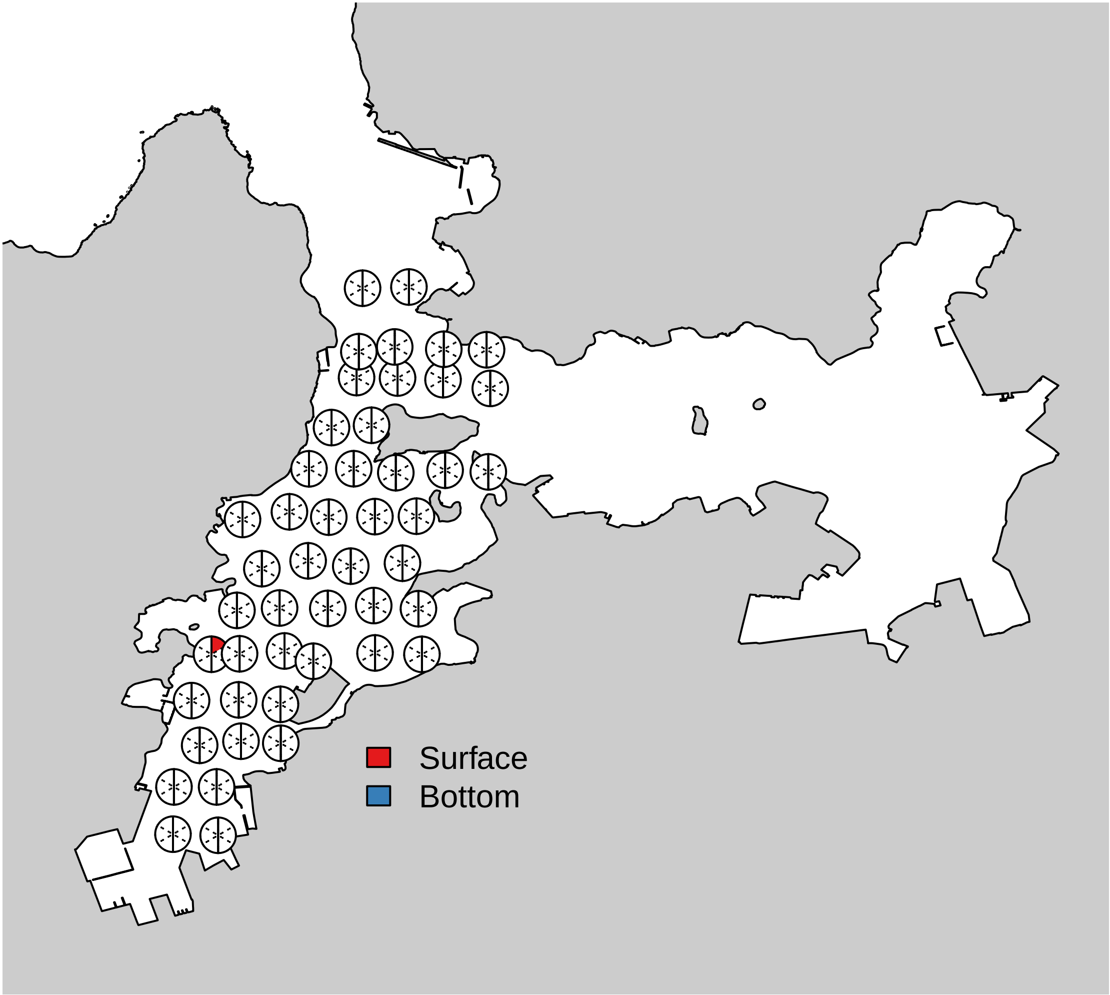

Fig.S1_Tribolodon_hakonensis

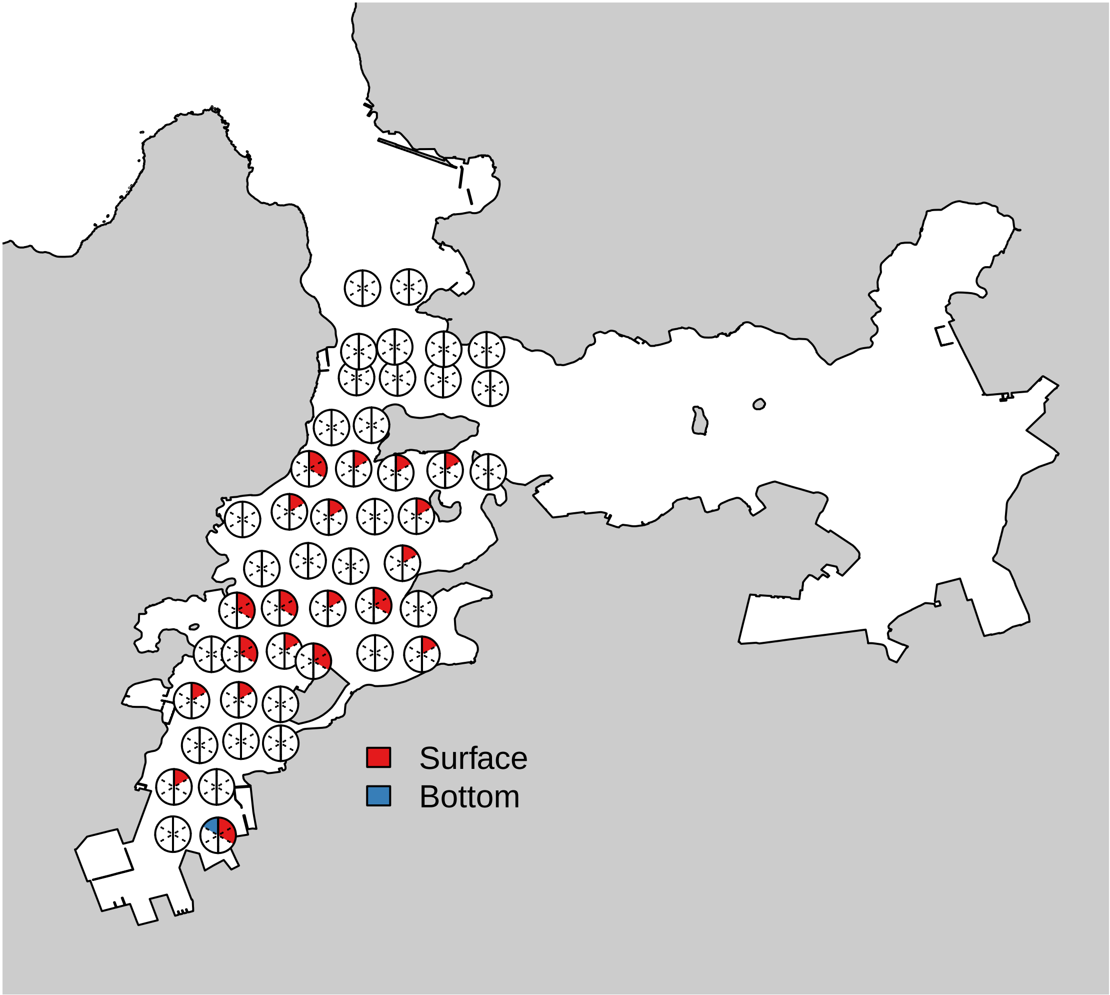

Fig.S1_Trichiurus_japonicus

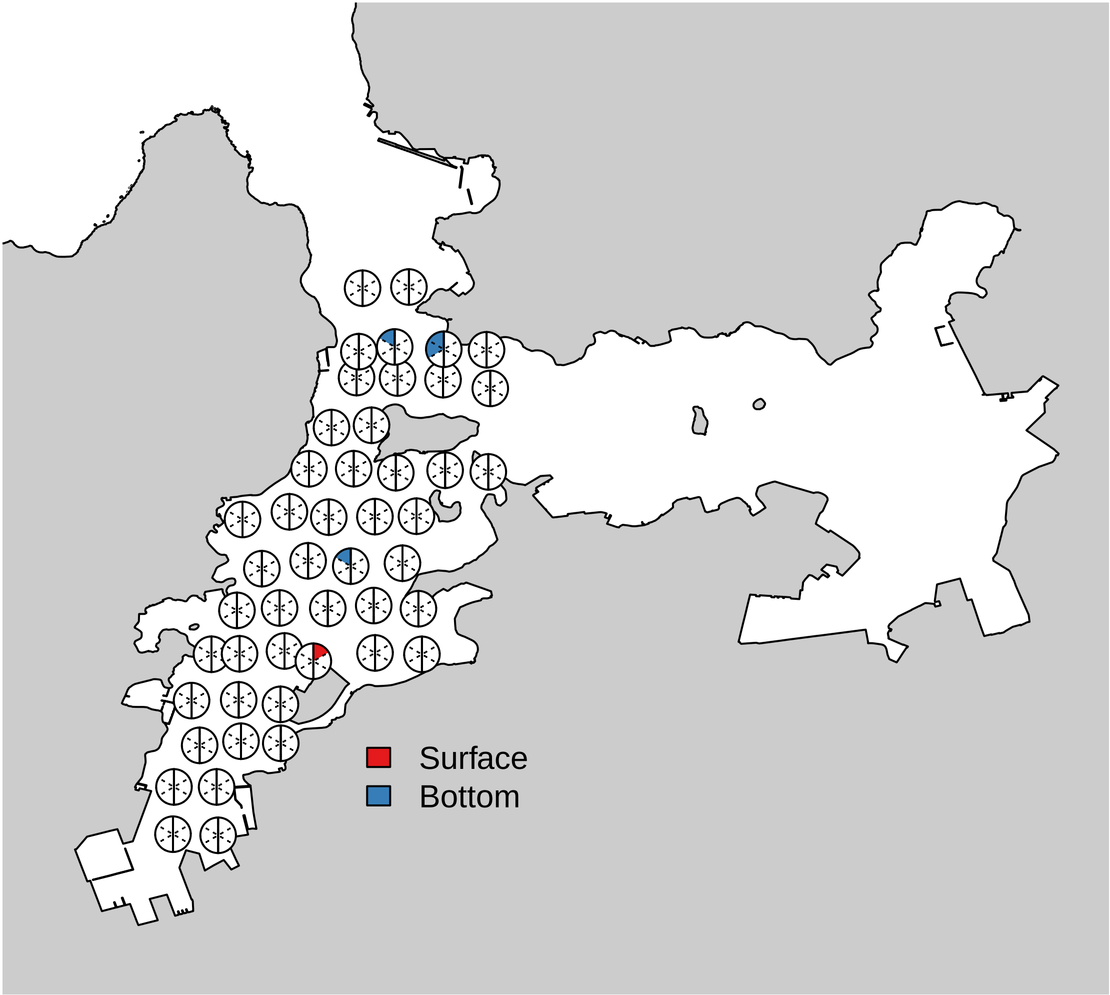

Fig.S1_Tridentiger_sp.1

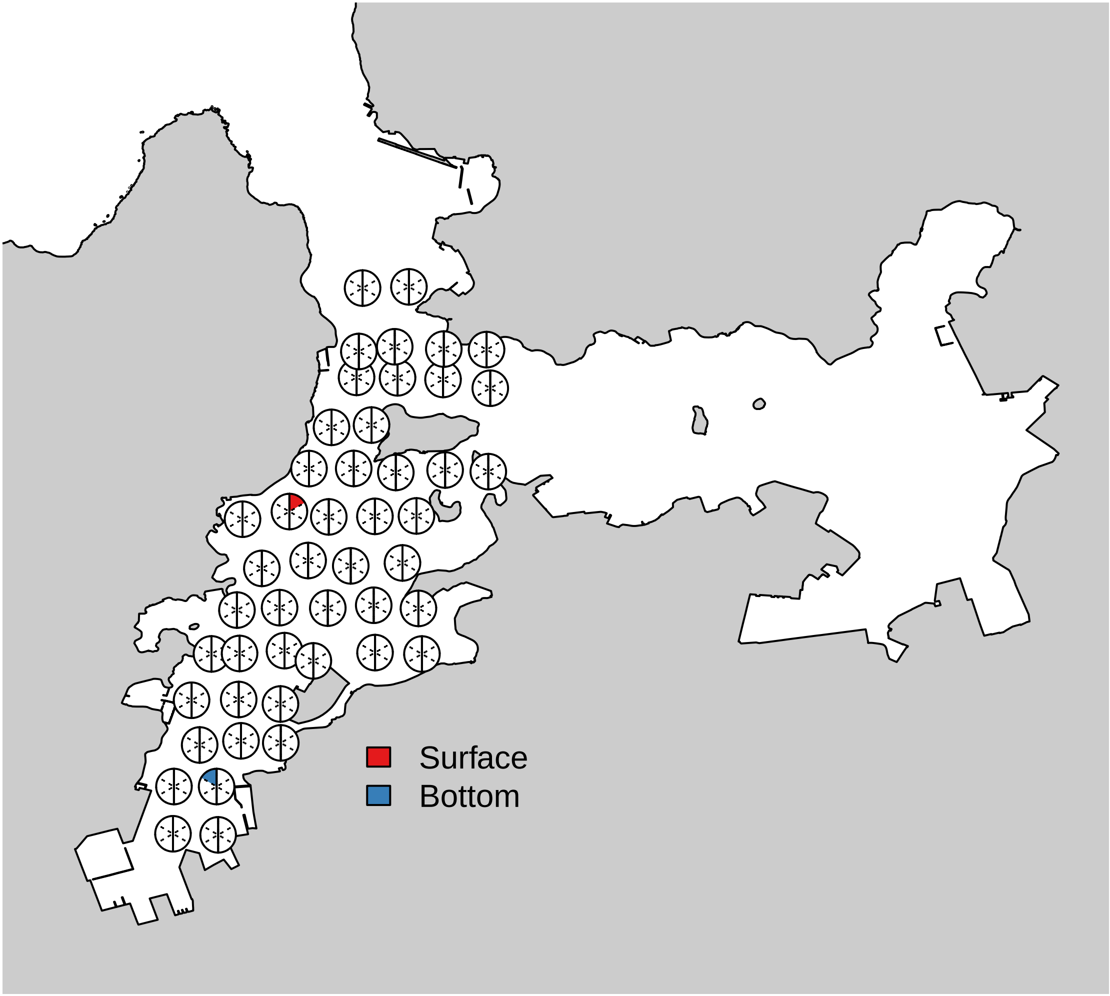

Fig.S1_Tridentiger_sp.2

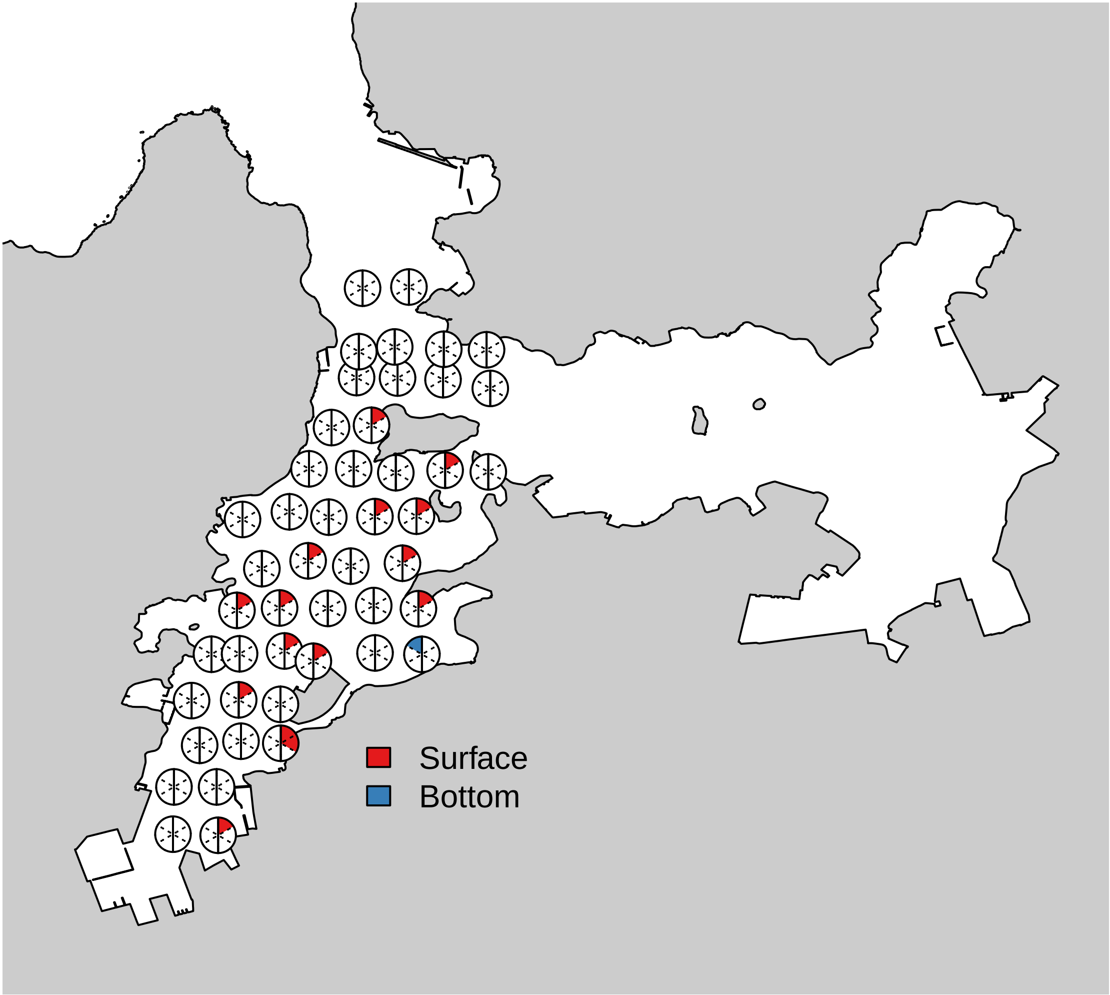

Fig.S1_Tridentiger_trigonocephalus

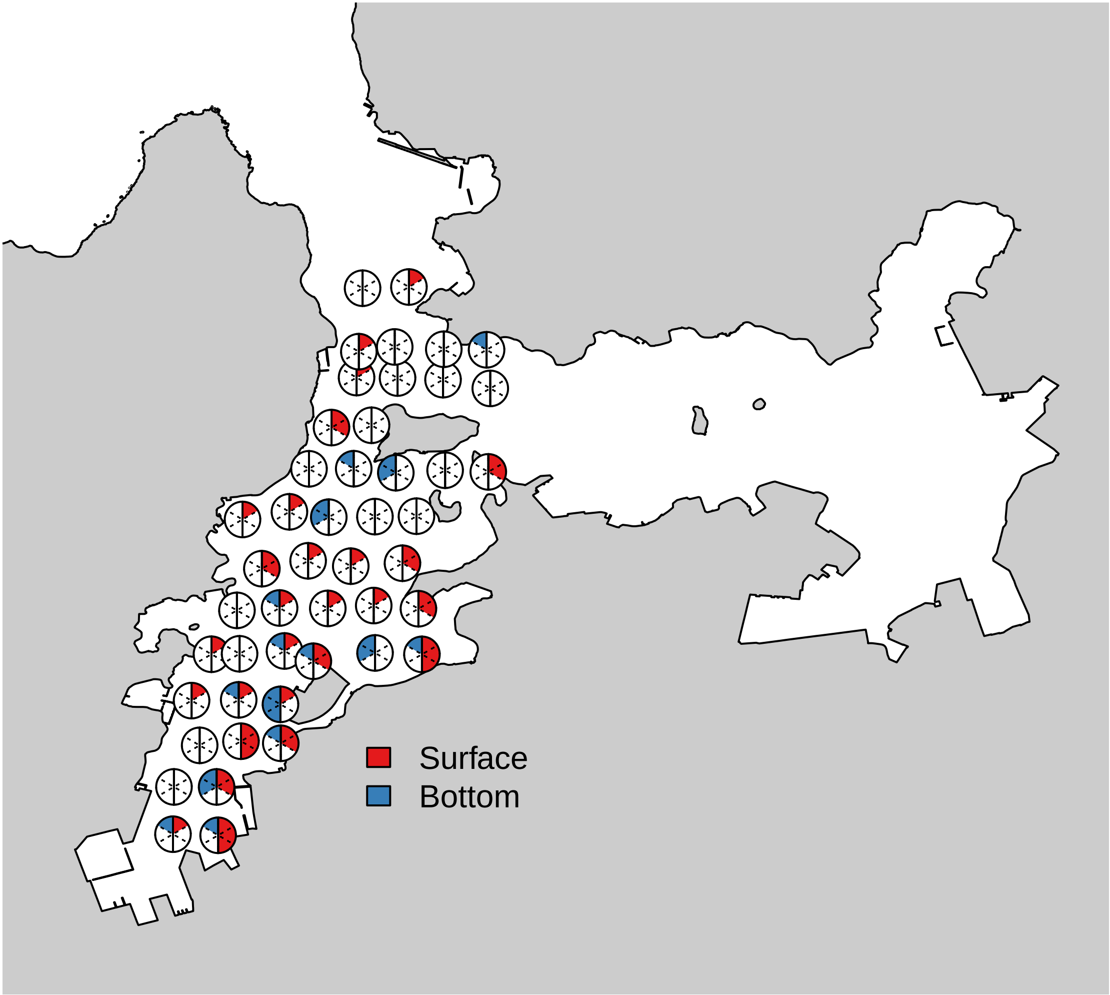

Fig.S1_Upeneus_japonicus

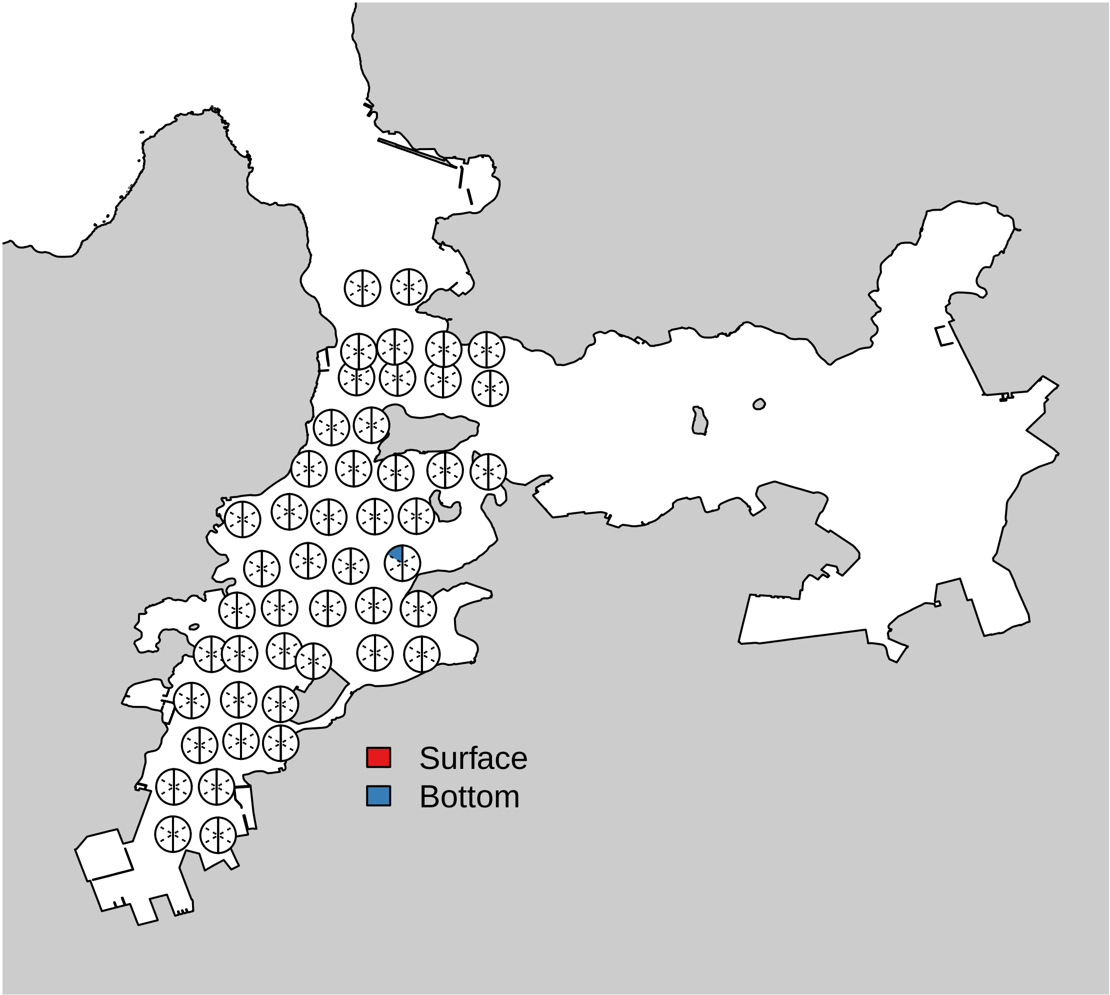

Fig.S1_Zacco_platypus

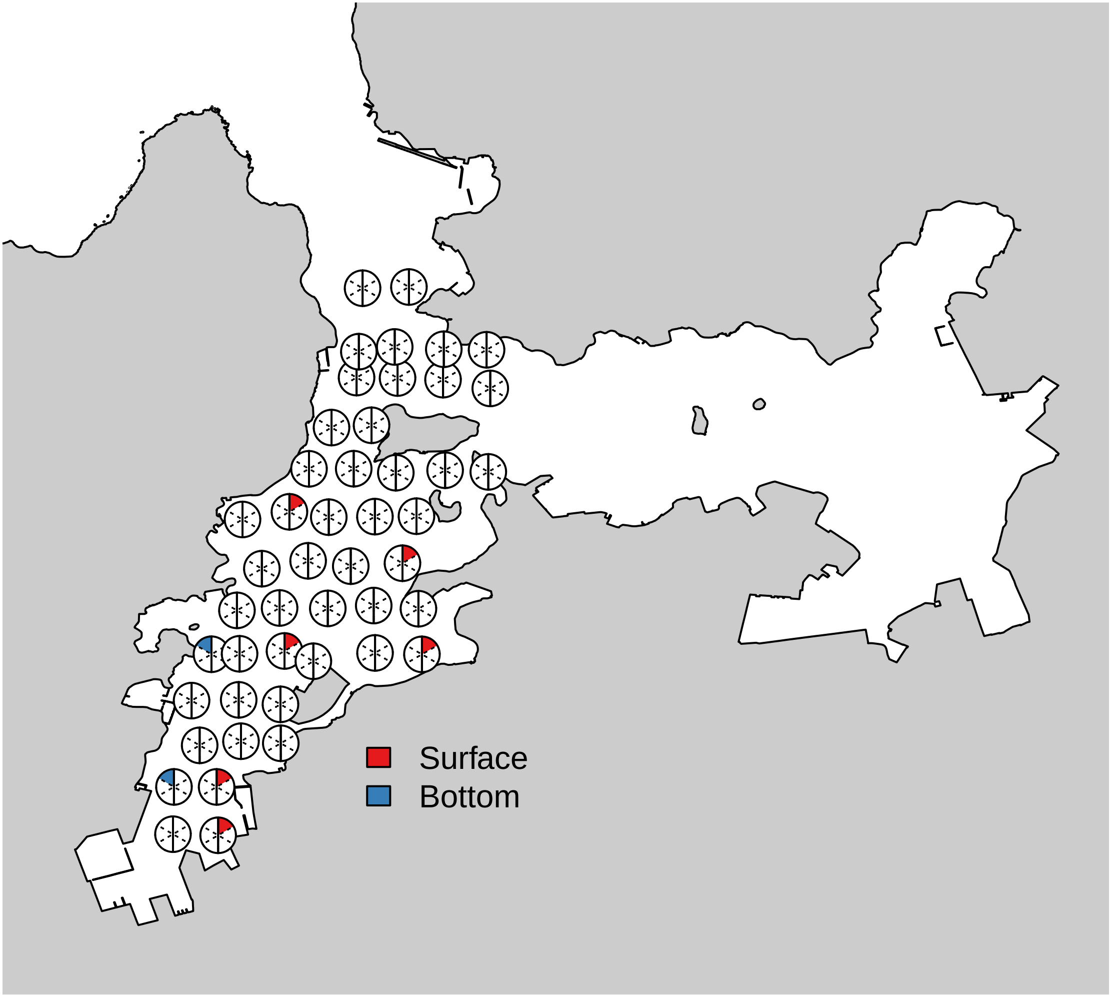

Fig.S1_Zoarchias_major

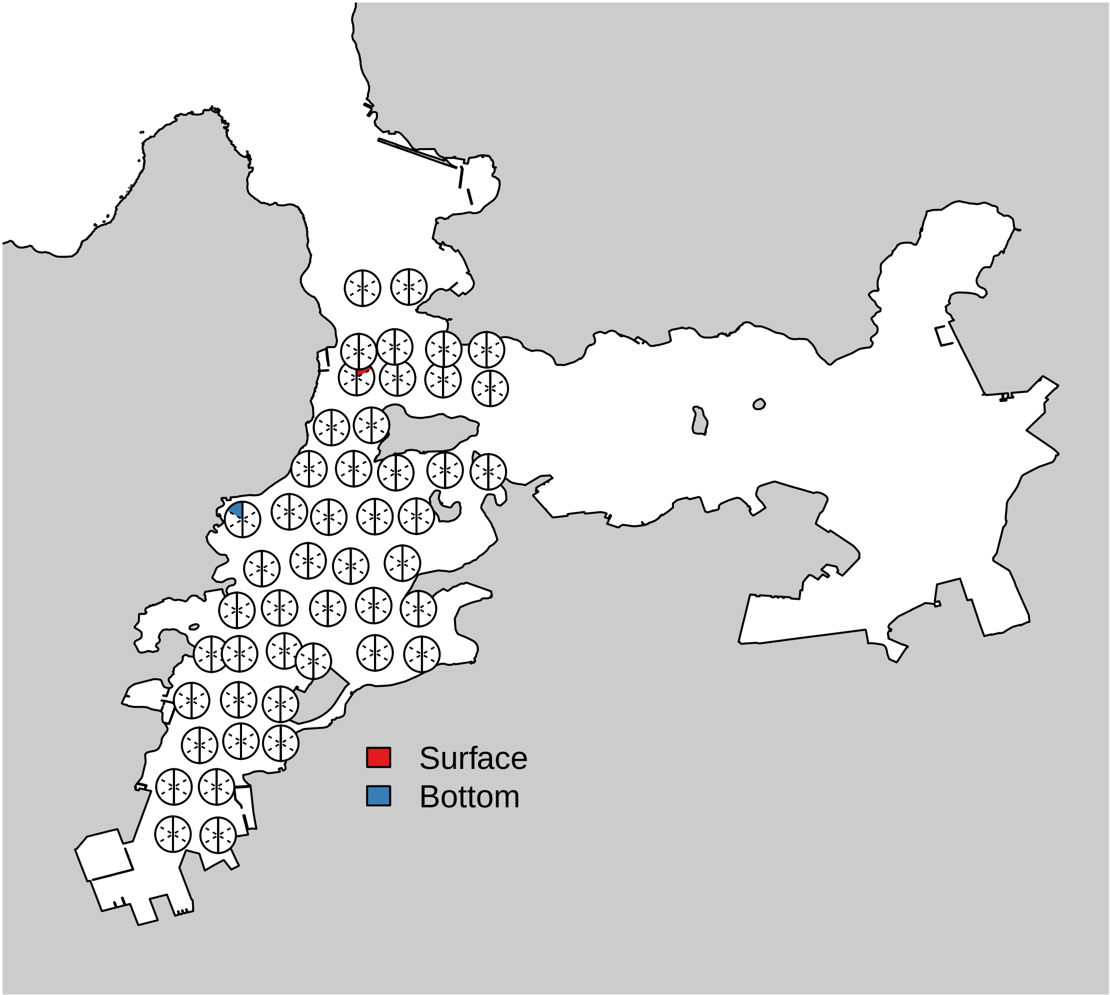

Supplement: Supplementary Information [file srep40368-s1.pdf]
